# Carbonylation of Polyfluorinated Alkylbenzenes and Benzocycloalkenes at the Benzyl C-F and C-Cl Bonds Under the Action of CO/SbF_5_

**DOI:** 10.3390/molecules30040931

**Published:** 2025-02-17

**Authors:** Yaroslav V. Zonov, Siqi Wang, Vladislav V. Komarov, Victor M. Karpov, Dmitriy A. Parkhomenko, Tatyana V. Mezhenkova

**Affiliations:** 1 N. N. Vorozhtsov Novosibirsk Institute of Organic Chemistry SB RAS, Lavrentiev Ave. 9, Novosibirsk 630090, Russia; vkomarov@nioch.nsc.ru (V.V.K.); karpov@nioch.nsc.ru (V.M.K.); parkhomenko@nioch.nsc.ru (D.A.P.); mtv@nioch.nsc.ru (T.V.M.); 2 Department of Natural Sciences, Novosibirsk State University, Pirogov Str. 1, Novosibirsk 630090, Russia; 3 Daqing Petrochemical Research Center, PetroChina Company Limited, Daqing 163714, China; wsq459@petrochina.com.cn

**Keywords:** carbonylation, carbon monoxide, benzyl chloride, benzyl fluoride, polyfluorinated compound, carboxylic acid, antimony pentafluoride

## Abstract

The carbonylation at the benzyl C-Hal bonds (Hal = F, Cl) of a number of polyfluorinated alkylbenzenes and benzocycloalkenes using carbon monoxide in the presence of SbF_5_ is described. The reaction provided the corresponding *α*-arylcarboxylic acids or their methyl esters following aqueous or methanol treatment. The products of double carbonylation were obtained from bis(chloromethyl)tetrafluorobenzenes and benzal fluorides. For benzal chloride derivatives, the possibility of selective mono- or dicarbonylation was shown to depend on the amount of antimony pentafluoride. In the case of polyfluorinated secondary benzyl halides with a hydrogen atom at the α-carbon atom and vicinal fluorine atoms, the addition of CO was found to be accompanied by the elimination of HF, resulting in *α*,*β*-unsaturated *α*-arylcarboxylic acids. The double elimination of HF during the carbonylation of 1,4-dichloro-2,2,3,3,5,6,7,8-octafluorotetralin yielded dimethyl perfluoronaphthalene-1,4-dicarboxylate.

## 1. Introduction

**Scheme 1:**
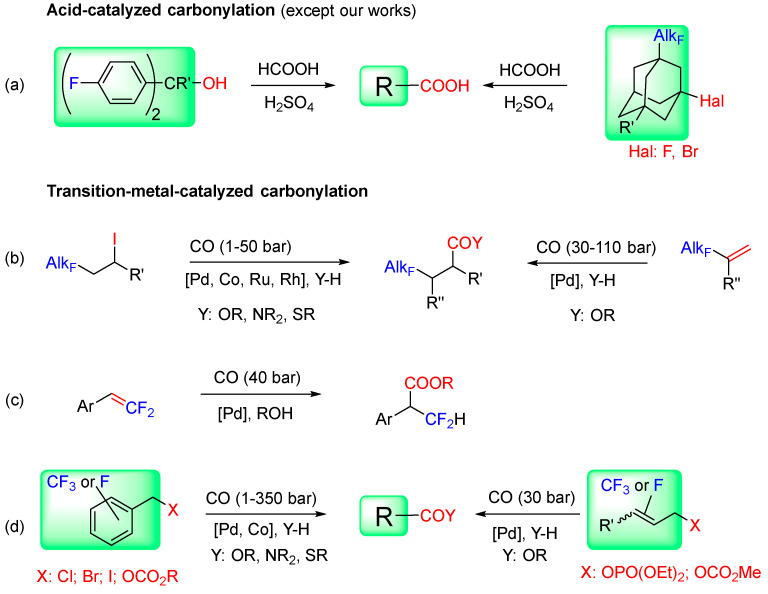
Acid-catalyzed carbonylation reactions of organofluorine compounds (**a**) and similar metal-catalyzed transformations (**b**–**d**).

Organofluorine compounds are objects of interest for basic research in organic chemistry as well as for application-oriented research in materials science, biomedicine, and agriculture [1,2,3,4,5,6,7,8,9,10]. Therefore, the development of new synthetic methods to novel fluorinated building blocks is of great importance for the future of the science. In the chemistry of non-fluorinated hydrocarbons and their derivatives, there are many well-known examples of carbonylation reactions catalyzed by acids (Brønsted or Lewis) that are applicable to alkyl halides, alcohols, and other compounds able to generate carbocations in acidic systems, and these reactions, proceeding via the CO addition to a carbocation, yield carboxylic acid derivatives [11,12,13]. In contrast to a plethora of reactions involving fluorinated cations, the acid-catalyzed carbonylation of polyfluorinated species had not been known before our recent studies [14,15,16,17,18], while a reverse reaction—decarbonylation of fluorinated acyl halides under the action of Lewis acids—is common [19,20,21,22]. The examination of the acid-catalyzed carbonylation of organofluorine compounds—accounting only for those substrates where fluorine atoms influence the reaction center—has showed that the examples are limited to mono- and difluoro-substituted diarylcarbinols [23], and to mono-(polyfluoroalkyl)-substituted adamantyl halides [24,25] (Scheme 1a).

Both transition metal-catalyzed carbonylation at a C(*sp*^3^)–X bond (X = Hal, OR) and the hydrocarboxylation or hydroesterification of alkenes provide an alternative to acid-catalyzed carbonylation, with both of these methods resulting in carboxylic acids or their derivatives. These approaches have more examples for fluorinated compounds (Scheme 1b–d), but the scope of substrates is quite small, and there is only little evidence regarding polyfluorinated species. For instance, aliphatic carboxylic acids with a perfluoroalkyl moiety (that can be quite long) at the *β*-position were synthesized by a Pd-, Ru-, or Co-catalyzed carbonylation of polyfluorinated alkyl iodides (Scheme 1b) [26,27,28,29,30,31,32]; Pd-catalyzed hydrocarboxylation and hydroesterification of perfluoroalkyl-substituted ethylenes using CO [33,34,35,36,37] were also employed to obtain the acids and their derivatives (Scheme 1b). For the synthesis of *α*-CF_2_H-substituted arylacetic acid esters, a Pd-catalyzed hydroesterification of 1,1-difluoro-2-arylethylenes was used [38] (Scheme 1c). Another case in point is a Pd- or Co-catalyzed carbonylation of primary benzyl derivatives ArCH_2_X [39,40,41,42,43,44,45,46,47,48] and esters of allylic alcohols [49,50], but fluorinated examples featured only substrates with one fluorine atom or CF_3_ group at the aryl moiety or at the double bond (Scheme 1d).

**Scheme 2:**
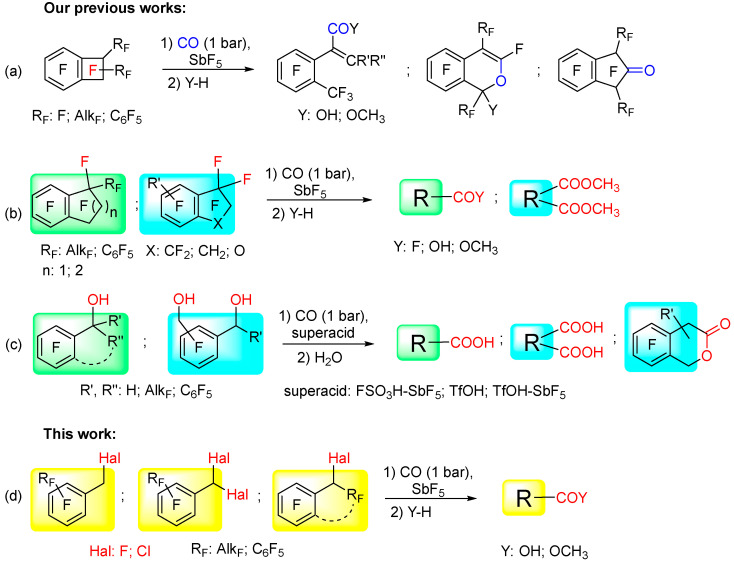
Acid-mediated carbonylation of polyfluorinated benzocyclobutenes (**a**), indans and tetralins (**b**), 1-arylalkan-1-ols (**c**) in our previous works and SbF_5_-mediated carbonylation of polyfluorinated benzyl halides in this work (**d**).

Previously, we demonstrated the use of a carbonylation reaction for the introduction of functional groups to some polyfluorinated and even perfluorinated compounds (Scheme 2). We found that perfluorinated benzocyclobutenes [14,15] and their carbonyl derivatives [17] undergo carbonylation/four-membered ring-opening tandem reactions in the presence of carbon monoxide and SbF_5_ (Scheme 2a). In these reactions, an irreversible four-membered ring transformation promotes the conversion of polyfluorobenzocyclobutenes into the final products. The carbonylation of per- and polyfluorinated indanes and tetralins, leading to fluorocarbonyl derivatives or corresponding acids (if subjected to hydrolysis), was also demonstrated by performing the reaction in the CO–SbF_5_ system [16]. In this case, when the substrate has a five- or six-membered cycloalkyl ring, no skeletal rearrangements occurred and the carbonylation proceeds as an equilibrium process, with the conversion substantially depending on the substrate structure (Scheme 2b). Recently, we have developed a carbonylation procedure for a series of polyfluorinated alkylbenzenes and benzocycloalkenes with primary, secondary, or tertiary OH groups at benzylic positions using CO in the presence of superacids, the method allowed obtaining the corresponding mono- and dicarboxylic acids or lactones [18] (Scheme 2c).

In continuation of our study on the carbonylation of polyfluorinated compounds, we now have focused on various benzyl halides. The current work describes the carbonylation of a series of polyfluorinated benzyl halides Ar_F_CHHalR (R = Hal, H, C_6_F_5_, Alk_F_) in the CO–SbF_5_ system. The developed method allows transformation of the above compounds into -COOH or -COOR derivatives (Scheme 2d). Synthesized by this method, polyfluorinated α-aryl-substituted carboxylic acids are now being considered to be used as building blocks for bioactive compounds, because similar acids and esters with fluorine atoms in both aliphatic and aromatic moieties have been investigated for use in medicine [51,52,53,54,55,56], as it is in the case of halogenated derivatives of benzocycloalkene-1-carboxylic acids [57,58,59,60,61]. Polyfluorinated aromatic moieties in a molecule would allow further synthetic modifications via S_N_Ar substitution. Additionally, the perfluorinated aryl group has unique physicochemical properties that can be used in the design of materials. For instance, derivatives of pentafluorophenyl acetic acid have been utilized to create gas sensors [62], supramolecular associates for biological applications [63,64], and semiconductor and photoluminescent materials [65,66].

## 2. Results and Discussion

### 2.1. Carbonylation of Polyfluorinated Aromatic Compounds with CF_2_H and CCl_2_H Groups

We showed that the CF_2_H and CCl_2_H groups in polyfluorinated benzal fluorides **1a**–**c** and benzal chlorides **2a**,**d** underwent transformation upon reaction with CO in a SbF_5_ medium at room temperature under atmospheric pressure, which resulted in dicarbonylation products (Table 1). Thus, assisted by SbF_5_, compound **1a** added two CO molecules to produce malonate **3′a** following methanol treatment (an aqueous treatment yielded the corresponding diacid **3a**, which was unstable, being prone to decarboxylation [67], and slowly decomposed in an Et_2_O solution to phenylacetic acid). Using compound **1a**, we showed that the highest conversion (96%) to the carbonylation product was achieved with an excess of SbF_5_ (Table 1, entries 1–3). The addition of CO to the alicyclic moiety of indan **1b**, as in the case of a number of other polyfluoroindanes [16], did not occur to a considerable extent.

The behavior of benzal chloride **2a** and benzal fluoride **1a** differed, and the conversion of compound **2a** to product **3′a** in the reaction with an excess of SbF_5_ reached only 35% (Table 1, entries 11, 12). The low conversion was probably due to the greater stability of the corresponding chlorine-substituted benzyl cation **4**, as compared with a fluorine-substituted counterpart. The CF_3_ group in compound **2d** decreases the stability of the benzyl cations **4** and **5** generated from it; as a result, its conversion into the dicarbonylation product under similar conditions was much higher (Table 1, entry 14).

We should note that the CF_3_ group in compound **2d** and CF_3_ groups in the other trifluoromethyl-substituted benzenes used in this work were unreactive towards CO addition; this observation agrees that the reverse reaction—the decarbonylation of Ar_F_ CF_2_COF under the action of SbF_5_—is much more highly favored [22].

Reducing the amount of SbF_5_ resulted in the selective monocarbonylation of compounds **2a**,**d**, yielding chloroacetic acids **6a**,**d**, or ester **6′a** (Table 1, entries 8, 9, 13). The highest content of the monocarbonylation products was achieved using 1 equiv. of SbF_5_ (Table 1, entries 7–10). But in contrast to this, the use of 1 equiv. of SbF_5_ in the case of difluoromethyl derivatives **1a**,**c** (Table 1, entries 1, 5) afforded a mixture of dicarbonylation products, along with the starting compounds and the solvolysis products of the cations from the starting compound (Ar_F_CH(OMe)_2_ and Ar_F_CHO). Monocarbonylation products were not formed in considerable amounts.

### 2.2. Carbonylation of Polyfluorinated Aromatic Compounds with CH_2_F and CH_2_Cl Groups

A number of polyfluorinated primary benzyl fluorides **7** and benzyl chlorides **8** selectively reacted with CO in the SbF_5_ medium to give the corresponding arylacetic acids **9** following hydrolysis of the reaction mixtures (Table 2). Previously, for the carbonylation of pentafluorobenzyl alcohol (C_6_F_5_CH_2_OH) we used the CO–TfOH system at 50 °C, and phenylacetic acid **9a** was the only product obtained with a 90% yield [18]. The carbonation of benzyl fluoride **7a**, as well as benzyl chloride **8a**, in the CO–SbF_5_ system at room temperature resulted in a similar yield of acid **9a** (Table 2, entries 4, 6). The reactions proceeded smoothly with an excess of SbF_5_, ensuring the complete conversion of the starting compound into a benzyl cation; a decrease in the SbF_5_ amount (Table 2, entries 1–3, 5) facilitated side reactions leading to resinous byproduct formation.

*Ortho*- and *meta*-CF_3_ derivatives **8e**,**f** were also shown to be able to be selectively carbonylated into arylacetic acids **9e**,**f** (Table 2, entries 9, 10). Despite the use of an excess of SbF_5_, the formation of corresponding 2- and 3-(carboxymethyl)tetrafluorobenzoic acids **10** and **11**—as a result of the conversion of the CF_3_ group upon hydrolysis—occurred only to a small extent and the yield of such products did not exceed 10%. In contrast to isomers **8e**,**f**, the carbonylation of the *para*-CF_3_ derivative **8d** in the CO–SbF_5_ system was unsuccessful, as was the case with benzyl fluoride **7d** (Table 2, entries 7, 8). The reaction resulted in a complex mixture of compounds, in which acid **9d** was detected only in small amounts as well as 4-(carboxymethyl)tetrafluorobenzoic acid **12**. This result was apparently owing to side transformations of compounds **7d** and **8d** in SbF_5_, which occurred instead of carbonylation, since the formation of a complex mixture of products was also observed in the absence of CO. One of the identified products formed in significant quantities in the reactions of compounds **7d** and **8d** was 4-methyltetrafluorobenzoic acid **13**, which indicates the occurrence of oxidation-reduction processes in the SbF_5_ medium. The replacement of the CF_3_ group in compound **8d** with a perfluoroisopropyl group or with a pentafluorophenyl group did not significantly change the result of the reactions of the corresponding benzyl chlorides, **8g**,**h**, with CO–SbF_5_. The reactions resulted in multicomponent mixtures with a low content of acids **9g**,**h** (Table 2, entries 11, 12).

The unsatisfactory result of the carbonylation of *para*-CF_3_ derivatives **7d** and **8d** aroused an interest to study the possibility of carbonylation of the corresponding alcohol **14d** in superacids using our previously proposed method [18]. We demonstrated that its interaction with CO in an equimolar mixture of TfOH–SbF_5_ at 60 °C led quite selectively to acid **9d** (Table 2, entry 13). Complete conversion to the carbonylation product required rather vigorous conditions, and, as a result, 4-(carboxymethyl)tetrafluorobenzoic acid **12** was also formed as a byproduct. The formation of other byproducts in significant amounts was not observed in this reaction. The isomeric alcohols **14e**,**f** can be selectively carbonylated in a less acidic medium, such as TfOH, without addition of SbF_5_, at 50 or 75 °C, respectively (Table 2, entries 14, 15).

We previously reported the carbonylation of bis(hydroxymethyl)tetra fluoro benzenes in superacids, the *para*- and *meta*-isomers selectively transformed into the corresponding dicarboxylic acids, whereas the *ortho*-isomer added only one molecule of CO, thereby yielding 5,6,7,8-tetrafluoroisochroman-3-one (**15**) [18]. The result of the carbonylation of bis(chloromethyl)tetrafluorobenzenes **16a**–**c** in the CO–SbF_5_ system was different (Table 3). The carbonylation of the *para*-isomer **16a**, as in the case of *para*-substituted benzyl halides **8d**,**g**,**h**, resulted in a mixture of products; dicarboxylic acid ester **17′a** was detected in the reaction mixture obtained after the methanol treatment, but its content did not exceed 15%, while the products of redox transformations containing the Ar_F_-CH_3_ moiety were also observed. The carbonylation of the *meta*-isomer **16b** proceeded more selectively, but resinification did occur, which reduced the yield of ester **17′b**. *Ortho*-isomer **16c** reacted with two CO molecules without resinification, the main product was diacid **17c** or its methyl ester **17′c** depending on subsequent treatment, and the admixture of lactone **15**, a monocarbonylation product, in the reaction mixture did not exceed 10%.

### 2.3. Carbonylation of Polyfluorinated Diphenylmethanes (C_6_F_5_)_2_CHHal

It was shown that benzyl halides **18** and **19** can be converted to acid **20** by carbonylation in the CO–SbF_5_ system with subsequent aqueous treatment of the reaction mixture (Table 4).

The equilibrium of the carbonylation reaction of halogenated diphenylmethanes **18** and **19** in an excess of SbF_5_ was strongly shifted toward bis(pentafluorophenyl)methyl cation **21** due to the high stability of the latter. With the use of 6 equiv. of SbF_5_, the conversion to acid **20** did not exceed 3% (Table 4, entries 4, 7). Reducing the amount of SbF_5_ to 0.5–1 equiv. for fluoro derivative **18** increased its conversion to acid **20** to 45–50% (Table 4, entries 1, 2), but with a lack of SbF_5_ the formation of a solid salt of cation **21** occurred, which impeded the carbonylation of compound **18**. The generation of cation **21** by the SbF_5_-assisted cleavage of the chloride anion from **19** did not result in the formation of a solid salt precipitate (because of different counterion), and probably that is why the conversion of compound **19** to acid **20** in the presence of 0.5 equiv. of SbF_5_ was significantly higher and reached 88% (Table 4, entry 5). The reaction mixture obtained after hydrolysis contained products (C_6_F_5_)_2_CF_2_ and (C_6_F_5_)_2_CO. This finding apparently implies that a chlorine-mediated oxidation process (i.e., the substitution of a hydrogen atom by fluorine) took place, and it was followed by the formation of perfluorodiphenylmethyl cation.

### 2.4. Carbonylation of Polyfluorinated Aromatic Compounds with CHHalAlk_F_ Groups and the Concomitant Elimination of HF

The interaction of benzyl halides **22b**,**d** and **23a**–**d** with CO in a SbF_5_ medium (Table 5) occurred more readily as compared to diphenylmethanes **18** and **19** (Table 4). A perfluoroalkyl group at the reaction center reduces the stability of the intermediate benzyl carbocation, thus increasing its reactivity; as a result, the carbonylation of chloro-derivatives **23a**–**c** and fluoro-derivatives **22b**,**d** occurred with complete conversion both in an excess of SbF_5_ and with one equiv. of SbF_5_. Using **23a** as a model compound, we demonstrated that further reduction in the SbF_5_ amount to 0.5 equiv. resulted in incomplete conversion of the starting compound (Table 5, entry 1).

**Scheme 3:**
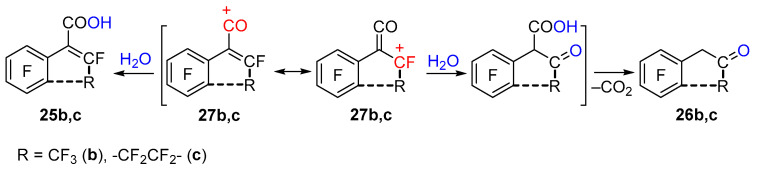
Formation of compounds **25b**,**c** and **26b**,**c** in the reaction of cations **27b**,**c** with water.

Since benzyl halides **22** and **23** have a perfluoroalkyl group at the *α*-carbon atom, their carbonylation can be accompanied by elimination of HF, thus giving rise to *α*,*β*-unsaturated carboxylic acids and esters **25** and **25′** along with, or instead of, saturated products **24** and **24′** (Table 5). The reaction of compound **23a** at room temperature selectively gave products **24a** or **24′a**, without appreciable contribution from the HF elimination process. Whereas after the carbonylation of compounds **22b** and **23b**,**c** in an excess of SbF_5_ (4–6 equiv.) at room temperature, along with acids **24b**,**c**, small amounts of *α*,*β*-unsaturated acids **25b**,**c** were obtained (Table 5, entries 7, 8, 11, 16); and if 1–2 equiv. of SbF_5_ was used, the formation of ketones **26b**,**c** was also observed (Table 5, entries 5, 6, 10, 15). In these reactions prior to a workup, the carbonylation with the concomitant elimination of HF would result in cations **27b**,**c**. During an aqueous workup, the formation of acids **25b**,**c** from cations **27b**,**c** is obvious. Ketones **26b**,**c** were probably formed from the same cations **27b**,**c** by the nucleophilic attack of a water molecule at the positively charged CF-position followed by the decarboxylation of the resulting ketoacid (Scheme 3). Therefore, compound **26** should be accounted for as an elimination product. In this regard, we can conclude that reducing the amount of SbF_5_ does not reduce the contribution of the HF elimination process that accompanies carbonylation.

A fivefold increase in the reaction time for compound **23b** increased the content of the elimination product **25b** by less than twofold (Table 5, entries 11, 12), whereas an increase in temperature to 70 °C significantly shifted the product ratio in favor of elimination products decreasing the content of **24b**,**c** up to their complete conversion (Table 5, entries 9, 13, 14, 17). The concomitant elimination of HF with the formation of *α*,*β*-unsaturated acids (including compounds **25b**,**c**) was observed earlier [18] in the carbonylation of polyfluorinated secondary 1-arylalkan-1-ols in super acids, but complete conversion could not be achieved by the method [18].

**Scheme 4:**
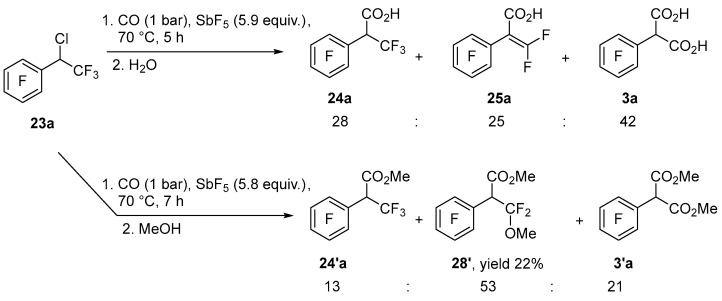
Carbonylation of compound **23a** in the CO–SbF_5_ system at 70 °C.

The aforementioned HF elimination reactions occurred more easily for compound **23c** with a six-membered aliphatic cycle compared to its acyclic analog **23b** (chloro- and fluoro-derivatives **23b** and **22b** showed similar reactivity, Table 5). For compound **23a** with a shorter aliphatic moiety, this process was even more unlikely and was observed only at elevated temperatures. In the case of compound **23a**, it was not possible to isolate unsaturated acid **25a** or its ester **25′a** from the reaction mixture obtained by its carbonylation in a CO–SbF_5_ system at 70 °C (Scheme 4) due to the significantly greater reactivity of these compounds containing two geminal fluorine atoms at the double bond toward nucleophilic addition reactions. Hydrolysis of the reaction mixture followed by extraction with Et_2_O yielded a mixture of three major products **3a**, **24a**, and **25a**, with the former predominating (Scheme 4). Compound **3a** apparently resulted from the addition of water to the double bond of acid **25a** during aqueous treatment. However, even after drying the Et_2_O extract over MgSO_4_, the content of acid **25a** in the solution rapidly decreased, probably due to its reaction with traces of water. When the reaction mixture was treated with methanol, the unsaturated ester **25′a** was not detected at all. Instead, its adduct with methanol **28′** was present, which was the main reaction product, with the minor being compounds **3′a** and **24′a**.

**Scheme 5:**
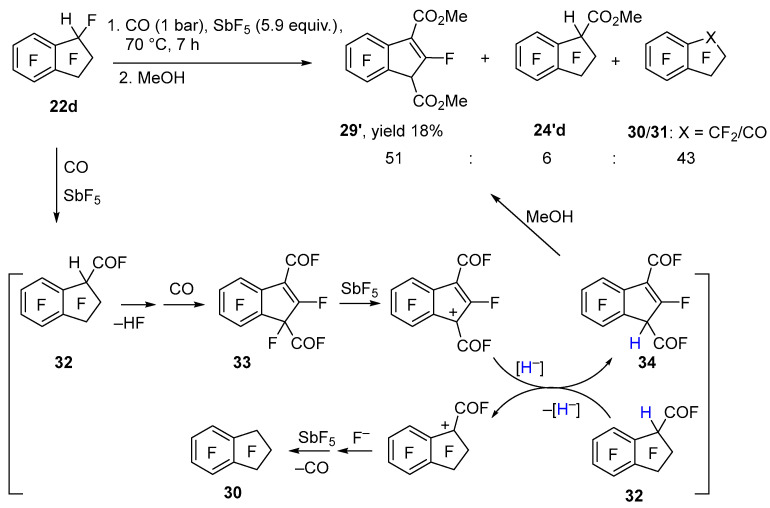
Carbonylation of compound **23a** in the CO–SbF_5_ system at 70 °C.

In contrast to tetralin **23c**, the carbonylation at room temperature of indan **22d** with a five-membered alicyclic ring yielded only traces of the HF elimination product **25d**. In the presence of 1 equiv. SbF_5_, the compound **22d** was smoothly converted into corresponding acid **24d** (Table 5, entry 18). Moreover, the carbonylation reactions of indan **22d** and tetralin **23c** at 70 °C gave completely different results. The carbonylation of indan **22d** in an excess of SbF_5_ at 70 °C, followed by treatment with methanol, gave dicarboxylic ester **29′** instead of ester **25′d** (Scheme 5). In addition, the reaction mixture also contained perfluorinated indan **30** and indanone **31**. To explain the formation of products **29′** and **30** in these conditions, the following sequence of transformations can be proposed. The carbonylation of compound **22d** yields carbonyl fluoride **32**. Subsequent elimination of HF gives the corresponding indene, which then adds a second CO molecule affording compound **33**. The indenyl cation generated from compound **33** is probably able to abstract a hydride ion from the initially formed carbonyl fluoride **32**, thereby giving indene **34** that yielded product **29′** after a methanol workup. The oxidation product **35** of carbonyl fluoride **32** was then converted to perfluoroindan **30** via the addition of fluoride ion and decarbonylation.

Carbonylation of chloroindan **23d** occurred with significantly lower selectivity (Table 5, entries 19, 20) if compared with the reaction of indan **22d** or chlorotetralin **23c** (Table 5, entries 15, 16, 18). Even though acid **24d** was the main reaction product, it was formed alongside many byproducts, containing benzylic CFCl or CO moieties. We presume that, in this case, side reactions of the starting compound and/or of the carbonylation product took place, involving the substitution of a fluorine atom at the benzylic CF_2_ moiety by chlorine. The resultant products are able to generate indanyl cations with a chlorine atom at the cationic center, and these cations would be more stable than those with a fluorine atom [68]. That is why after the water treatment of the reaction mixture, significant amounts of indanones (the hydrolysis products of chloroindanyl cations) and indanes with benzylic CFCl moiety were produced.

**Scheme 6:**
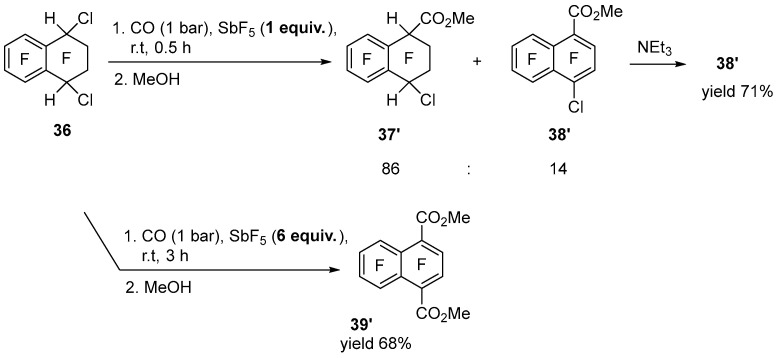
Carbonylation of compound **36** in the CO–SbF_5_ system.

In the case of tetralin **36** containing two benzylic CHCl moieties, mono- or diarbonylation is possible, the degree of carbonylation was shown to depend on the amount of antimony pentafluoride. The main product of the reaction with 1 equiv. SbF_5_ followed by a methanol treatment was monomethoxycarbonyl derivative **37′** (Scheme 6). The reaction mixture also contained naphthalenecarboxylic ester **38′**, which was the product of HF elimination. Complete conversion of compound **37′** to naphthalene derivative **38′** was achieved by exposing the reaction mixture to NEt_3_. In the presence of 6 equiv. SbF_5_, compound **36** added two molecules of CO, and, at room temperature, the concomitant elimination of two HF molecules occurred completely, thereby yielding naphthalenedicarboxylic ester **39′** after treatment of the reaction mixture with methanol. The methanol workup required long time to convert the obtained carbonyl fluorides into esters **38′** and **39′**, arguably because the fluorocarbonyl group at the α-carbon atom of polyfluoronaphthalenes is sterically hindered.

### 2.5. Starting Compounds

Chlorides **8a**,**d**–**h**, **19,** and **23a** were obtained by the reaction of alcohols **14a**,**d**–**h**, **40**, and **41a** with SOCl_2_ at 75 °C or with PCl_5_ at 100 °C (Table 6). Benzyl alcohols **14d**–**h** were derived from carboxylic acids **42d**–**h**, which were first converted into acyl chlorides via a reaction with SOCl_2_ and then the acyl chlorides were reduced with LiBH_4_. Fluorine derivatives **7a**,**d,** and **18** were synthesized by heating of the corresponding chlorides **8a**,**d**, and **19** with CsF at 250–260 °C without a solvent.

**Scheme 7:**
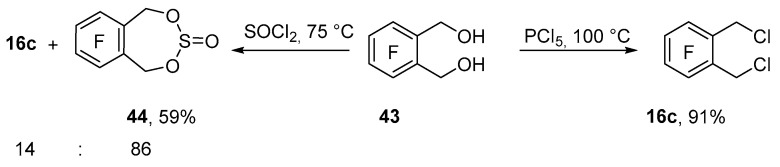
Reactions of diol **43** with SOCl_2_ and PCl_5_.

In contrast to alcohols **14a**,**d**–**g**, the reaction of diol **43** with SOCl_2_ under similar conditions gave mainly cyclic sulfite **44**, and the target dichloride **16c** was obtained in good yield by heating diol **43** with PCl_5_ (Scheme 7).

**Scheme 8:**
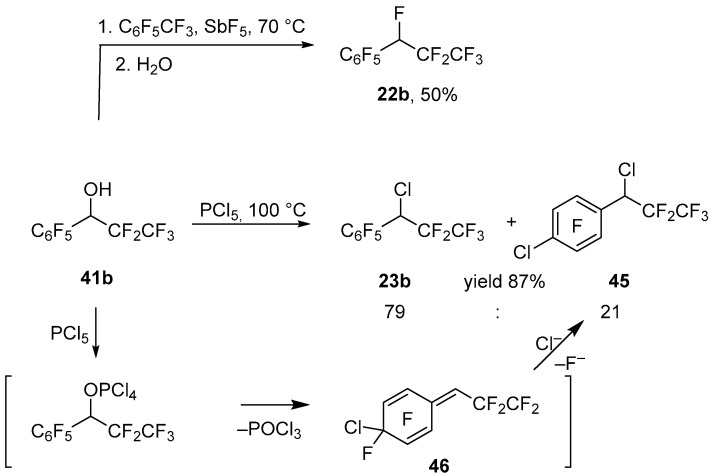
Reactions of alcohol **41b** with PCl_5_ and the synthesis of compound **22b**.

In contrast to alcohol **41a**, the reaction of its homolog **41b** with PCl_5_ not only yielded the target chloride **23b** but also resulted in considerable amounts of the *para*-chloroderivative **45** (Scheme 8). The greater steric hindrance of the reaction center probably accounts for the side product, which apparently formed via a PCl_5_-assisted S_N_1 substitution of the OH group accompanied by the attack of a chloride ion at the sterically more accessible position, yielding propylidenecyclohexadiene **46**. The subsequent elimination of one of the halogenide ions from the CFCl moiety and addition of a chloride ion to the benzylic position restores the aromaticity of the arene and yields a mixture of mono- and dichloroderivatives **23b** and **45**. Fluorine derivative **22b** was obtained from alcohol **41b** by the reaction with C_6_F_5_CF_3_ and SbF_5_ (Scheme 8). In this case, perfluorotoluene simultaneously acted as a donor of a fluoride ion to replace the OH group and as an acceptor of the oxygen, degrading into perfluorobenzoic acid.

**Scheme 9:**
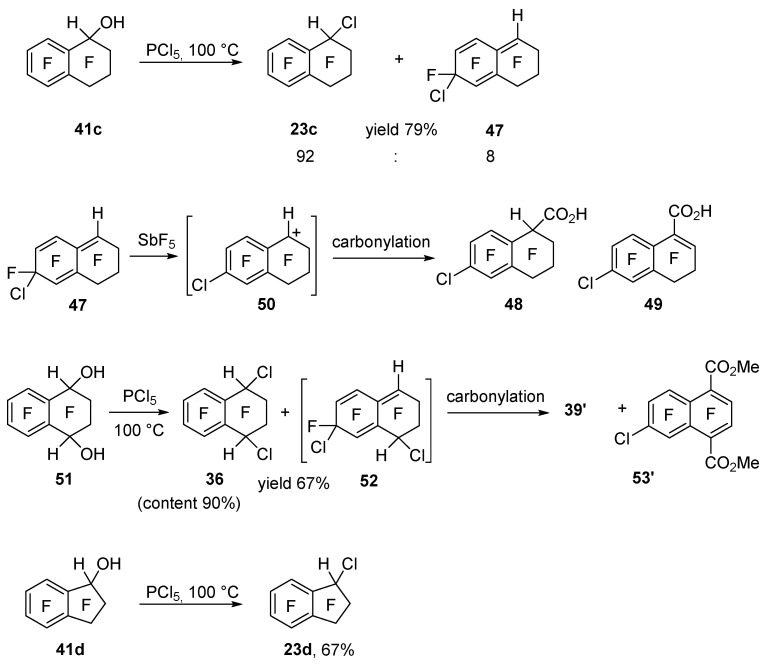
Synthesis of compounds **23c**,**d** and **36**; formation of chlorine-containing admixtures in carbonylation of the obtained products.

When replacing the OH group of tetralinol **41c** in the reaction with PCl_5_ at 100 °C, the addition of chloride ion to the aromatic ring also occurred, but to a lesser extent compared to alcohol **41b**, while subsequent rearomatization almost did not occur. As a result, non-aromatic triene isomer **47** was formed as minor product along with target **23c** (Scheme 9). The interaction of this obtained mixture of compounds **23c** and **47** with CO–SbF_5_ gave carbonylation products **24c** and **25c** along with admixture (6–8%) of their monochloro-derivatives **48** and **49** with a chlorine atom in the aromatic moiety. The content of the latter was close to the content of compound **47** in the starting compound. This indicates that under the action of antimony pentafluoride on compound **47**, the fluoride ion is predominantly abstracted, rather than the chloride ion, with the formation of a chlorine-substituted cation **50**, which then reacts with CO. Dichlorotetralin **36**, obtained by the interaction of diol **51** with PCl_5_, apparently also contained an admixture of non-aromatic isomer **52**, since its carbonylation gave diester **39′** with 8% of the chloro-derivative **53′** as an impurity.

In contrast to compounds **41b**,**c**, the reaction of indanol **41d** with PCl_5_ at 100 °C gave no products with a chlorine atom in the aromatic moiety; however, along with the target chloroindan **23d**, a considerable amount of non-volatile by-products quite similar to the starting alcohol was obtained, which were apparently its esters with phosphoric acid, and that explains the rather modest yield of the target compound (Scheme 9).

The other starting compounds were prepared according to the procedures outlined in the literature: CF_2_H derivatives **1a**–**c [69]**, CCl_2_H derivatives **2a**,**d [70]**, dichlorides **16a [71]** and **16b [72]**, indane **22d [73]**, alcohols **14a**, **40 [74]**, **41a**–**d**, **51 [75]**, and diol **43c [18]**.

### 2.6. Structural Analysis of Compounds

The structures of new compounds were established by means of high-resolution mass spectrometry (HRMS), elemental analysis, and spectral characteristics (copies of the ^1^H and ^19^F NMR spectra are available in Supplementary Materials). Signals in NMR spectra of compounds were assigned on the basis of chemical shifts of the signals, their fine structure, and integral intensities. The obtained compounds **20**, **24a**–**d**, **24′a**,**d**, **25′b**, **26c**, **39′** [18], **9a [76]**, **10 [67]**, **30 [69]**, **31 [77]** were identified by the comparison of ^19^F NMR findings with the data in the literature.

To confirm the structure of some products obtained in the reactions, we also synthesized compounds **11**–**13** in separate experiments and recorded their NMR spectra. Notably, 3- and 4-(Carboxymethyl)tetrafluorobenzoic acids **11** and **12** were obtained by the reaction of compounds **9e** and **9d** with CF_3_CO_2_H and SbF_5_. This approach for synthesis of carbonyl derivatives was developed earlier, in [78]. Methylbenzoic acid **13** was obtained by hydrolysis of a solution of 4-methylheptafluorotoluene in SbF_5_, according to the method in [79].

## 3. Materials and Methods

### 3.1. General Information

Analytical measurements and spectral analyses were carried out at the Multi-Access Chemical Research Center SB RAS. IR spectra were acquired on a Bruker Vector 22 IR (Bruker Corporation, Billerica, MA, USA) spectrophotometer. ^19^F, ^1^H NMR spectra were recorded on a Bruker AV 300 (Bruker Corporation, USA) instrument (282.4 MHz, and 300 MHz, respectively). Chemical shifts are given in *δ* ppm from CCl_3_F (^19^F) and TMS (^1^H). Hexafluorobenzene C_6_F_6_ (^19^F, *δ_F_* −162.9 ppm), residual CHCl_3_ (^1^H, *δ_H_* 7.24 ppm), and acetone-*d*_5_ (^1^H, *δ_H_* 2.04 ppm) served as internal standards. Spin–spin coupling constant values are given in Hz. Gas chromatography coupled with mass spectrometry (GC-MS) was performed on a Hewlett Packard G1081A (Agilent Technologies, Santa Clara, CA, USA) instrument equipped with a gas chromatograph Hewlett Packard 5890 and a mass selective detector HP 5971 (EI 70 eV). Molecular masses of the compounds were determined by HRMS with a Thermo Electron Corporation DFS (Thermo Electron, Karlsruhe, Germany) instrument (EI 70 eV). Elemental analysis was performed on an Eurovector EA 3000 (Eurovector, S.p.A., Pavia, Italy) automatic CHNS analyzer. Melting points were determined on an Electrothermal IA 9100 (Electrothermal, Chelmsford, UK) apparatus (1 °C/min). Contents of products in reaction mixtures were determined using ^19^F NMR spectroscopic data. Column chromatography was carried out on silica gel 60 (Merck, Darmstadt, Germany), particle size 0.063–0.200 mm).

Antimony pentafluoride was distilled under atmospheric pressure (bp 142–143 °C). TfOH was purchased from commercial sources (99% purity). Carbon monoxide was prepared by the decomposition of formic acid in concentrated sulfuric acid, and was additionally dried by passing it through a layer of concentrated sulfuric acid. All reactions were carried out in glassware.

### 3.2. Typical Carbonylation Procedures

*a*. A mixture of a substrate and SbF_5_ (a substrate was added dropwise to SbF_5_ under stirring and cooling the flask in an ice bath) was intensively stirred in a round-bottom glass flask (5–10 mL) in a slow flow of CO under atmospheric pressure. After the time (mentioned below for each experiment), the mixture was poured into 5% hydrochloric acid (10 mL per 1 g of SbF_5_) and extracted three times with 3–5 mL of Et_2_O (if not specified otherwise). The extract was dried over MgSO_4_ and analyzed by ^19^F NMR spectroscopy.

*b*. A mixture of a substrate and SbF_5_ (a substrate was added dropwise to SbF_5_ under stirring and cooling of the flask in an ice bath) was intensively stirred in a round-bottom glass flask (5–10 mL) in a slow flow of CO under atmospheric pressure. The mixture was poured into methanol (10 mL per 1 g of SbF_5_) cooled to −10–0 °C. The resulting solution was allowed to warm up until it reached room temperature, and then it was poured into 5% hydrochloric acid (7 mL per 1 mL of methanol) and extracted three times with 10–15 mL of CH_2_Cl_2_. The extract was dried over MgSO_4_ and analyzed by ^19^F NMR spectroscopy.

The reaction scale was 0.6–2.4 mmol of a substrate. Reagent amounts, reaction conditions, and procedures for separation of mixtures and product isolation are detailed in the experiments below. In some experiments, C_6_F_6_ or (C_4_F_9_)O were added to the reaction mixture to reduce the viscosity of the mixture and aid mixing during dissolution of a starting alkyl halide in SbF_5_ and/or during the carbonylation process. It is not advisable to use hexafluorobenzene C_6_F_6_ for chlorine-containing substrates because it readily undergoes chlorofluorination in the SbF_5_ medium in the presence of nascent chloride ions.

### 3.3. Carbonylation Experiments

#### 3.3.1. Carbonylation of (Difluoromethyl)pentafluorobenzene (**1a**)

*a*. Compound **1a** (0.324 g, 1.49 mmol) and SbF_5_ (0.320 g, 1.48 mmol) (molar ratio 1:1), according to the typical procedure (Section 3.2a) (2.5 h, r.t.), gave a mixture of compounds **1a**, **3a**, **9a**, and C_6_F_5_CHO in an 12:64:6:18 molar ratio. The content of a compound with CHF moiety (^19^F NMR: *δ* −182.2 (dm, *J*_H,F_ = 45.5) in the reaction mixture did not exceed 1%.

*b*. Compound **1a** (0.190 g, 0.87 mmol), SbF_5_ (0.387 g, 1.79 mmol) (molar ratio 1:2), and C_6_F_6_ (0.2 mL) according to the typical procedure (Section 3.2b) (2.5 h, r.t., 20 min in methanol), gave a mixture of compound **3′a** and C_6_F_5_CH(OMe)_2_ in a 92:8 molar ratio. 

*c*. Compound **1a** (0.504 g, 2.31 mmol) and SbF_5_ (2.646 g, 12.22 mmol) (molar ratio 1:5.3), according to the typical procedure (Section 3.2b) (6 h, r.t., 20 min in methanol), gave a mixture of compound **3′a** and C_6_F_5_CH(OMe)_2_ in a 96:4 molar ratio. Silica gel column chromatography (CHCl_3_ as the eluent) gave 0.622 g of ester **3′a** (yield 90%)

*Dimethyl 2-(perfluorophenyl)malonate (**3′a**).* Colorless liquid. IR (film) ν, cm^−1^: 2960 (CH), 1751 (C=O), 1525, 1508 [FAR (fluorinated aromatic ring)]. ^1^H NMR (CDCl_3_): *δ* 4.94 (s, 1H, CH), 3.79 (s, 6H, CH_3_). ^19^F NMR (CDCl_3_): *δ* −141.1 (m, 2F, F-*ortho*), −154.1 (tt, 1F, *J_para,meta_* = 21, *J_para,ortho_* = 2, F-*para*), −162.5 (m, 2F, F-*meta*). HRMS, *m*/*z*: calcd. for C_11_H_7_O_4_F_5_ 298.0259; found 298.0260.

#### 3.3.2. Carbonylation of 5-(Difluoromethyl)nonafluoroindan (**1b**)

Compound **1b** (0.532 g, 1.61 mmol) and SbF_5_ (2.076 g, 9.59 mmol) (molar ratio 1:5.9), according to the typical procedure (Section 3.2b) (3 h 15 min, r.t., 10 min in methanol), gave a mixture of compounds containing ~85% of ester **3′b**. Silica gel column chromatography (with CH_2_Cl_2_ as the eluent) gave 0.503 g of ester **3′b** (yield 76%).

*Dimethyl 2-(perfluoroindan-5-yl)malonate (**3′b**).* Colorless liquid. IR (film) ν, cm^−1^: 2962 (CH), 1757 (C=O), 1504 (FAR). ^1^H NMR (CDCl_3_): *δ* 5.06 (s, 1H, CH), 3.82 (s, 6H, CH_3_). ^19^F NMR (CDCl_3_): *δ* −107.7 (m, 2F, CF_2_-1 or 3), −108.4 (m, 2F, CF_2_-1 or 3), −117.0 (dtd, 1F, F-4), −121.9 (dd, 1F, F-6), −130.7 (quintet, 2F, CF_2_-2), −141.4 (ddt, 1F, F-7); *J*_1,2_ = *J*_2,3_ = 4, *J*_1,7_ = 7, *J*_3,4_ = 7.5, *J*_4,6_ = 5, *J*_4,7_ = 20, *J*_5,6_ = 19, *J*_6,7_ = 20.5. HRMS, *m*/*z*: calcd. for C_14_H_7_O_4_F_9_ 410.0195; found 410.0197.

#### 3.3.3. Carbonylation of 6-(Difluoromethyl)undecafluorotertalin (**1c**)

*a*. Compound **1c** (0.575 g, 1.51 mmol) and SbF_5_ (1.978 g, 9.14 mmol) (molar ratio 1:6), according to the typical procedure (Section 3.2b) (2 h 15 min, r.t., 10 min in methanol), gave a mixture of compounds containing ~90% of ester **3′c**. Silica gel column chromatography (CH_2_Cl_2_ as the eluent) gave 0.562 g of ester **3′c** (yield 81%).

*b*. Compound **1c** (0.467 g, 1.23 mmol) and SbF_5_ (0.267 g, 1.23 mmol) (molar ratio 1:1), according to the typical procedure (Section 3.2b) (45 min, r.t., 10 min in methanol), gave a mixture of compounds containing 55% of starting compound **1c** and 30% of ester **3′c**. Content of a compound with CHF moiety (^19^F NMR: *δ* −186.9 (dm, *J*_H,F_ = 45.5)) did not exceed 5%.

*Dimethyl 2-(perfluorotertalin-6-yl)malonate (**3′c**).* Colorless liquid. IR (film) ν, cm^−1^: 2962 (CH), 1753 (C=O), 1495, 1482 (FAR). ^1^H NMR (CDCl_3_): *δ* 5.07 (s, 1H, CH), 3.82 (s, 6H, CH_3_). ^19^F NMR (CDCl_3_): *δ* −106.5 (m, 2F, CF_2_-1 or 4), −107.2 (m, 2F, CF_2_-1 or 4), −113.6 (tdd, 1F, F-4), −122.7 (dd, 1F, F-6), −135.4 (m, 2F, CF_2_-2 or 3), −135.6 (m, 2F, CF_2_-2 or 3), −138.5 (qd, 1F, F-7); *J*_4,5_ = 22, *J*_5,7_ = 6, *J*_5,8_ = 15, *J*_7,8_ = *J*_1,8_ = 20.5. HRMS, *m*/*z*: calcd. for C_15_H_7_O_4_F_11_ 460.0163; found 460.0167.

#### 3.3.4. Carbonylation of (Dichloromethyl)pentafluorobenzene (**2a**)

*a*. Compound **2a** (0.467 g, 1.86 mmol) and SbF_5_ (0.202 g, 0.93 mmol) (molar ratio 1:0.5) according to the typical procedure (Section 3.2a) (3 h, r.t.), gave a mixture of compounds **1a**, **2a**, **6a**, and C_6_F_5_CHO in a 5:44:41:10 molar ratio.

*b*. Compound **2a** (0.524 g, 2.09 mmol), SbF_5_ (0.458 g, 2.12 mmol) (molar ratio 1:1), and C_6_F_6_ (0.3 mL, was added 2.5 h after the start of carbonylation) according to the typical procedure (Section 3.2a) (3 h, r.t.), gave a mixture of compounds **1a**, **2a**, **6a**, and C_6_F_5_CHO in an 1:3:89:7 molar ratio. Evaporation of the solvent and sublimation (110 °C, 3 Torr) afforded 0.373 g (yield 69%) of acid **6a**.

*c*. Compound **2a** (0.390 g, 1.55 mmol), SbF_5_ (0.355 g, 1.55 mmol) (molar ratio 1:1), and C_6_F_6_ (0.3 mL, was added 2 h after the start of carbonylation) according to the typical procedure (Section 3.2b) (3 h, r.t., 15 min in methanol), gave a mixture of compounds **1a**, **2a**, **6′a**, C_6_F_5_CHO, and C_6_F_5_CH(OMe)_2_ in an 1:3:86:3:7 molar ratio. Silica gel column chromatography (CCl_4_–CH_2_Cl_2_ [1:1, *v*/*v*] as the eluent) gave 0.199 g of ester **6′a** (yield 47%), such modest yield is due to the difficulty of separating from other products.

*d*. Compound **2a** (0.498 g, 1.98 mmol), SbF_5_ (0.860 g, 3.97 mmol) (molar ratio 1:2), and C_6_F_6_ (1 mL, was added 1 h after the start of carbonylation) according to the typical procedure (Section 3.2b) (3 h, r.t., 15 min in methanol), gave a mixture of compounds **1a**, **6′a**, C_6_F_5_CHO, and C_6_F_5_CH(OMe)_2_ in an 12:76:2:10 molar ratio.

*e*. Compound **2a** (0.323 g, 1.29 mmol) and SbF_5_ (1.134 g, 5.24 mmol) (molar ratio 1:4) according to the typical procedure (Section 3.2b) (3 h, r.t., 15 min in methanol), gave a mixture of compounds **1a**, **2a**, **6′a**, C_6_F_5_CHO, and C_6_F_5_CH(OMe)_2_ in a 9:4:35:2:50 molar ratio.

*f*. Compound **2a** (0.305 g, 1.22 mmol) and SbF_5_ (1.578 g, 7.29 mmol) (molar ratio 1:6), according to the typical procedure (Section 3.2b) (4.5 h, r.t., 20 min in methanol), gave a mixture of compounds **1a**, **2a**, **3′a**, C_6_F_5_CHO, and C_6_F_5_CH(OMe)_2_ in a 2:4:25:9:60 molar ratio. 

*2-Chloro-2-(perfluorophenyl)acetic acid (**6a**).* White solid; mp 102–103.5 °C (CCl_4_). IR (KBr) ν, cm^−1^: 1754 (C=O), 1525, 1512 (FAR). ^1^H NMR (CDCl_3_): *δ* 11.37 (s, 1H, COOH), 5.78 (s, 1H, CHCl). ^19^F NMR (CDCl_3_): *δ* −141.4 (m, 2F, F-*ortho*), −151.5 (tt, 1F, *J_para_*_,*meta*_ = 21, *J_para_*_,*ortho*_ = 3, F-*para*), −161.7 (m, 2F, F-*meta*). HRMS, *m*/*z*: calcd. for C_8_H_2_O_2_Cl_1_F_5_ 259.9658; found 259.9655. Anal. calcd. for C_8_H_2_O_2_Cl_1_F_5_: C, 36.88; H, 0.77; Cl, 13.61; F, 36.46%. Found: C, 36.77; H, 0.93; Cl, 13.31; F, 36.38%.

*Methyl 2-chloro-2-(perfluorophenyl)acetate (**6′a**).* Colorless liquid. IR (film) ν, cm^−1^: 2962 (CH), 1770, 1747 (C=O), 1525, 1510 (FAR). ^1^H NMR (CDCl_3_): *δ* 5.70 (s, 1H, CHCl), 3.82 (s, 3H, CH_3_). ^19^F NMR (CDCl_3_): *δ* −141.8 (m, 2F, F-*ortho*), −152.4 (tt, 1F, *J_para_*_,*meta*_ = 21, *J_para_*_,*ortho*_ = 2.5, F-*para*), −161.2 (m, 2F, F-*meta*). HRMS, *m*/*z*: calcd. for C_9_H_4_O_2_Cl_1_F_5_ 273.9815; found 273.9818.

#### 3.3.5. Carbonylation of 1-(Dichloromethyl)-2,3,5,6-tetrafluoro-4-(trifluoromethyl)benzene (**2d**)

*a*. Compound **2d** (0.377 g, 1.25 mmol) and SbF_5_ (0.274 g, 1.27 mmol) (molar ratio 1:1), according to the typical procedure (Section 3.2a) (1 h 45 min, r.t.), gave a mixture of compounds containing ~70% of acid **6d**. After evaporation of Et_2_O, the residue was dissolved in CH_2_Cl_2_ (10 mL) and washed with a saturated aqueous solution of NaHCO_3_ (30 mL). The aqueous layer was separated, acidified with HCl (pH < 1), and extracted with Et_2_O (3 × 5 mL). Evaporation of the solvent and sublimation (120 °C, 1 Torr) gave 0.254 g of acid **6d** (yield 65%).

*b*. Compound **2d** (0.421 g, 1.40 mmol) and SbF_5_ (1.758 g, 8.12 mmol) (molar ratio 1:5.8), according to the typical procedure (Section 3.2b) (3 h 15 min, r.t., 10 min in methanol), gave a mixture of compounds containing ~80% of ester **3′d**. Silica gel column chromatography (CH_2_Cl_2_ as the eluent) gave 0.313 g of ester **3′d** (yield 64%).

*2-Chloro-2-(2,3,5,6-tetrafluoro-4-(trifluoromethyl)phenyl)acetic acid (**6d**).* White solid; mp 68–69 °C (after sublimation). IR (KBr) ν, cm^−1^: 1743 (C=O), 1504 (FAR). ^1^H NMR (CDCl_3_): *δ* 8.60 (br.s, 1H, COOH), 5.80 (s, 1H, CHCl). ^19^F NMR (CDCl_3_): *δ* −57.7 (t, 3F, *J*_CF3,F(3)_ = *J*_CF3,F(5)_ = 22, CF_3_), −139.7 (m, 4F, F-2,3,5,6). HRMS, *m*/*z*: calcd. for C_9_H_2_O_2_Cl_1_F_7_ 309.9626; found 309.9621. Anal. calcd. for C_9_H_2_O_2_Cl_1_F_7_: C, 34.81; H, 0.65; Cl, 11.42; F, 42.82%. Found: C, 35.08; H, 1.08; Cl, 11.14; F, 42.82%.

*Dimethyl 2-(2,3,5,6-tetrafluoro-4-(trifluoromethyl)phenyl)malonate (**3′d**).* Colorless liquid. IR (film) ν, cm^−1^: 2962 (CH), 1755 (C=O), 1500 (FAR). ^1^H NMR (CDCl_3_): *δ* 5.02 (s, 1H, CH), 3.81 (s, 6H, CH_3_). ^19^F NMR (CDCl_3_): *δ* −57.5 (t, 3F, *J*_CF3,F(3)_ = *J*_CF3,F(5)_ = 21.5, CF_3_), −139.3 (m, 2F, F-2,6), −141.0 (m, 2F, F-3,5). HRMS, *m*/*z*: calcd. for C_11_H_7_O_4_F_5_ 348.0227; found 348.0222.

#### 3.3.6. Carbonylation of (Fluoromethyl)pentafluorobenzene (**7a**)

*a*. Compound **7a** (0.398 g, 1.99 mmol), SbF_5_ (0.218 g, 1.01 mmol) (molar ratio 1:0.5), and C_6_F_6_ (0.2 mL), according to the typical procedure (Section 3.2a) (2 h, r.t.), gave a mixture of compounds containing ~15% of acid **9a** along with resignification products.

*b*. Compound **7a** (0.300 g, 1.50 mmol), SbF_5_ (0.640 g, 2.96 mmol) (molar ratio 1:2), and C_6_F_6_ (0.2 mL), according to the typical procedure (Section 3.2a) (45 min, r.t.), gave a mixture of compounds containing ~10% of acid **9a**.

*c*. Compound **7a** (0.330 g, 1.65 mmol), SbF_5_ (1.369 g, 6.32 mmol) (molar ratio 1:3.8), and C_6_F_6_ (0.3 mL), according to the typical procedure (Section 3.2a) (45 min, r.t.), gave a mixture of compounds containing ~75% of acid **9a**.

*d*. Compound **7a** (0.504 g, 2.52 mmol), SbF_5_ (3.261 g, 15.06 mmol) (molar ratio 1:6), and C_6_F_6_ (0.4 mL), according to the typical procedure (Section 3.2a) (45 min, r.t.), gave a mixture of compounds containing ~90–95% of acid **9a**. After evaporation of Et_2_O, the residue was dissolved in CH_2_Cl_2_ (10 mL) and washed with a saturated aqueous solution of NaHCO_3_ (30 mL). The aqueous layer was separated, acidified with HCl (pH < 1), and extracted with Et_2_O (3 × 5 mL). Evaporation of the solvent and sublimation (100 °C, 1 Torr) gave 0.491 g of acid **9a** (yield 86%).

#### 3.3.7. Carbonylation of (Chloromethyl)pentafluorobenzene (**8a**)

*a*. Compound **8a** (0.285 g, 1.32 mmol) and SbF_5_ (0.148 g, 0.68 mmol) (molar ratio 1:0.5), according to the typical procedure (Section 3.2a) (2 h, r.t.), gave a mixture of compounds containing ~10% of acid **9a** along with the starting compound (~10%) and resinification products.

*b*. Compound **8a** (0.521 g, 2.41 mmol), SbF_5_ (3.145 g, 14.53 mmol) (molar ratio 1:6), and (C_4_F_9_)_2_O (0.3 mL), according to the typical procedure (Section 3.2a) (45 min, r.t.), gave a mixture of compounds containing ~90–95% of acid **9a**. After evaporation of Et_2_O, the residue was dissolved in CH_2_Cl_2_ (10 mL) and washed with a saturated aqueous solution of NaHCO_3_ (30 mL). The aqueous layer was separated, acidified with HCl (pH < 1), and extracted with Et_2_O (3 × 5 mL). Evaporation of the solvent and sublimation (100 °C, 1 Torr) gave 0.474 g of acid **9a** (yield 87%).

#### 3.3.8. Carbonylation of 1-(Fluoromethyl)-2,3,5,6-tetrafluoro-4-(trifluoromethyl)benzene (**7d**)

Compound **7d** (0.491 g, 1.96 mmol), SbF_5_ (2.594 g, 11.98 mmol) (molar ratio 1:6.1), and (C_4_F_9_)_2_O (0.2 mL), according to the typical procedure (Section 3.2a) (1 h, r.t.), gave a complex mixture of compounds containing acid **9d** (yield 6%, judging by ^19^F NMR with an internal standard) and acid **13** (yield 14%, ^19^F NMR), along with a complex mixture of other products.

#### 3.3.9. Carbonylation of 1-(Chloromethyl)-2,3,5,6-tetrafluoro-4-(trifluoromethyl)benzene (**8d**)

Compound **8d** (0.508 g, 1.91 mmol), SbF_5_ (2.454 g, 11.33 mmol) (molar ratio 1:5.9), and (C_4_F_9_)_2_O (0.2 mL), according to the typical procedure (Section 3.2a) (2.5 h, r.t.), gave a complex mixture of compounds. After evaporation of Et_2_O, the residue was dissolved in CH_2_Cl_2_ (10 mL) and washed with a saturated aqueous solution of NaHCO_3_ (30 mL). The resulting organic layer contained (GS-MS) a mixture of dimeric and oligomeric products with M ≥ 448. The aqueous layer was acidified with HCl (pH < 1), and extracted with Et_2_O (2 × 5 mL). The extract contain acid **9d** (yield 6%, ^19^F NMR) along with a complex mixture of other products, including acid **13** (yield 4%, ^19^F NMR).

#### 3.3.10. Carbonylation of 1-(Chloromethyl)-2,4,5,6-tetrafluoro-3-(trifluoromethyl)benzene (**8e**)

Compound **8e** (0.343 g, 1.29 mmol), SbF_5_ (1.686 g, 7.79 mmol) (molar ratio 1:6), and (C_4_F_9_)_2_O (0.2 mL), according to the typical procedure (Section 3.2a) (45 min, r.t.), gave a mixture of compounds **9e** and **11** in a 90:10 molar ratio. After evaporation of Et_2_O, the residue was dissolved in CH_2_Cl_2_ (10 mL) and washed with a saturated aqueous solution of NaHCO_3_ (30 mL). The aqueous layer was separated, acidified with HCl (pH < 1), and extracted with CH_2_Cl_2_ (3 × 5 mL). Evaporation of the solvent gave 0.303 g of acid **9e** (yield 85%).

*(2,4,5,6-Tetrafluoro-3-(trifluoromethyl)phenyl)acetic acid (**9e**).* White solid; mp 75–76 °C (after sublimation). IR (KBr) ν, cm^−1^: 1726 (C=O), 1510 (FAR). ^1^H NMR (CDCl_3_): *δ* 6.2 (br.s, 1H, COOH), 3.78 (s, 2H, CH_2_). ^19^F NMR (CDCl_3_): *δ* −57.3 (t, 3F, CF_3_), −119.1 (qdddt, 1F, F-2), −128.8 (dddm, 1F, F-6), −134.1 (qddd, 1F, F-4), −163.0 (ddd, 1F, F-5); *J*_CF3,F(2)_ = *J*_CF3,F(4)_ = 22.5, *J*_F(2),CH2_ = 1.5, *J*_2,4_ = 3.5, *J*_2,5_ = 11.5, *J*_2,6_ = 4, *J*_4,5_ = 21.5, *J*_4,6_ = 10.5, *J*_5,6_ = 22. HRMS, *m*/*z*: calcd. for C_9_H_3_O_2_F_7_ 276.0016; found 276.0014. Anal. calcd. for C_9_H_3_O_2_F_7_: C, 39.15; H, 1.10; F, 48.17%. Found: C, 39.70; H, 1.30; F, 48.16%.

#### 3.3.11. Carbonylation of 1-(Chloromethyl)-3,4,5,6-tetrafluoro-2-(trifluoromethyl)benzene (**8f**)

Compound **8f** (0.461 g, 1.73 mmol), SbF_5_ (2.214 g, 10.22 mmol) (molar ratio 1:5.9), and (C_4_F_9_)_2_O (0.2 mL), according to the typical procedure (Section 3.2a) (45 min, r.t.), gave a mixture of compounds **9f**, **10**, and 4,5,6,7-tetrafluorophthalide in a 92:3:5 molar ratio. After evaporation of Et_2_O, the residue was dissolved in CH_2_Cl_2_ (10 mL) and washed with a saturated aqueous solution of NaHCO_3_ (30 mL). The aqueous layer was separated, acidified with HCl (pH < 1), and extracted with CH_2_Cl_2_ (3 × 5 mL). Evaporation of the solvent gave 0.420 g of acid **9f** (yield 88%).

*(3,4,5,6-Tetrafluoro-2-(trifluoromethyl)phenyl)acetic acid (**9f**).* White solid; mp 73.5–75 °C (after sublimation). IR (KBr) ν, cm^−1^: 1726 (C=O), 1529, 1485 (FAR). ^1^H NMR (acetone-*d_6_*): *δ* 4.2 (br.s, 1H, COOH), 3.96 (s, 2H, CH_2_). ^19^F NMR (acetone-*d_6_*): *δ* −54.0 (d, 3F, CF_3_), −138.1 (ddm, 1F, F-6), −139.0 (qddd, 1F, F-3), −149.7 (ddd, 1F, F-5), −154.9 (ddd, 1F, F-4); *J*_CF3,F(3)_ = 26, *J*_3,4_ = 21, *J*_3,5_ = 7.5, *J*_3,6_ = 11.5, *J*_4,6_ = 4.5, *J*_4,5_ = 21, *J*_5,6_ = 22. HRMS, *m*/*z*: calcd. for C_9_H_3_O_2_F_7_ 276.0016; found 276.0017. Anal. calcd. for C_9_H_3_O_2_F_7_: C, 39.15; H, 1.10; F, 48.17%. Found: C, 39.56; H, 1.08; F, 48.26%.

#### 3.3.12. Carbonylation of 1-(Chloromethyl)-2,3,5,6-tetrafluoro-4-(perfluoroisopropyl)benzene (**8g**)

Compound **8g** (0.502 g, 1.37 mmol), SbF_5_ (1.825 g, 8.43 mmol) (molar ratio 1:6.1), and (C_4_F_9_)_2_O (0.2 mL), according to the typical procedure (Section 3.2a) (1.5 h, r.t.), gave a complex mixture of compounds containing ~20% of acid **9g**. After evaporation of Et_2_O, the residue was dissolved in CH_2_Cl_2_ (10 mL) and washed with a saturated aqueous solution of NaHCO_3_ (30 mL). The aqueous layer was separated, acidified with HCl (pH < 1), and extracted with CH_2_Cl_2_ (3 × 5 mL). Evaporation of the solvent and sublimation (130 °C, 1 Torr) resulted in a mixture (0.110 g) containing ~90% of acid **9g**, recrystallization from hexane gave 55 mg (yield 11%) of the pure substance.

*(2,3,5,6-Tetrafluoro-4-(perfluoroisopropyl)phenyl)acetic acid (**9g**).* White solid; mp 117–118 °C (hexane). IR (KBr) ν, cm^−1^: 1716 (C=O), 1493 (FAR). ^1^H NMR (CDCl_3_): *δ* 6.5 (br.s, 1H, COOH), 3.87 (s, 2H, CH_2_). ^19^F NMR (CDCl_3_): *δ* −76.4 (m, 6F, CF_3_), −135.6 (br.s, 1F, Ar-F), −138.6 (br.s, 1F, Ar-F), −140.4 (br.s, 1F, Ar-F), −141.4 (br.s, 1F, Ar-F), −179.7 (m, 1F, CF(CF_3_)_2_). HRMS, *m*/*z*: calcd. for C_11_H_3_O_2_F_11_ 375.9952; found 375.9950.

#### 3.3.13. Carbonylation of (Perfluoro-[1,1′-biphenyl]-4-yl)acetic acid (**8h**)

Compound **8h** (0.498 g, 1.37 mmol), SbF_5_ (1.776 g, 8.20 mmol) (molar ratio 1:6), and (C_4_F_9_)_2_O (0.3 mL), according to the typical procedure (Section 3.2a) (2 h, r.t.), gave a complex mixture of compounds containing ~20% of starting compound **8h** and ~5% acid **9h**. After evaporation of Et_2_O, the residue was dissolved in CH_2_Cl_2_ (10 mL) and washed with a saturated aqueous solution of NaHCO_3_ (30 mL). The aqueous layer was separated, acidified with HCl (pH < 1), and extracted with Et_2_O (2 × 5 mL). Evaporation of the solvent, sublimation (180 °C, 1 Torr), and recrystallization from hexane afforded 14 mg of acid **9h** (yield 3%).

*(Perfluoro-[1,1′-biphenyl]-4-yl)acetic acid (**9h**).* White solid; mp 211–214 °C (after sublimation). IR (KBr) ν, cm^−1^: 1709 (C=O), 1489, 1417 (FAR). ^1^H NMR (acetone-*d_6_*): *δ* 3.96 (s, 2H, CH_2_), 3.3 (br.s, COOH + traces of water). ^19^F NMR (acetone-*d_6_*): *δ* −138.0 (m, 2F, Ar-F), −139.5 (m, 2F, Ar-F), −141.5 (m, 2F, Ar-F), −151.0 (tt, 1F, F-4′, *J*_4′,3′_ = *J*_4′,5′_ = 20.5, *J*_4′,2′_ = *J*_4′,6′_ = 3), −161.4 (m, 2F, F-3′,5′). HRMS, *m*/*z*: calcd. for C_14_H_3_O_2_F_9_ 373.9984; found 373.9985.

#### 3.3.14. Carbonylation of (Perfluoro-4-methylphenyl)methanol (**14d**)

A mixture of alcohol **14d** (0.145 g, 0.58 mmol), TfOH (0.263 g, 1.75 mmol), and SbF_5_ (0.379 g, 1.75 mmol) (molar ratio 1:3:3) was vigorously stirred in a 10 mL round-bottom glass flask (7 h, 60 °C) in a slow flow of CO, then poured into 5% hydrochloric acid (15 mL), extracted three times with 4 mL of a CH_2_Cl_2_–Et_2_O mixture (3:1, *v*/*v*), and the extract was dried over MgSO_4_. The resulting solution contained compounds **9d** and **12** in an 88:12 molar ratio. After evaporation of the solvent, the residue was dissolved in CH_2_Cl_2_ (10 mL) and washed with a saturated aqueous solution of NaHCO_3_ (30 mL). The aqueous layer was separated, acidified with HCl (pH < 1), and extracted with CH_2_Cl_2_ (3 × 5 mL). Evaporation of the solvent and sublimation (120 °C, 3 Torr) gave 0.104 g of acid **9d** (yield 65%).

*(2,3,5,6-Tetrafluoro-4-(trifluoromethyl)phenyl)acetic acid (**9d**).* White solid; mp 77–80 °C (after sublimation). IR (KBr) ν, cm^−1^: 1732 (C=O), 1497 (FAR). ^1^H NMR (CDCl_3_): *δ* 11.67 (br.s, 1H, COOH), 3.87 (s, 2H, CH_2_). ^19^F NMR (CDCl_3_): *δ* −57.4 (t, 3F, *J*_CF3,F(3)_ = *J*_CF3,F(5)_ = 21.5, CF_3_), −141.2 (m, 2F, F-2,6), −141.6 (m, 2F, F-3,5). HRMS, *m*/*z*: calcd. for C_9_H_3_O_2_F_7_ 276.0016; found 276.0013.

#### 3.3.15. Carbonylation of (Perfluoro-3-methylphenyl)methanol (**14e**)

Compound **14e** (0.198 g, 0.80 mmol) and TfOH (0.840 g, 5.60 mmol) (molar ratio 1:7), used similarly to procedure 3.3.14 (6 h, 50 °C, extraction with CH_2_Cl_2_), gave a mixture of compounds containing ~95% of acid **9e**. An analogous treatment gave 0.165 g of acid **9e** (yield 75%).

#### 3.3.16. Carbonylation of (Perfluoro-2-methylphenyl)methanol (**14f**)

Compound **14f** (0.218 g, 0.88 mmol) and TfOH (0.923 g, 6.15 mmol) (molar ratio 1:7), similarly to procedure 3.3.14 (6 h, 75 °C), gave a mixture of compounds containing ~85% of acid **9f** in the absence of acid **10** after hydrolysis of the reaction mixture. An analogous treatment gave 0.155 g of acid **9f** (yield 64%).

#### 3.3.17. Carbonylation of 1,4-Bis(chloromethyl)-2,3,5,6-tetrafluorobenzene (**16a**)

Compound **16a** (0.174 g, 0.70 mmol), SbF_5_ (0.974 g, 4.50 mmol) (molar ratio 1:6.4), and (C_4_F_9_)_2_O (0.1 mL), according to the typical procedure (Section 3.2b) (2 h, r.t., 20 min in methanol), gave a complex mixture of compounds containing ~15% ester **17′a** (yield 12%, ^19^F NMR).

*Dimethyl (2,3,5,6-tetrafluoro-1,3-phenylene)diacetate (**17′a**)* in the reaction mixture. ^1^H NMR (CDCl_3_): *δ* 3.72 (s, 6H, CH_3_), 3.71 (s, 4H, CH_2_).^19^F NMR (CDCl_3_): *δ* −144.3 (s, 4F, F-Ar). GC-MS, *m*/*z*: calcd. for C_12_H_10_O_4_F_4_ 294; found 294.

#### 3.3.18. Carbonylation of 1,3-Bis(chloromethyl)-2,4,5,6-tetrafluorobenzene (**16b**)

Compound **16b** (0.272 g, 1.10 mmol), SbF_5_ (1.555 g, 7.18 mmol) (molar ratio 1:6.5), and (C_4_F_9_)_2_O (0.2 mL), according to the typical procedure (Section 3.2b) (2 h, r.t., 20 min in methanol), gave a mixture of compounds containing ~65% of ester **17′b**. Silica gel column chromatography (CH_2_Cl_2_ as the eluent) gave 0.143 g of ester **17′b** (yield 44%).

*Dimethyl (2,4,5,6-tetrafluoro-1,3-phenylene)diacetate (**17′b**).* Colorless liquid. IR (film) ν, cm^−1^: 2958 (CH), 1747 (C=O), 1496 (FAR). ^1^H NMR (CDCl_3_): *δ* 3.68 (s, 6H, CH_3_), 3.66 (s, 4H, CH_2_).^19^F NMR (CDCl_3_): *δ* −123.3 (d, 1F, F-2), −137.8 (d, 2F, F-4,6), −164.0 (td, 1F, F-5); *J*_2,5_ = 11.5, *J*_4,5_ = *J*_5,6_ = 21.5. HRMS, *m*/*z*: calcd. for C_12_H_10_O_4_F_4_ 294.0510; found 294.0509.

#### 3.3.19. Carbonylation of 1,2-Bis(chloromethyl)-3,4,5,6-tetrafluorobenzene (**16c**)

*a*. Compound **16c** (0.287 g, 1.16 mmol), SbF_5_ (1.512 g, 6.98 mmol) (molar ratio 1:6), and (C_4_F_9_)_2_O (0.3 mL), according to the typical procedure (Section 3.2b) (1 h, r.t., 20 min in methanol), gave a mixture of compounds **17′c**, **15**, and 4,5,6,7-tetrafluorophthalide in an 86:10:4 molar ratio. Silica gel column chromatography (CH_2_Cl_2_ as the eluent) gave 0.247 g of inseparable mixture of compounds **17′c** and **15** in an 89:11 molar ratio.

*b*. Compound **16c** (0.477 g, 1.93 mmol), SbF_5_ (2.896 g, 13.38 mmol) (molar ratio 1:6.9), and (C_4_F_9_)_2_O (0.7 mL), according to the typical procedure (Section 3.2a) (2.5 h, r.t.), gave a mixture of compounds containing ~80% of acid **17c**. After evaporation of Et_2_O, the residue was dissolved in a saturated aqueous solution of NaHCO_3_ (50 mL), acidified with HCl (pH < 1), and extracted with CH_2_Cl_2_ (3 × 5 mL). The aqueous layer was extracted with Et_2_O (3 × 5 mL). Evaporation of the Et_2_O extract, sublimation (170 °C, 1 Torr), and recrystallization from CCl_4_-acetone resulted in 0.343 g of acid **17c** (yield 67%).

*Dimethyl (3,4,5,6-tetrafluoro-1,2-phenylene)diacetate (**17′c**)* in the mixture with lactone **15** (**17′c**:**15** = 89:11). Colorless liquid. IR (film) ν, cm^−1^: 2958 (CH), 1745 (C=O), 1520, 1487 (FAR). ^1^H NMR (CDCl_3_): *δ* 3.69 (s, 4H, CH_2_), 3.68 (s, 6H, CH_3_).^19^F NMR (CDCl_3_): *δ* −141.8 (m, 2F, F-3,6), −157.9 (m, 2F, F-4,5). HRMS, *m*/*z*: calcd. for C_12_H_10_O_4_F_4_ 294.0510; found 294.0507.

*(3,4,5,6-Tetrafluoro-1,2-phenylene)diacetic acid (**17c**).* White solid; mp 196.5–197.5 °C (CCl_4_-acetone). IR (KBr) ν, cm^−1^: 1720 (C=O), 1518, 1489 (FAR). ^1^H NMR (acetone-*d_6_*): *δ* 5.5 (br.s, 2H, COOH), 3.82 (s, 4H, CH_2_). ^19^F NMR (acetone-*d_6_*): *δ* −141.2 (m, 2F, F-3,6), −159.1 (m, 2F, F-4,5). HRMS, *m*/*z*: calcd. for C_10_H_6_O_4_F_4_ 266.0197; found 266.0193. Anal. calcd for C_10_H_6_O_4_F_4_: C, 45.13; H, 2.27; F, 28.55%. Found: C, 45.56; H, 2.70; F, 28.45%.

#### 3.3.20. Carbonylation of (Fluoromethylene)bis(pentafluorobenzene) (**18**)

*a*. Compound **18** (0.375 g, 1.02 mmol), SbF_5_ (0.112 g, 0.51 mmol) (molar ratio 1:0.5), and C_6_F_6_ (0.5 mL), according to the typical procedure (Section 3.2a) (6 h, r.t.), gave a mixture of compounds containing 45% of acid **19** along with hydrolysis products of bis(perfluophenyl)methyl cation (alcohol **40** and its ether).

*b*. Compound **18** (0.349 g, 0.95 mmol), SbF_5_ (0.205 g, 0.95 mmol) (molar ratio 1:1), and C_6_F_6_ (0.5 mL), according to the typical procedure (Section 3.2a) (4.5 h, r.t.), gave a mixture of compounds containing 50% of acid **20** along with alcohol **40** and its ether. After evaporation of Et_2_O, the residue was dissolved in CH_2_Cl_2_ (10 mL) and washed with a saturated aqueous solution of NaHCO_3_ (30 mL). The aqueous layer was separated, acidified with HCl (pH < 1), and extracted with CH_2_Cl_2_ (3 × 5 mL). Evaporation of the solvent gave 0.164 g of acid **20** (yield 44%).

*c*. Compound **18** (0.385 g, 1.05 mmol), SbF_5_ (0.800 g, 3.70 mmol) (molar ratio 1:6), and C_6_F_6_ (0.4 mL), according to the typical procedure (Section 3.2a) (4.5 h, r.t.), gave a mixture of compounds containing 5% of acid **20** along with alcohol **40** and its ether.

*d*. Compound **18** (0.222 g, 0.61 mmol) and SbF_5_ (0.800 g, 3.70 mmol) (molar ratio 1:6), according to the typical procedure (Section 3.2a) (5.5 h, r.t.), gave a mixture of compounds containing 3% of acid **20** along with alcohol **40** and its ether.

#### 3.3.21. Carbonylation of (Chloromethylene)bis(pentafluorobenzene) (**19**)

*a*. Compound **19** (0.383 g, 1.00 mmol) and SbF_5_ (0.111 g, 0.51 mmol) (molar ratio 1:0.5), according to the typical procedure (Section 3.2a) (4.5 h, r.t.), gave a mixture of compounds containing 88% of acid **20** along with perfluorodiphenylmethane (5%), perfluorobenzophenone (2%), alcohol **40**, its ether, and the starting compound. After the evaporation of Et_2_O, the residue was dissolved in CH_2_Cl_2_ (10 mL) and washed with a saturated aqueous solution of NaHCO_3_ (30 mL). The aqueous layer was separated, acidified with HCl (pH < 1), and extracted with CH_2_Cl_2_ (3 × 5 mL). Evaporation of the solvent gave 0.335 g of acid **20** (yield 85%).

*b*. Compound **19** (0.468 g, 1.22 mmol) and SbF_5_ (0.262 g, 1.21 mmol) (molar ratio 1:1), according to the typical procedure (Section 3.2a) (4.5 h, r.t.), gave a mixture of compounds containing 45% of acid **20** along with perfluorodiphenylmethane (2%), perfluorobenzophenone (3%), alcohol **40**, its ether and the starting compound.

*c*. Compound **19** (0.342 g, 0.89 mmol) and SbF_5_ (1.167 g, 5.39 mmol) (molar ratio 1:6), according to the typical procedure (Section 3.2a) (4.5 h, r.t.), gave a mixture of alcohol **40** and its ether with admixture of the starting compound, and the content of acid **20** in the mixture did not exceed 1%.

#### 3.3.22. Carbonylation of Compound (1-Chloro-2,2,2-trifluoroethyl)pentafluorobenzene (**23a**)

*a*. Compound **23a** (0.528 g, 1.86 mmol) and SbF_5_ (0.200 g, 0.92 mmol) (molar ratio 1:0.5), according to the typical procedure (Section 3.2a) (1 h, r.t.), gave a mixture of compounds **23a** and **24a** in a 29:71 molar ratio.

*b*. Compound **23a** (0.436 g, 1.53 mmol) and SbF_5_ (0.322 g, 1.53 mmol) (molar ratio 1:1), according to the typical procedure (Section 3.2a) (1 h, r.t.), after evaporation of the solvent and sublimation (120 °C, 3 Torr), afforded 0.416 g of acid **24a** (yield 92%) with a 3% admixture of acid **9a**.

*c*. Compound **23a** (0.567 g, 2.00 mmol) and SbF_5_ (0.428 g, 1.98 mmol) (molar ratio 1:1), according to the typical procedure (Section 3.2b) (1.5 h, r.t., 15 min in methanol), after washing of the extract with a saturated aqueous solution of NaHCO_3_ (30 mL) and evaporation of the solvent gave 0.556 g of ester **24′a** (yield 90%).

*d*. Compound **23a** (0.358 g, 1.35 mmol) and SbF_5_ (1.758 g, 8.12 mmol) (molar ratio 1:6), according to the typical procedure (Section 3.2a) (0.5 h, r.t.), after evaporation of the solvent and sublimation (120 °C, 1 Torr), afforded 0.380 g of acid **24a** (yield 96%) with a 2% admixture of acid **9a**.

*e*. Compound **23a** (0.347 g, 1.22 mmol) and SbF_5_ (1.560 g, 7.21 mmol) (molar ratio 1:5.9), according to the typical procedure (Section 3.2a) (5 h, 70 °C), gave a mixture of compounds **24a**, **25a**, **3a**, **9a** in the following molar ratios: 28:25:42:5 (2.5 h after extraction, r.t.); 28:2:38:32 (24 h after extraction, r.t.).

*f*. Compound **23a** (0.362 g, 1.27 mmol) and SbF_5_ (1.603 g, 7.40 mmol) (molar ratio 1:5.8), according to the typical procedure (Section 3.2b) (7 h, 70 °C, 15 min in methanol) gave a mixture of compounds which did not contain acid **25a** or its methyl ester. The main products in the mixture were compounds **28′**, **3′a**, **24′a**, and **24a** in a 53:21:13:13 molar ratio with total content ~90%. The extract was washed with a saturated aqueous solution of NaHCO_3_ (30 mL), and silica gel column chromatography (CCl_4_–CH_2_Cl_2_ [1:1, *v*/*v*] as the eluent) gave 88 mg of compound **28′** (yield 22%).

*Perfluoro-2-phenylpropenoic acid (**25a**)* was in the mixture with acids **24a**, **3a**, **9a**. ^19^F NMR (CDCl_3_): *δ* −58.4 (dt, 1F, F-3A), −59.1 (dt, 1F, F-3B), −138.5 (m, 2F, F-*ortho*), −152.3 (tt, 1F, F-*para*), −162.3 (m, 2F, F-*meta*); *J*_3Z,3E_ = 38, *J*_3Z,*ortho*_ = 1.5, *J*_3E,*ortho*_ = 6, *J_para_*_,*meta*_ = 21, *J_para_*_,*ortho*_ = 2.5.

*Methyl 3,3-difluoro-3-methoxy-2-perfluorophenylpropanoate (**28′**).* Colorless liquid. IR (film) ν, cm^−1^: 2964 (CH), 1770 (C=O), 1525, 1508 (FAR). ^1^H NMR (CDCl_3_): *δ* 4.56 (t, 1H, *J*_H(2),F(3)_ = 8, H-2), 3.76 (s, 3H, CO_2_CH_3_), 3.57 (s, 3H, CF_2_OCH_3_). ^19^F NMR (CDCl_3_): *δ* −77.6 (dq, 1F, F_A_-3), −78.0 (dq, 1F, F_B_-3), −140.4 (m, 2F, F-*ortho*), −153.9 (tt, 1F, F-*para*), −162.7 (m, 2F, F-*meta*); *J*_3A,3B_ = 141, *J*_3,*ortho*_ = *J*_H(2),F(3)_ = 8, *J_para_*_,*meta*_ = 21, *J_para_*_,*ortho*_ = 2.5. HRMS, *m*/*z*: calcd. for C_11_H_7_O_3_F_7_ 320.0278; found 320.0278.

#### 3.3.23. Carbonylation of (1,2,2,3,3,3-Hexafluoropropyl)pentafluorobenzene (**22b**)

*a*. Compound **22b** (0.376 g, 1.18 mmol) and SbF_5_ (0.253 g, 1.17 mmol) (molar ratio 1:1), according to the typical procedure (Section 3.2a) (0.5 h, r.t.), gave a mixture of compounds **24b**, **25b** (*E:Z* = 55:45), and **26b** in an 86:9:5 molar ratio. After evaporation of Et_2_O, the residue was dissolved in CH_2_Cl_2_ (10 mL) and washed with a saturated aqueous solution of NaHCO_3_ (30 mL). The resulting solution contained mainly compound **26b**. The aqueous layer was separated, acidified with HCl (pH < 1), and extracted with Et_2_O (3 × 5 mL). Evaporation of the solvent and sublimation (120 °C, 3 Torr) resulted in a mixture (0.345 g) of acids **24b** and **25b** in a 90:10 molar ratio.

*b*. Compound **22b** (0.276 g, 0.87 mmol) and SbF_5_ (0.388 g, 1.79 mmol) (molar ratio 1:2), according to the typical procedure (Section 3.2a) (0.5 h, r.t.), gave a mixture of compounds **24b**, **25b** (*E:Z* = 60:40), and **26b** in a 90:9:1 molar ratio.

*c*. Compound **22b** (0.210 g, 0.66 mmol) and SbF_5_ (0.583 g, 2.69 mmol) (molar ratio 1:4), according to the typical procedure (Section 3.2a) (0.5 h, r.t.), gave a mixture of compounds **24b** and **25b** (*E:Z* = 60:40) in a 95:5 molar ratio.

*d*. Compound **22b** (0.378 g, 1.19 mmol) and SbF_5_ (1.556 g, 7.19 mmol) (molar ratio 1:6), according to the typical procedure (Section 3.2a) (0.5 h, r.t.), gave a mixture of compounds **24b** and **25b** (*E:Z* = 60:40) in a 95:5 molar ratio. Evaporation of the solvent, sublimation (90 °C, 1 Torr), and recrystallization from hexane resulted in 0.333 g of acid **24b** (yield 81%).

*e*. Compound **22b** (0.359 g, 1.13 mmol) and SbF_5_ (1.458 g, 6.73 mmol) (molar ratio 1:6), according to the typical procedure (Section 3.2b) (6 h, 70 °C, 10 min in methanol), gave a mixture of compounds **25′b** (*E:Z* = 85:15) and **25b** (*E:Z* = 60:40) in a 80:20 molar ratio with total content ~90%. Methyl ester **24′b** was not detected in the mixture. Silica gel column chromatography (CCl_4_–CH_2_Cl_2_ [1:1, *v*/*v*] as the eluent) gave 0.263 g of compound **25′b** (yield 69%).

*1,1,1-Trifluoro-3-(perfluorophenyl)propan-2-one (**26b**) in the mixture.*^1^H NMR (CDCl_3_): *δ* 4.10 (s, 2H, CH_2_). ^19^F NMR (CDCl_3_): *δ* −79.9 (s, 3F, CF_3_), −143.2 (m, 2F, F-*ortho*), −153.8 (t, 1F, *J_para,meta_* = 20.5, F-*para*), −162.1 (m, 2F, F-*meta*). GC-MS, *m*/*z*: calcd. for C_9_H_2_O_1_F_8_ 278; found 278.

#### 3.3.24. Carbonylation of (1-Chloro-2,2,3,3,3-pentafluoropropyl)pentafluorobenzene (**23b**) (With Admixture of P-chloroderivative 45, See Procedure 3.4.4e)

*a*. Compound **23b** (0.360 g, 1.08 mmol) and SbF_5_ (0.238 g, 1.10 mmol) (molar ratio 1:1), according to the typical procedure (Section 3.2a) (1 h, r.t.), gave a mixture of compounds **24b**, **25b**, and **26b** in an 89:5:6 molar ratio (with admixtures of the corresponding *p*-chloroderivatives). 

*b*. Compound **23b** (0.335 g, 1.00 mmol) and SbF_5_ (1.295 g, 5.98 mmol) (molar ratio 1:6), according to the typical procedure (Section 3.2a) (1 h, r.t.), gave a mixture of compounds **24b** and **25b** (*E:Z* = 60:40) in a 95:5 molar ratio (with admixtures of the corresponding *p*-chloroderivatives). Similar procedure with a reaction time of 5 h gave a mixture of compounds **24b** and **25b** (*E:Z* = 60:40) in a 91:9 molar ratio.

*c*. Compound **23b** (0.358 g, 1.07 mmol) and SbF_5_ (1.390 g, 6.42 mmol) (molar ratio 1:6), according to the typical procedure (Section 3.2a) (1 h, 70 °C), gave a mixture of compounds **24b**, **25b**, and **26b** in the 36:60:4 molar ratio (with admixtures of the corresponding *p*-chloroderivatives). Similar procedure with a reaction time of 5 h gave a mixture of compounds **24a**, **25b**, and **26b** in a 2:92:6 molar ratio.

#### 3.3.25. Carbonylation of 1-Chloro-2,2,3,3,4,4,5,6,7,8-decafluorotetralin (**23c**) (With Admixture of Isomer 47, See Procedure 3.4.4f)

*a*. Compound **23c** (0.346 g, 1.00 mmol) and SbF_5_ (0.212 g, 0.98 mmol) (molar ratio 1:1), according to the typical procedure (Section 3.2a) (1 h, r.t.), gave a mixture of compounds **24c**, **25c**, and **26c** in an 81:7:12 molar ratio (with 8% of admixtures of the corresponding 6-chloroderivatives). After evaporation of Et_2_O, the residue was dissolved in CH_2_Cl_2_ (10 mL) and washed with a saturated aqueous solution of NaHCO_3_ (30 mL). The aqueous layer was separated, acidified with HCl (pH < 1), and extracted with Et_2_O (3 × 5 mL). Evaporation of the solvent, sublimation (100 °C, 1 Torr), and recrystallization from hexane resulted in 0.210 g of acid **24c** (yield 59%). The product contained 3% of the 6-chloroderivative **48**.

*b*. Compound **23c** (0.380 g, 1.10 mmol) and SbF_5_ (1.413 g, 6.53 mmol) (molar ratio 1:6), according to the typical procedure (Section 3.2a) (1 h, r.t.), gave a mixture of compounds **24c** and **25c** in an 87:13 molar ratio (with 8% admixtures of 6-chloroderivatives **48** and **49**), the content of compound **26c** in the mixture did not exceed 2%.

*c*. Compound **23c** (0.356 g, 1.03 mmol) and SbF_5_ (1.343 g, 6.20 mmol) (molar ratio 1:6), according to the typical procedure (Section 3.2a) (1 h, 70 °C), gave a mixture of compounds **25c** and **26c** in a 94:6 molar ratio (with 6% admixtures of the corresponding 6-chloroderivatives). After evaporation of Et_2_O, the residue was dissolved in CH_2_Cl_2_ (10 mL) and washed with a saturated aqueous solution of NaHCO_3_ (30 mL). The aqueous layer was separated, acidified with HCl (pH < 1), and extracted with CH_2_Cl_2_ (3 × 5 mL). Evaporation of the solvent, sublimation (110 °C, 1 Torr), and recrystallization from hexane resulted in 0.234 g of acid **25c** (yield 68%), the product contained 3% of 6-chloroderivative **49**.

*Perfluoro-3,4-dihydronaphthalene-1-carboxylic acid (**25c**).* White solid; mp 102.5–104.5 °C (hexane). IR (KBr) ν, cm^−1^: 1732 (C=O), 1520, 1497 (FAR). ^1^H NMR (CDCl_3_): *δ* 10.08 (s, 1H, CO_2_H). ^19^F NMR (CDCl_3_): *δ* −120.0 (tm, 1F, F-3), −120.5 (dm, 2F, F-4), −130.9 (m, 2F, F-3), −133.9 (dddd, 1F, F-8), −136.5 (tdddd, 1F, F-5), −147.0 (tdq, 1F, F-7), −149.2 (dddd, 1F, F-6); *J*_2,3_ = 18, *J*_2,5_ = 2, *J*_2,6_ = 7, *J*_2,7_ = 2, *J*_2,8_ = 6, *J*_5,6_ = 20.5, *J*_5,7_ = 9, *J*_5,8_ = 11.5, *J*_6,7_ = 20, *J*_6,8_ = 6, *J*_7,8_ = 20. HRMS, *m*/*z*: calcd. for C_11_H_1_O_2_F_9_ 335.9829; found 335.9827. Anal. calcd. for C_11_H_1_O_2_F_9_: C, 39.31; H, 0.30; F, 50.87%. Found: C, 38.83; H, 0.50; F, 50.80%.

#### 3.3.26. Carbonylation of 1,2,2,3,3,4,5,6,7-Nonafluoroindan (**22d**)

*a*. Compound **22d** (0.422 g, 1.51 mmol) and SbF_5_ (0.314 g, 1.45 mmol) (molar ratio 1:1), according to the typical procedure (Section 3.2a) (0.5 h, r.t.), gave a mixture of compounds containing ~95% of acid **24d**. The content of acid **25d** in the mixture did not exceed 2%. Evaporation of the solvent, sublimation (105 °C, 1 Torr), and recrystallization from CCl_4_-hexane resulted in 0.411 g of acid **24d** (yield 79%).

*b*. Compound **22d** (0.676 g, 2.41 mmol) and SbF_5_ (3.083 g, 14.24 mmol) (molar ratio 1:5.9), according to the typical procedure (Section 3.2b) (7 h, 70 °C, 20 min in methanol), gave a mixture of compounds **24′d**, **29′**, **30**, and **31** in a 6:51:35:8. Silica gel column chromatography (CH_2_Cl_2_ as the eluent) gave 0.138 g of compounds **29′** (yield 18%).

*Dimethyl 2,4,5,6,7-pentafluoro-1H-indene-1,3-dicarboxylate (**29′**).* Colorless liquid. IR (film) ν, cm^−1^: 2960 (CH), 1741 (C=O), 1512, 1493 (FAR). ^1^H NMR (CDCl_3_): *δ* 4.69 (s, 1H, H-1), 3.90 (s, 3H, CO_2_CH_3_-3), 3.79 (s, 3H, CO_2_CH_3_-1). ^19^F NMR (CDCl_3_): *δ* −101.7 (dd, 1F, F-2), −139.9 (dddd, 1F, F-4), −142.0 (ddm, 1F, F-7), −154.5 (ddd, 1F, F-5), −157.9 (dddd, 1F, F-6); *J*_2,4_ = 5, *J*_2,6_ = 14, *J*_4,5_ = 20, *J*_4,6_ = 2.5, *J*_4,7_ = 15, *J*_5,6_ = 19, *J*_5,7_ = 3.5, *J*_6,7_ = 21. HRMS, *m*/*z*: calcd. for C_13_H_7_O_4_F_5_ 322.0259; found 322.0256.

#### 3.3.27. Carbonylation of 1-Chloro-2,2,3,3,4,5,6,7-octafluoroindan (**23d**)

*a*. Compound **23d** (0.422 g, 1.42 mmol) and SbF_5_ (0.310 g, 1.43 mmol) (molar ratio 1:1), according to the typical procedure (Section 3.2a) (1 h, r.t.), gave a mixture of compounds containing 50% of acid **24d** and 9% of starting compound **23d** along with their derivatives with moieties C=O, CFCl, CCl_2_ instead of CF_2_ in the benzylic position.

*b*. Compound **23d** (0.358 g, 1.30 mmol) and SbF_5_ (1.693 g, 7.82 mmol) (molar ratio 1:6), according to the typical procedure (Section 3.2a) (0.5 h, r.t.), gave a mixture of compounds containing 50% of acid **24d** along with 3-X-2,2,4,5,6,7-hexafluoroindan-1-ones (X = COOH, Cl, F), the content of acid **25d** in the mixture did not exceed 2%.

#### 3.3.28. Carbonylation of 1,4-Dichloro-2,2,3,3,5,6,7,8-octafluorotetralin (**36**) (With Admixtures of Isomeric Compounds, See Procedure 3.4.4h)

*a*. Compound **36** (0.395 g, 1.14 mmol) and SbF_5_ (0.242 g, 1.12 mmol) (molar ratio 1:1), according to the typical procedure (Section 3.2b) (0.5 h, r.t., 26 h in methanol), gave a mixture of compounds **36**, **37′**, **38′**, **39′**, and dimethyl 1,2,2,3,3,5,6,7,8-nonafluorotetralin-1,4-dicarboxylate in a 7:66:10:1:16 molar ratio (with admixtures of the corresponding chloroderivatives). After evaporation of a solvent, the residue was dissolved in a solution of NEt_3_ (0.770 g) in dry CH_2_Cl_2_ (8 mL). The solution was kept at r.t. for 1 h, washed with 5% hydrochloric acid (20 mL), and dried over MgSO_4_. The mixture of compounds in the solution contained **38′**, **39′**, and 1,4-dichlorohexafluoronaphthalene in a 76:17:7 molar ratio. Silica gel column chromatography (CCl_4_–CH_2_Cl_2_ [1:1, *v*/*v*] as the eluent) gave 0.268 g of compound **38′** (yield 71%). The product contained 9% of the 6- and 7-chloroderivatives.

*b*. Compound **36** (0.316 g, 0.92 mmol) and SbF_5_ (1.200 g, 5.54 mmol) (molar ratio 1:6), according to the typical procedure (Section 3.2b) (3 h, r.t., 28 h in methanol), gave a mixture of compounds containing (GC-MS) 73% of ester **39′**, 9% of its 6-chloroderivative **53′**, and 3% of ester **38′**. Silica gel column chromatography (CCl_4_–CH_2_Cl_2_ [1:1, *v*/*v*] as the eluent) gave 0.220 g of compound **39′** (yield 68%), the product contained 8% of the 6-chloroderivative **53′**.

*Methyl 4-chloro-2,2,3,3,5,6,7,8-octafluorotetralin-1-carboxylate (**37′**)* (isomers A:B = 83:17) in the mixture with other products. Isomer A. ^19^F NMR (CDCl_3_): −112.8 (dm, 1F, *J*_A,B_ = 264, CF_2_-F_A_), −116.6 (dm, 1F, *J*_A,B_ = 263, CF_2_-F_A_), −116.8 (dm, 1F, *J*_A,B_ = 264, CF_2_-F_B_), −128.2 (dm, 1F, *J*_A,B_ = 263, CF_2_-F_B_), −136.5 (dddd, 1F, F-8), −138.8 (ddd, 1F, F-5), −151.9 (ddd, 1F, F-7), −153.3 (dddd, 1F, F-6); *J*_5,6_ = 20.5, *J*_5,7_ = 5.5, *J*_5,8_ = 11.5, *J*_6,7_ = 20.5, *J*_6,8_ = 5, *J*_F(6),H_ = 2, *J*_7,8_ = 20.5, *J*_F(8),H_ = 1.5.

Isomer B. ^19^F NMR (CDCl_3_): −115.5 (dm, 1F, *J*_A,B_ = 265, CF_2_-F_A_), −115.9 ÷ −117.4 (2F, CF_2_-F_A_,F_B_), −127.3 (dm, 1F, *J*_A,B_ = 265, CF_2_-F_B_), −137.2 (ddd, 1F, F-5), −138.7 (dddd, 1F, F-8), −152.1 (ddd, 1F, F-7), −153.2 (ddd, 1F, F-6); *J*_5,6_ = 20.5, *J*_5,7_ = 6, *J*_5,8_ = 11.5, *J*_6,7_ = 20.5, *J*_6,8_ = 5, *J*_7,8_ = 20.5, *J*_F(8),H_ = 1.5.

*Methyl perfluoro-4-chloro-1-naphthoate (**38′**).* White solid; mp 61–62.5 °C (hexane). IR (KBr) ν, cm^−1^: 2962 (CH), 1749 (C=O), 1527, 1508, 1465, 1450 (FAR). ^1^H NMR (CDCl_3_): *δ* 4.02 (s, 3H, CH_3_). ^19^F NMR (CDCl_3_): *δ* −129.7 (m, 1F, F-3), −133.4 (m, 1F, F-2), −142.3 (m, 2F, F-5,8), −154.4 (m, 2F, F-6,7). HRMS, *m*/*z*: calcd. for C_12_H_3_O_2_Cl_1_F_6_ 327.9720; found 327.9718.

*Dimethyl perfluoro-6-chloronaphthalene-1,4-dicarboxylate (**53′**)* in a mixture with compound **39′** (**39′**:**53′** = 92:8). ^1^H NMR (CDCl_3_): *δ* 4.01 (s, 6H, CH_3_). ^19^F NMR (CDCl_3_): *δ* −118.7 (ddd, 1F, F-5), −133.4 (dddd, 1F, F-2), −134.9 (dddd, 1F, F-3), −136.5 (ddd, 1F, F-7), −144.0 (dddd, 1F, F-8); *J*_2,3_ = 21.5, *J*_2,5_ = 4, *J*_2,7_ = 2, *J*_2,8_ = 6, *J*_3,5_ = 5.5, *J*_3,7_ = 8.5, *J*_3,8_ = 4, *J*_5,8_ = 15.5, *J*_7,8_ = 18. HRMS, *m*/*z*: calcd. for C_14_H_6_O_2_Cl_1_F_5_ 367.9869; found 367.9865.

### 3.4. Synthesis of the Starting Compounds

#### 3.4.1. Synthesis of Alcohols **14d**–**h**

*a*. A mixture of acid **42d** (1.460 g, 5.57 mmol), SOCl_2_ (1.326 g, 11.14 mmol), and 4 drops of DMF was heated (5.5 h, 70–75 °C), the excess of SOCl_2_ was distilled off, the residue was dissolved in Et_2_O (6 mL) and added dropwise to a mixture of LiBH_4_ (0.268 g, 12.29 mmol) and Et_2_O (6 mL) cooled on ice. The obtained mixture was stirred at r.t. for 15 min, poured into 5% hydrochloric acid (10 mL), the organic layer combined with Et_2_O extract (10 mL) was washed with a 10% aqueous solution of K_2_CO_3_ (30 mL) and dried over MgSO_4_. Evaporation of the solvent and distillation in vacuum (120 °C, 6 Torr) gave 1.016 g of alcohol **14d** (yield 74%).

*b*. An analogous procedure for acid **42e** (2.00 g, 7.63 mmol), SOCl_2_ (1.817 g, 15.27 mmol), and LiBH_4_ (0.366 g, 16.79 mmol) afforded 1.600 g of alcohol **14e** (yield 85%). 

*c*. An analogous procedure for acid **42f** (1.414 g, 5.39 mmol), SOCl_2_ (1.284 g, 8.59 mmol), and LiBH_4_ (0.259 g, 11.88 mmol) afforded 0.975 g of alcohol **14f** (yield 73%).

*d*. An analogous procedure for acid **42g** (3.440 g, 9.50 mmol), SOCl_2_ (3.28 g, 27.56 mmol), and LiBH_4_ (0.470 g, 21.56 mmol) afforded 2.963 g of alcohol **14g** (yield 90%).

*e*. An analogous procedure for acid **42h** (3.450 g, 9.50 mmol), SOCl_2_ (4.92 g, 41.34 mmol), and LiBH_4_ (0.490 g, 22.48 mmol) with subsequent sublimation (110 °C, 1 Torr) of the product afforded 2.970 g of alcohol **14h** (yield 90%).

*(Perfluoro-4-methylphenyl)methanol (**14d**).* White solid; mp 41.5–42.5 °C (after distillation). IR (KBr) ν, cm^−1^: 3330 (OH), 1497 (FAR). ^1^H NMR (CDCl_3_): *δ* 4.85 (s, 2H, CH_2_), 2.04 (s, 1H, OH). ^19^F NMR (CDCl_3_): *δ* −57.6 (t, 3F, CF_3_ *J*_CF3,F(3)_ = *J*_CF3,F(5)_ = 22), −141.4 (m, 2F, F-3,5), −143.6 (m, 2F, F-2,6). HRMS, *m*/*z*: calcd. for C_8_H_3_O_1_F_7_ 248.0067; found 248.0064.

*(Perfluoro-3-methylphenyl)methanol (**14e**).* Colorless liquid. IR (film) ν, cm^−1^: 3338 (OH), 1506 (FAR). ^1^H NMR (CDCl_3_): *δ* 4.75 (s, 2H, CH_2_), 2.59 (s, 1H, OH). ^19^F NMR (CDCl_3_): *δ* −57.3 (t, 3F, CF_3_), −121.2 (qdddt, 1F, F-2), −130.9 (dddm, 1F, F-6), −133.5 (qddd, 1F, F-4), −163.0 (ddd, 1F, F-5); *J*_CF3,F(2)_ = *J*_CF3,F(4)_ = 22.5, *J*_2,4_ = 3, *J*_2,5_ = 11.5, *J*_2,6_ = 5, *J*_4,5_ = 21, *J*_4,6_ = 10.5, *J*_5,6_ = 21.5, *J*_CH2,F(2)_ = 2. HRMS, *m*/*z*: calcd. For [*M-H*]^+^ C_8_H_2_O_1_F_7_ 246.9986; found 246.9985.

*(Perfluoro-2-methylphenyl)methanol (**14f**).* Colorless liquid. IR (film) ν, cm^−1^: 3360 (OH), 1527, 1483 (FAR). ^1^H NMR (CDCl_3_): *δ* 4.80 (s, 2H, CH_2_), 2.49 (s, 1H, OH). ^19^F NMR (CDCl_3_): *δ* −55.2 (d, 3F, CF_3_), −138.3 (qddd, 1F, F-3), −140.9 (dddt, 1F, F-6), −149.0 (ddd, 1F, F-5), −153.4 (ddd, 1F, F-5); *J*_CF3,F(3)_ = 28.5, *J*_3,4_ = 20.5, *J*_3,5_ = 8, *J*_3,6_ = 12, *J*_4,5_ = 20, *J*_4,6_ = 5, *J*_5,6_ = 21.5, *J*_CH2,F(6)_ = 2. HRMS, *m*/*z*: calcd. for C_8_H_3_O_1_F_7_ 248.0067; found 248.0066.

*(2,3,5,6-Tetrafluoro-4-(perfluoroisopropyl)phenyl)methanol (**14g**).* Colorless liquid. IR (film) ν, cm^−1^: 3356 (OH), 1489 (FAR). ^1^H NMR (CDCl_3_): *δ* 4.86 (d, 2H, CH_2_, *J*_CH2,OH_ = 6), 2.18 (t, 1H, OH, *J*_CH2,OH_ = 6). ^19^F NMR (CDCl_3_): *δ* −76.4 (m, 6F, CF_3_), −135.3 (br.s, 1F, Ar-F), −138.3 (br.s, 1F, Ar-F), −142.6 (br.s, 1F, Ar-F), −143.7 (br.s, 1F, Ar-F), −179.8 (m, 1F, CF(CF_3_)_2_). HRMS, *m*/*z*: calcd. for C_10_H_3_OF_11_ 348.0003; found 347.9999.

*(Perfluoro-[1,1′-biphenyl]-4-yl)methanol (**14h**).* White solid; mp 73–74 °C (after sublimation). IR (KBr) ν, cm^−1^: 3292 (OH), 1535, 1508, 1479 (FAR). ^1^H NMR (CDCl_3_): *δ* 4.89 (s, 2H, CH_2_), 2.15 (s, 1H, OH). ^19^F NMR (CDCl_3_): *δ* −138.5 (m, 2F, Ar-F), −139.4 (m, 2F, Ar-F), −144.7 (m, 2F, Ar-F), −151.3 (tt, 1F, F-4′, *J*_4′,3′_ = *J*_4′,5′_ = 21, *J*_4′,2′_ = *J*_4′,6′_ = 3), −161.7 (m, 2F, F-3′,5′). HRMS, *m*/*z*: calcd. for C_13_H_3_OF_9_ 346.0035; found 346.0032.

#### 3.4.2. Synthesis of Chloroderivatives **8a**,**d**–**g**

*a*. A mixture of alcohol **14a** (2.950 g, 14.90 mmol) and SOCl_2_ (3.608 g, 30.32 mmol) was heated (2 h, 75 °C), the excess of SOCl_2_ was distilled off, distillation in vacuum (110 °C, 55 Torr) gave 2.940 g of compound **8a** (yield 91%).

*b*. A mixture of alcohol **14d** (1.761 g, 5.26 mmol) and SOCl_2_ (1.640 g, 15.16 mmol) was heated (1.5 h, 75 °C), the excess of SOCl_2_ was distilled off, and distillation in vacuum (110 °C, 20 Torr) gave 1.633 g of compound **8d** (yield 86%).

*c*. A mixture of alcohol **14e** (1.12 g, 4.52 mmol) and SOCl_2_ (1.148 g, 9.64 mmol) was heated (1.5 h, 75 °C), poured into ice water (10 mL), extracted with CH_2_Cl_2_ (2 × 2 mL), and dried over MgSO_4_. Evaporation of the solvent and distillation in vacuum (115 °C, 30 Torr) gave 1.139 g of compound **8e** (yield 94%).

*d*. A mixture of alcohol **14f** (2.488 g, 10.03 mmol) and SOCl_2_ (6.560 g, 55.13 mmol) was heated (4.5 h, 75 °C), poured into ice water (40 mL), extracted with CH_2_Cl_2_ (2 × 4 mL), and dried over MgSO_4_. Evaporation of the solvent and distillation in vacuum (115 °C, 42 Torr) gave 2.476 g of compound **8f** (yield 93%).

*e*. A mixture of alcohol **14g** (1.829 g, 7.10 mmol) and SOCl_2_ (1.804 g, 15.16 mmol) was heated (1.5 h, 75 °C), the excess of SOCl_2_ was distilled off, and distillation in vacuum (120 °C, 17 Torr) gave 1.798 g of compound **8g** (yield 93%).

*1-(Chloromethyl)-2,3,5,6-tetrafluoro-4-(trifluoromethyl)benzene (**8d**).* Colorless liquid. ^1^H NMR (CDCl_3_): *δ* 4.82 (s, CH_2_). ^19^F NMR (CDCl_3_): *δ* −57.5 (t, 3F, CF_3_ *J*_CF3,F(3)_ = *J*_CF3,F(5)_ = 22), −140.7 (m, 2F, F-3,5), −141.7 (m, 2F, F-2,6). HRMS, *m*/*z*: calcd. for C_8_H_2_ClF_7_ 265.9728; found 265.9724.

*1-(Chloromethyl)-2,4,5,6-tetrafluoro-3-(trifluoromethyl)benzene (**8e**).* Colorless liquid. ^1^H NMR (CDCl_3_): *δ* 4.62 (t, CH_2_, *J*_CH2,F(2)_ = *J*_CH2,F(6)_ = 1.5). ^19^F NMR (CDCl_3_): *δ* −57.3 (td, 3F, CF_3_), −119.5 (qdddt, 1F, F-2), −129.2 (dddtq, 1F, F-6), −131.9 (qddd, 1F, F-4), −162.2 (td, 1F, F-5); *J*_CF3,F(2)_ = *J*_CF3,F(4)_ = 22.5, *J*_CF3,F(6)_ = 1.5, *J*_2,4_ = 2.5, *J*_2,5_ = 11.5, *J*_2,6_ = 3.5, *J*_4,5_ = 21.5, *J*_4,6_ = 11.5, *J*_5,6_ = 21.5, *J*_CH2,F(2)_ = *J*_CH2,F(6)_ = 1.5. HRMS, *m*/*z*: calcd. for C_8_H_2_ClF_7_ 265.9728; found 265.9732.

*1-(Chloromethyl)-3,4,5,6-tetrafluoro-2-(trifluoromethyl)benzene (**8f**).* Colorless liquid. ^1^H NMR (CDCl_3_): *δ* 4.71 (d, CH_2_, *J*_CH2,F(2)_ = 2). ^19^F NMR (CDCl_3_): *δ* −55.6 (d, 3F, CF_3_), −137.6 (qddd, 1F, F-3), −138.7 (dddt, 1F, F-6), −148.5 (ddd, 1F, F-5), −152.0 (ddd, 1F, F-5); *J*_CF3,F(3)_ = 28, *J*_3,4_ = 21, *J*_3,5_ = 8, *J*_3,6_ = 11.5, *J*_4,5_ = 20.5, *J*_4,6_ = 5.5, *J*_5,6_ = 21.5, *J*_CH2,F(6)_ = 2. HRMS, *m*/*z*: calcd. for C_8_H_2_ClF_7_ 265.9728; found 265.9725.

*1-(Chloromethyl)-2,3,5,6-tetrafluoro-4-(perfluoroisopropyl)benzene (**8g**).* Colorless liquid. ^1^H NMR (CDCl_3_): *δ* 4.66 (s, CH_2_). ^19^F NMR (CDCl_3_): *δ* −76.3 (m, 6F, CF_3_), −134.7 (br.s, 1F, Ar-F), −137.7 (br.s, 1F, Ar-F), −141.8 (br.s, 1F, Ar-F), −142.0 (br.s, 1F, Ar-F), −179.7 (m, 1F, CF(CF_3_)_2_). HRMS, *m*/*z*: calcd. for C_10_H_2_ClF_11_ 365.9664; found 365.9662.

#### 3.4.3. Reaction of (2,3,4,5-Tetrafluoro-6-(hydroxymethyl)phenyl)methanol (**43**) with SOCl_2_

A mixture of diol **43** (0.262 g, 1.25 mmol) and SOCl_2_ (0.820 g, 6.89 mmol) was heated (1 h 40 min, 75 °C), the excess of SOCl_2_ was distilled off, the resulting mixture contained compounds **44** and **16c** in an 86:14 molar ratio. Sublimation (110 °C, 1 Torr) and recrystallization from hexane resulted in 0.189 g of compound **44** (yield 59%).

*1,5-Dihydrobenzo[e][1,3,2]dioxathiepine 3-oxide (**44**).* White solid; mp 76–77 °C (hexane). ^1^H NMR (CDCl_3_): *δ* 5.80 (d, 2H, CH_2_, *J*_A,B_ = 15), 4.97 (d, 2H, CH_2_, *J*_A,B_ = 15). ^19^F NMR (CDCl_3_): *δ* −143.0 (m, 2F, F-3,6), −155.5 (m, 2F, F-4,5). HRMS, *m*/*z*: calcd. for C_8_H_4_O_3_F_4_S 255.9812; found 255.9815. Anal. calcd. for C_8_H_4_O_3_F_4_S: C, 37.51; H, 1.57; F, 29.67%; S, 12.52%. Found: C, 38.19; H, 2.04; F, 29.88%; S, 12.44%.

#### 3.4.4. Synthesis of Chloroderivatives **8h**, **16c**, **19**, **23a**–**d**, and **36**

*a*. A mixture of alcohol **14h** (0.985 g, 2.85 mmol) and PCl_5_ (0.940 g, 4.51 mmol) was sealed in a glass tube (before the start of the reaction) and heated (3 h, 100 °C), poured into ice water (10 mL), extracted with CH_2_Cl_2_ (2 × 5 mL), and dried over MgSO_4_. Evaporation of the solvent and sublimation (100 °C, 1 Torr) gave 1.011 g of compound **8h** (yield 97%). 

*b*. A mixture of diol **43** (2.000 g, 9.52 mmol) and PCl_5_ (5.237 g, 25.12 mmol) was sealed in a glass tube (before the start of the reaction) and heated (3 h 40 min, 100 °C), poured into ice water (50 mL), extracted with CH_2_Cl_2_ (2 × 10 mL), and dried over MgSO_4_. Evaporation of the solvent and distillation in vacuum (130 °C, 15 Torr) gave 2.136 g of compound **16c** (yield 91%).

*c*. A mixture of alcohol **40** (4.06 g, 11.15 mmol) and PCl_5_ (2.95 g, 14.15 mmol) was sealed in a glass tube (before the start of the reaction) and heated (3.5 h, 100 °C), poured into ice water (40 mL), extracted with CH_2_Cl_2_ (2 × 5 mL), and dried over MgSO_4_. Evaporation of the solvent gave 4.18 g of compound **19** (yield 98%).

*d*. A mixture of alcohol **41a** (5.96 g, 22.4 mmol) and PCl_5_ (6.96 g, 33.4 mmol) was sealed in a glass tube (before the start of the reaction) and heated (5.5 h, 100 °C), poured into ice water (50 mL), extracted with CH_2_Cl_2_ (2 × 5 mL), and dried over MgSO_4_. The resulting mixture of products contained ~90% of compound **23a**. Evaporation of the solvent and distillation in vacuum (120 °C, 40 Torr) gave 5.40 g of compound **23a** (yield 85%).

*e*. A mixture of alcohol **41b** (4.14 g, 13.1 mmol) and PCl_5_ (4.11 g, 19.7 mmol) was sealed in a glass tube (before the start of the reaction) and heated (10 h, 100 °C), poured into ice water (50 mL), extracted with CH_2_Cl_2_ (2 × 5 mL), and dried over MgSO_4_. The resulting mixture of products contained ~90% of compounds **23b** and **45** in a 76:24 molar ratio. Evaporation of the solvent and distillation in vacuum (110 °C, 25–15 Torr) gave 3.83 g (yield 87%) of a mixture of compounds **23b** and **45** in a 79:21 molar ratio. This mixture was used in the carbonylation experiments (Section 3.3.24).

*f*. A mixture of alcohol **41c** (3.00 g, 9.15 mmol) and PCl_5_ (2.53 g, 12.13 mmol) was sealed in a glass tube (before the start of the reaction) and heated (5.5 h, 100 °C), poured into ice water (40 mL), extracted with CH_2_Cl_2_ (2 × 5 mL), and dried over MgSO_4_. The resulting mixture of products contained ~90% of compounds **23c** and **47** in a 91:9 molar ratio. Steam distillation and subsequent distillation in vacuum (135 °C, 22 Torr) gave 2.51 g (yield 79%) of a mixture of compounds **23c** and **47** in a 92:8 molar ratio. The content of a dichloroderivative did not exceed 1% (GC-MS). This mixture was used in the carbonylation experiments (Section 3.3.25).

*g*. A mixture of alcohol **41d** (6.46 g, 23.2 mmol) and PCl_5_ (7.33 g, 35.2 mmol) was sealed in a tube (before the start of reaction) and heated (6 h, 100 °C), poured into ice water (100 mL), extracted with CH_2_Cl_2_ (2 × 10 mL), and dried over MgSO_4_. The resulting mixture of products contained ~70% of compounds **23d**, along with a number of nonvolatile admixtures containing a tetrafluorosubstituted aromatic ring which were separated by steam distillation. Distillation in vacuum (120 °C, 45 Torr) gave 4.61 g (yield 67%) of compounds **23d**.

*h*. A mixture of alcohol **51** (2.5 g, 8.11 mmol) and PCl_5_ (4.30 g, 20.62 mmol) was sealed in a glass tube (before the start of the reaction) and heated (10 h, 100 °C), poured into ice water (40 mL), extracted with CH_2_Cl_2_ (2 × 5 mL), and dried over MgSO_4_. The resulting mixture of products contained ~70% of compounds **36**. Steam distillation and subsequent distillation in vacuum (150 °C, 15 Torr) gave 1.88 g (yield 67%) of a mixture containing (NMR ^19^F, GC-MS) ~90% of compounds **36** (isomers A:B = 64:36) along with isomeric compounds. The content of trichloroderivatives did not exceed 3% (GC-MS). This mixture was used in the carbonylation experiments (Section 3.3.28).

*4-(Chloromethyl)nonafluoro-1,1′-biphenyl (**8h**).* White solid; mp 65.5–66 °C (after sublimation).^1^H NMR (CDCl_3_): *δ* 4.71 (s, CH_2_). ^19^F NMR (CDCl_3_): *δ* −138.3 (m, 2F, Ar-F), −138.7 (m, 2F, Ar-F), −142.9 (m, 2F, Ar-F), −150.9 (tt, 1F, F-4′, *J*_4′,3′_ = *J*_4′,5′_ = 21, *J*_4′,2′_ = *J*_4′,6′_ = 3), −161.5 (m, 2F, F-3′,5′). HRMS, *m*/*z*: calcd. for C_13_H_2_ClF_9_ 363.9696; found 363.9694.

*1,2-Bis(chloromethyl)-3,4,5,6-tetrafluorobenzene (**16c**).* Colorless liquid. ^1^H NMR (CDCl_3_): *δ* 4.73 (s, CH_2_).^19^F NMR (CDCl_3_): *δ* −141.8 (m, 2F, F-3,6), −154.2 (m, 2F, F-4,5). HRMS, *m*/*z*: calcd. for C_8_H_4_Cl_2_F_4_ 245.9621; found 245.9620.

*(1-Chloro-2,2,2-trifluoroethyl)pentafluorobenzene (**23a**).* Colorless liquid. ^1^H NMR (CDCl_3_): *δ* 5.50 (qt, 1H, *J*_H,CF3_ = 7, *J*_H,F(*ortho*)_ = 1, CHCl). ^19^F NMR (CDCl_3_): *δ* −73.8 (td, 3F, *J*_CF3,F(*ortho*)_ = 10, *J*_H,CF3_ = 7, CF_3_), ~−138.4 (br.s, 2F, F-*ortho*), −150.1 (tt, 1F, *J_para,meta_* = 21, *J_para,ortho_* = 4, F-*para*), −160.9 (br.s, 2F, F-*meta*).

*(1-Chloro-2,2,3,3,3-pentafluoropropyl)pentafluorobenzene (**23b**)* in the mixture with compound 45 (23b:45 = 79:21). Colorless liquid. ^1^H NMR (CDCl_3_): δ 5.58 (ddt, 1H, J_H,F(B)_ = 13.5, J_H,F(A)_ = 12.5, J_H,F(ortho)_ = 1.5, CHCl). ^19^F NMR (CDCl_3_): δ −82.6 (s, 3F, CF_3_), −118.5 (ddd, 1F, J_A,B_ = 267, J_A,ortho_ = 31.5, J_H,F(A)_ = 12.5, CF_2_-F_A_), −119.4 (ddd, 1F, J_A,B_ = 267, J_B,ortho_ = 28.5, J_H,F(B)_ = 13.5, CF_2_-F_B_), −133.6 (br.s, 1F, F-ortho), −141.9 (br.s, 1F, F-ortho), −149.7 (tt, 1F, J_para,meta_ = 21, J_para,ortho_ = 4, F-para), −160.3 (br.s, 1F, F-meta), −161.3 (br.s, 1F, F-meta). HRMS, *m*/*z*: calcd. for C_9_H_1_Cl_1_F_10_ 333.9602; found 333.9607.

*1-(1-Chloro-2,2,3,3,3-pentafluoropropyl)-4-chloro-2,3,5,6-tetrafluorobenzene (**45**)* in the mixture with compound **23b** (**23b**:**45** = 79:21). ^1^H NMR (CDCl_3_): δ 5.60 (ddt, 1H, J_H,F(B)_ = 13.5, J_H,F(A)_ = 12.5, J_H,F(2)_ = J_H,F(6)_ = 1.5, CHCl). ^19^F NMR (CDCl_3_): δ −82.6 (s, 3F, CF_3_), −118.4 (ddd, 1F, J_A,B_ = 267, J_A,ortho_ = 31.5, J_H,F(A)_ = 12.5, CF_2_-F_A_), −119.3 (ddd, 1F, J_A,B_ = 267, J_B,ortho_ = 28.5, J_H,F(B)_ = 13.5, CF_2_-F_B_), −133.6 (br.s, 1F, F-2,6), −139.3 (br.s, 1F, F-3,5), −140.3 (br.s, 1F, F-3,5), −141.7 (br.s, 1F, F-2,6). HRMS, *m*/*z*: calcd. for C_9_H_1_Cl_2_F_9_ 349.9306; found 349.9309.

*1-Chloro-2,2,3,3,4,4,5,6,7,8-decafluorotetralin (**23c**)* in the mixture with compound **47** (**23c**:**47** = 92:8). Colorless liquid. ^1^H NMR (CDCl_3_): δ 5.52 (dt, 1H, J = 10.5, J = 4, H-1). ^19^F NMR (CDCl_3_): −97.9 (dm, 1F, J_A,B_ = 291, F_A_-4), −114.5 (dm, 1F, J_A,B_ = 291, F_B_-4), −115.4 (dm, 1F, J_A,B_ = 273, F_A_-2), −123.7 (dm, 1F, J_A,B_ = 273, F_A_-3), −125.4 (dm, 1F, J_A,B_ = 273, F_B_-2), −136.2 (dddd, 1F, F-8), −136.6 (ddddd, 1F, F-5), −143.1 (dm, 1F, J_A,B_ = 273, F_B_-3), −146.2 (dddd, 1F, F-7), −148.6 (ddd, 1F, F-6); J_5,A4_ = 8.5, J_5,B4_ = 29.5, J_5,6_ = 20.5, J_5,7_ = 8, J_5,8_ = 12, J_6,7_ = 20.5, J_6,8_ = 7, J_7,8_ = 20.5, J_7,A4_ = 3, J_8,B4_ = 2.5. HRMS, *m*/*z*: calcd. for C_10_H_1_Cl_10_F_9_ 345.9602; found 345.9600.

*7-Chloro-1,1,2,2,3,3,5,6,7,8-decafluoro-1,2,3,7-tetrahydronaphthalene (**47**)* in the mixture with compound **23c** (**23c**:**47** = 92:8). ^1^H NMR (CDCl_3_): δ 6.54 (m, 1H, H-1). ^19^F NMR (CDCl_3_): −107.5 (ddm, 1F, J_7,8_ = 28.5, J_6,7_ = 26.5, F-7), −110.2 (br.d, 1F, J_A,B_ ~ 300, F_A_-1 or 3), −111.8 (br.d, 1F, J_A,B_ ~ 300, F_B_-1 or 3), −111.8 (td, 1F, J_1,8_ = 29, J_7,8_ = 28.5, F-8), −114.4 (br.d, 1F, J_A,B_ ~ 290, F_A_-1 or 3), −116.8 (br.d, 1F, J_A,B_ ~ 290, F_B_-1 or 3), −137.7 (br.d, 1F, J_A,B_ ~ 270, F_A_-2), −139.9 (br.d, 1F, J_A,B_ ~ 270, F_B_-2), −145.4 (dm, 1F, J_6,7_ = 26.5, F-6), −150.1 (m, 1F, F-5).

*1,4-Dichloro-2,2,3,3,5,6,7,8-octafluorotetralin (**36**)* (isomers A:B = 64:36). Colorless liquid. Isomer A. ^1^H NMR (CDCl_3_): δ 5.53 (m, 2H, H-1,4). ^19^F NMR (CDCl_3_): −109.6 (dm, 2F, J ~ 280, CF_2_), −125.8 (dm, 2F, J ~ 280, CF_2_), −137.3 (m, 2F, F-5,8), −150.6 (m, 2F, F-6,7). Isomer B. ^1^H NMR (CDCl_3_): δ 5.45 (m, 2H, H-1,4). ^19^F NMR (CDCl_3_): −118.0 (dm, 2F, J ~ 260, CF_2_), −119.1 (dm, 2F, J ~ 260, CF_2_), −135.3 (m, 2F, F-5,8), −150.7 (m, 2F, F-6,7). HRMS, *m*/*z*: calcd. for C_10_H_1_Cl_2_F_8_ 343.9400; found 343.9403.

#### 3.4.5. Synthesis of Fluoroderivatives **7a**,**d**, and **18**

*a*. A mixture of compound **8a** (4.35 g, 20.1 mmol) and CsF (9.00 g, 59.2 mmol) was sealed in a glass tube and heated (10 h, 250–260 °C). Distillation of volatile product gave 3.44 g of compound **7a** (yield 86%).

*b*. A mixture of compound **8d** (0.740 g, 2.78 mmol) and CsF (2.220 g, 14.6 mmol) was sealed in a glass tube and heated (9 h, 250–260 °C). Distillation of volatile product gave 0.560 g of compound **7d** (yield 81%).

*c*. A mixture of compound **19** (2.75 g, 7.2 mmol) and CsF (6.39 g, 42.0 mmol) was sealed in a glass tube and heated (17 h, 250–260 °C). Steam distillation gave 1.706 g of compound **18** (yield 65%).

#### 3.4.6. Synthesis of (1,2,2,3,3,3-Hexafluoropropyl)pentafluorobenzene (**22b**)

Alcohol **41b** (3.664 g, 11.59 mmol) was added to a mixture of C_6_F_5_CF_3_ (3.275 g, 13.88 mmol) with SbF_5_ (2.516 g, 11.62 mmol), the resulting mixture was intensively stirred (4 h, 70 °C), poured into of 5% hydrochloric acid (25 mL), extracted with CH_2_Cl_2_ (2 × 10 mL), and dried over MgSO_4_. The extract contained compounds **22b** and pentafluorobenzoate of the starting alcohol (GC-MS, *M* = 510) in a 90:10 molar ratio, along with C_6_F_5_CF_3_ and C_6_F_5_CO_2_H. It was washed with a saturated aqueous solution of NaHCO_3_ (60 mL), the solvent was evaporated, and fractional distillation of the residue in a vacuum (100 Torr) gave a fraction (1.828 g, bp 90 °C) containing 98% of compound **22b** (yield 50%).

*(1,2,2,3,3,3-Hexafluoropropyl)pentafluorobenzene (**22b**).* Colorless liquid. ^1^H NMR (CDCl_3_): *δ* 6.11 (ddd, 1H, *J*_H,F(1)_ = 43, *J*_H,F(B)_ = 19, *J*_H,F(A)_ = 4, CHF). ^19^F NMR (CDCl_3_): *δ* −83.6 (d, 3F, CF_3_), −124.4 (ddtd, 1F, CF_2_-F_A_), −131.1 (ddtd, 1F, CF_2_-F_B_), −139.8 (m, 2F, F-*ortho*), −148.8 (ttd, 1F, F-*para*), −160.8 (br.s, 2F, F-*meta*), −206.0 (dddtqd, 1F, F-1); *J*_F(1),H_ = 43, *J*_1,A_ = 14.5, *J*_1,B_ = 15.5, *J*_1,3_ = 10, *J*_1,*ortho*_ = 13, *J*_1,*para*_ = 2, *J*_A,B_ = 282.5, *J*_F(A),H_ = 4, *J*_A,*ortho*_ = 6.5, *J*_F(B),H_ = 19, *J*_B,*ortho*_ = 17, *J*_H,F(A)_ = 12.5, *J*_A,B_ = 267, *J*_H,F(B)_ = 13.5, *J_para_*_,*meta*_ = 21, *J_para_*_,*ortho*_ = 4. HRMS, *m*/*z*: calcd. for C_9_H_1_F_11_ 317.9897; found 317.9893.

#### 3.4.7. Synthesis of Acids **11**–**13**

*a*. A mixture of acid **9e** (0.524 g, 1.90 mmol), CF_3_CO_2_H (0.330 g, 2.89 mmol), and SbF_5_ (1.690 g, 7.81 mmol) (molar ratio 1:1.5:4.1) was stirred at room temperature for 4.5 h, r.t., poured into 5% hydrochloric acid (20 mL), and extracted with Et_2_O. The resulting solution contained compounds **9e** and **11** in an 18:82 molar ratio. After evaporation of Et_2_O, the residue was dissolved in a saturated aqueous solution of NaHCO_3_ (50 mL), acidified with HCl (pH < 1), and extracted with CH_2_Cl_2_ (2 × 5 mL) to separate the acid **9e**. The aqueous layer was extracted with Et_2_O (3 × 5 mL). Evaporation of the solvent, sublimation (190 °C, 1 Torr), and recrystallization from CCl_4_-acetone resulted in 0.337 g of acid **11** (yield 70%).

*b*. Acid **9d** (0.559 g, 2.02 mmol), CF_3_CO_2_H (0.391 g, 3.42 mmol), and SbF_5_ (1.956 g, 9.03 mmol) (molar ratio 1:1.7:4.5), similarly to previous procedure, gave a mixture of compounds **9d** and **12** in a 15:85 molar ratio. Evaporation of the solvent fractional sublimation (130 °C, 1 Torr), then (210 °C, 1 Torr) and recrystallization of the second fraction from CCl_4_-acetone gave 0.322 g of acid **12** (yield 63%).

*c*. In a manner analogous to [79], 4-methylheptafluorotoluene (0.565 g, 2.44 mmol) was dissolved in SbF_5_ (3.175 g, 14.67 mmol), the resulting solution was poured into of 5% hydrochloric acid (30 mL), extracted with CH_2_Cl_2_ (2 × 10 mL), and dried over MgSO_4_. The extract contained compound **13** and the starting compound in a 75:25 molar ratio. Evaporation of the solvent and sublimation (130 °C, 2 Torr) resulted in 0.312 g of acid **13** (yield 61%).

*3-Carboxymethyl-2,4,5,6-tetrafluorobenzoic acid (**11**).* White solid; mp 176.5–178 °C (CCl_4_-acetone). IR (KBr) ν, cm^−1^: 1705, 1743 (C=O), 1502, 1490, 1431, 1416 (FAR). ^1^H NMR (acetone-*d_6_*): *δ* 5.9 (br.s, 2H, COOH), 3.81 (s, 2H, CH_2_). ^19^F NMR (acetone-*d_6_*): *δ* −117.3 (dddt, 1F, F-2), −131.4 (dddt, 1F, F-4), −134.5 (dd, 1F, F-6), −164.9 (td, 1F, F-5); *J*_2,4_ = 4, *J*_2,5_ = 11, *J*_2,6_ = 1.5, *J*_4,5_ = 21, *J*_4,6_ = 8, *J*_5,6_ = 21, *J*_CH2,F(2)_ = *J*_CF2,F(4)_ = 1.5. HRMS, *m*/*z*: calcd. for C_9_H_4_O_4_F_4_ 252.0040; found 252.0038. Anal. calcd. for C_9_H_4_O_4_F_4_: C, 42.88; H, 1.60; F, 30.14%. Found: C, 43.3; H, 2.10; F, 30.26%.

*4-Carboxymethyl-2,3,5,6-tetrafluorobenzoic acid (**12**).* White solid; mp 216.5–218 °C (after sublimation). IR (KBr) ν, cm^−1^: 1714 (C=O), 1495 (FAR). ^1^H NMR (acetone-*d_6_*): *δ* 5.9 (br.s, 2H, COOH), 3.91 (s, 2H, CH_2_). ^19^F NMR (acetone-*d_6_*): *δ* −141.2 (m, 2F), −141.9 (m, 2F). HRMS, *m*/*z*: calcd. for C_9_H_4_O_4_F_4_ 252.0040; found 252.0043. Anal. calcd. for C_9_H_4_O_4_F_4_: C, 42.88; H, 1.60; F, 30.14%. Found: C, 42.76; H, 1.35; F, 30.06%.

*2,3,5,6-Tetrafluoro-4-methylbenzoic acid (**13**).* White solid; mp 166–168 °C (after sublimation). IR (KBr) ν, cm^−1^: 1730 (C=O), 1535, 1508, 1500, 1481 (FAR). ^1^H NMR (acetone-*d_6_*): *δ* 4.7 (br.s, 1H, COOH), 2.33 (t, 3H, CH_3_, *J*_CH3,F(3)_ = *J*_CF3,F(5)_ = 2). ^19^F NMR (acetone-*d_6_*): *δ* −141.6 (m, 2F, F-2,6), −142.7 (m, 2F, F-3,5).

## 4. Conclusions

The carbonylation at the benzyl C-Hal (Hal = F, Cl) bond of polyfluorinated alkyl aromatic compounds with different types of their aliphatic moiety in the CO–SbF_5_ system, resulting in corresponding carboxylic acids, or their methyl esters was demonstrated. Polyfluorinated alkylaromatic compounds Ar_F_CHHalR (R = Hal, H, Alk_F_, C_6_F_5_) containing a hydrogen atom at the benzyl position undergo carbonylation in the CO–SbF_5_ system; for CCl_2_H derivatives, the possibility of selective mono- and dicarbonylation depending on the amount of SbF_5_ was demonstrated, whereas for CF_2_H derivatives, only dicarbonylation is possible. The carbonylation of *para*-substituted benzyl halides XC_6_F_4_CH_2_Hal (X = Alk_F_, C_6_F_5_, CH_2_Cl), in contrast to *meta*- and *ortho*-isomers (X = CF_3_, CH_2_Cl), gives corresponding acids in low yields, due to side transformations of the starting compounds in an SbF_5_ medium. The addition of CO to compounds Ar_F_CHHalAlk_F_ can be accompanied by the elimination of HF, thus leading to saturated or *α*,*β*-unsaturated *α*-arylcarboxylic acids (or esters), with the outcome depending on the reaction conditions.

## Figures and Tables

**Table 1:** Carbonylation of compounds **1a**–**c** and **2a**,**d** in the CO–SbF_5_ system.

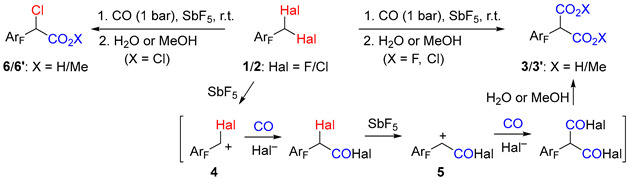						
Entry	Substrate	Ar_F_	Hal	SbF_5_ (Equiv)	Time (h)	Product/Content {Yield} ^a^ (%)
1	**1a**	C_6_F_5_	F	1	2.5	**3a**/64 ^b^
2	″	″	F	2	2.5	**3′a**/92
3	″	″	F	5.3	6	**3′a**/96 {90}
4	**1b**	Perfluoroindan-5-yl	F	5.9	3.25	**3′b**/85 {76}
5	**1c**	Perfluorotetralin-6-yl	F	1	0.75	**3′c**/30
6	″	″	F	6	3.25	**3′c**/90 {81}
7	**2a**	C_6_F_5_	Cl	0.5	3	**6a**/41
8	″	″	Cl	1	2.5	**6a**/89 {69}
9	″	″	Cl	1	3	**6′a**/86 {47}
10	″	″	Cl	2	3	**6′a**/76
11	″	″	Cl	4	3	**3′a**/35
12	″	″	Cl	6	4.5	**3′a**/25
13	**2d**	*p*-CF_3_C_6_F_4_	Cl	1	1.75	**6d**/70 {65}
14	″	″	Cl	5.8	3.25	**3′d**/80 {64}

^a^ Isolated yield. ^b^ The mixture also contained 6% of its decarboxylation product **9a**.

**Table 2:** Carbonylation of benzyl halides **7a**,**d** and **8a**,**d**–**h** in the CO–SbF_5_ system and alcohols **14 d**–**f** in superacids.

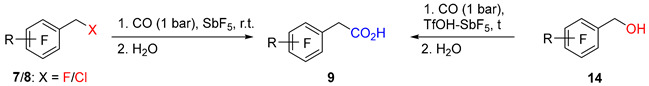								
Entry	Substrate	R	X	SbF_5_ (Equiv)	TfOH (Equiv)	Time (h)	t (°C)	Product/Content {Yield} ^a^ (%)
1	**7a**	F	F	0.5	-	2	r.t.	**9a**/15
2	″	″	F	2	-	0.75	r.t.	**9a**/10
3	″	″	F	3.8	-	0.75	r.t.	**9a**/75
4	″	″	F	6	-	0.75	r.t.	**9a**/90 {86}
5	**8a**	F	Cl	0.5	-	2	r.t.	**9a**/10
6	″	″	Cl	6	-	0.75	r.t.	**9a**/90 {87}
7	**7d**	*p*-CF_3_	F	6.1	-	1	r.t.	**9d**/6
8	**8d**	*p*-CF_3_	Cl	5.9	-	2.5	r.t.	**9d**/6
9	**8e**	*m*-CF_3_	Cl	6	-	0.75	r.t.	**9e**/90 {85}
10	**8f**	*o*-C_6_F_4_	Cl	5.9	-	0.75	r.t.	**9f**/92 {88}
11	**8g**	*p*-CF(CF_3_)_2_	Cl	6.1	-	1.5	r.t.	**9g**/20 {11}
12	**8h**	*p*-C_6_F_5_	Cl	6	-	2	r.t.	**9h**/5 {3}
13	**14d**	*p*-CF_3_	OH	3	3	7	60	**9d**/88 {65}
14	**14e**	*m*-CF_3_	OH	-	7	6	50	**9e**/85 {64}
15	**14f**	*o*-CF_3_	OH	-	7	6	75	**9f**/95 {75}

^a^ Isolated yield.

**Table 3:** Carbonylation of compounds **16a**–**c** in the CO–SbF_5_ system.

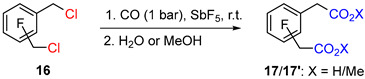					
Entry	Substrate	Substitution Pattern	SbF_5_ (Equiv)	Time (h)	Product/Content {Yield} ^a^ (%)
1	**16a**	*para*	6.4	2	**17′a**/15
2	**16b**	*meta*	6.5	2	**17′b**/65 {44}
3	**16c**	*ortho*	6	1	**17′c**/86
4	″	″	6.9	2.5	**17c**/80 {67}

^a^ Isolated yield.

**Table 4:** Carbonylation of compounds **18** and **19** in the CO–SbF_5_ system.

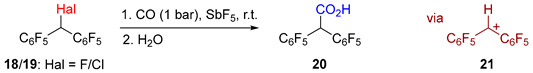					
Entry	Substrate	Hal	SbF_5_ (Equiv)	Time (h)	Content {Yield} ^a^ of Acid 20 (%)
1	**18**	F	0.5	6	45
2	″	F	1	4.5	50 {44}
3	″	F	2.1	4.5	4
4	″	F	6	5.5	3
5	**19**	Cl	0.5	4.5	88 {85}
6	″	Cl	1	4.5	45
7	″	Cl	6	4.5	<1

^a^ Isolated yield.

**Table 5:** Carbonylation of compounds **22b**,**d** and **23a**–**d** in the CO–SbF_5_ system.

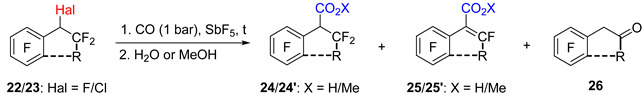									
Entry	Substrate	R	Hal	SbF_5_ (eq.)	Time (h)	t (°C)	Product/Content {Yield} ^a^ (%)		
							24/24′	25/25′	26
1	**23a**	F	Cl	0.5	1	r.t.	**24a**/71	-	-
2	″	″	Cl	1	1	r.t.	**24a**/>95 {92}	-	-
3	″	″	Cl	1	1.5	r.t.	**24′a**/>95 {90}	-	-
4	″	″	Cl	6	0.5	r.t.	**24a**/>95 {96}	-	-
5	**22b**	CF_3_	F	1	0.5	r.t.	**24b**/86	**25b**/9	**26b**/5
6	″	″	F	2	0.5	r.t.	**24b**/90	**25b**/9	**26b**/1
7	″	″	F	4	0.5	r.t.	**24b**/95	**25b**/5	-
8	″	″	F	6	0.5	r.t.	**24b**/95 {81}	**25b**/5	-
9	″	″	F	6	6	70	-	**25′b**/72 ^b^ {69}	-
10	**23b**	CF_3_	Cl	1	1	r.t.	**24b**/89	**25b**/5	**26b**/6
11	″	″	Cl	6	1	r.t.	**24b**/95	**25b**/5	-
12	″	″	Cl	6	5	r.t.	**24b**/91	**25b**/9	-
13	″	″	Cl	6	1	70	**24b**/36	**25b**/60	**26b**/4
14	″	″	Cl	6	5	70	**24b**/2	**25b**/92	**26b**/6
15	**23c**	–CF_2_CF_2_–	Cl	1	1	r.t.	**24c**/81	**25c**/7	**26c**/12
16	″	″	Cl	6	1	r.t.	**24c**/87 {59}	**25c**/13	
17	″	″	Cl	6	1	70	-	**25c**/94 {68}	**26c**/6
18	**22d**	–CF_2_–	F	1	0.5	r.t.	**24d**/95 {79}	-	-
19	**23d**	–CF_2_–	Cl	1	1	r.t.	**24d**/50	-	-
20	″	″	Cl	6	0.5	r.t.	**24d**/50	-	-

^a^ Isolated yield. ^b^ The mixture also contained 18% of acid **25b**.

**Table 6:** Synthesis of starting compounds **7a**,**d**, **8a**,**d**–**h**, **18**, **19**, and **23a**.

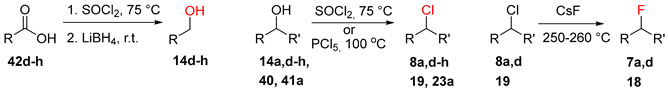					
Entry	R	R′	Alcohol, Yield ^a^ (%)	Chloride, Yield ^a^ (%)	Fluoride, Yield ^a^ (%)
1	C_6_F_5_	H	-	**8a**, 91 ^b^	**7a**, 86
2	*p*-C_6_F_4_CF_3_	H	**14d**, 74	**8d**, 86 ^b^	**7d**, 81
3	*m*-C_6_F_4_CF_3_	H	**14e**, 85	**8e**, 94 ^b^	-
4	*o*-C_6_F_4_CF_3_	H	**14f**, 73	**8f**, 93 ^b^	-
5	*p*-C_6_F_4_CF(CF_3_)_2_	H	**14g**, 90	**8g**, 93 ^b^	-
6	*p*-C_6_F_4_C_6_F_5_	H	**14h**, 90	**8h**, 97 ^c^	-
7	C_6_F_5_	C_6_F_5_	-	**19**, 98 ^c^	**18**, 65
8	C_6_F_5_	CF_3_	-	**23a**, 85 ^c^	-

^a^ Isolated yield. ^b^ SOCl_2_ as the reagent. ^c^ PCl_5_ as the reagent.

## Data Availability

All raw data of this study are available within this article.

## Supplementary Materials

The following supporting information can be downloaded at: https://www.mdpi.com/article/10.3390/molecules30040931/s1, Figures S1–S80: ^1^H and ^19^F NMR spectra of the products.
