# Supplementary material for: Carbonylation of Polyfluorinated Alkylbenzenes and Benzocycloalkenes at the Benzyl C-F and C-Cl Bonds Under the Action of CO/SbF5
Source: Molecules. 2025 Feb 17;30(4):931. doi: 10.3390/molecules30040931 (PMC11858302; doi:10.3390/molecules30040931)
Supplement: Supplementary file 1 [file molecules-30-00931-s001.zip › molecules-3457537-supplementary.pdf]

Supplementary information

Carbonylation of Polyfluorinated Alkylbenzenes and Benzocycloalkenes at the Benzyl C-F and C-Cl Bonds under the Action of CO/SbF_5

Yaroslav V. Zonov [1,2,*], Siqi Wang [3], Vladislav V. Komarov [1], Victor M. Karpov [1], Dmitriy A. Parkhomenko [1] and Tatyana V. Mezhenkova[1]

[1] N. N. Vorozhtsov Novosibirsk Institute of Organic Chemistry SB RAS, Lavrentiev Ave. 9, Novosibirsk 630090, Russia
[2] Department of Natural Sciences, Novosibirsk State University, Pirogov Str. 1, Novosibirsk 630090, Russia
[3] Daqing Petrochemical Research Center, PetroChina Company Limited, Daqing 163714, China

* Correspondence: yzonov@nioch.nsc.ru

^{1}H and ^{19}F NMR spectra of products

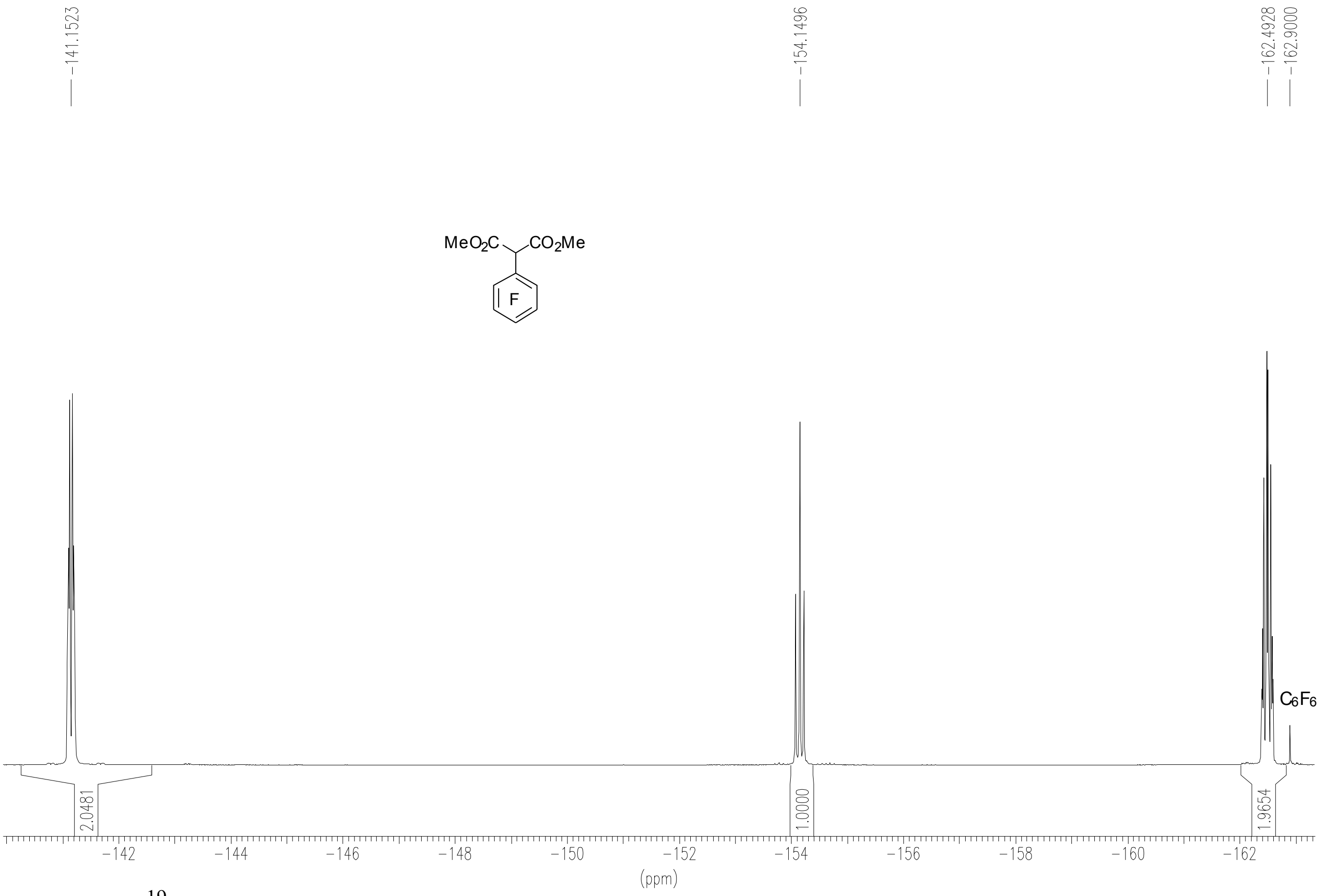

Figure S1. ^{19}F NMR spectrum of **3’a** ($CDCl_3$).

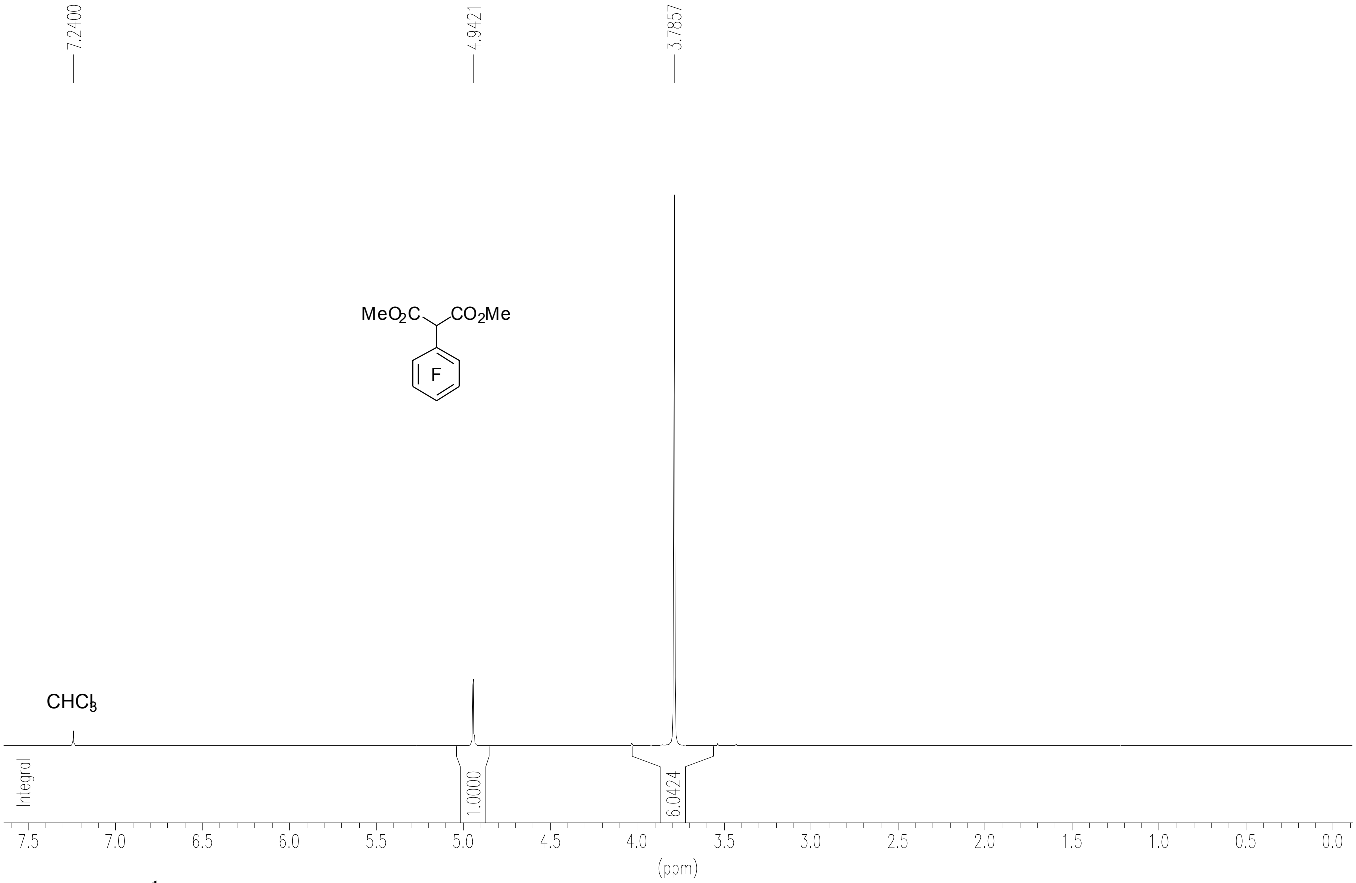

Figure S2. ^{1}H NMR spectrum of **3’a** ($CDCl_3$).

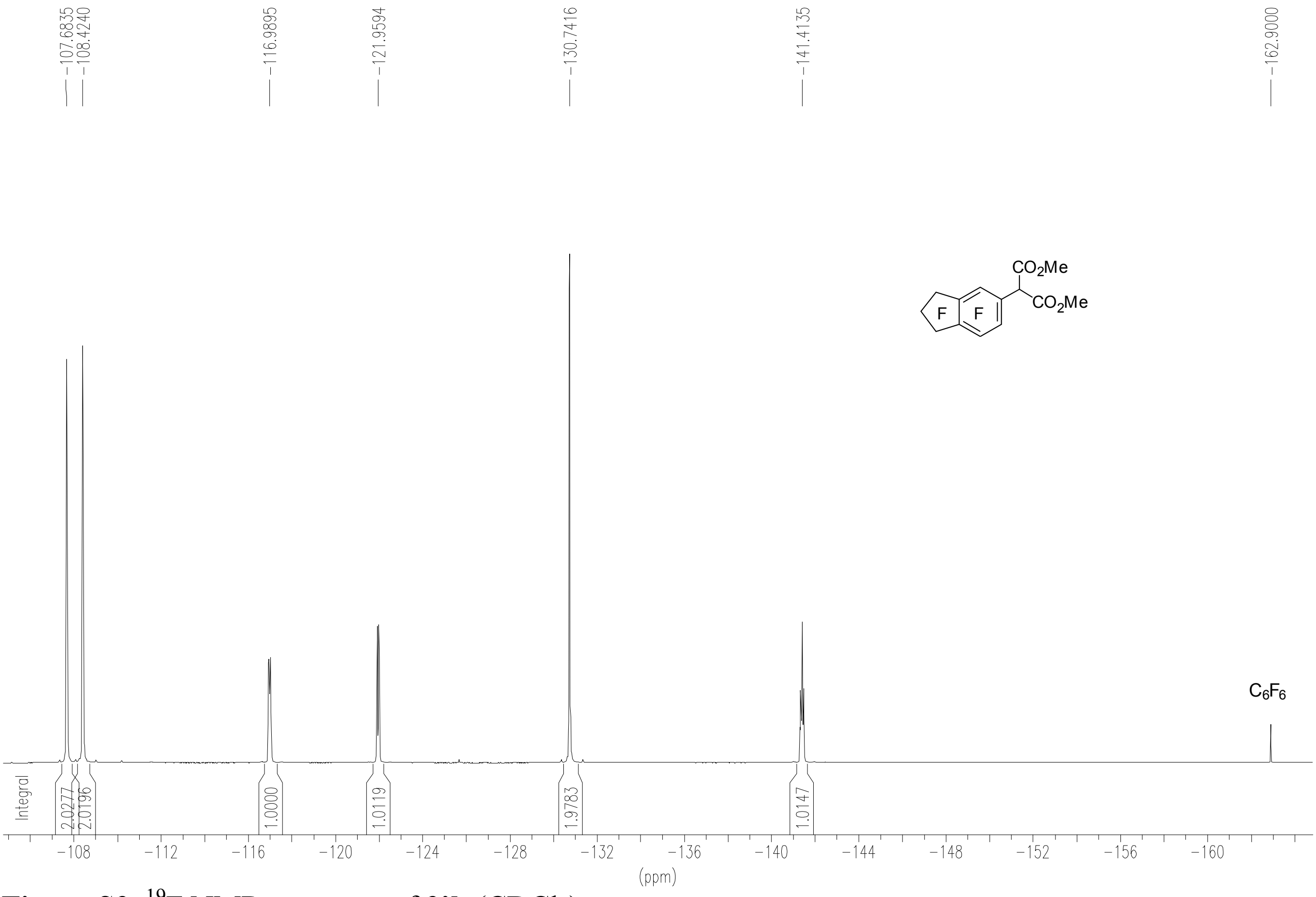

Figure S3. ^{19}F NMR spectrum of **3’b** ($CDCl_3$).

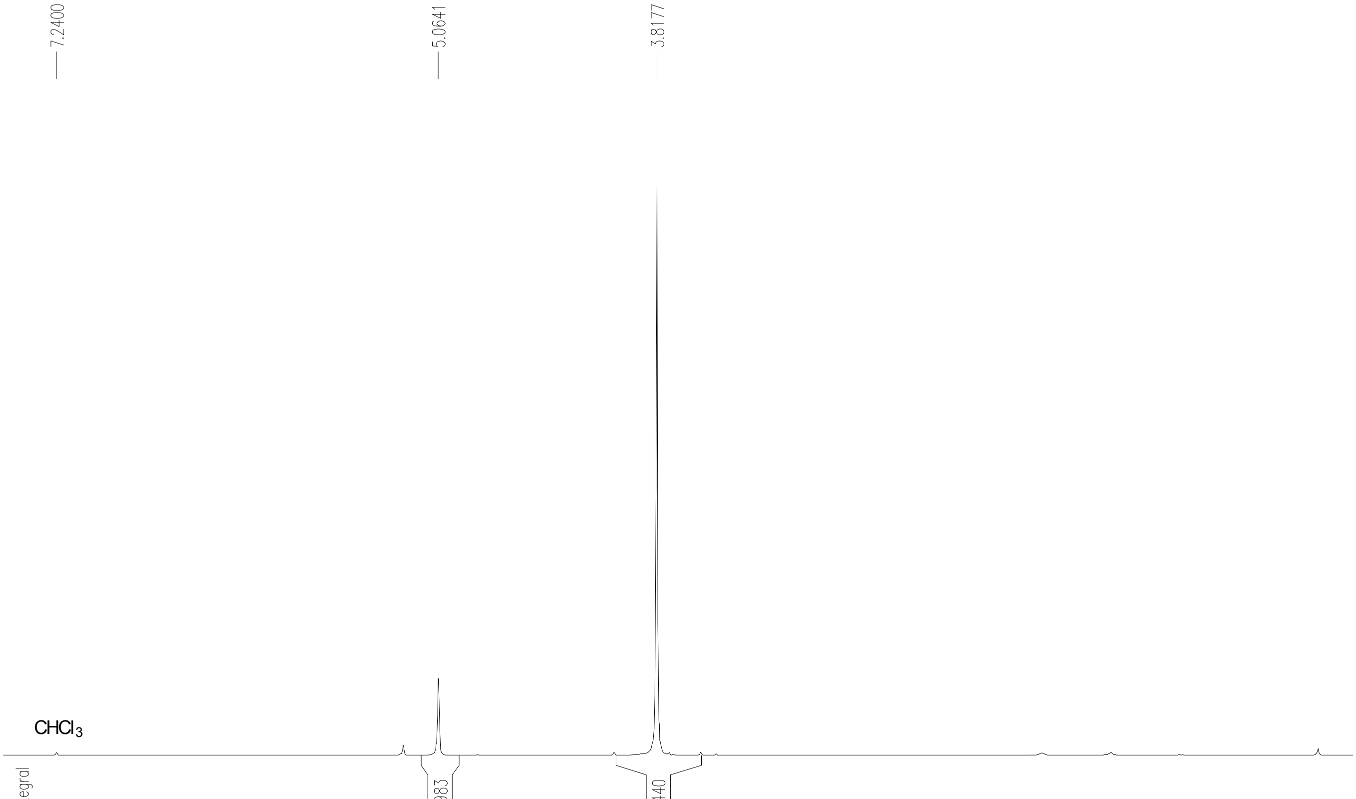

Figure S4. ^1H NMR spectrum of **3’b** ($CDCl_3$).

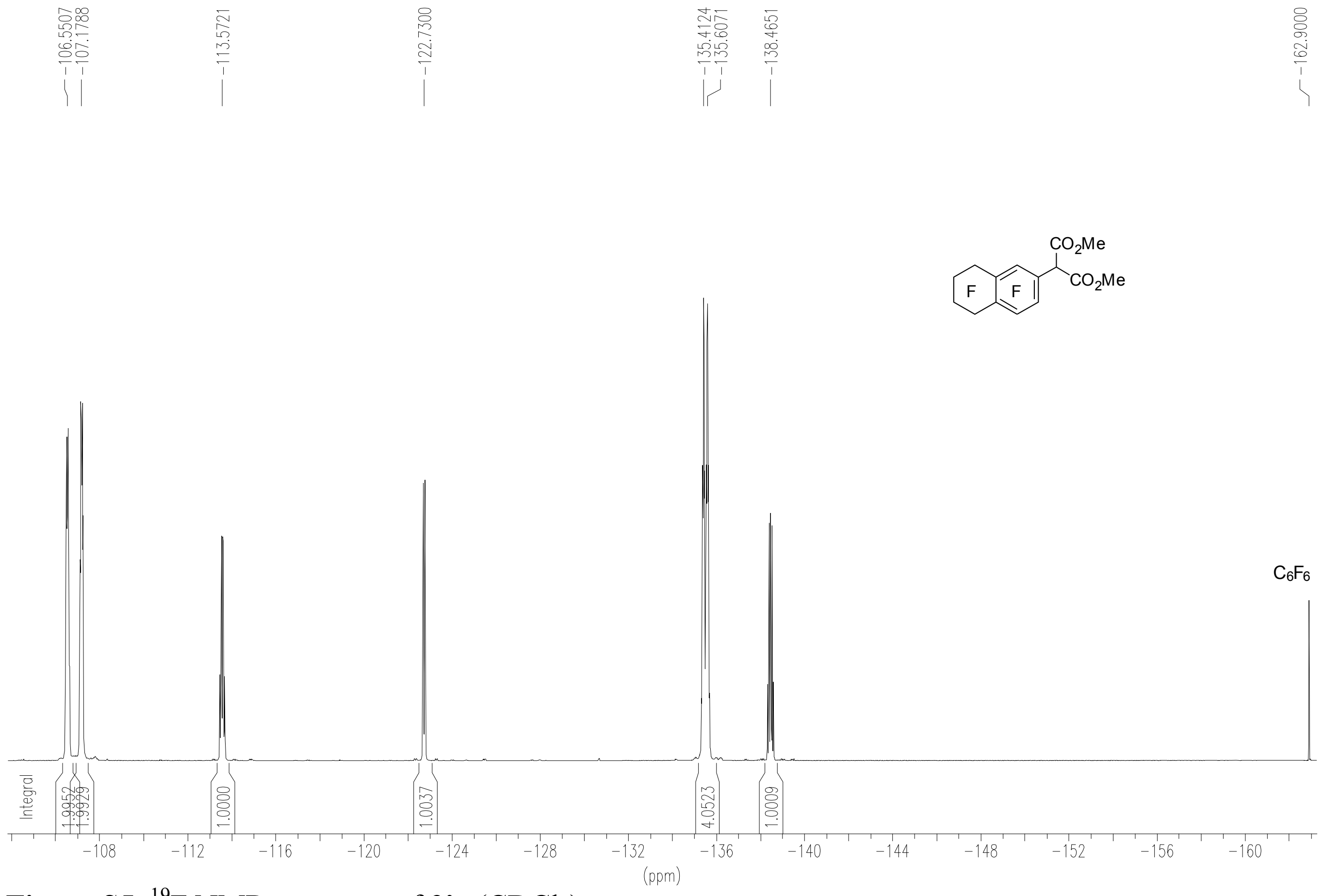

Figure S5. ^{19}F NMR spectrum of **3'c** ($CDCl_3$).

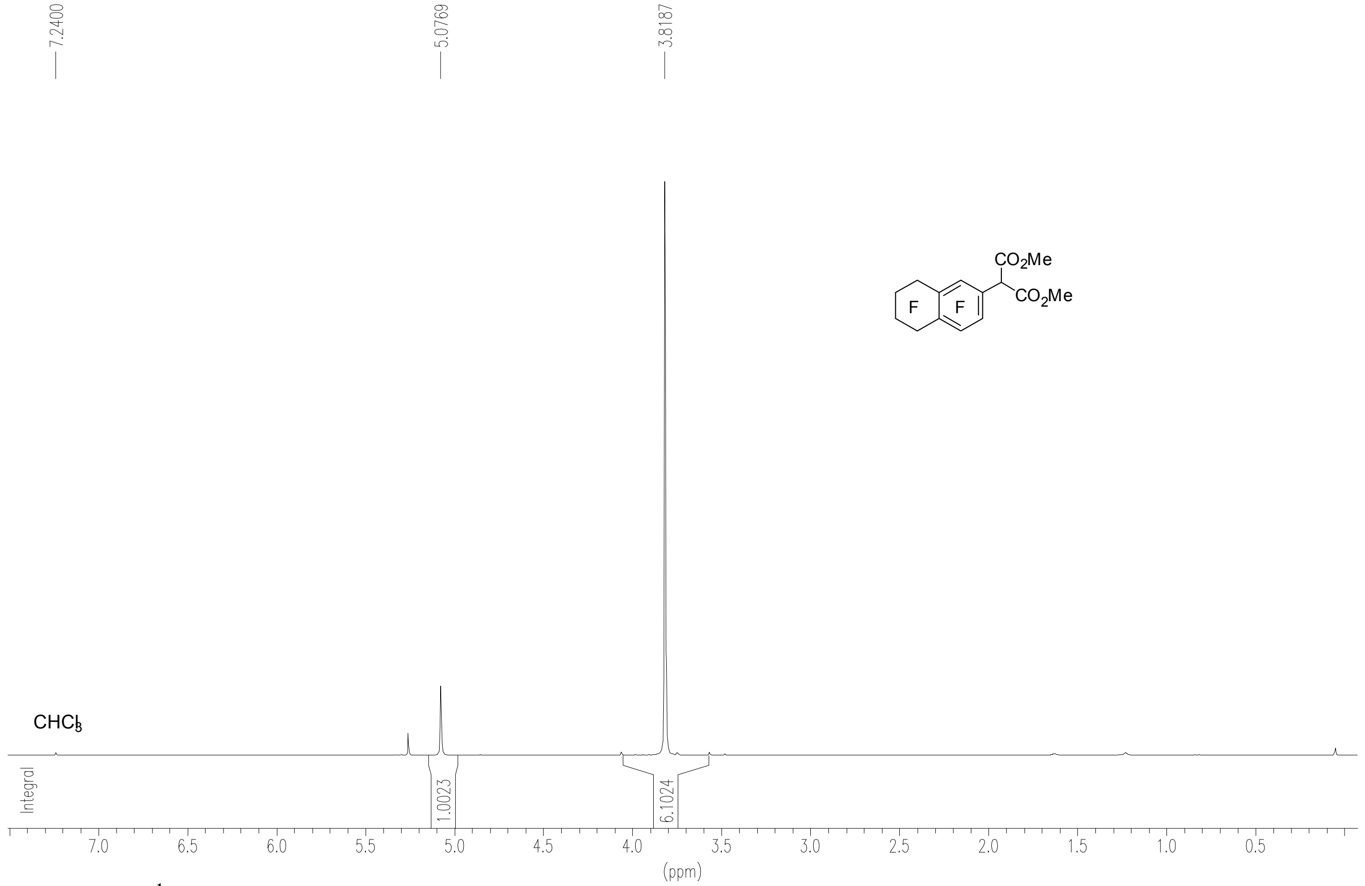

Figure S6. ^{1}H NMR spectrum of **3'c** ($CDCl_3$).

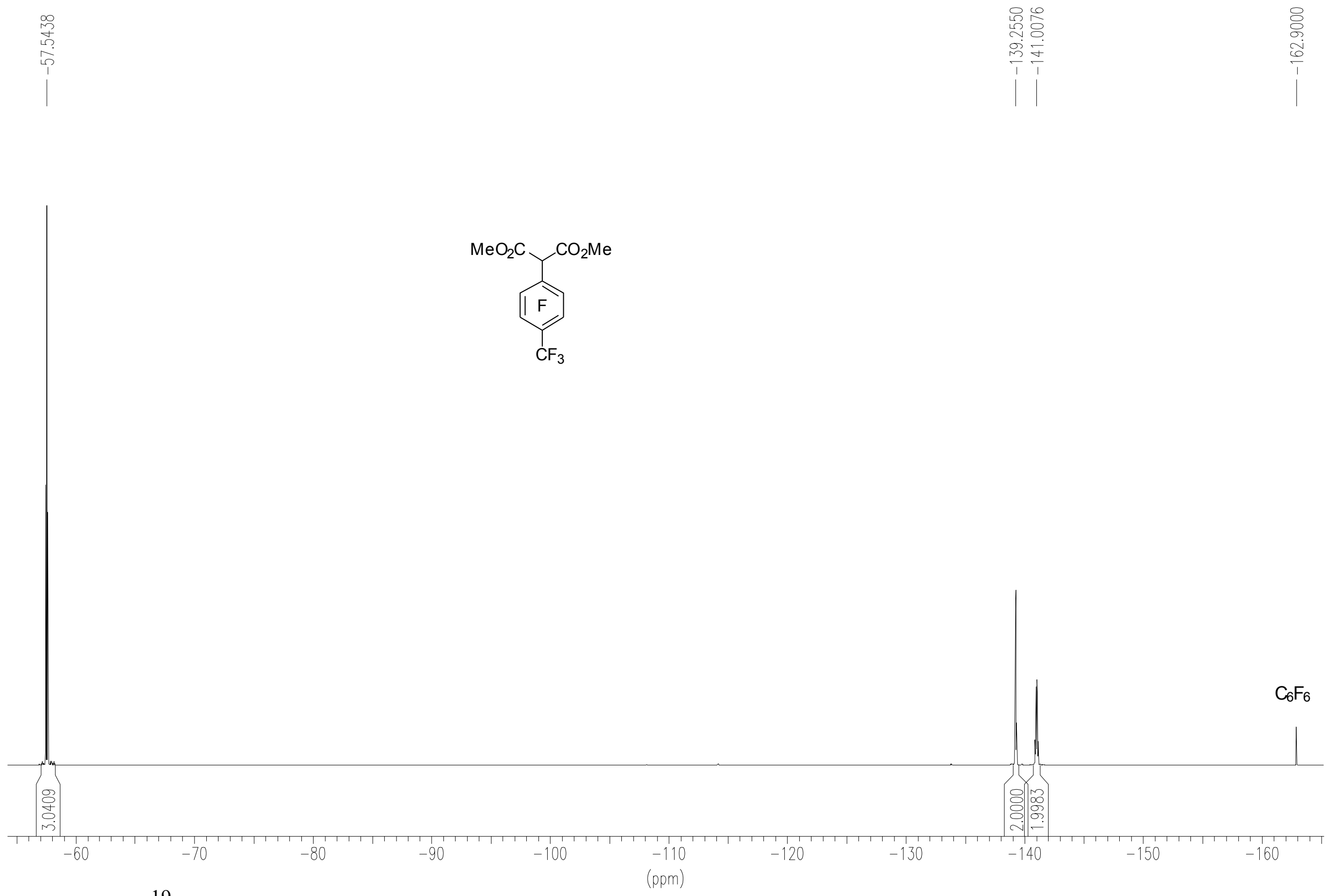

Figure S7. ^{19}F NMR spectrum of **3’d** ($CDCl_3$).

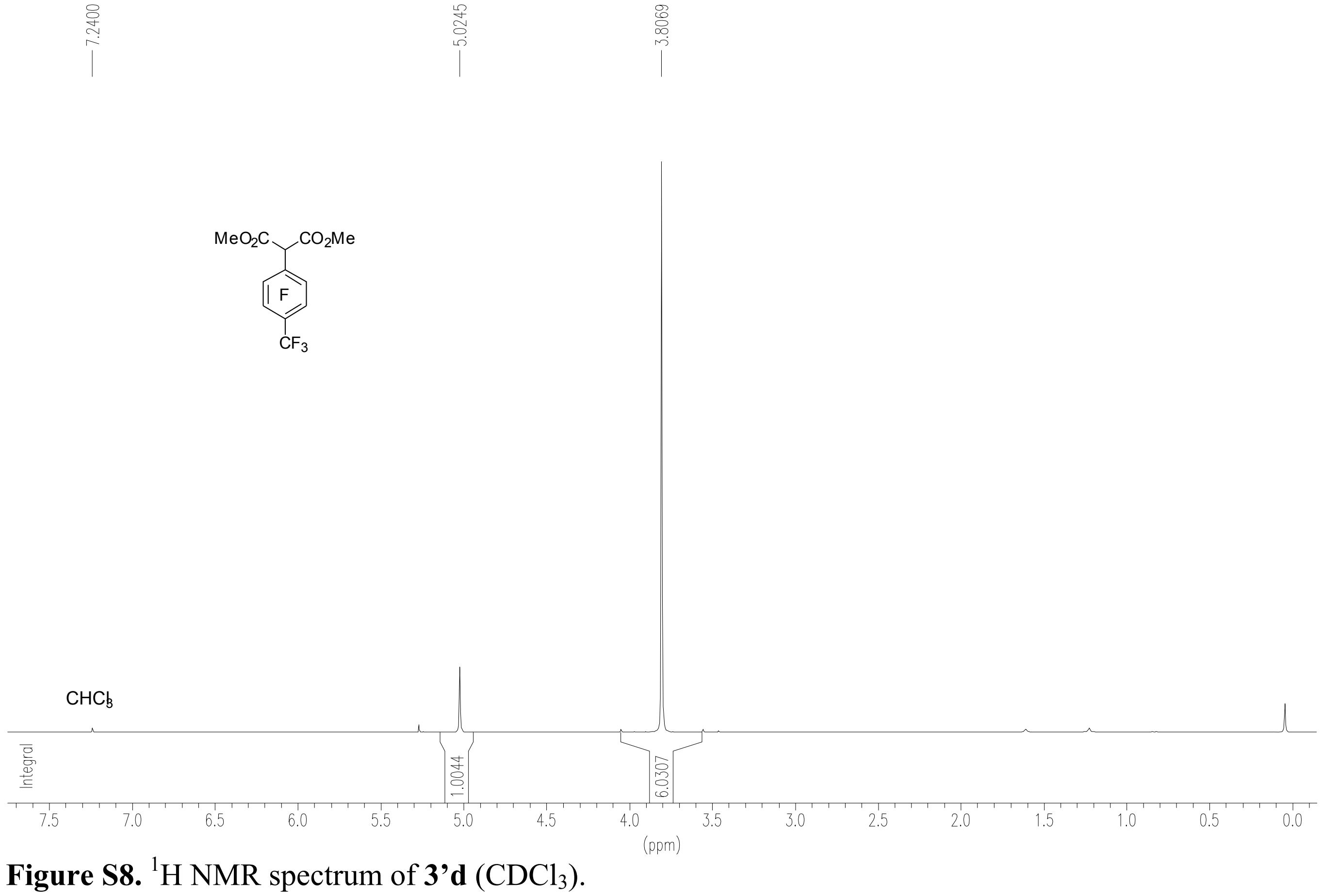

Figure S8. ^{1}H NMR spectrum of **3’d** ($CDCl_3$).

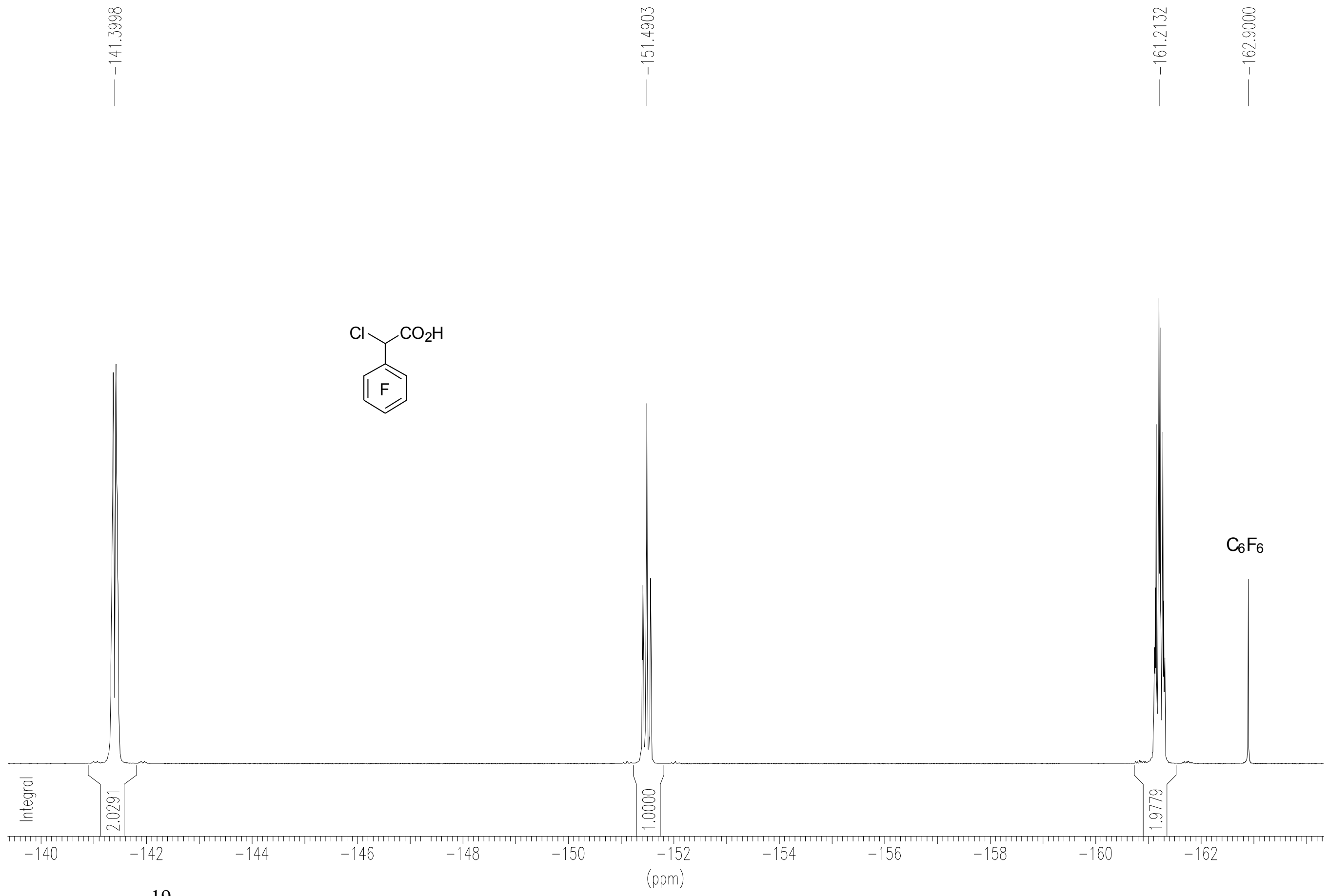

Figure S9. ^{19}F NMR spectrum of **6a** ($CDCl_3$).

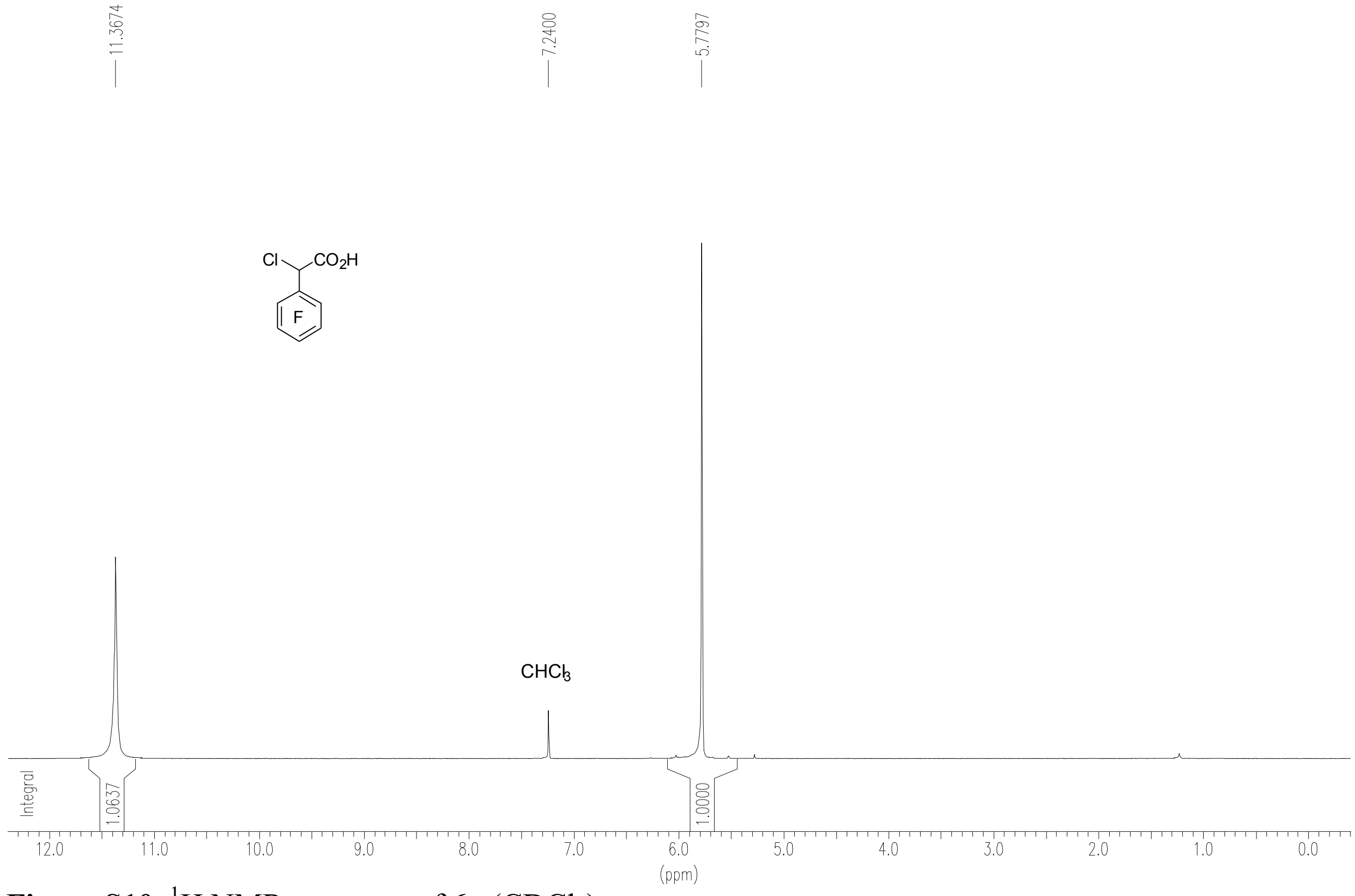

Figure S10. ^{1}H NMR spectrum of **6a** ($CDCl_3$).

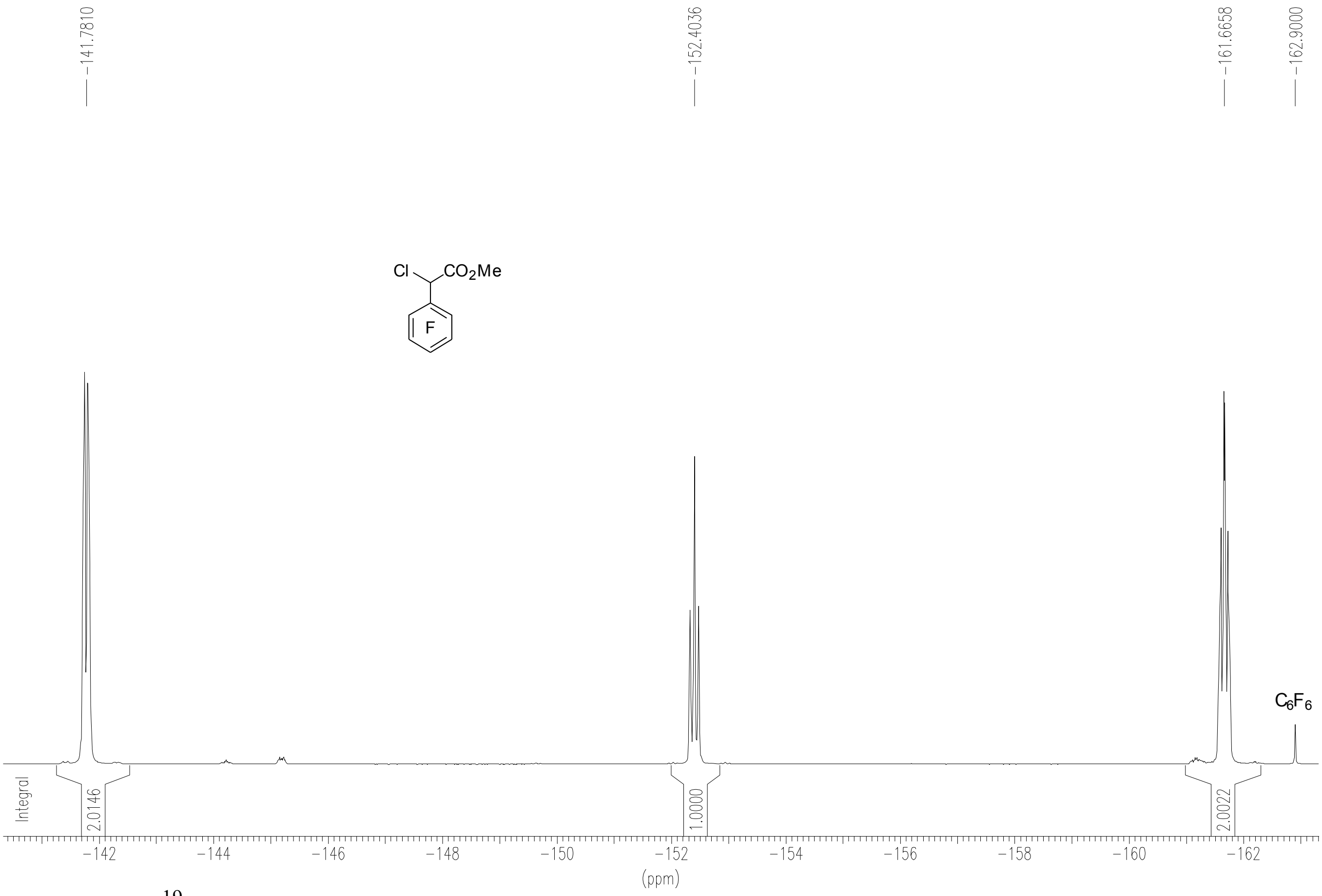

Figure S11. ^{19}F NMR spectrum of **6'a** ($CDCl_3$).

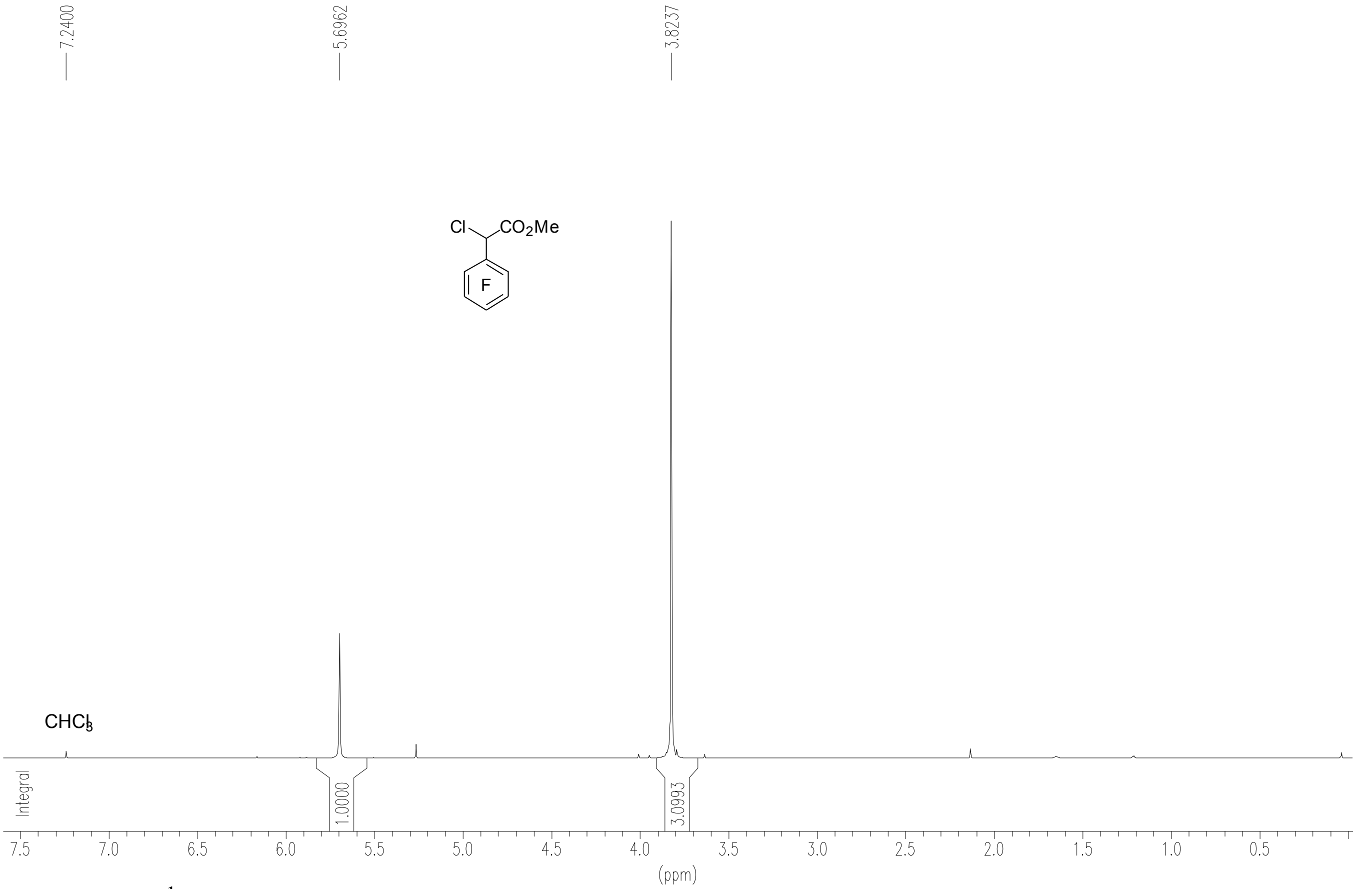

Figure S12. ^{1}H NMR spectrum of **6'a** ($CDCl_3$).

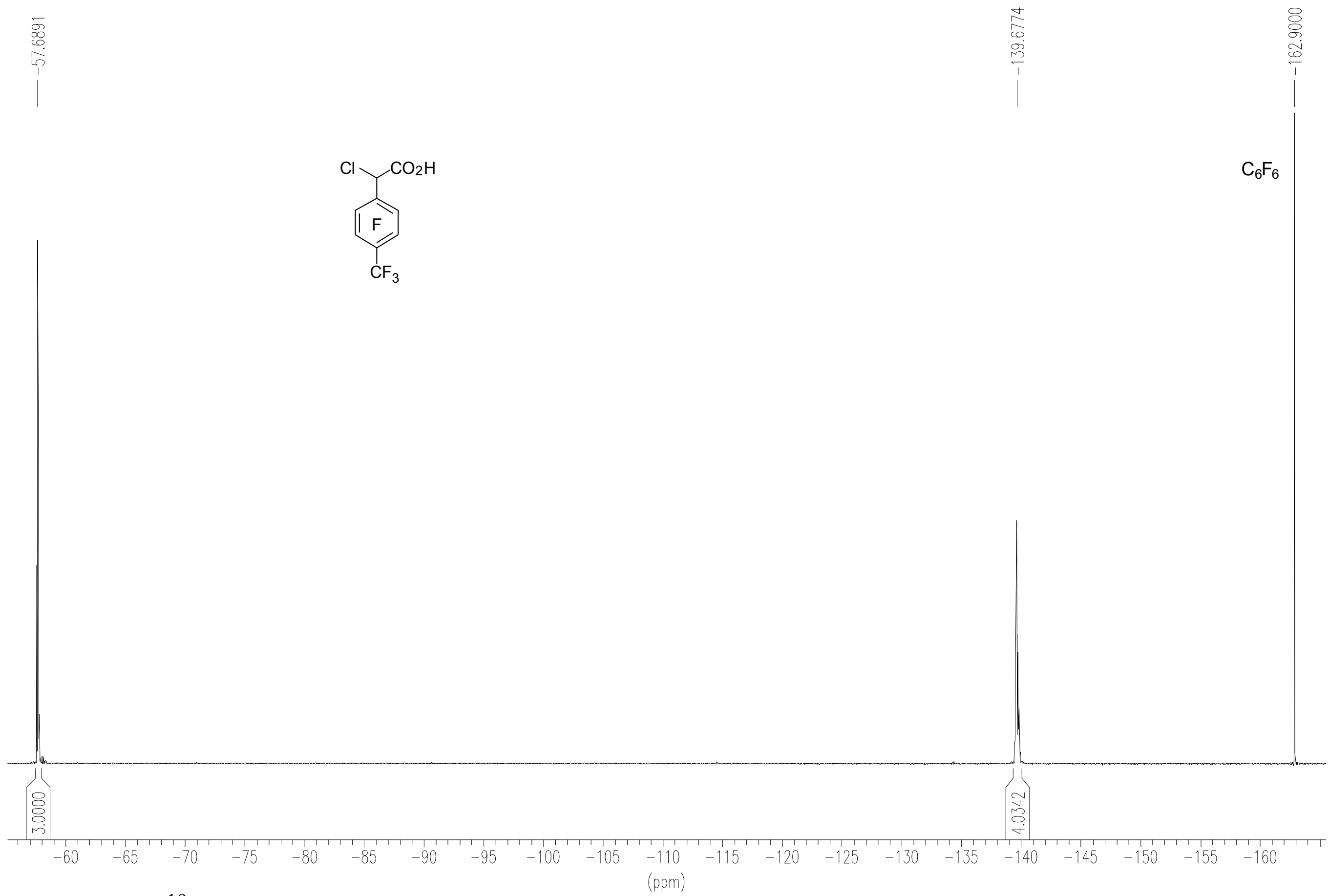

Figure S13. ^{19}F NMR spectrum of **6d** ($CDCl_3$).

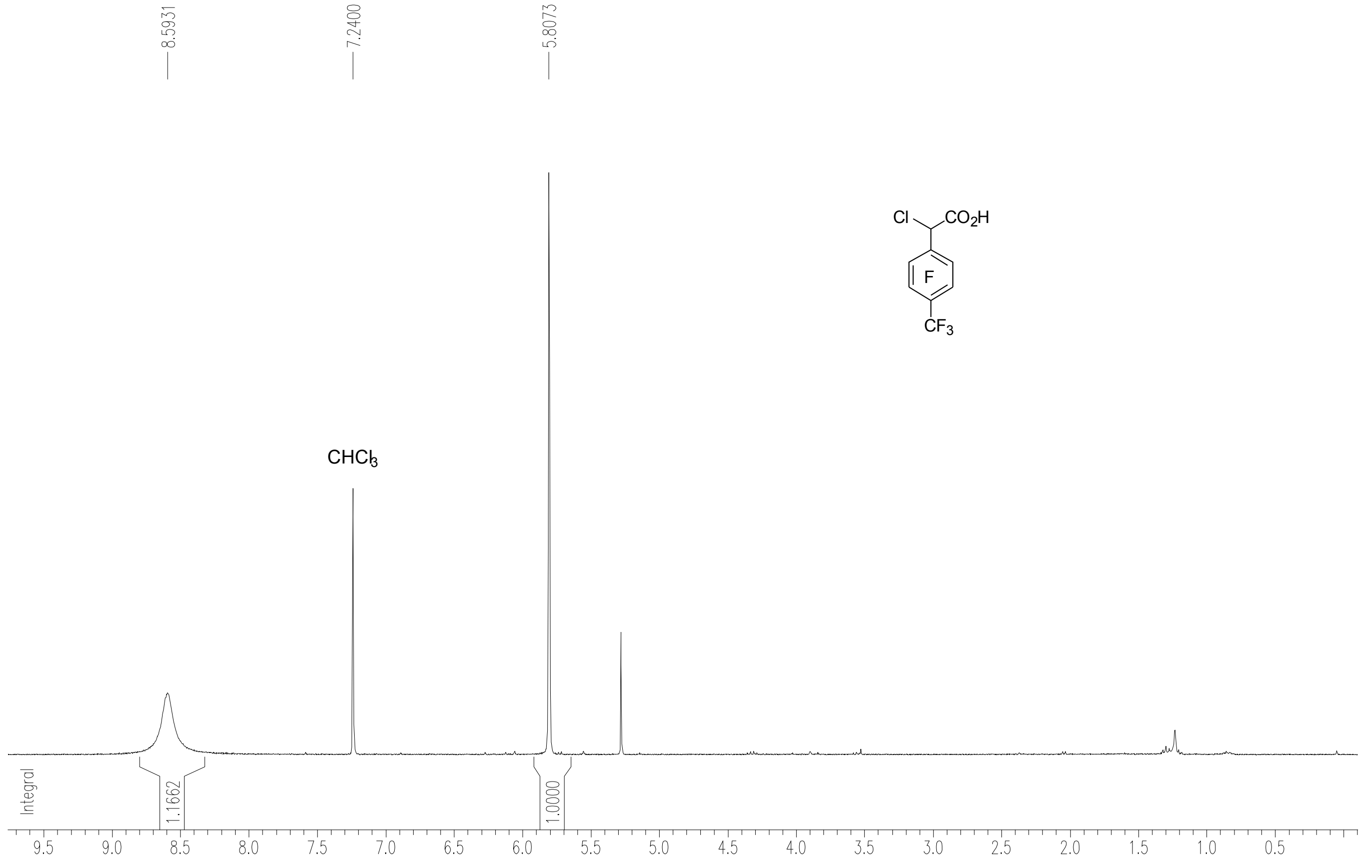

Figure S14. ^{1}H NMR spectrum of **6d** ($CDCl_3$).

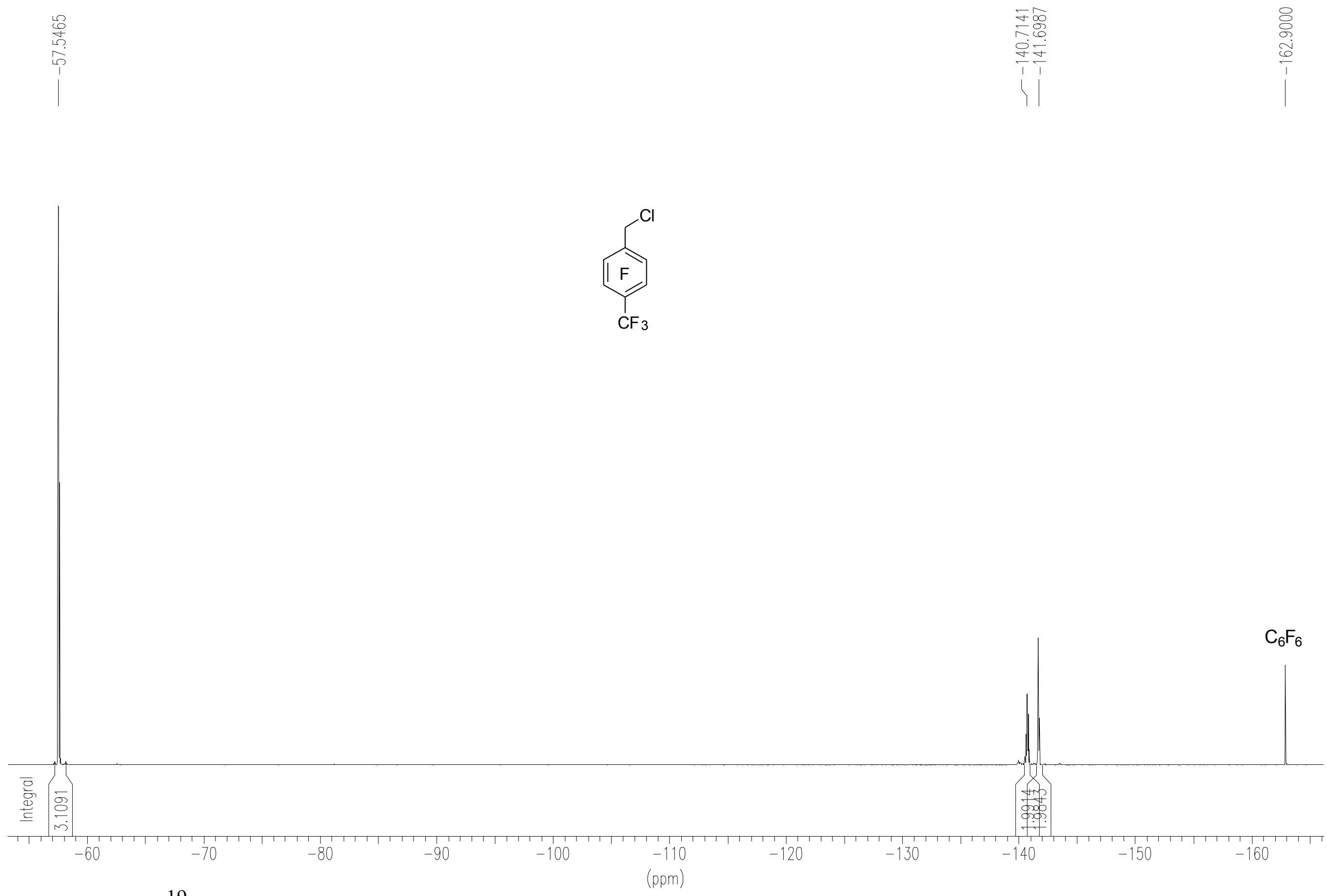

Figure S15. ^{19}F NMR spectrum of **8d** ($CDCl_3$).

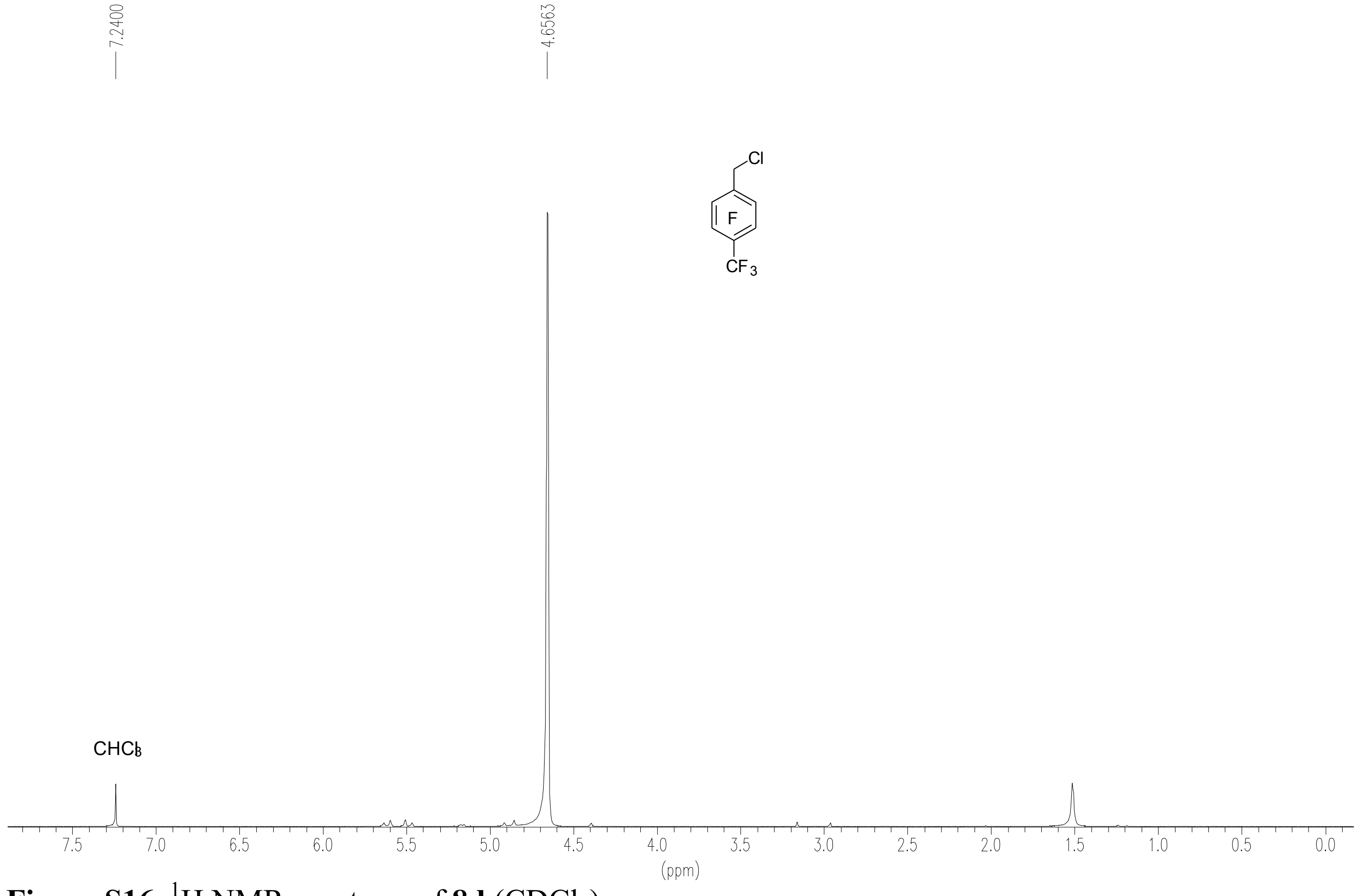

Figure S16. ^{1}H NMR spectrum of **8d** ($CDCl_3$).

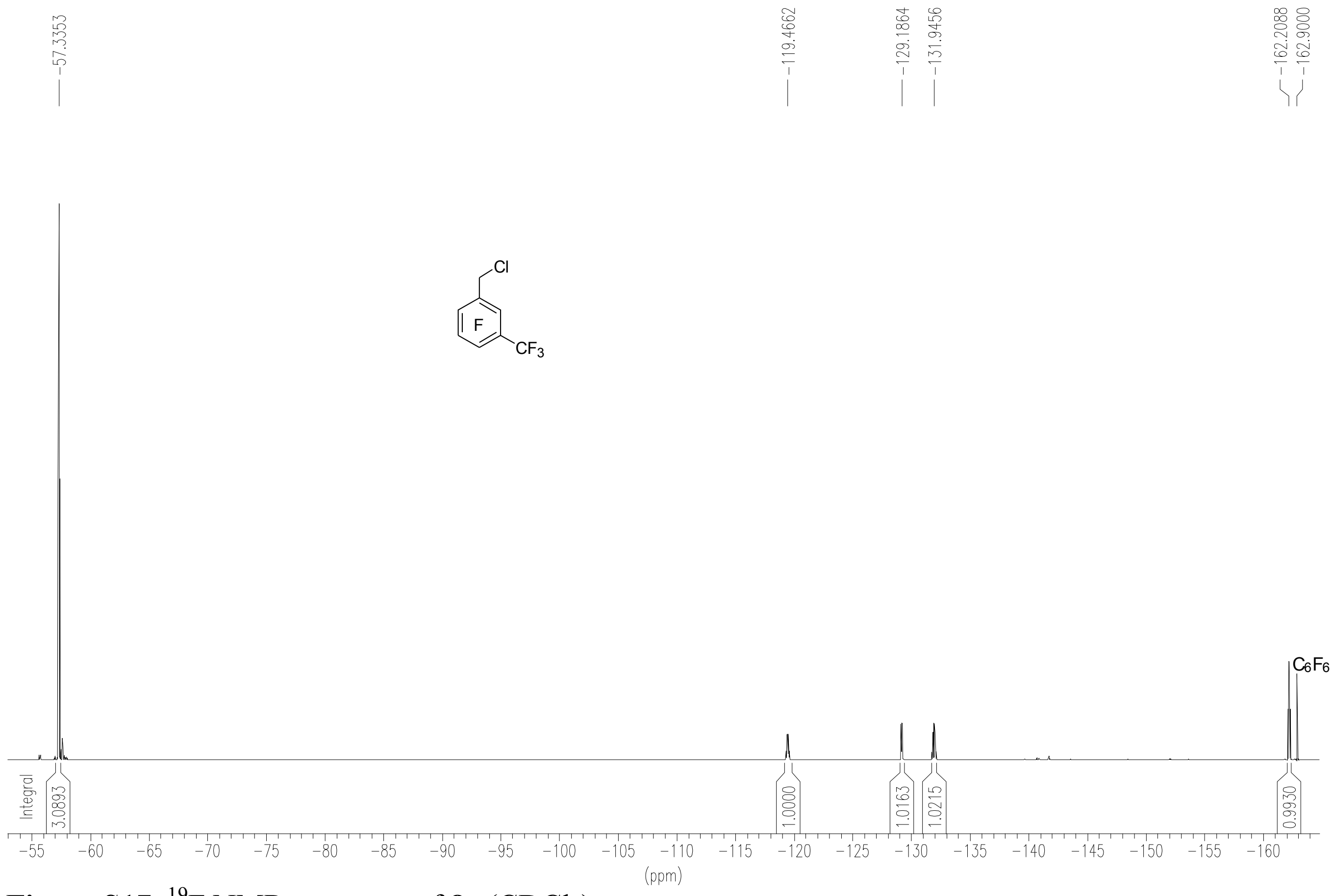

Figure S17. ^{19}F NMR spectrum of **8e** ($CDCl_3$).

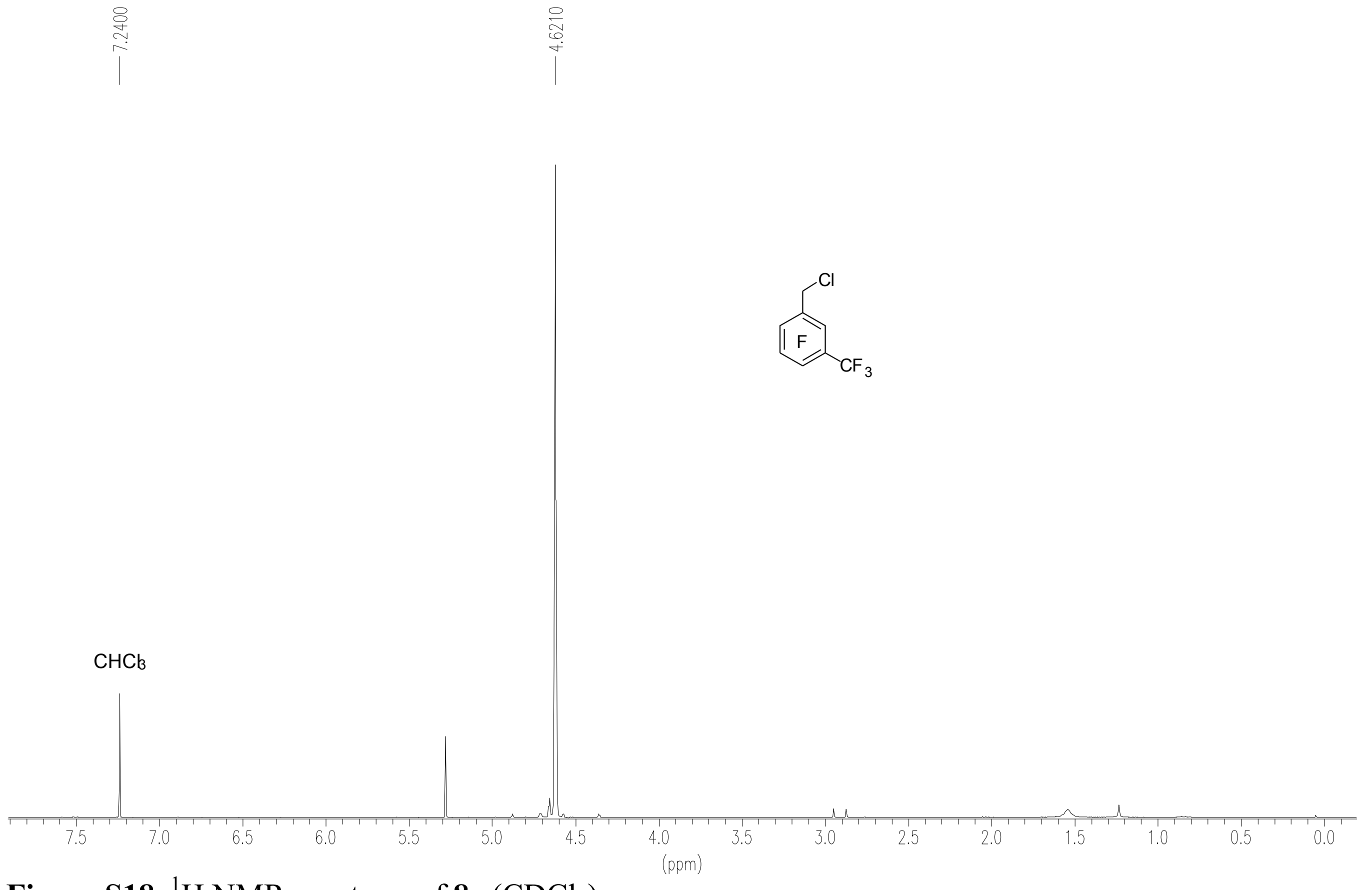

Figure S18. ^{1}H NMR spectrum of **8e** ($CDCl_3$).

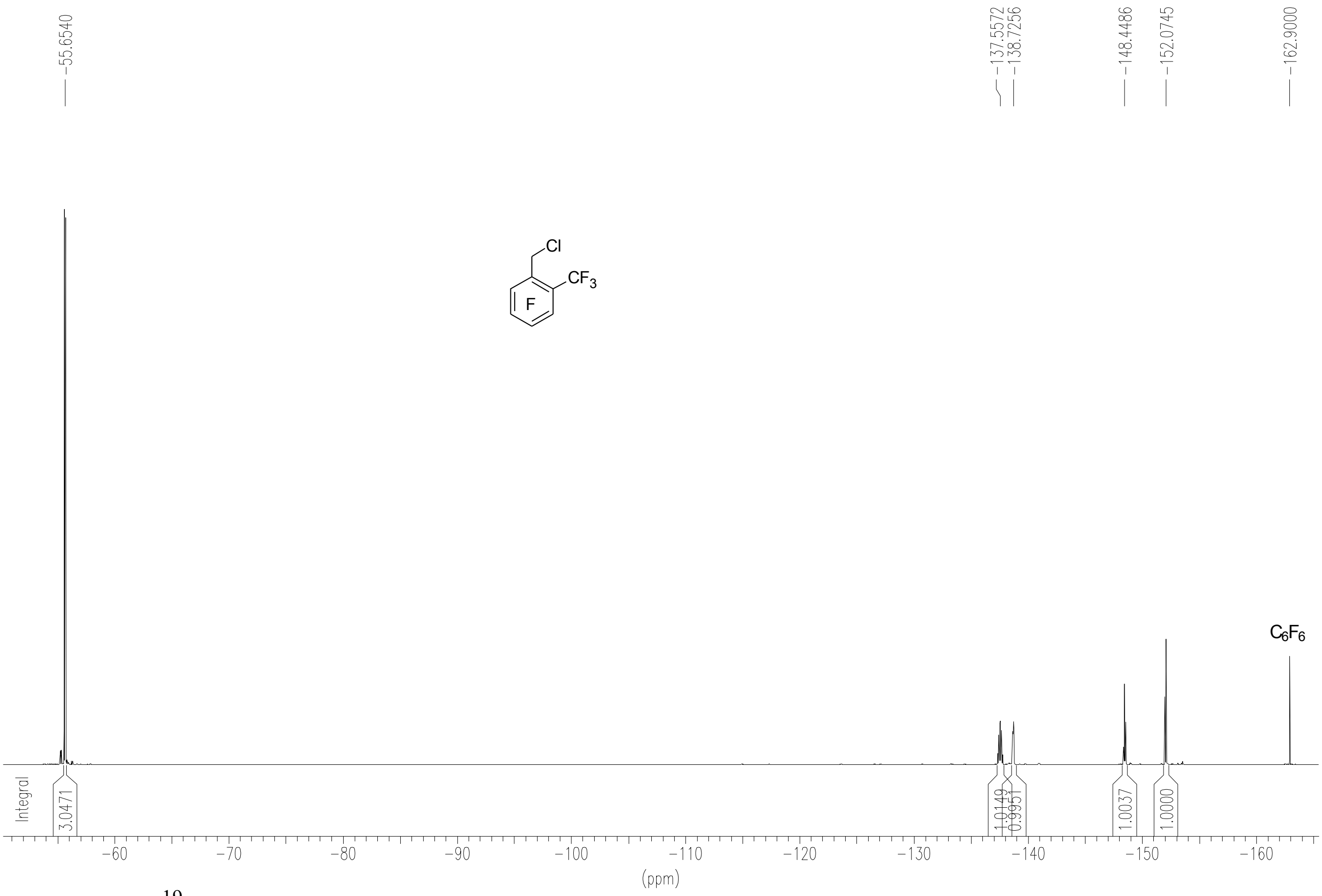

Figure S19. ^{19}F NMR spectrum of **8f** ($CDCl_3$).

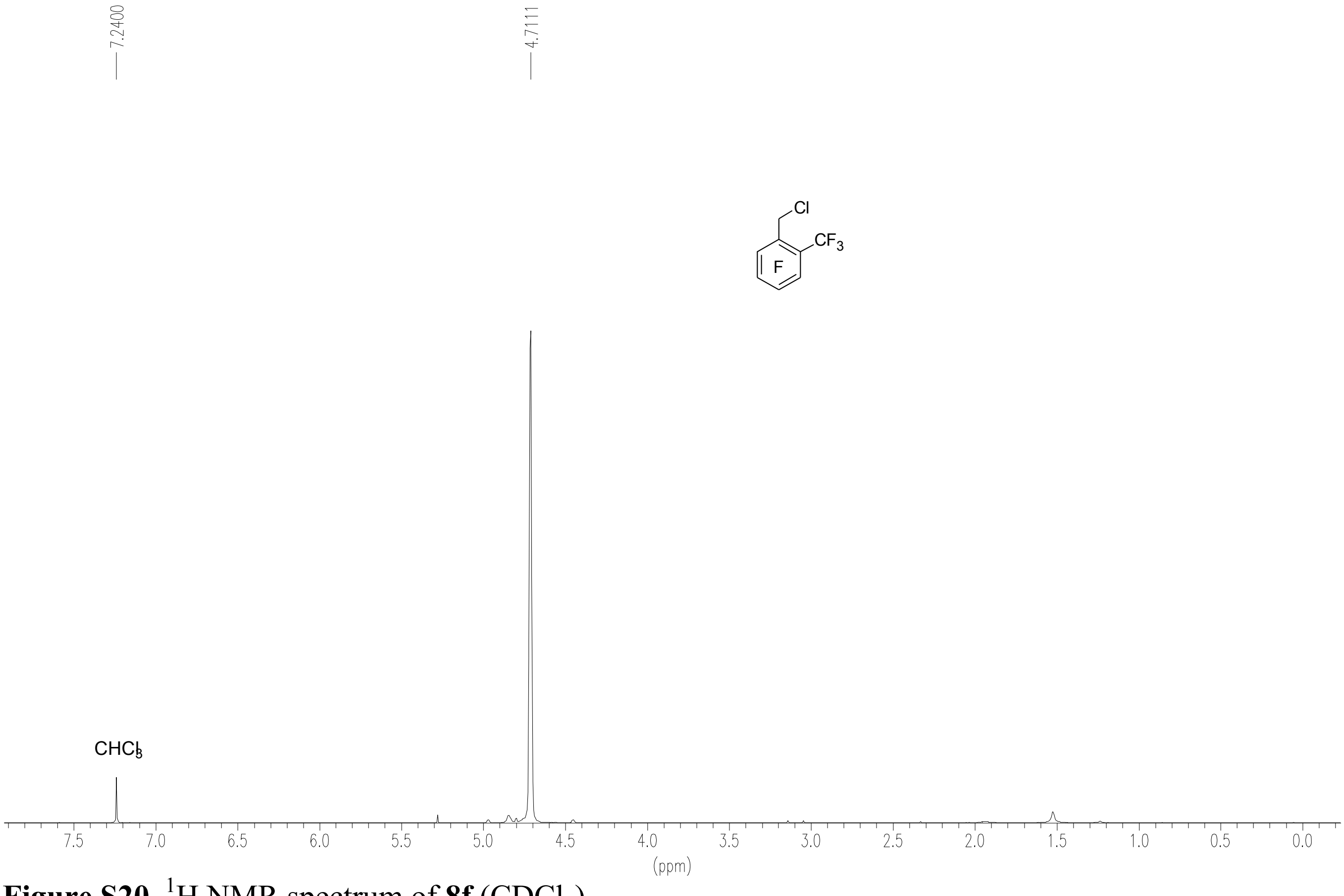

Figure S20. ^{1}H NMR spectrum of **8f** ($CDCl_3$).

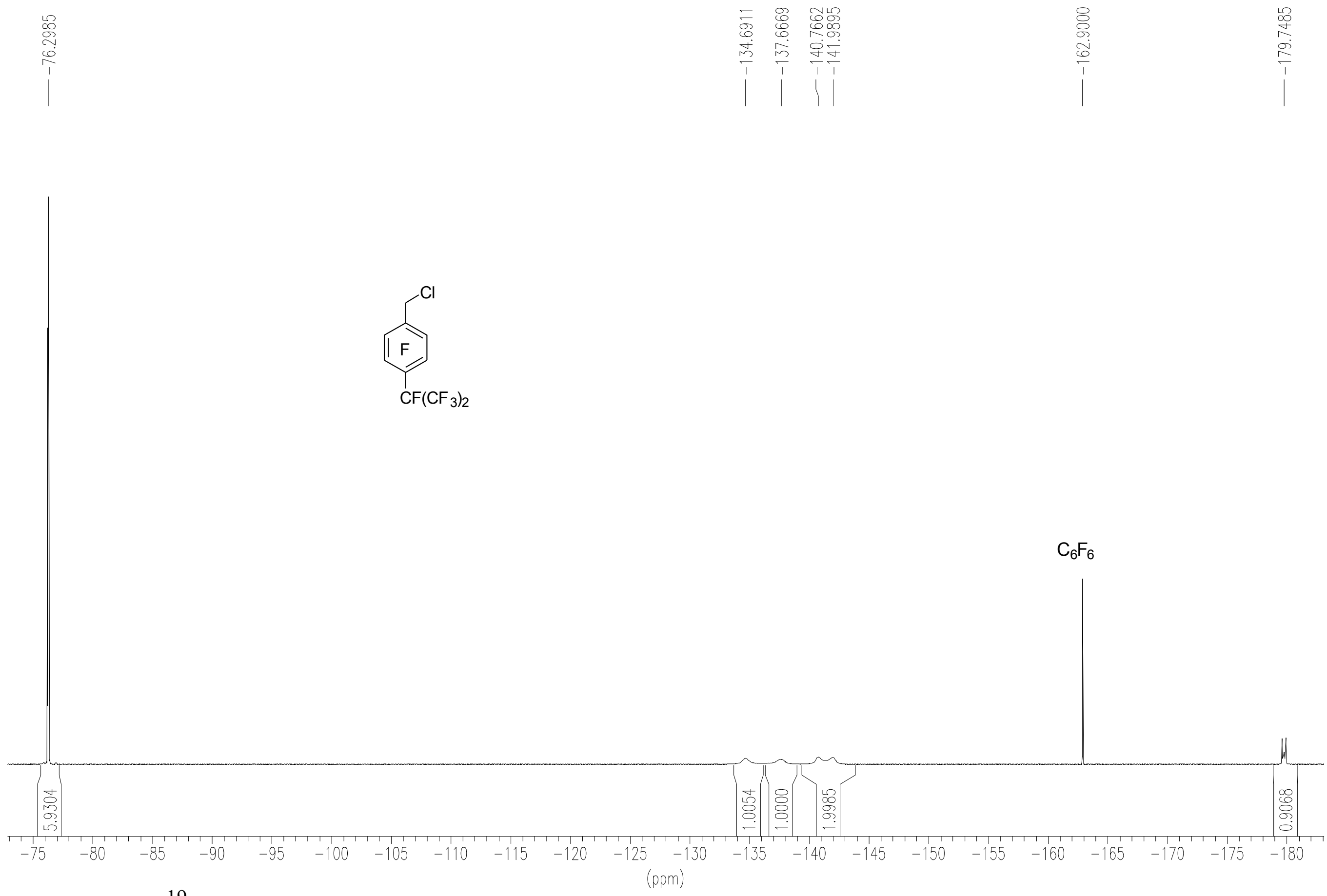

Figure S21. ^{19}F NMR spectrum of **8g** ($CDCl_3$).

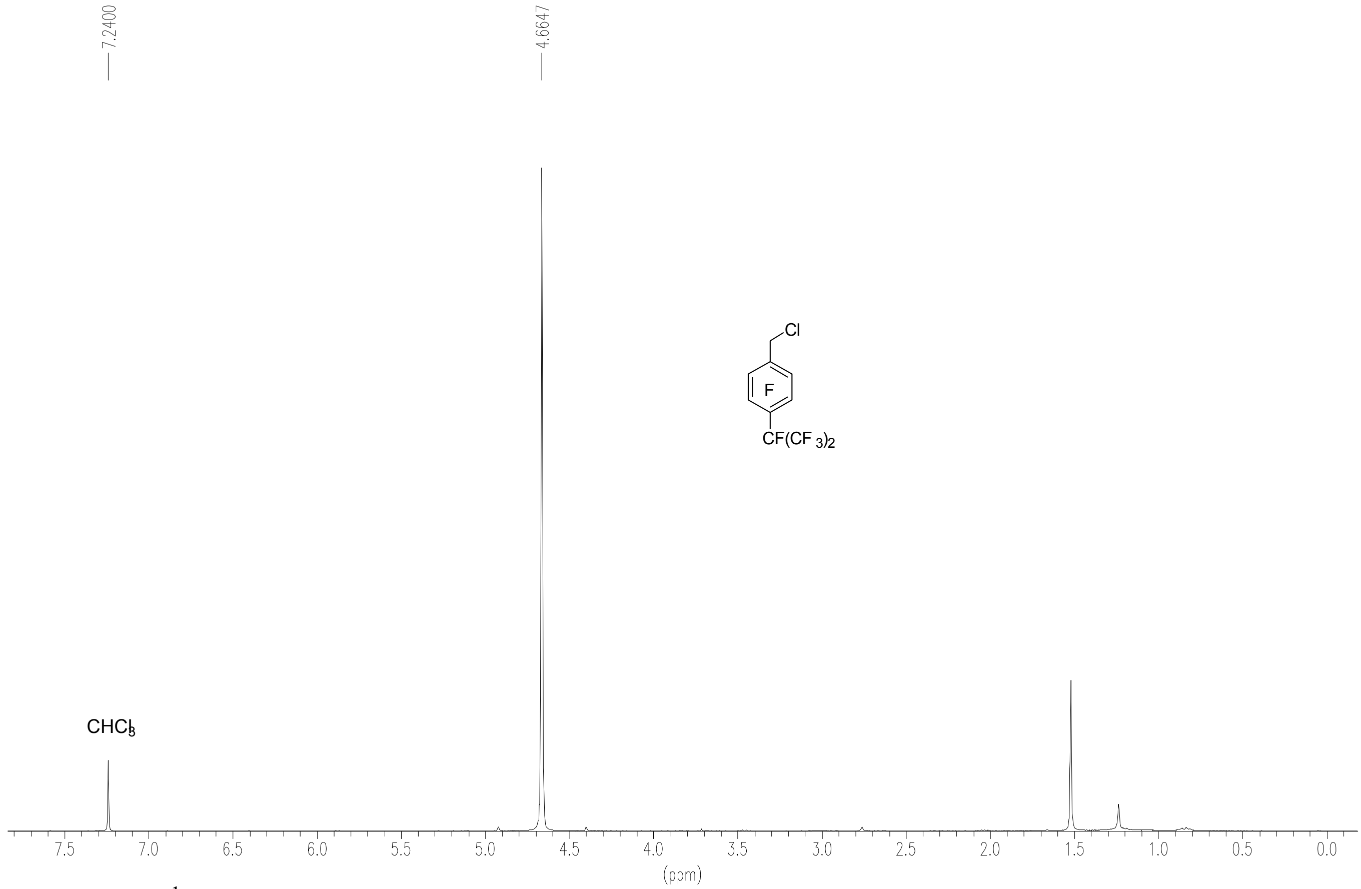

Figure S22. ^{1}H NMR spectrum of **8g** ($CDCl_3$).

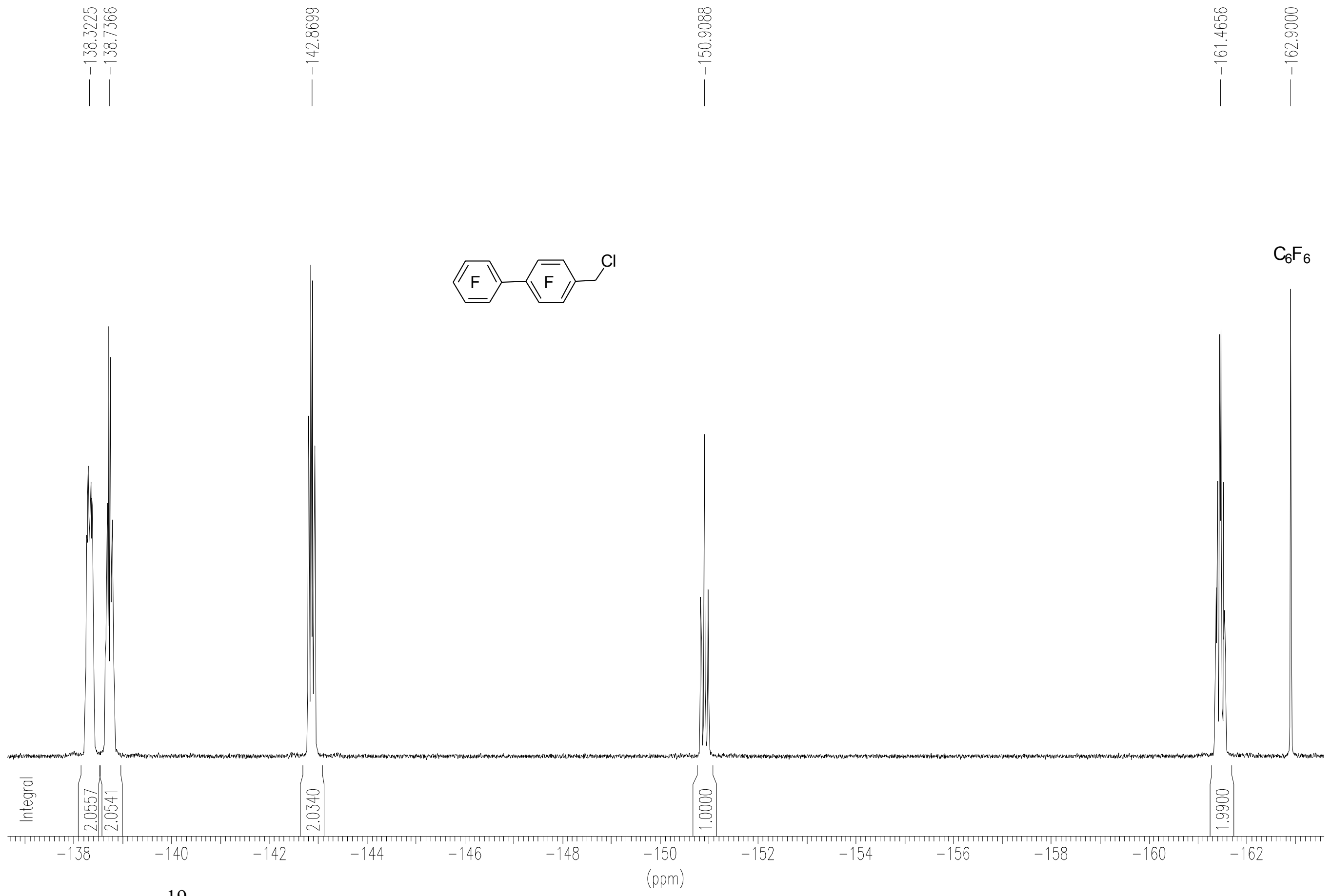

Figure S23. ^{19}F NMR spectrum of **8h** ($CDCl_3$).

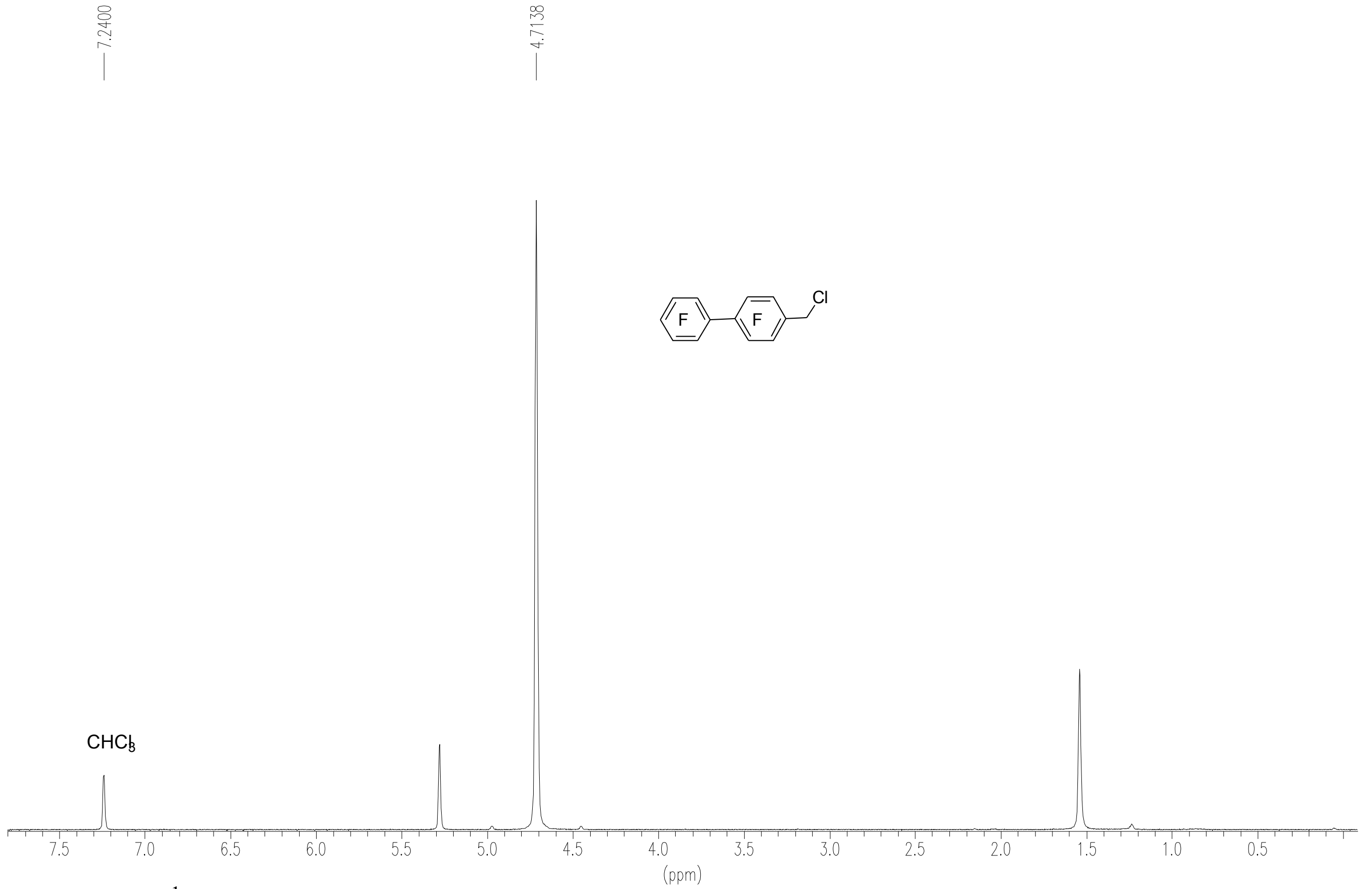

Figure S24. ^{1}H NMR spectrum of **8h** ($CDCl_3$).

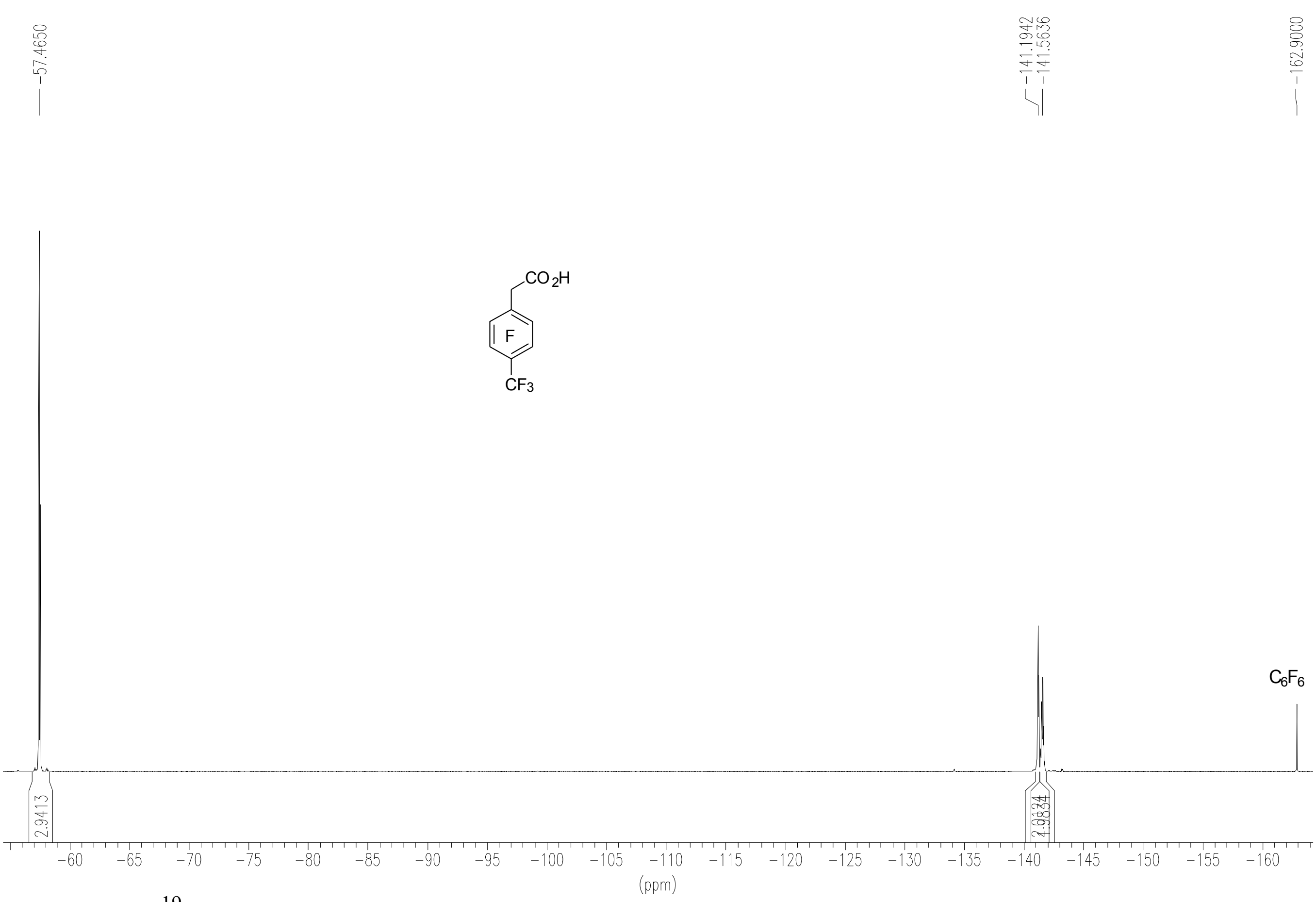

Figure S25. ^{19}F NMR spectrum of **9d** ($CDCl_3$).

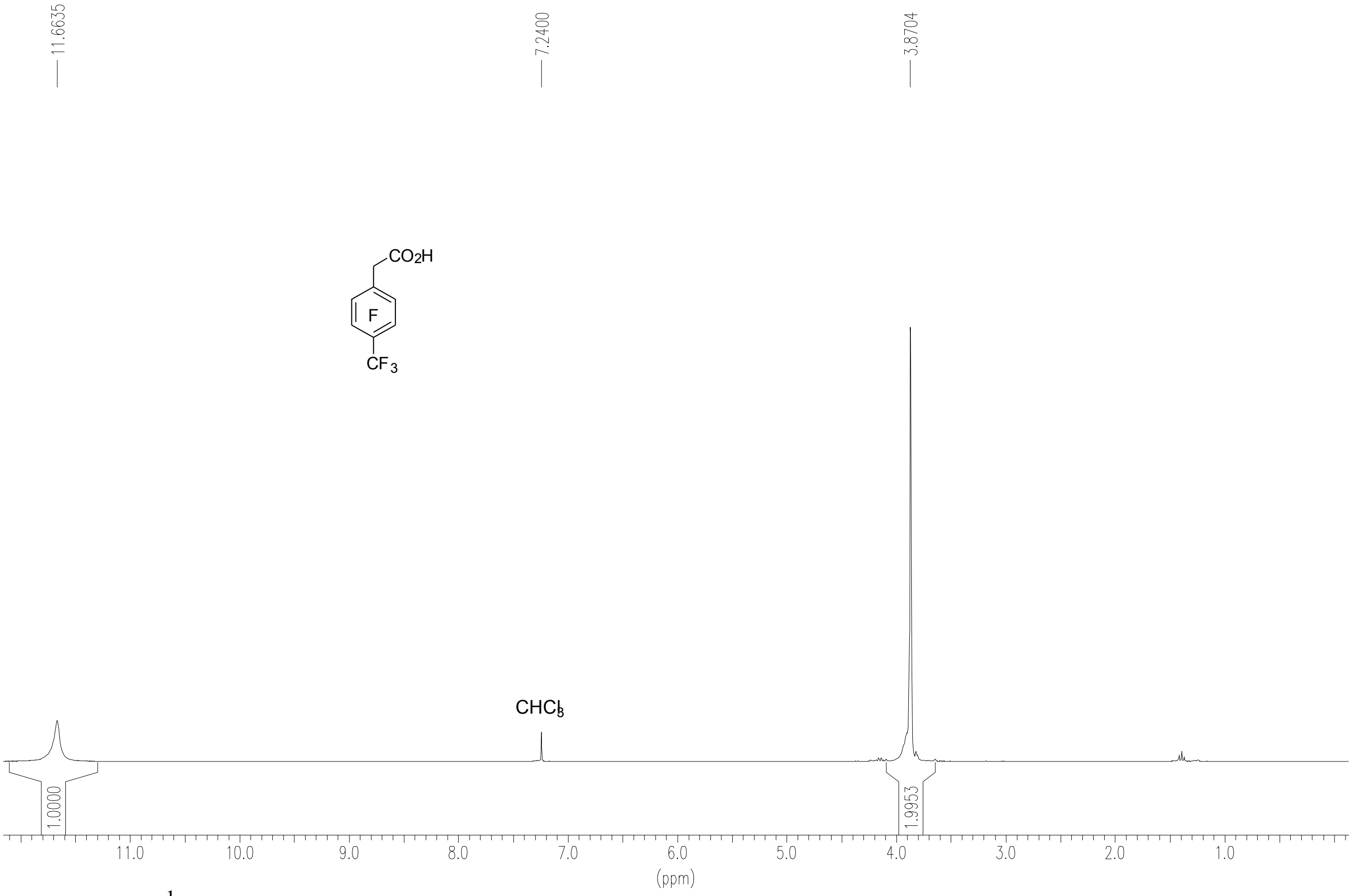

Figure S26. ^{1}H NMR spectrum of **9d** ($CDCl_3$).

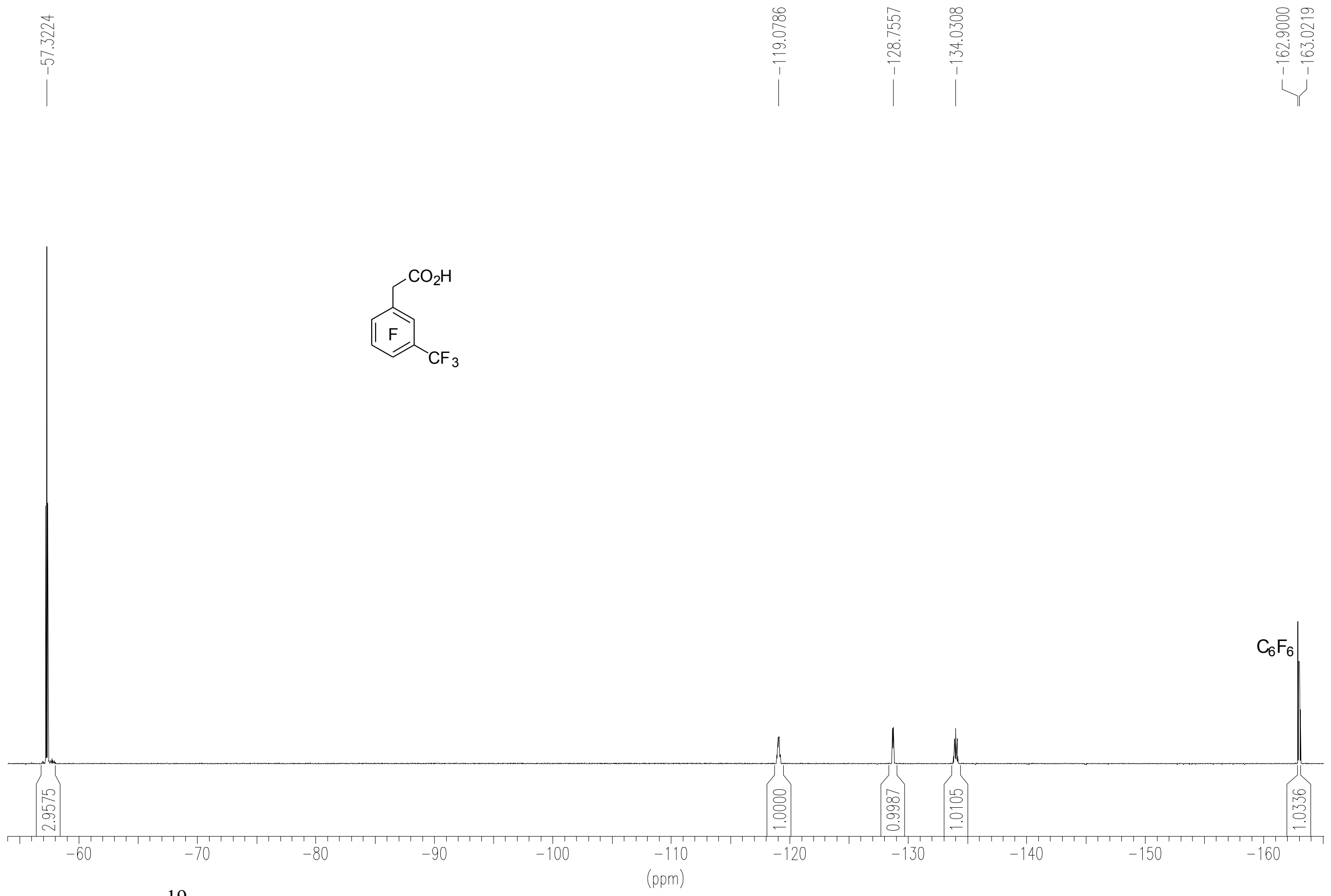

Figure S27. ^{19}F NMR spectrum of **9e** ($CDCl_3$).

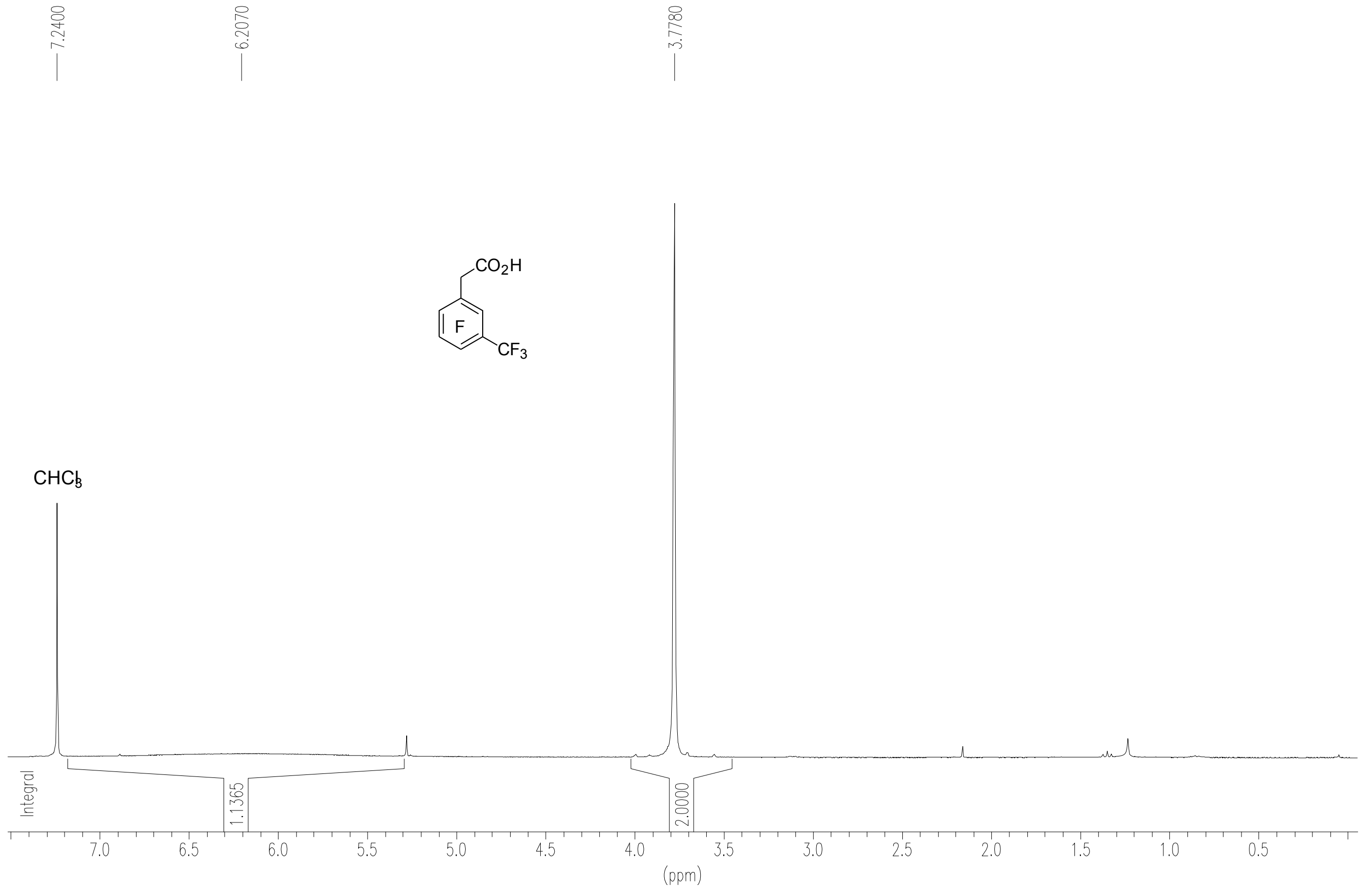

Figure S28. ^{1}H NMR spectrum of **9e** ($CDCl_3$).

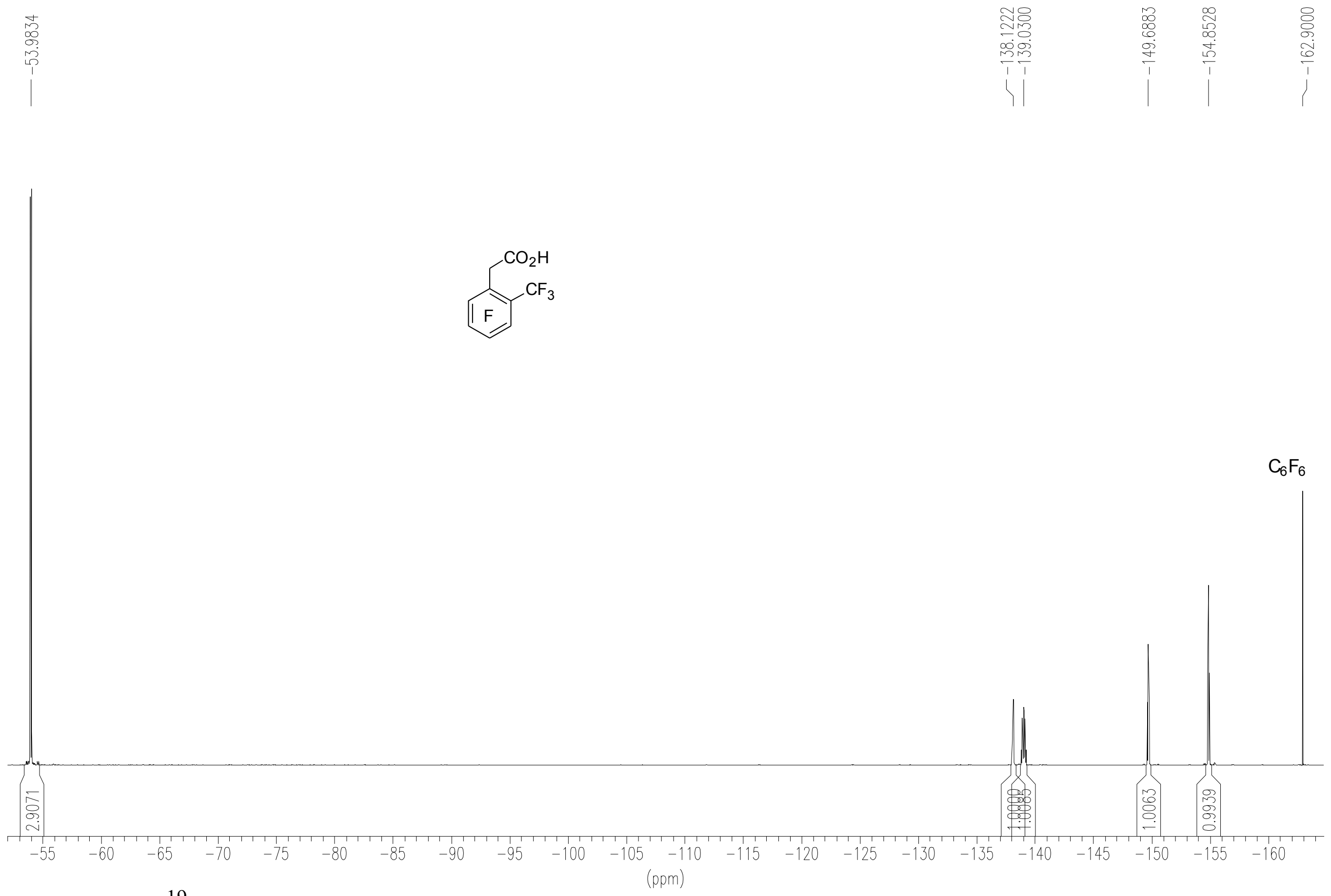

Figure S29. ^{19}F NMR spectrum of **9f** (acetone-d_6).

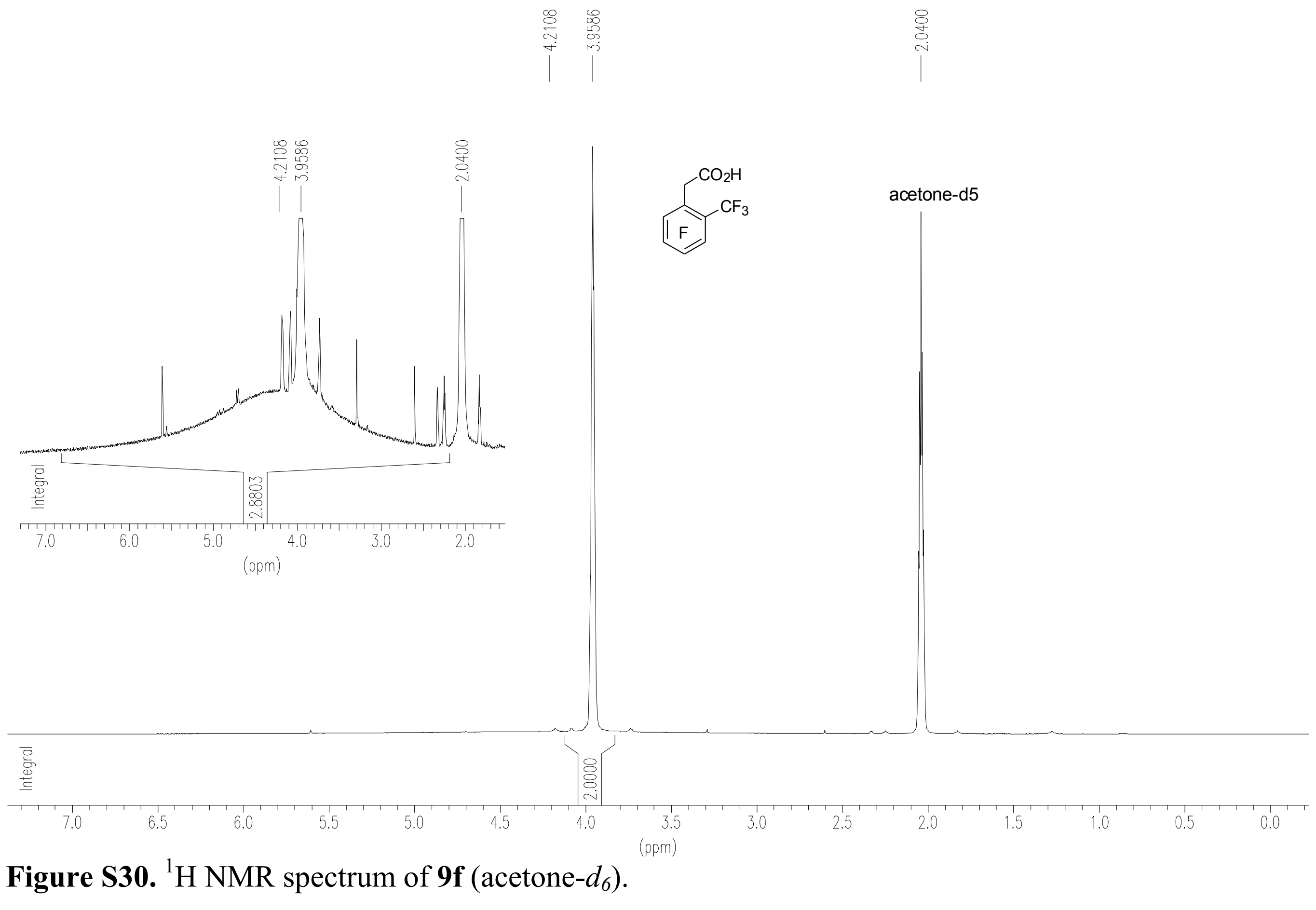

Figure S30. ^{1}H NMR spectrum of **9f** (acetone-d_6).

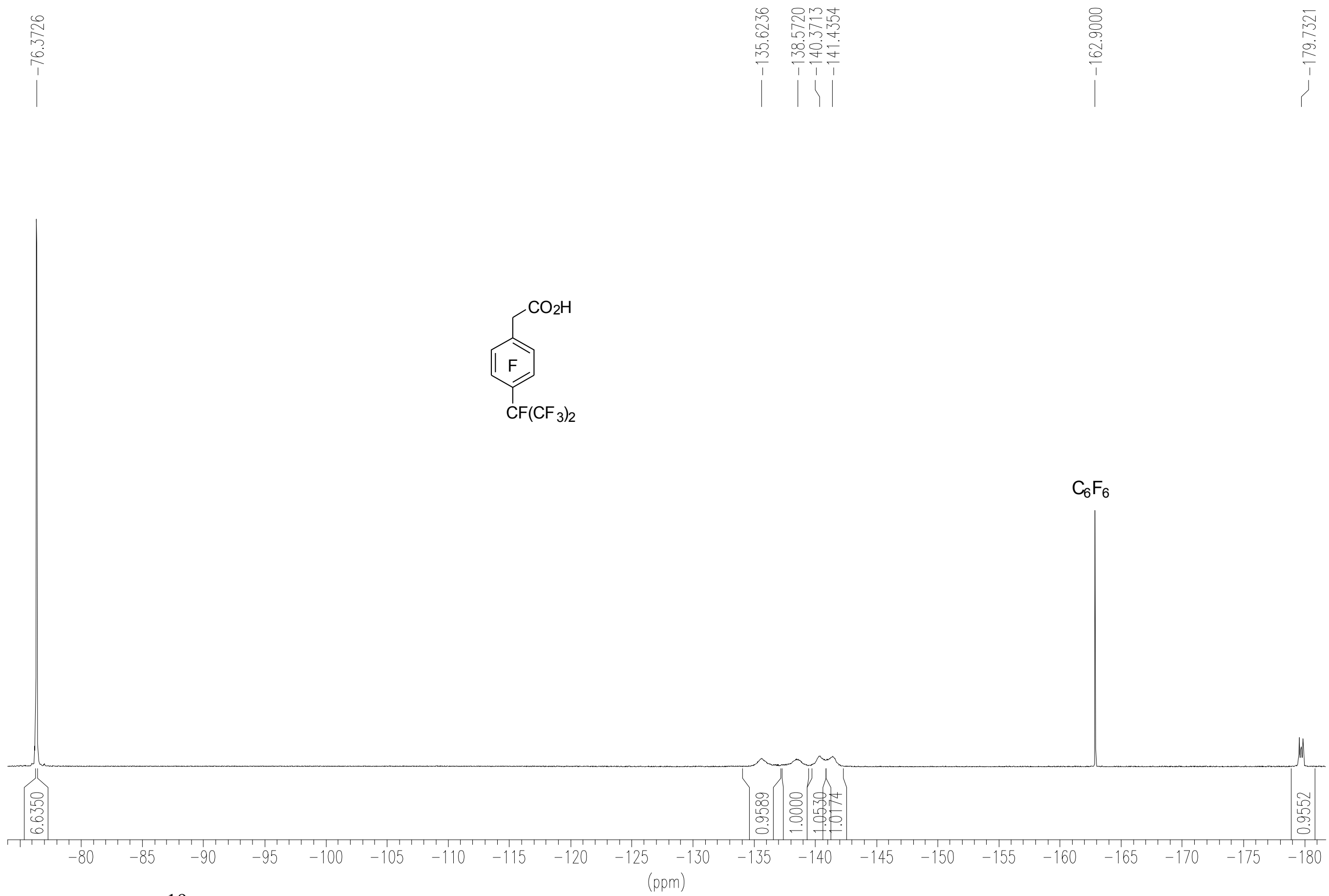

Figure S31. ^{19}F NMR spectrum of **9g** ($CDCl_3$).

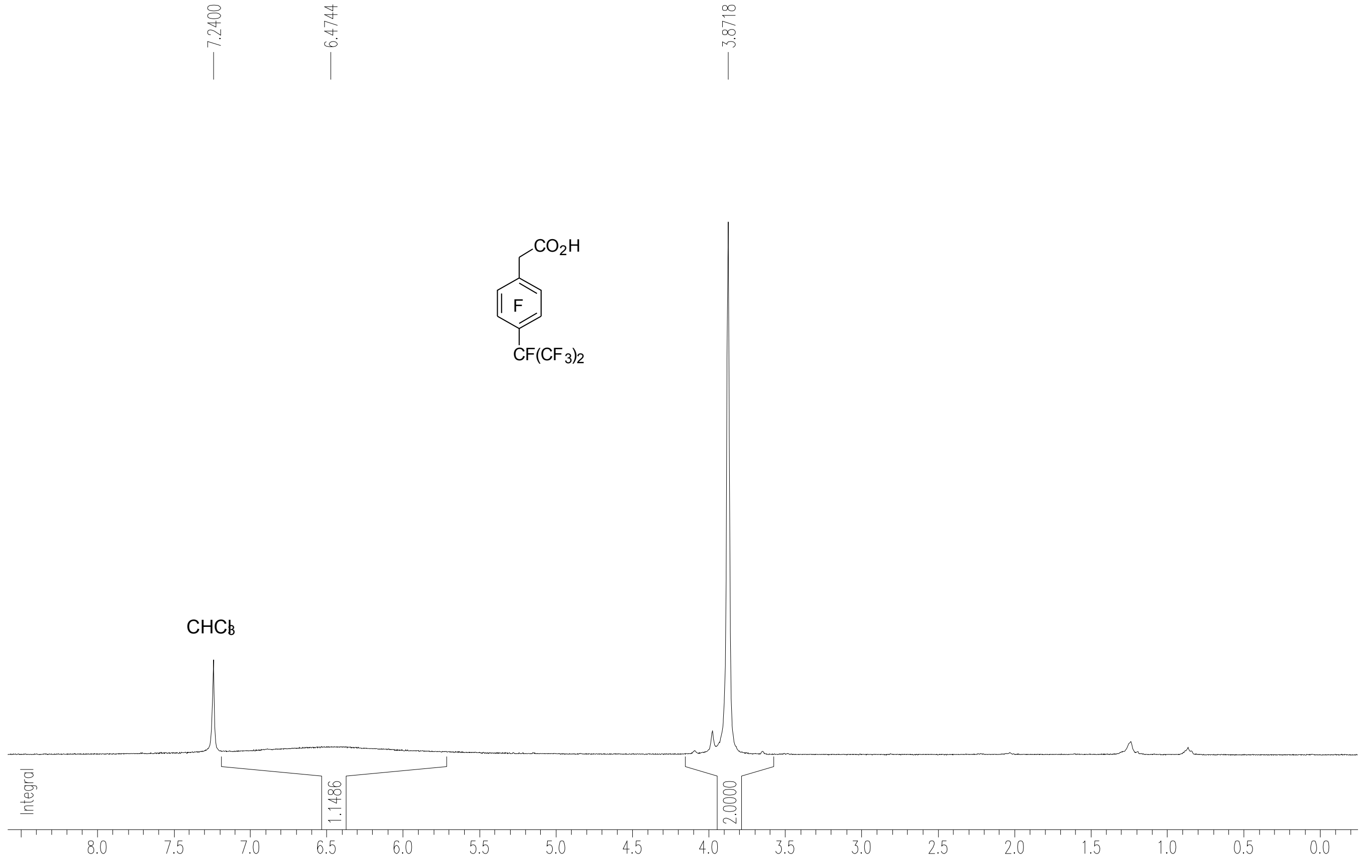

Figure S32. ^{1}H NMR spectrum of **9g** ($CDCl_3$).

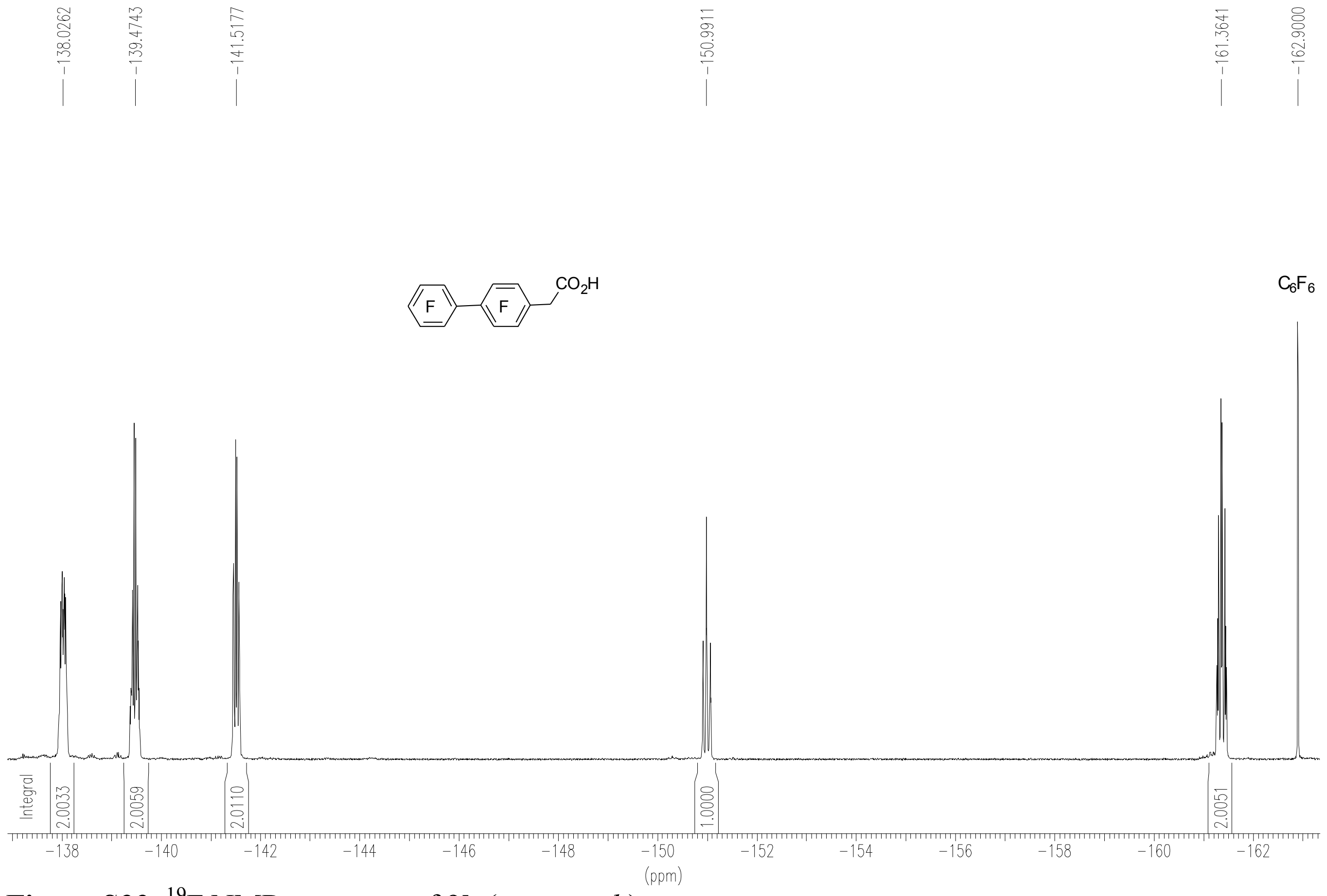

Figure S33. ^{19}F NMR spectrum of **9h** (acetone-d_6).

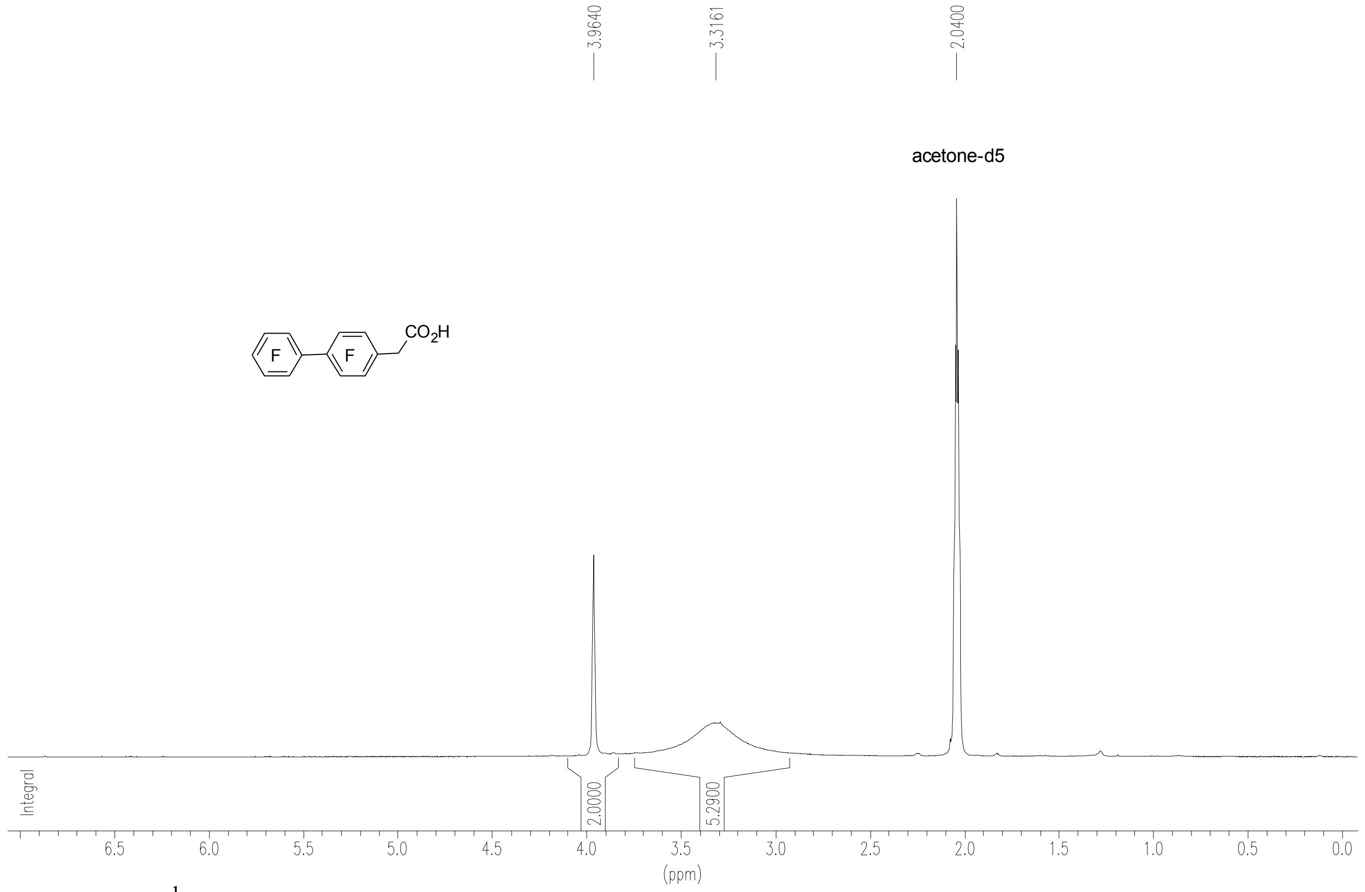

Figure S34. ^{1}H NMR spectrum of **9h** (acetone-d_6).

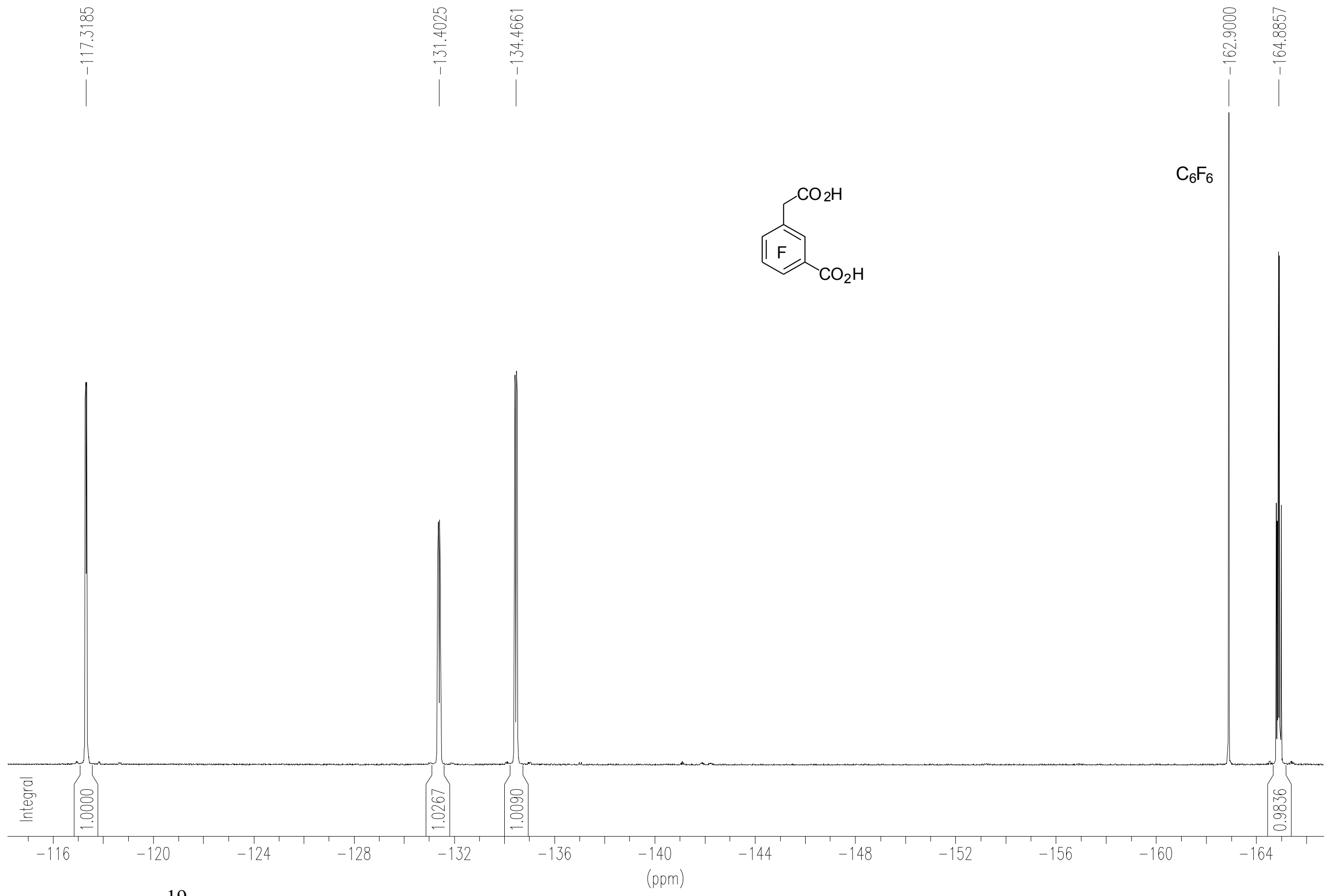

Figure S35. ^{19}F NMR spectrum of **11** (acetone-d_6).

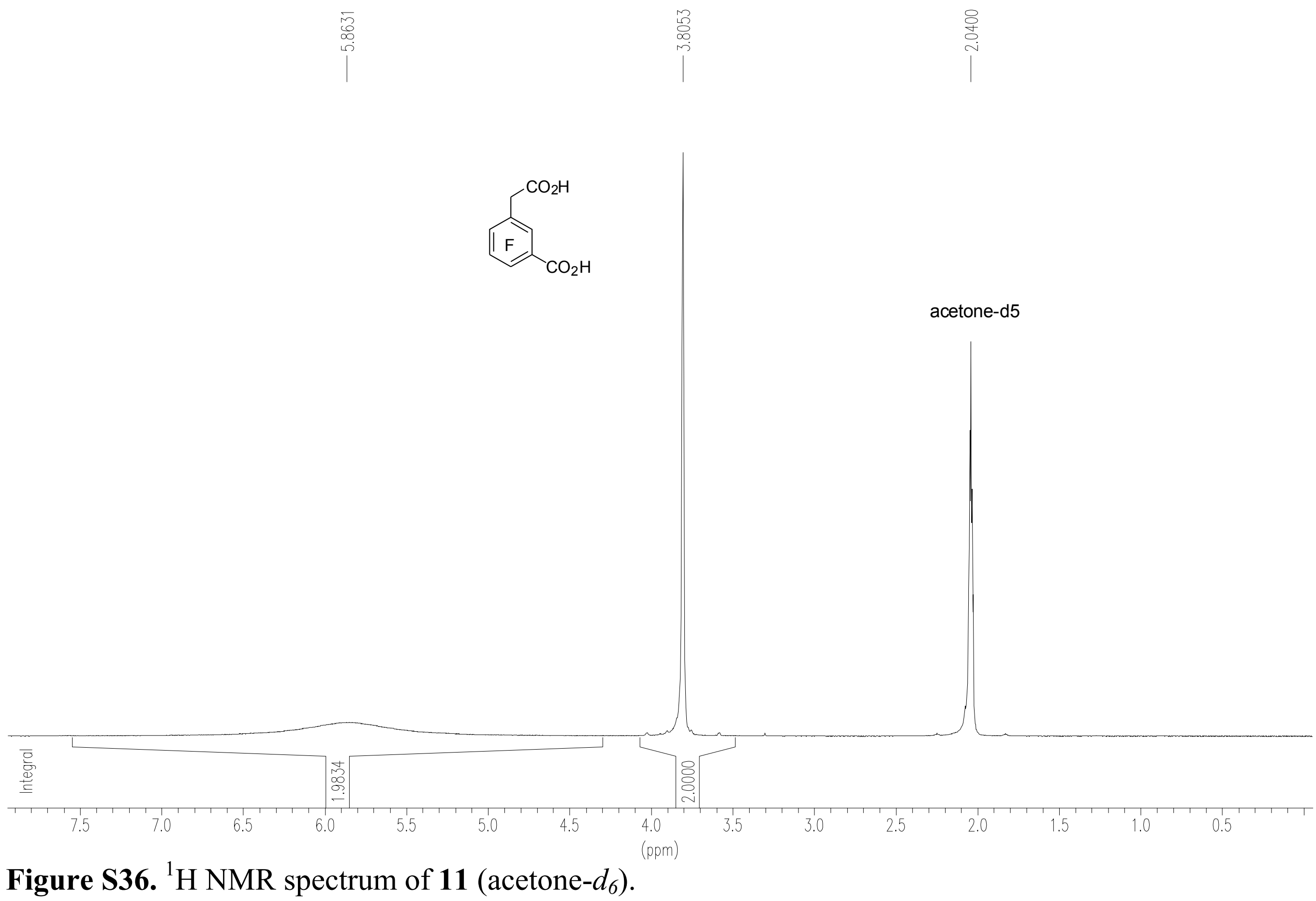

Figure S36. ^{1}H NMR spectrum of **11** (acetone-d_6).

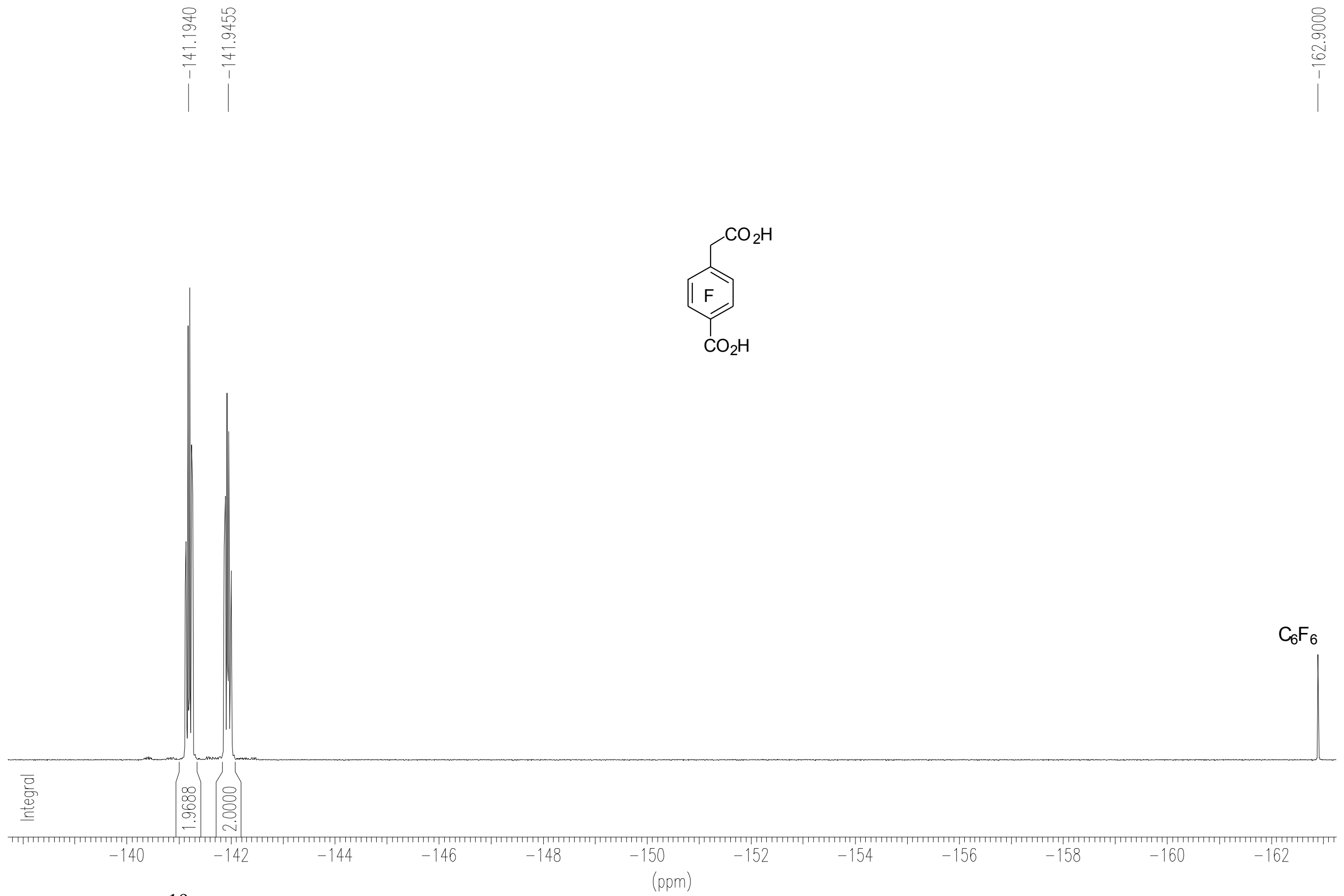

Figure S37. ^{19}F NMR spectrum of **12** (acetone-d_6).

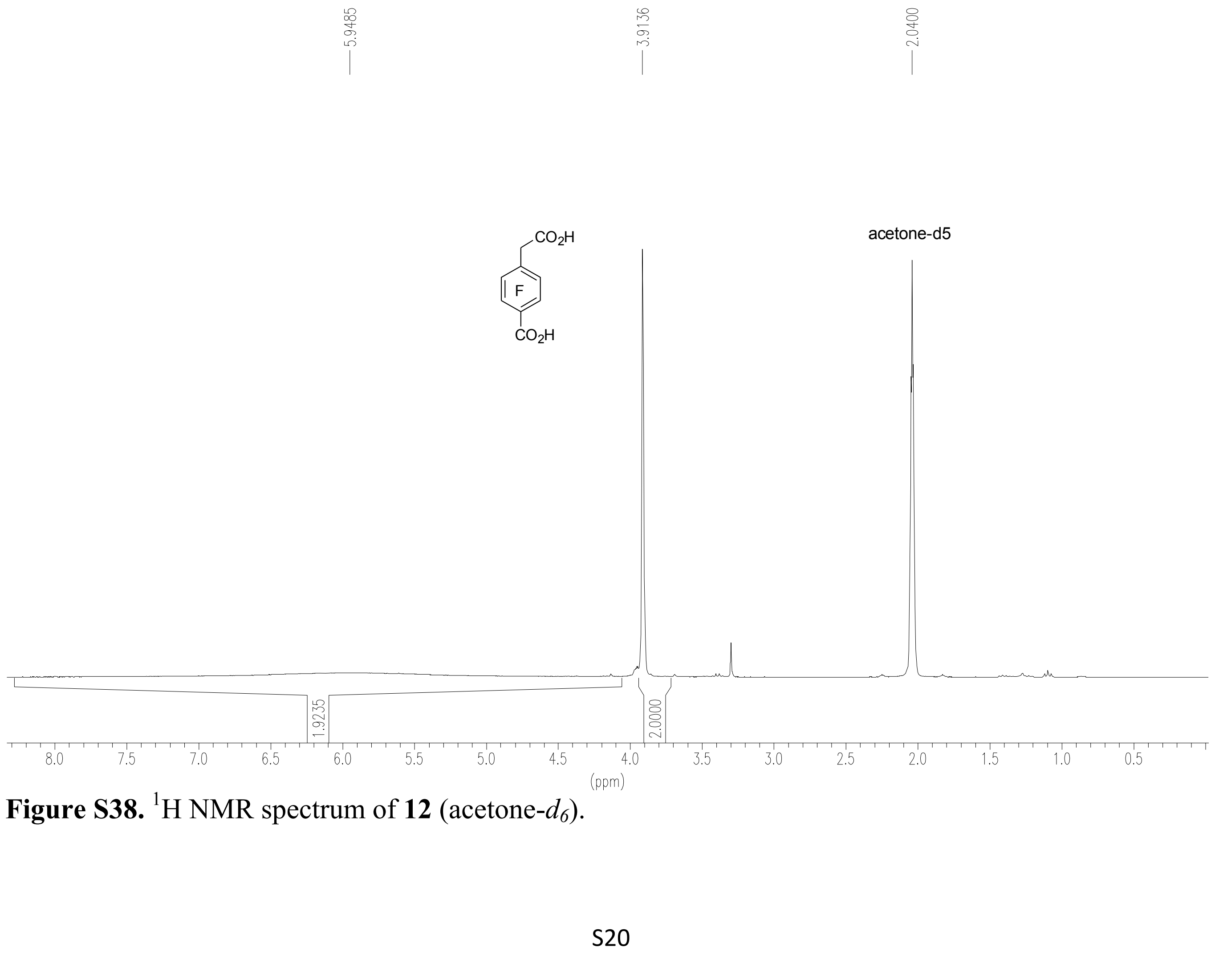

Figure S38. ^{1}H NMR spectrum of **12** (acetone-d_6).

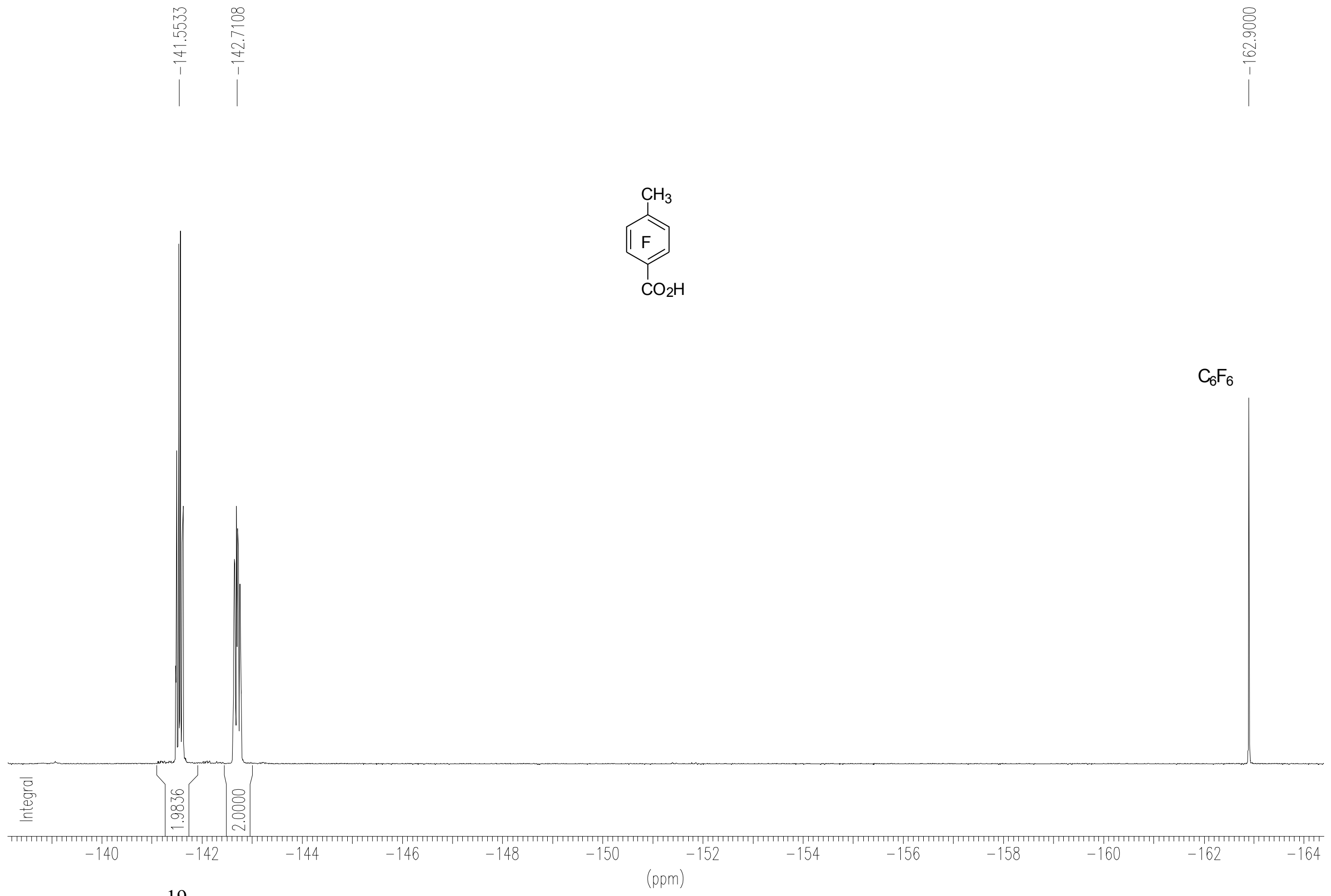

Figure S39. ^{19}F NMR spectrum of **13** (acetone-d_6).

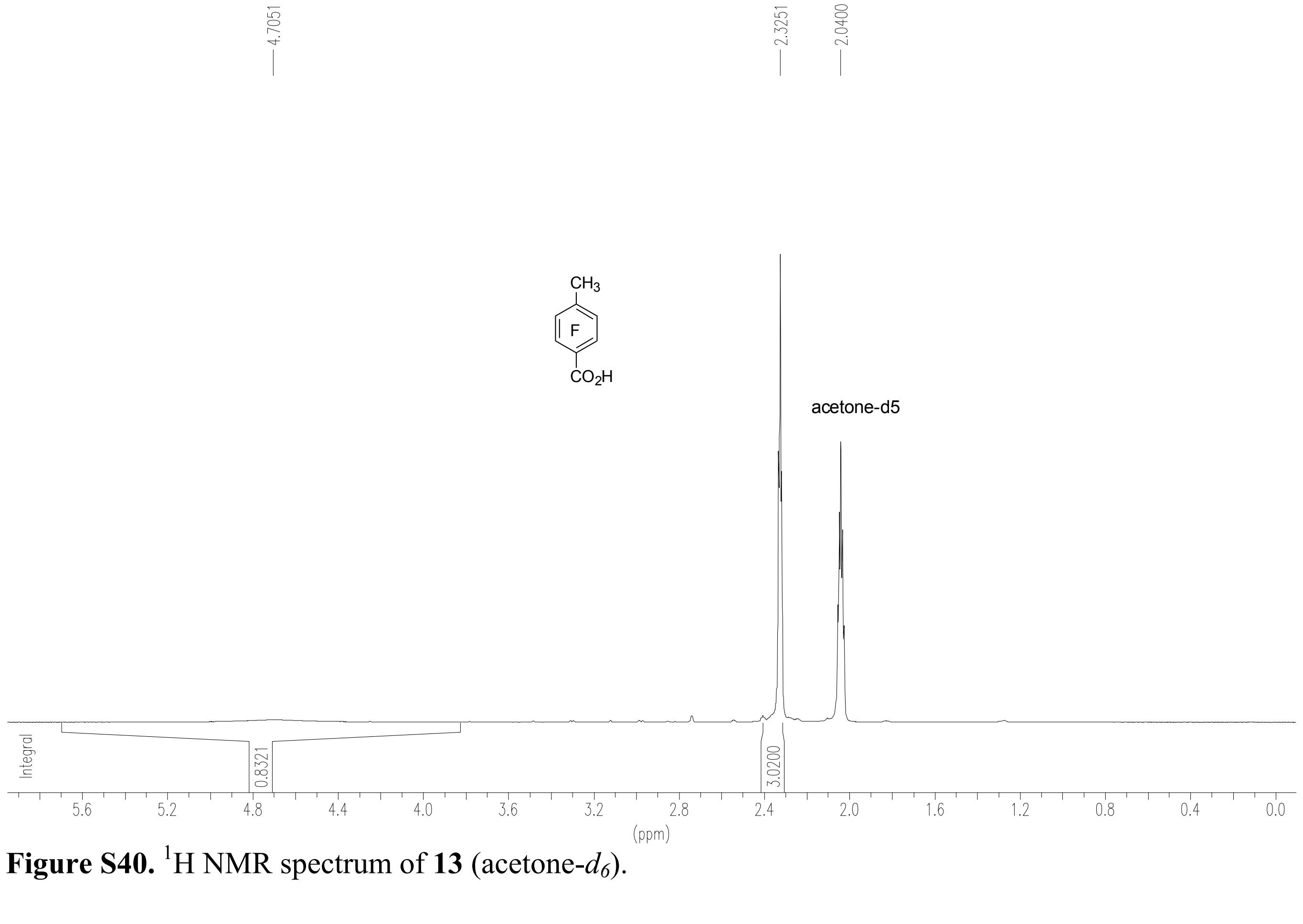

Figure S40. ^{1}H NMR spectrum of **13** (acetone-d_6).

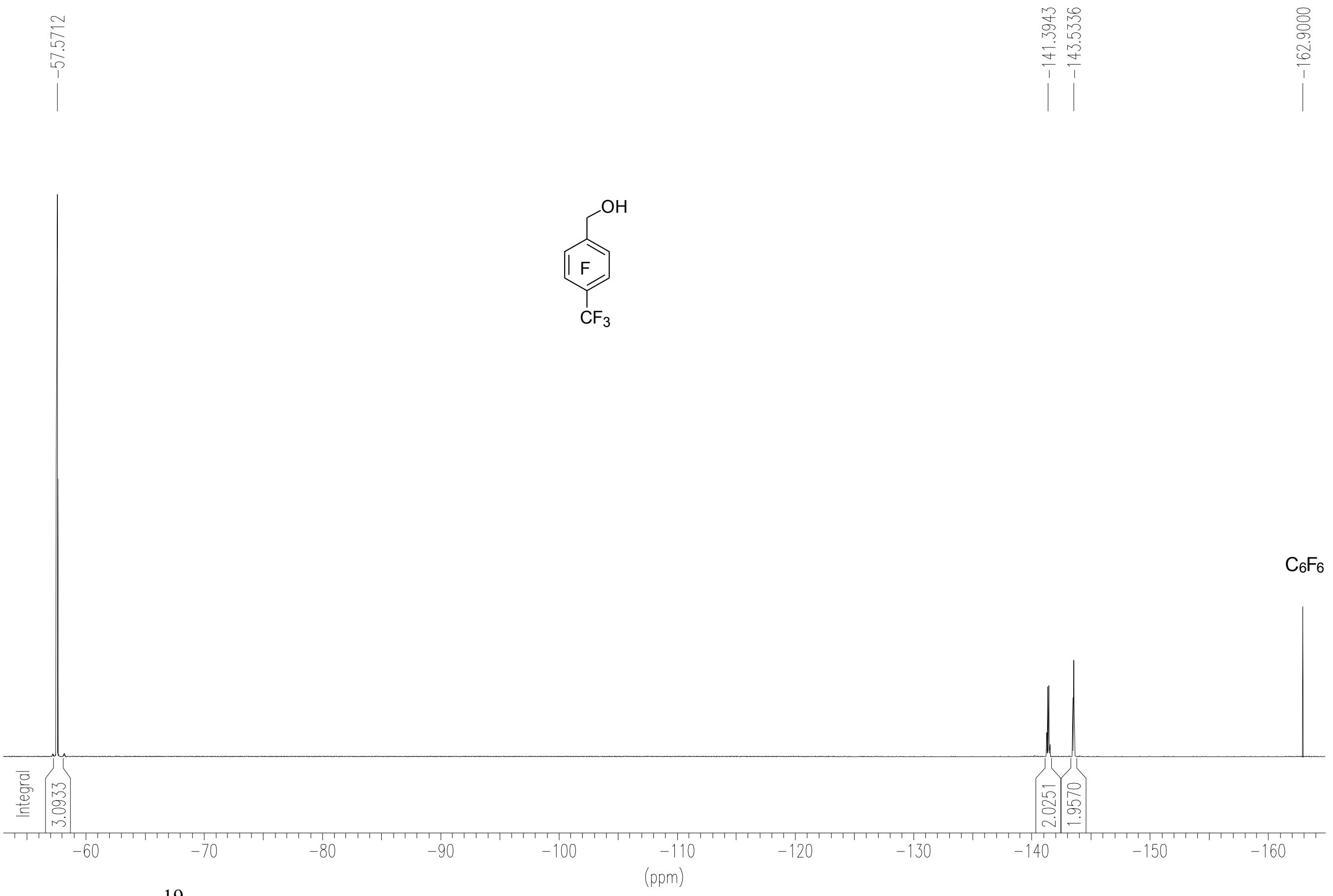

Figure S41. ^{19}F NMR spectrum of **14d** ($CDCl_3$).

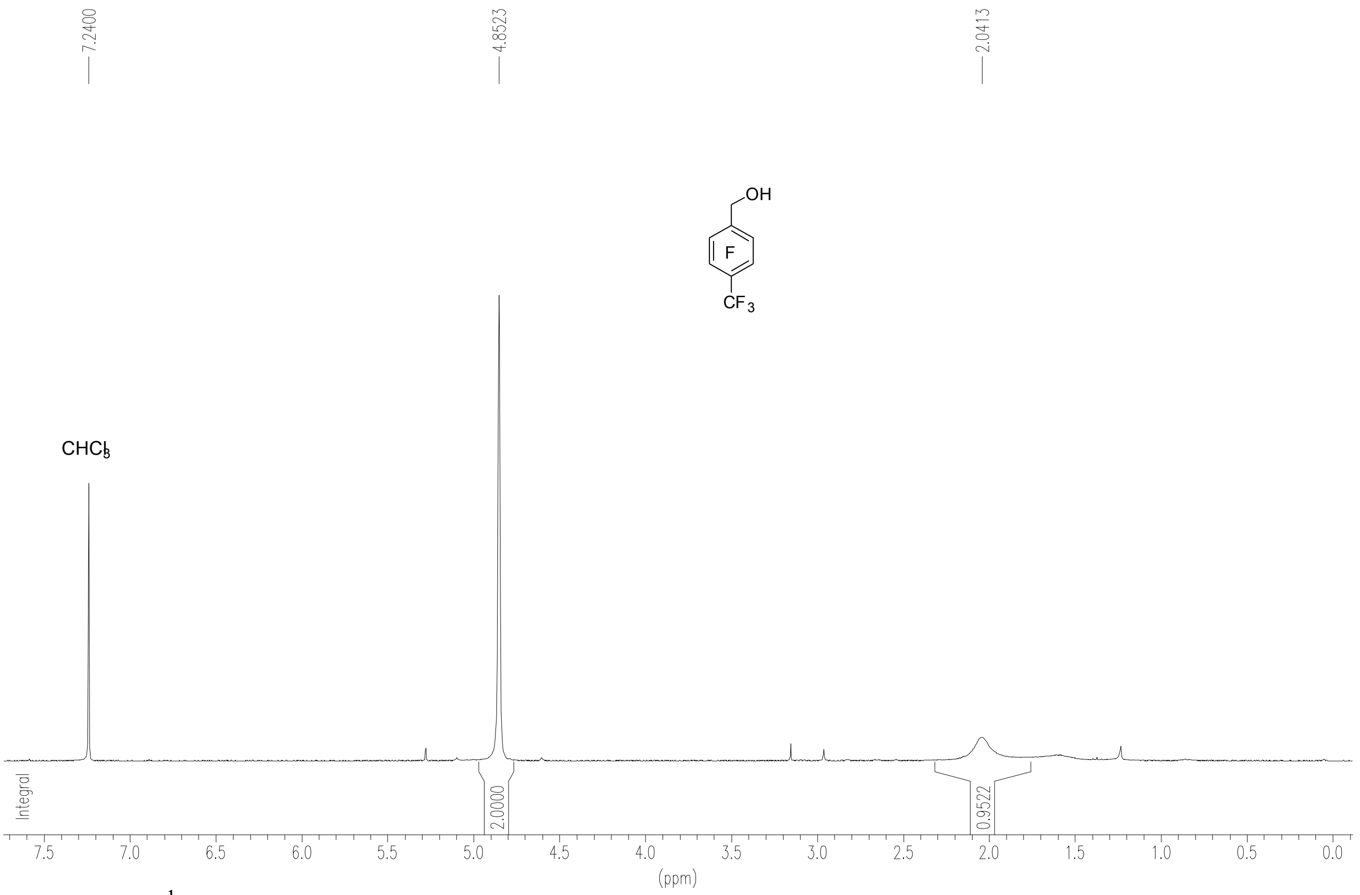

Figure S42. ^{1}H NMR spectrum of **14d** ($CDCl_3$).

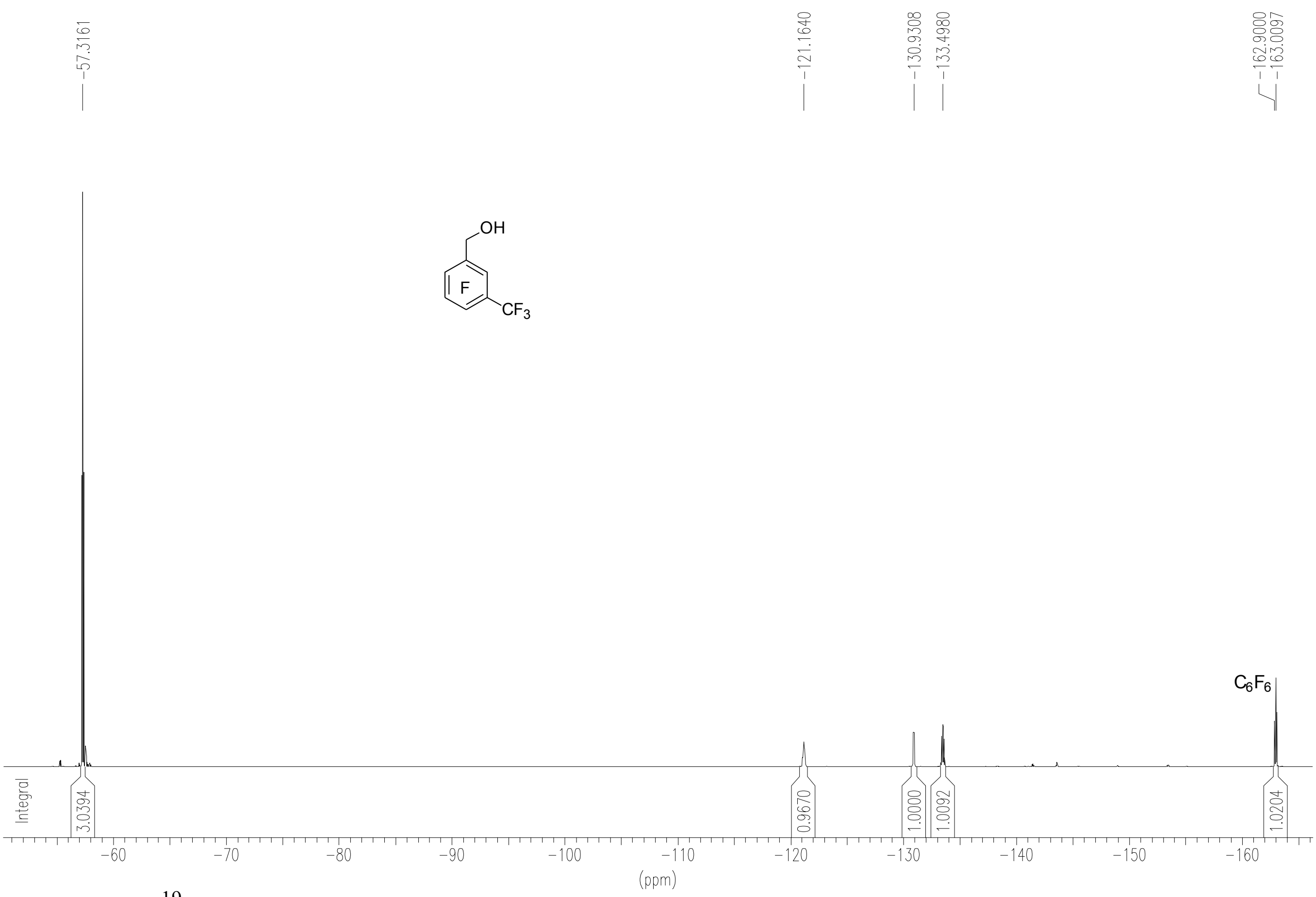

Figure S43. ^{19}F NMR spectrum of **14e** ($CDCl_3$).

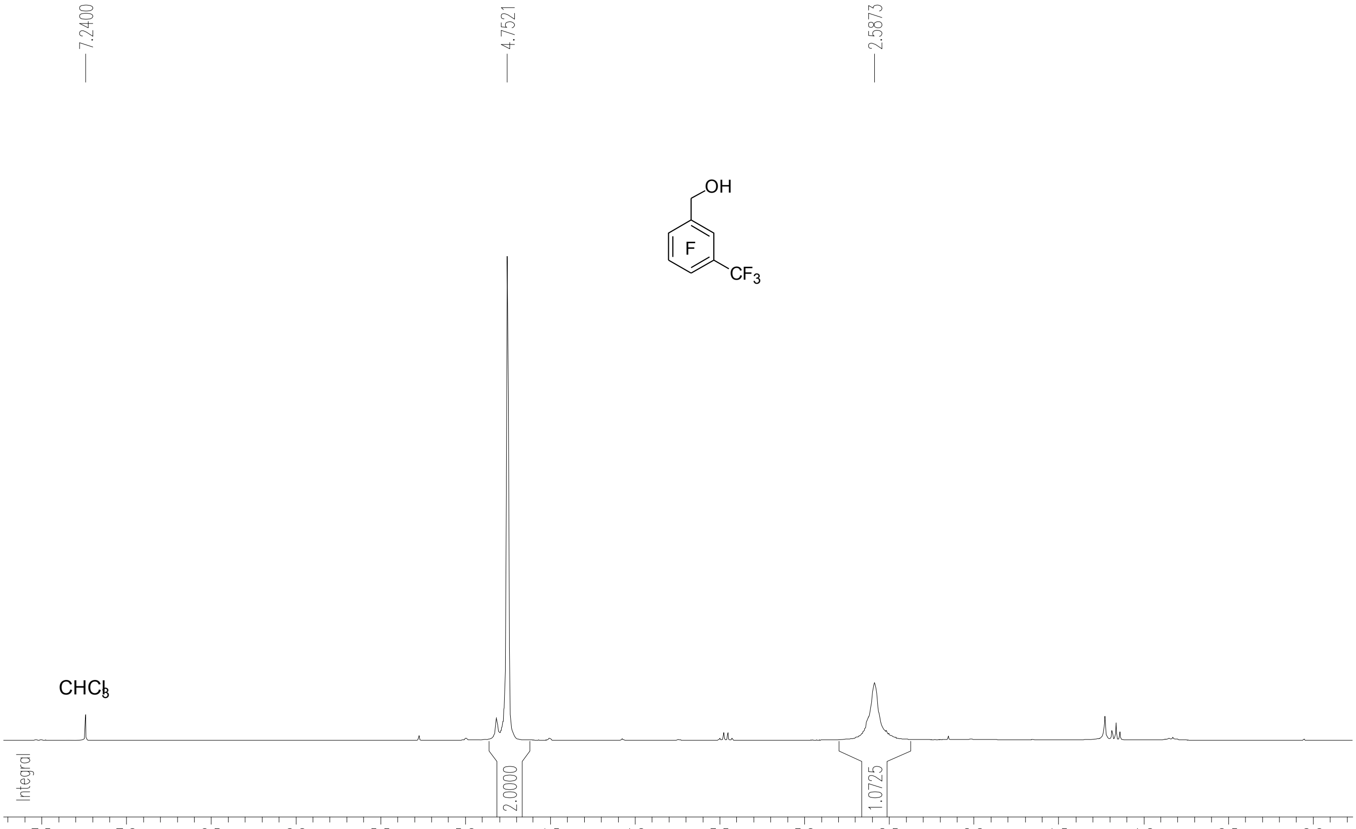

Figure S44. ^{1}H NMR spectrum of **14e** ($CDCl_3$).

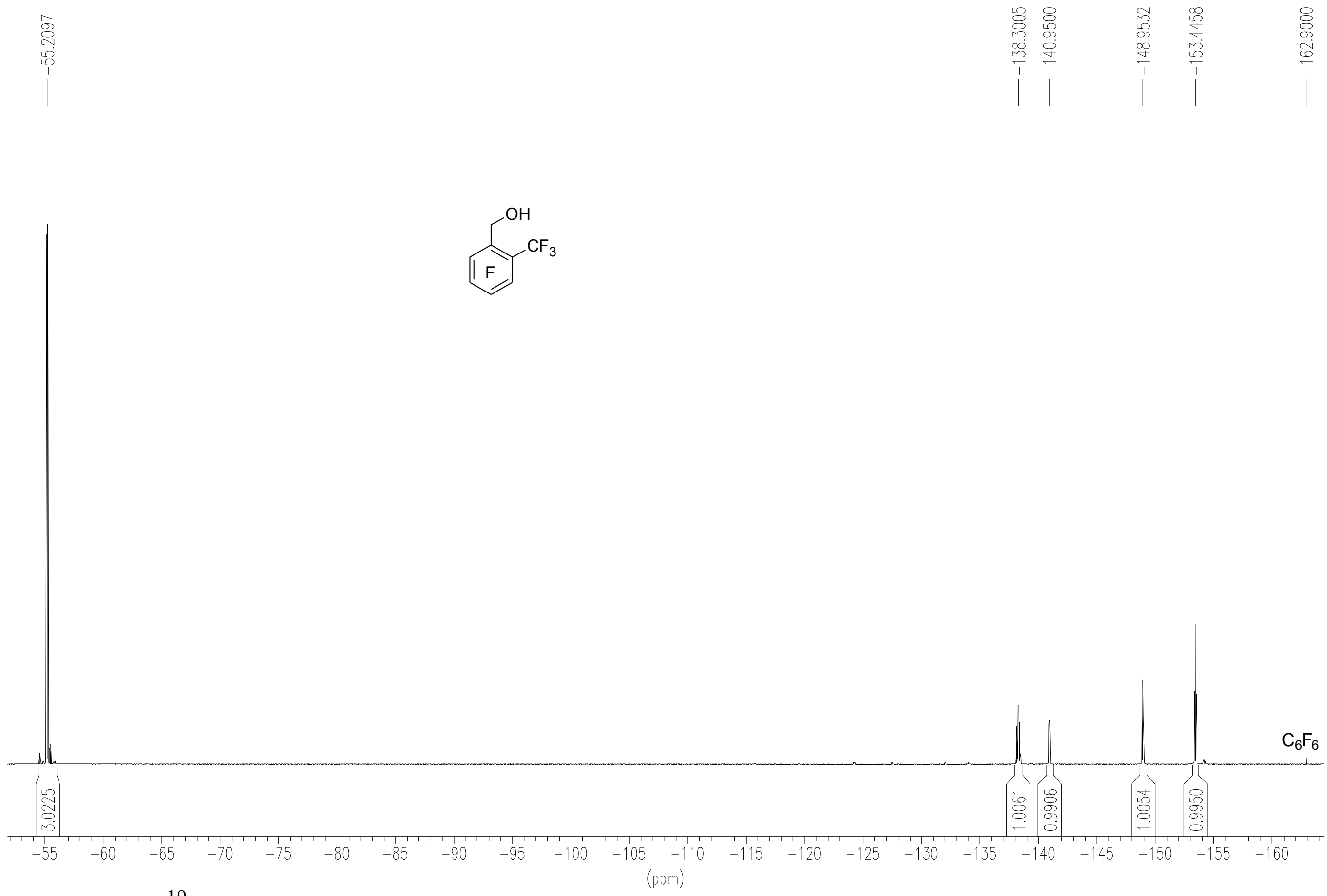

Figure S45. ^{19}F NMR spectrum of **14f** ($CDCl_3$).

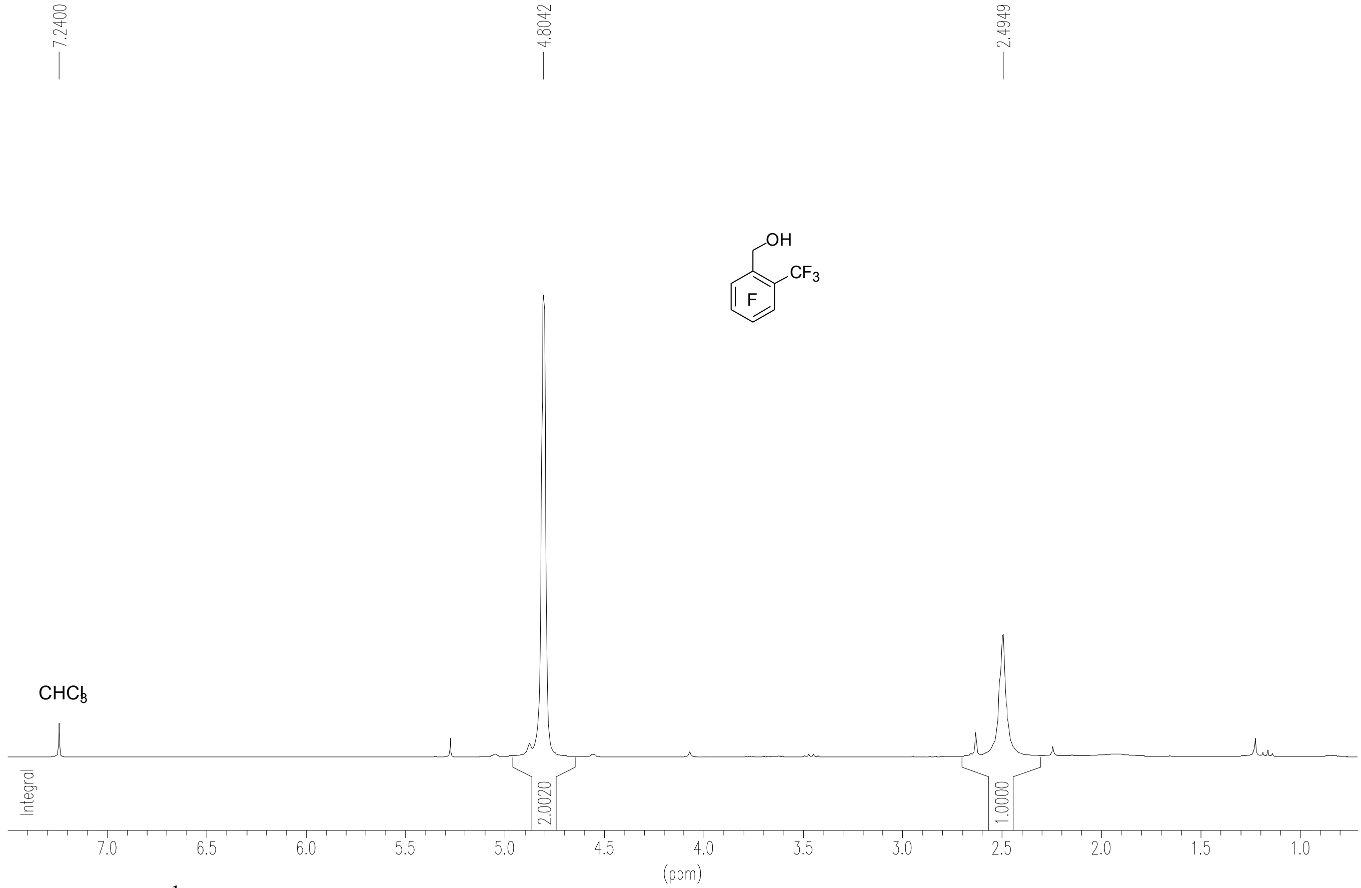

Figure S46. ^{1}H NMR spectrum of **14f** ($CDCl_3$).

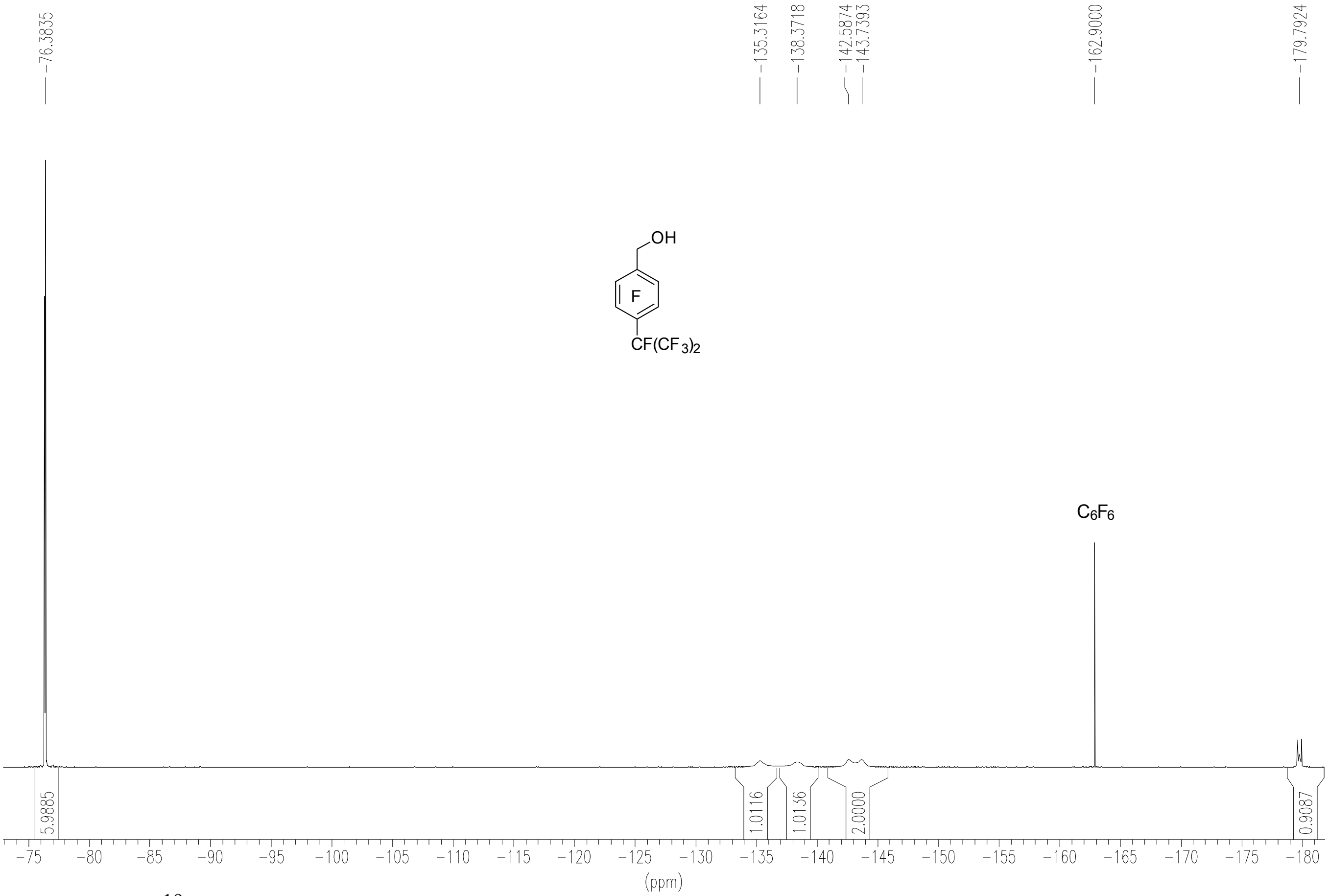

Figure S47. ^{19}F NMR spectrum of **14g** ($CDCl_3$).

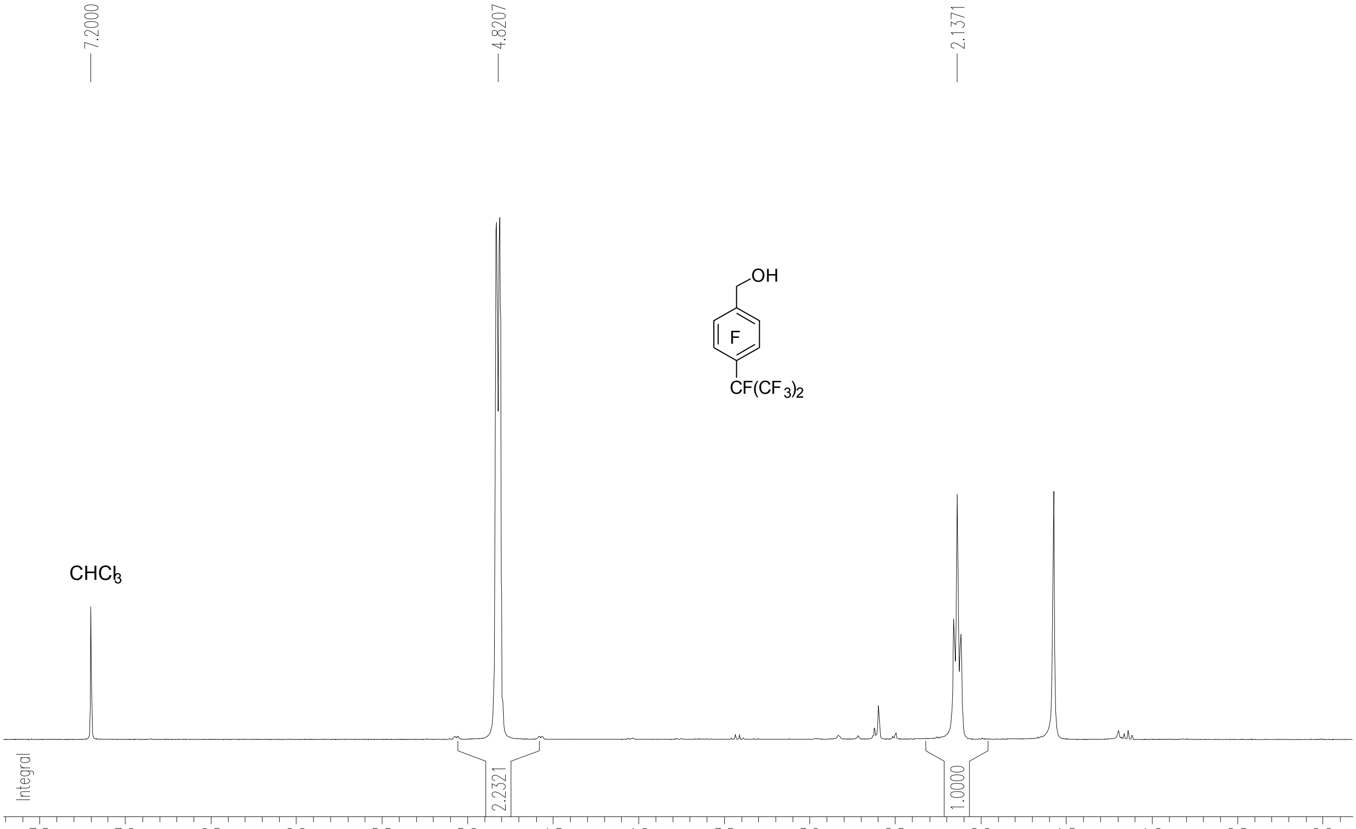

Figure S48. ^{1}H NMR spectrum of **14g** ($CDCl_3$).

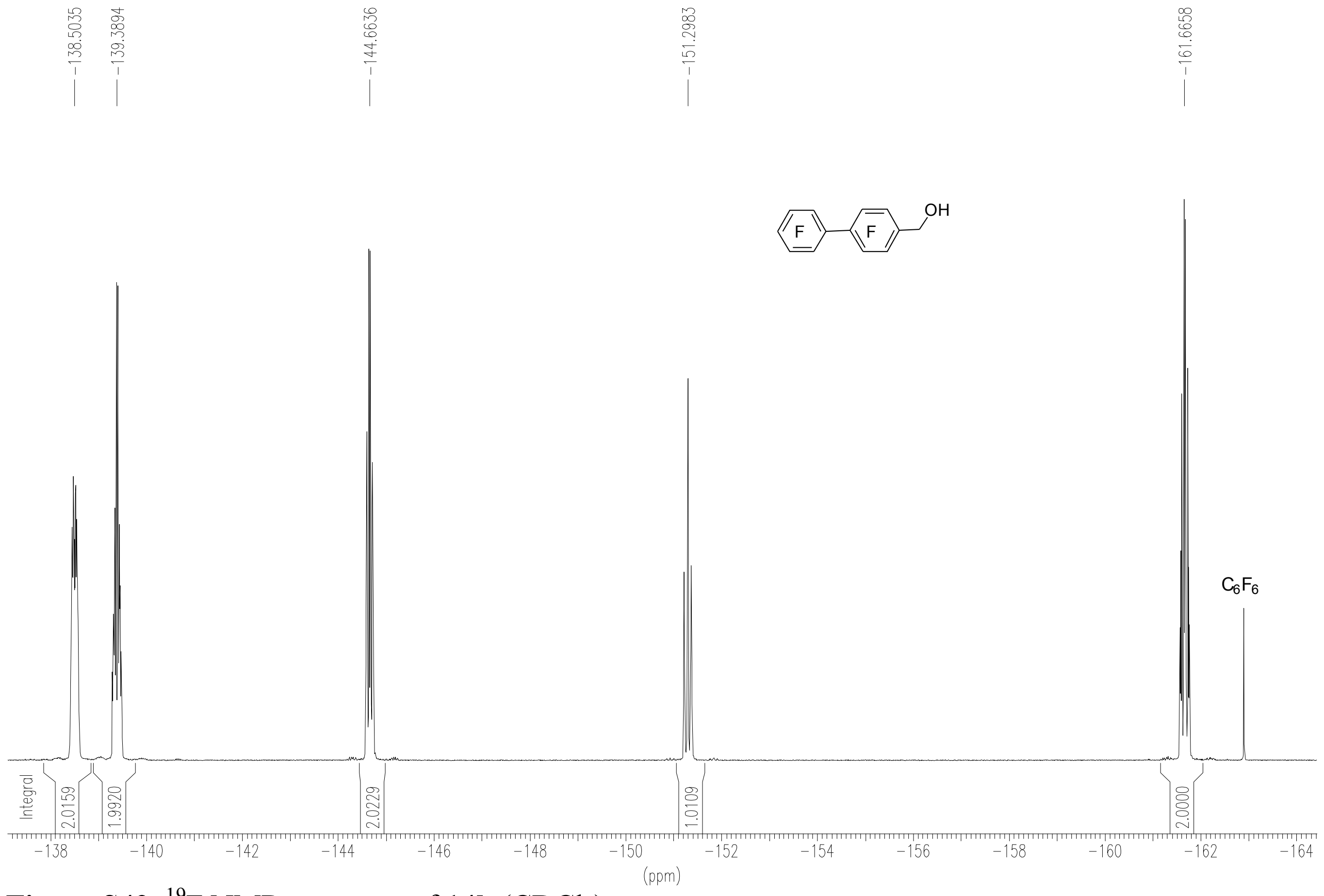

Figure S49. ^{19}F NMR spectrum of **14h** ($CDCl_3$).

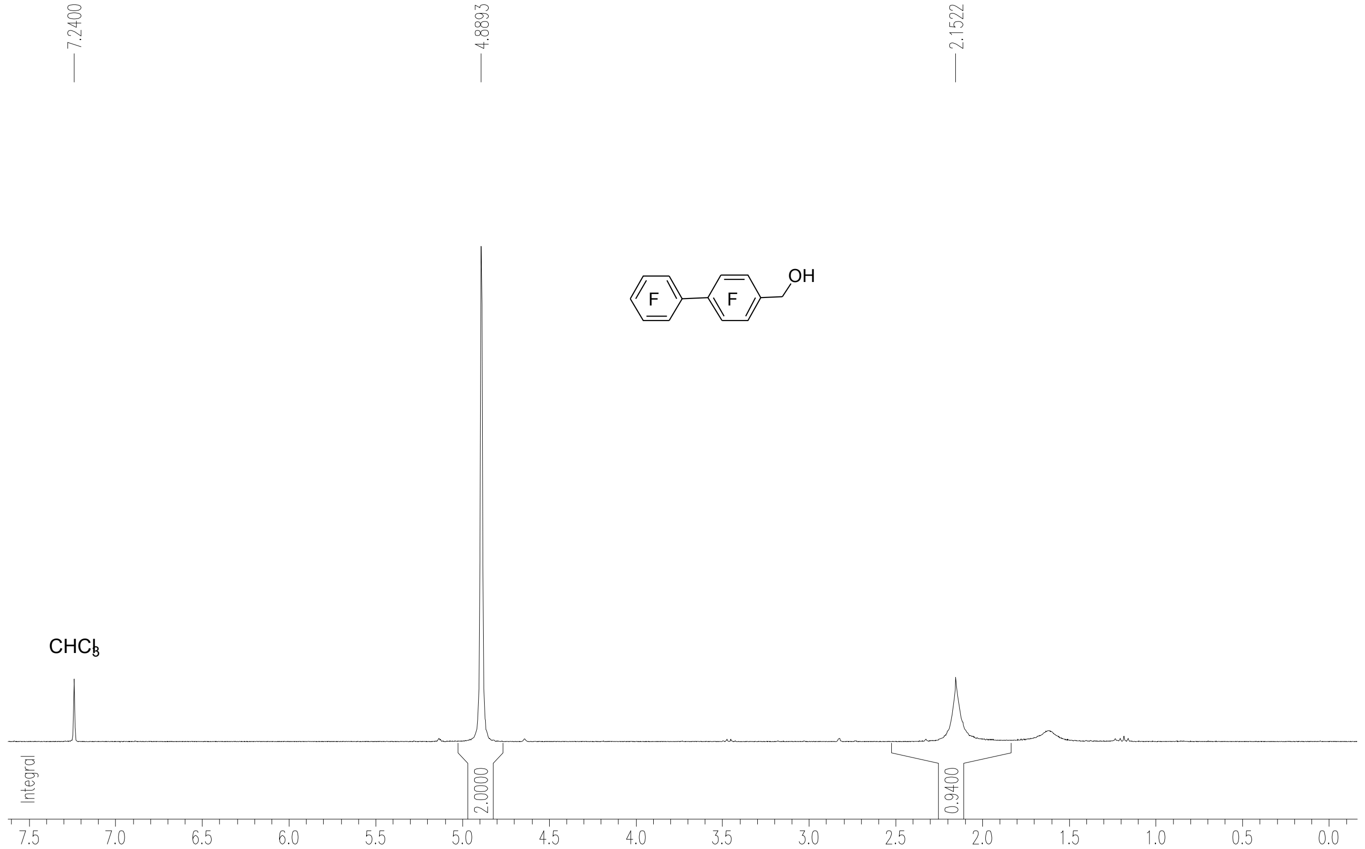

Figure S50. ^{1}H NMR spectrum of **14h** ($CDCl_3$).

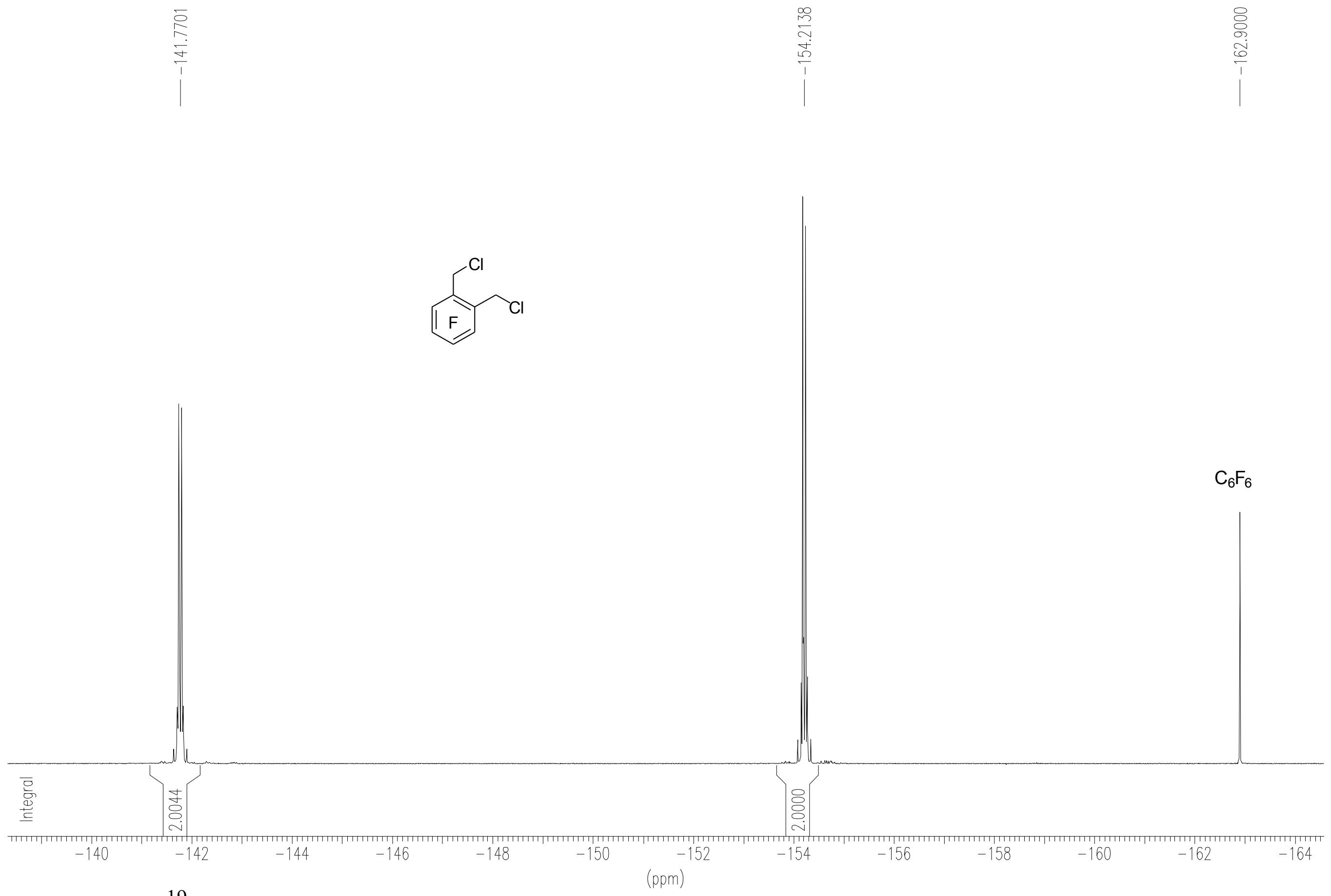

Figure S51. ^{19}F NMR spectrum of **16c** ($CDCl_3$).

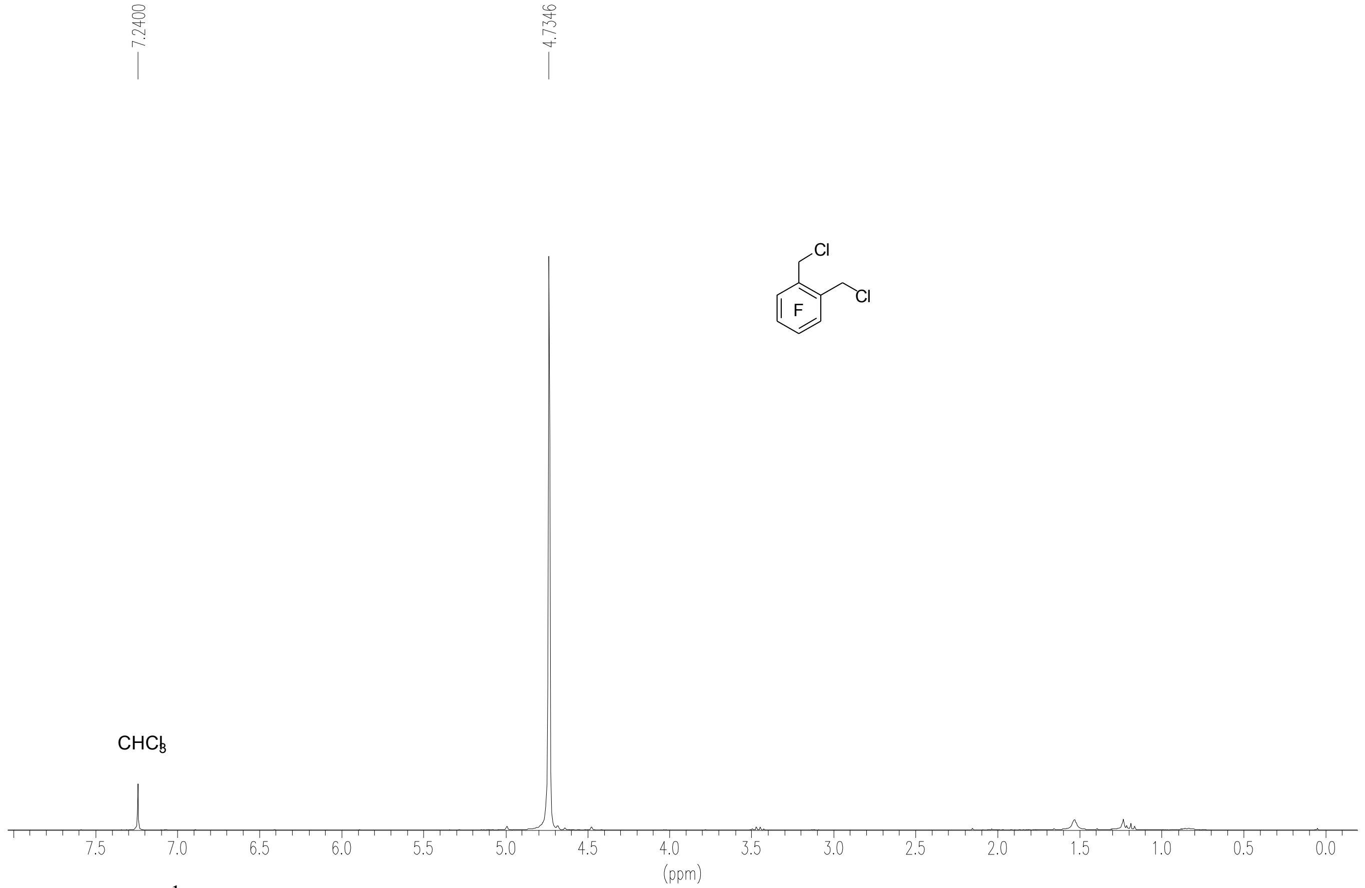

Figure S52. ^{1}H NMR spectrum of **16c** ($CDCl_3$).

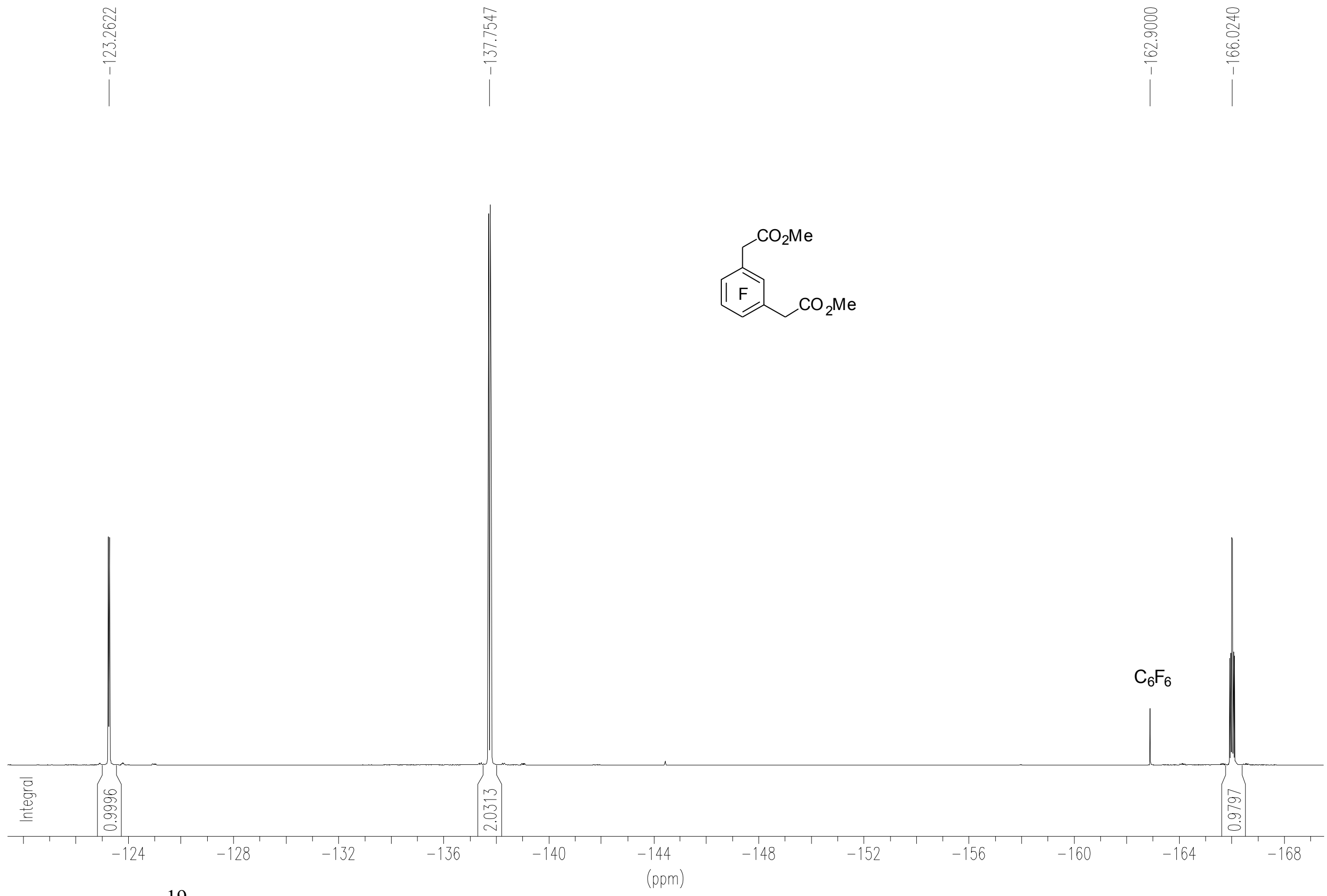

Figure S53. ^{19}F NMR spectrum of **17'b** ($CDCl_3$).

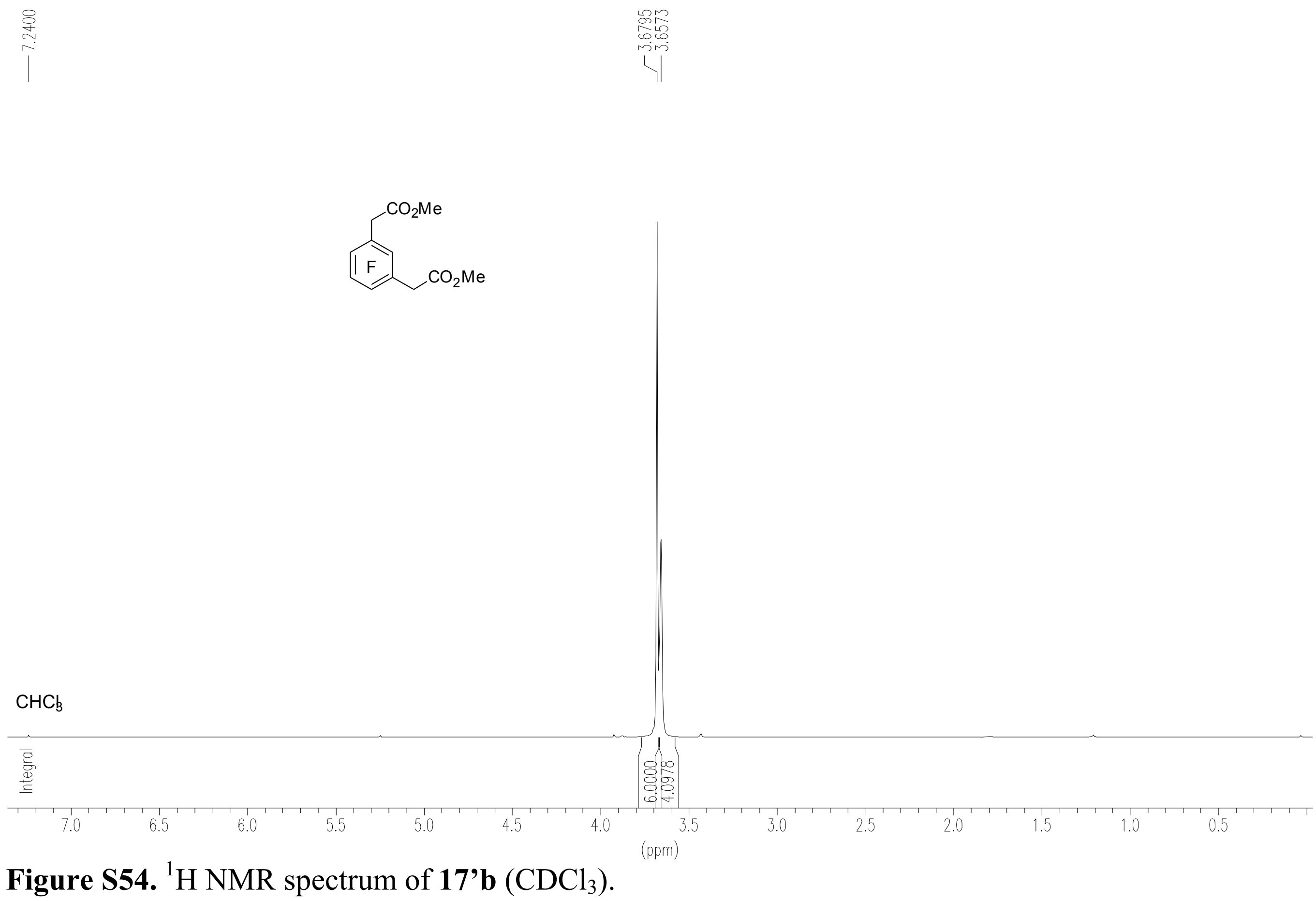

Figure S54. ^{1}H NMR spectrum of **17'b** ($CDCl_3$).

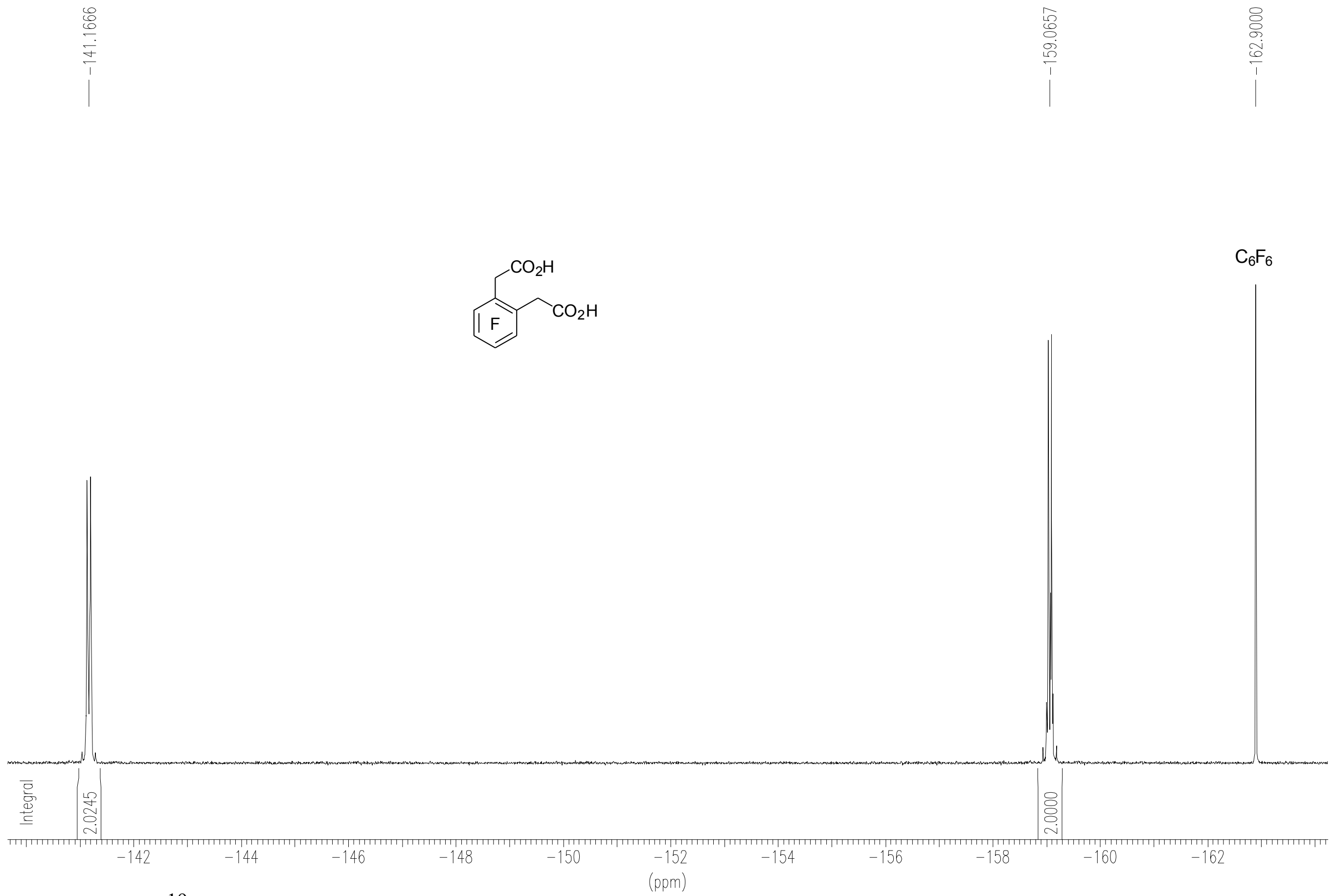

Figure S55. ^{19}F NMR spectrum of **17c** (acetone-d_6).

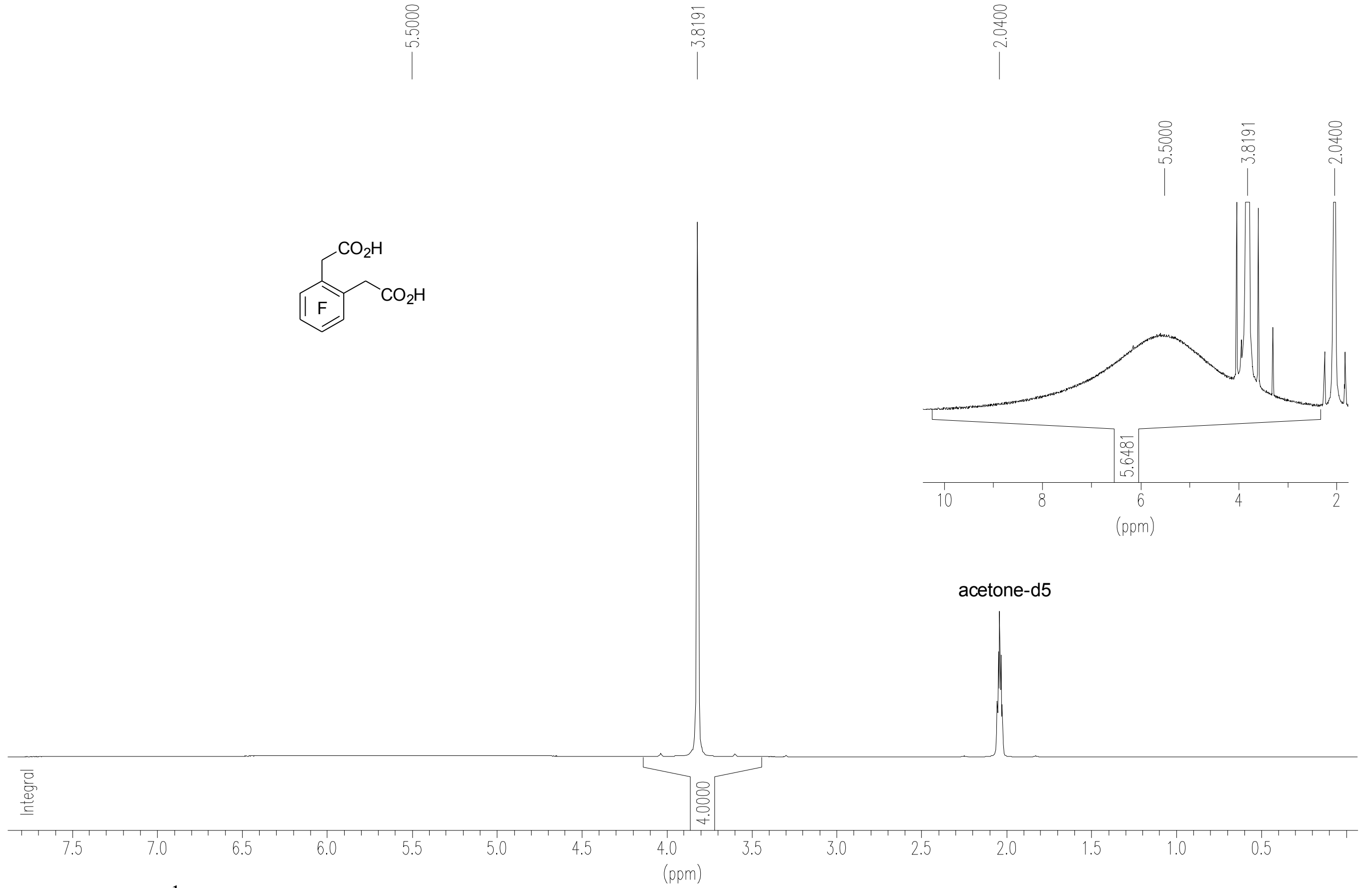

Figure S56. ^{1}H NMR spectrum of **17c** (acetone-d_6).

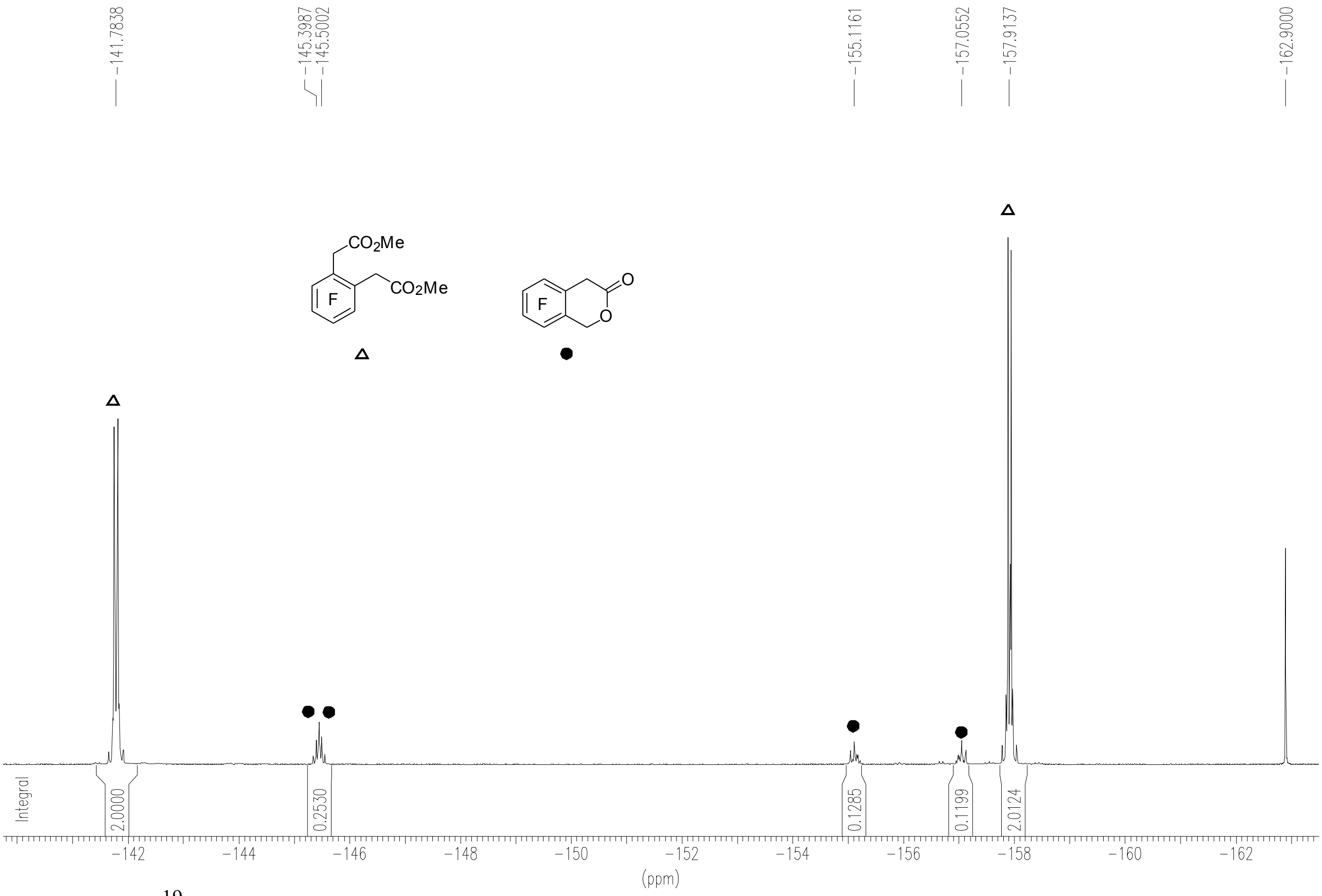

Figure S57. ^{19}F NMR spectrum of a mixture of **17'c** and **15** ($CDCl_3$).

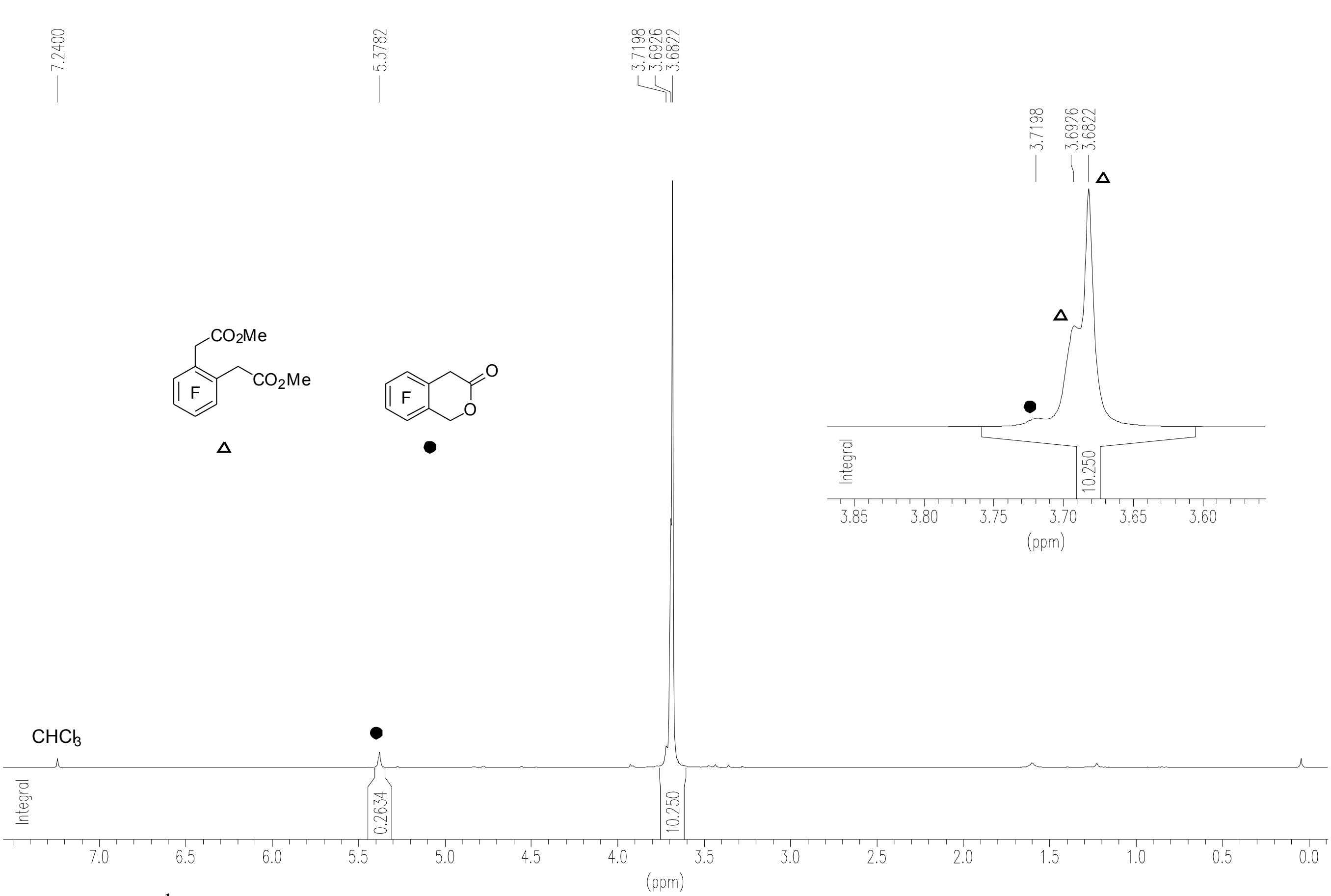

Figure S58. ^{1}H NMR spectrum of a mixture of **17'c** and **15** ($CDCl_3$).

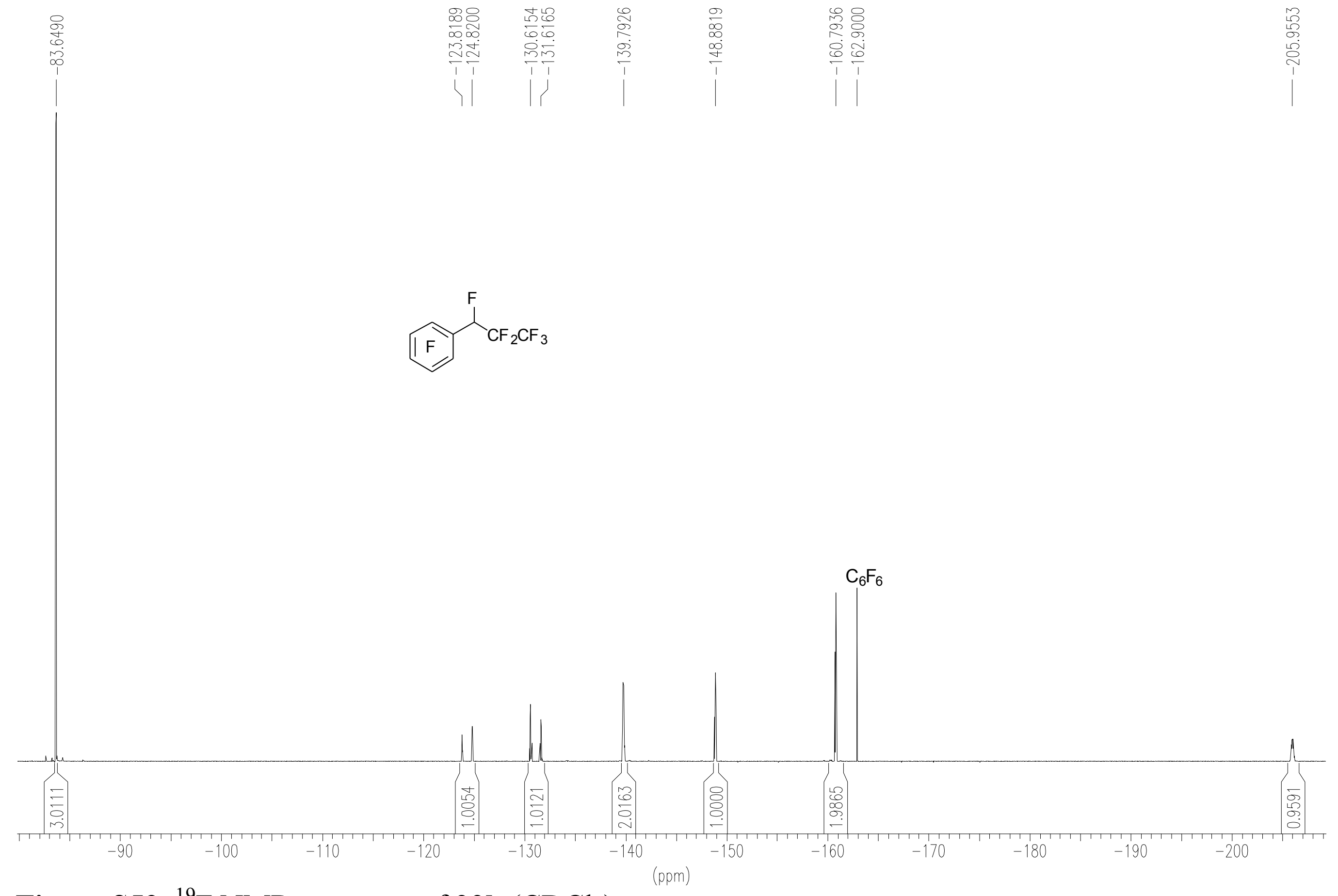

Figure S59. ^{19}F NMR spectrum of **22b** ($CDCl_3$).

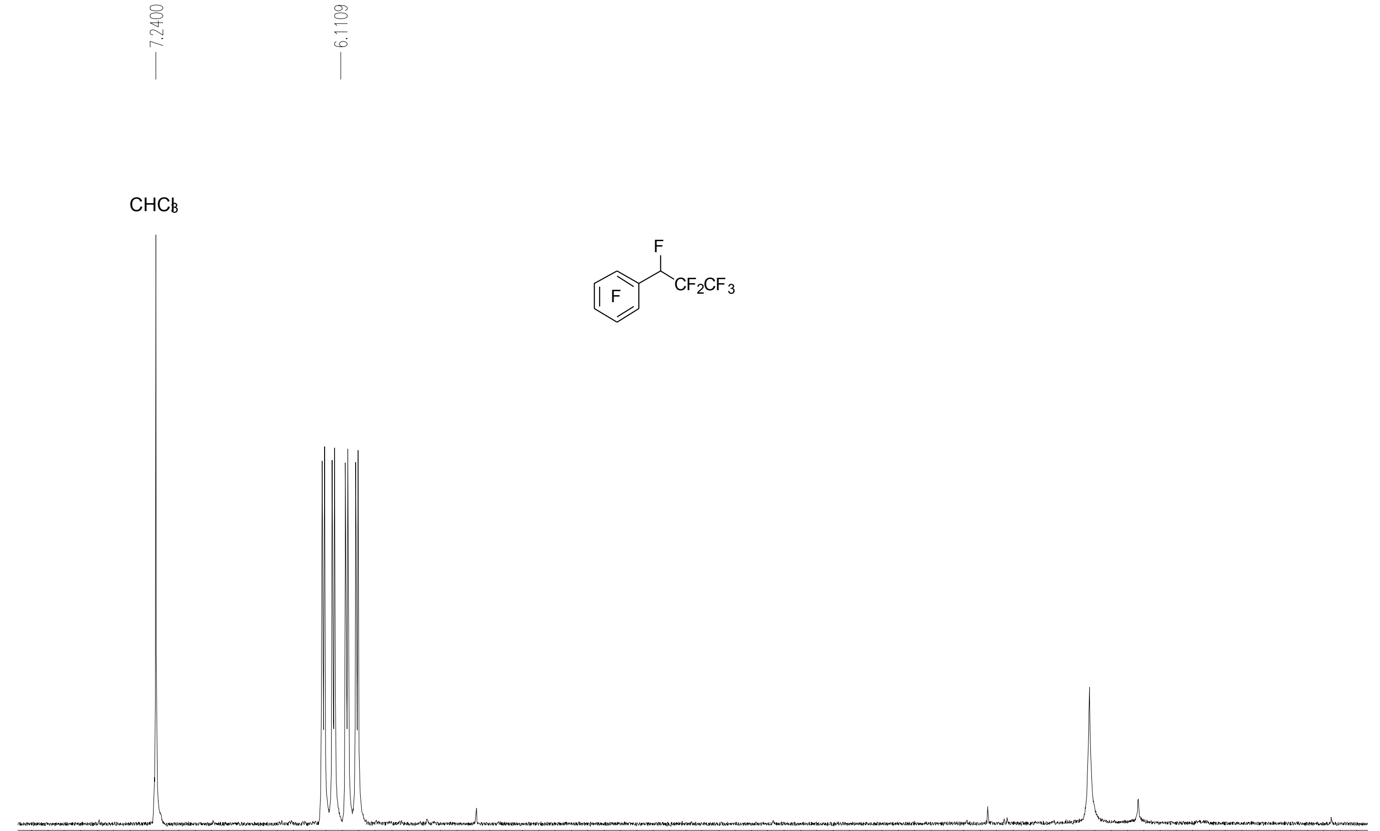

Figure S60. ^{1}H NMR spectrum of **22b** ($CDCl_3$).

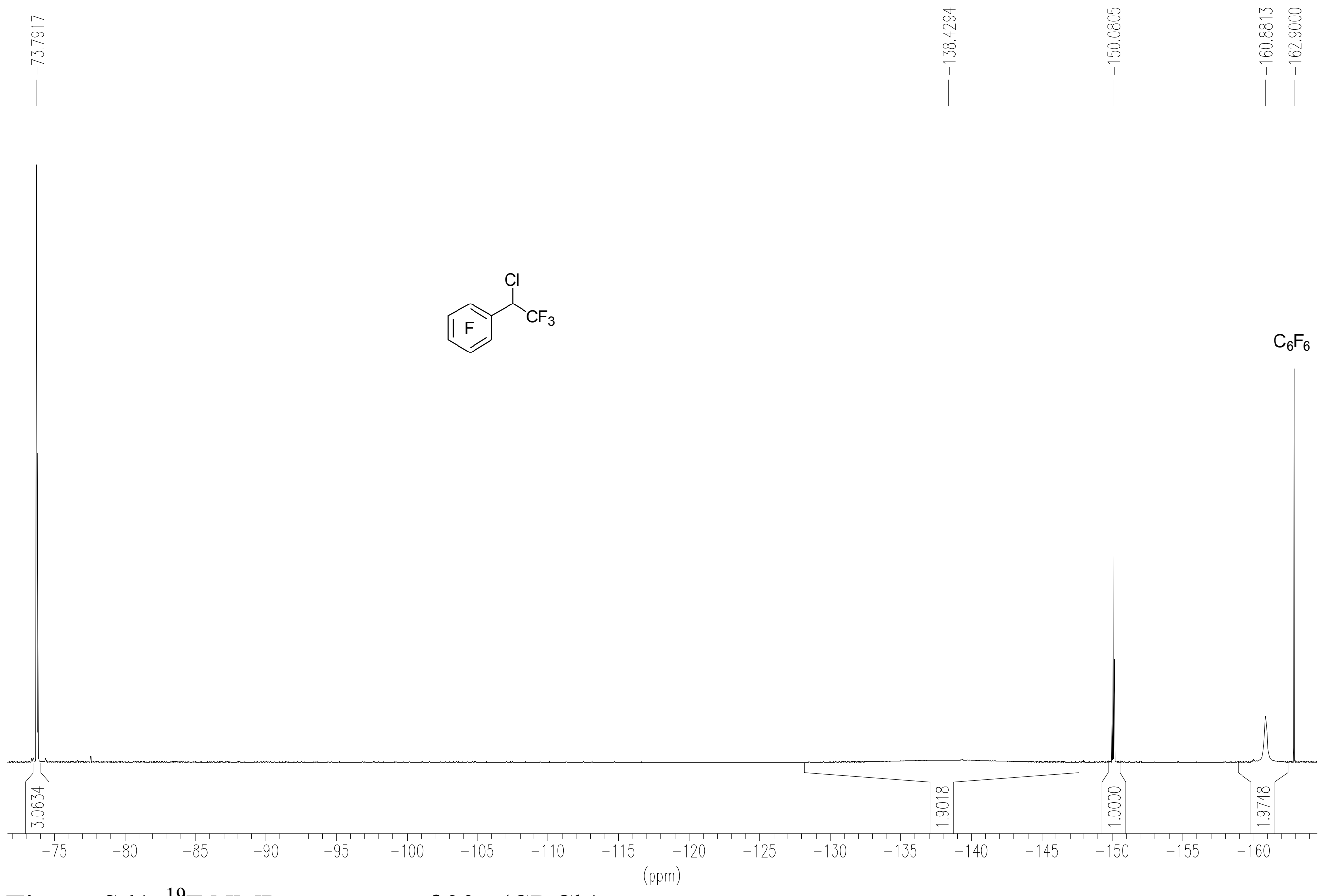

Figure S61. ^{19}F NMR spectrum of **23a** ($CDCl_3$).

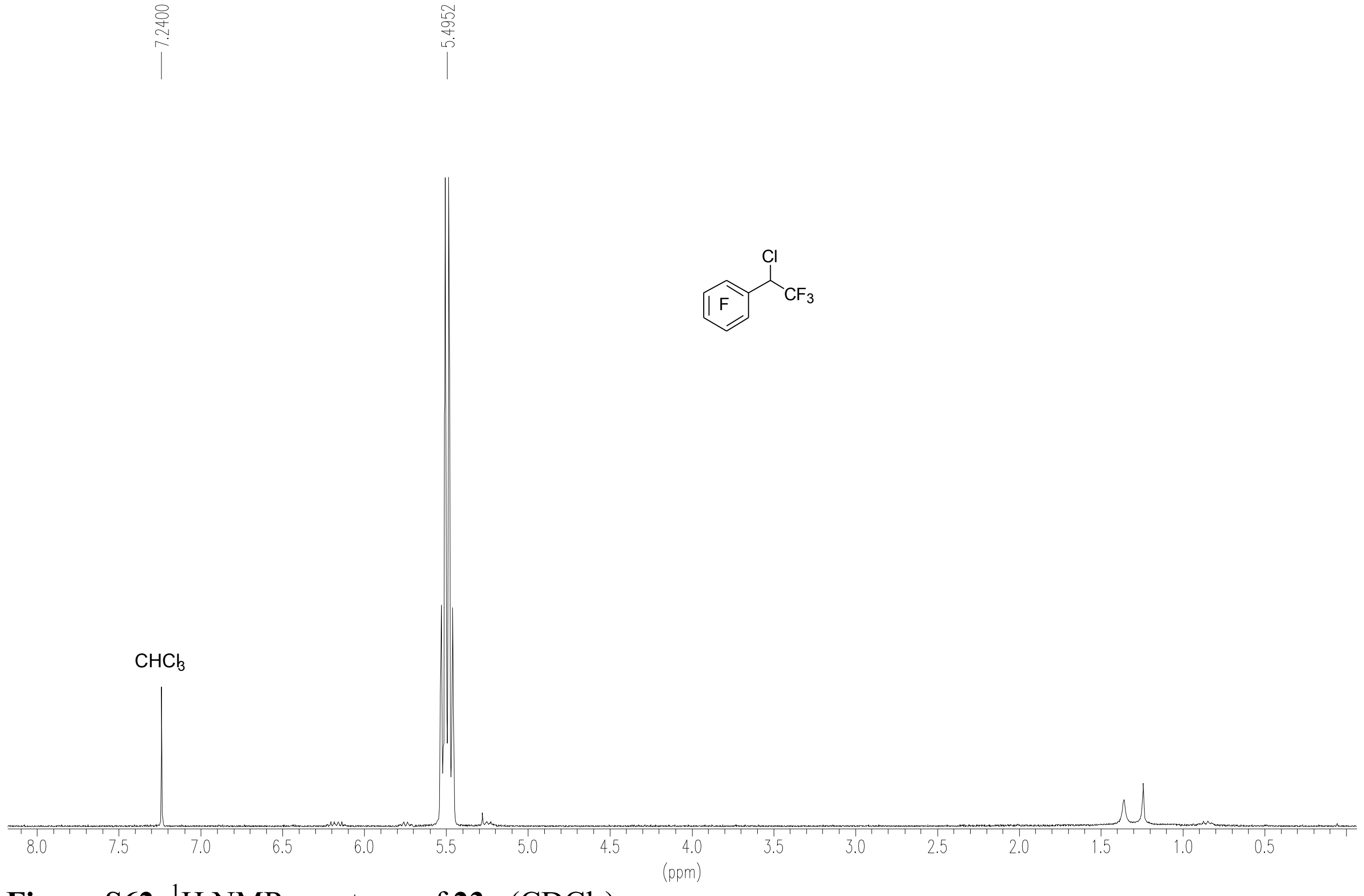

Figure S62. ^{1}H NMR spectrum of **23a** ($CDCl_3$).

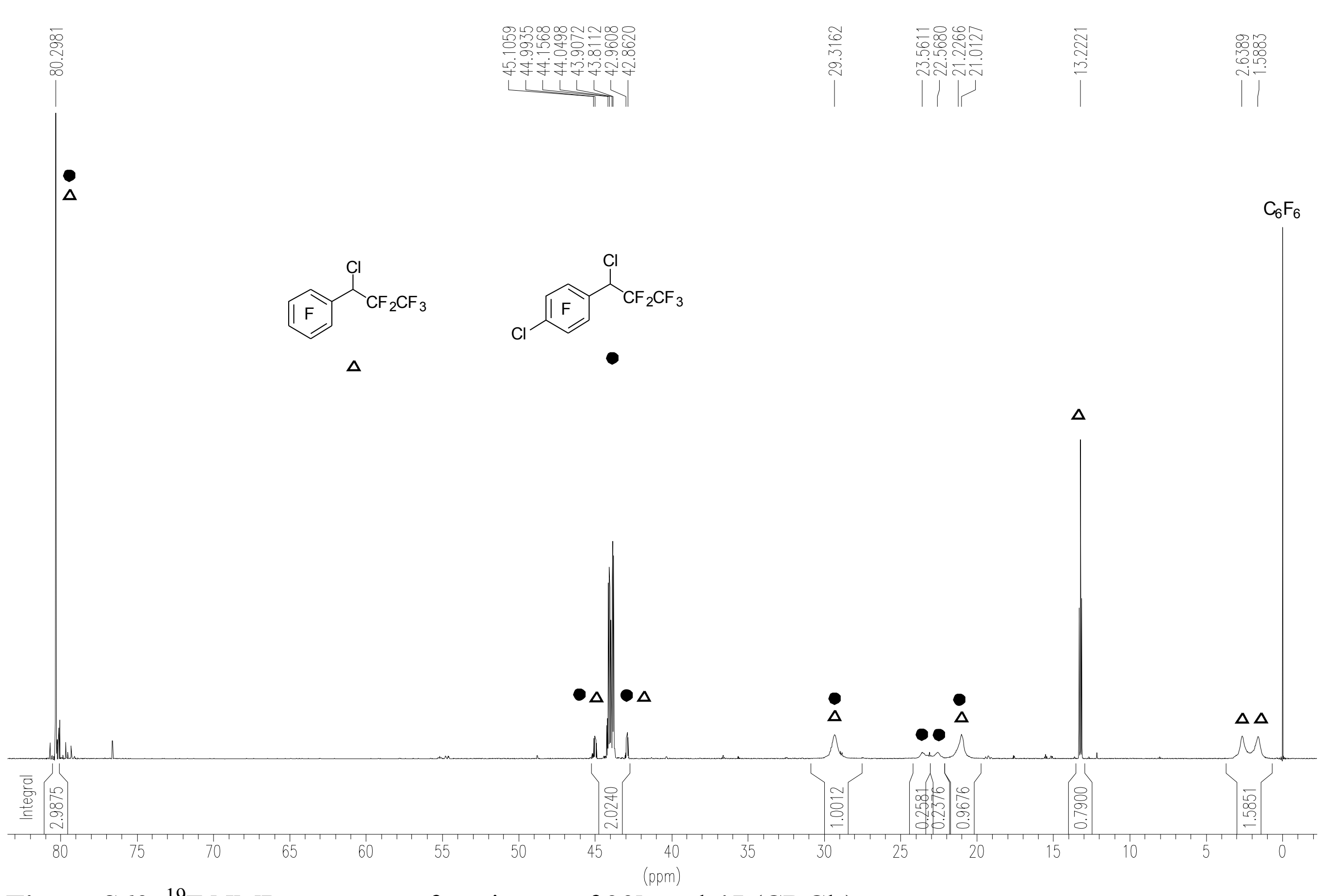

Figure S63. ^{19}F NMR spectrum of a mixture of **23b** and **45** ($CDCl_3$).

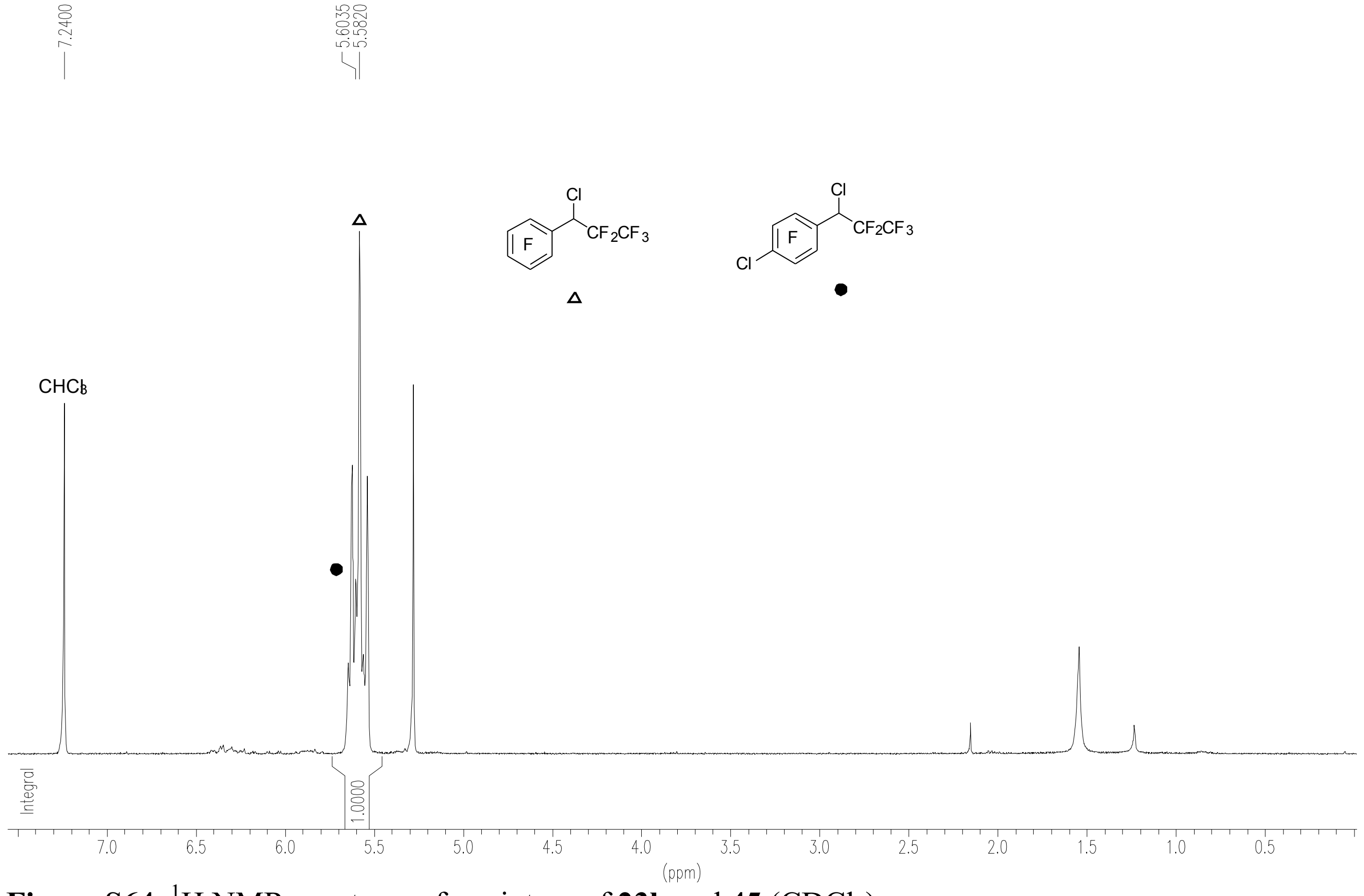

Figure S64. ^{1}H NMR spectrum of a mixture of **23b** and **45** ($CDCl_3$).

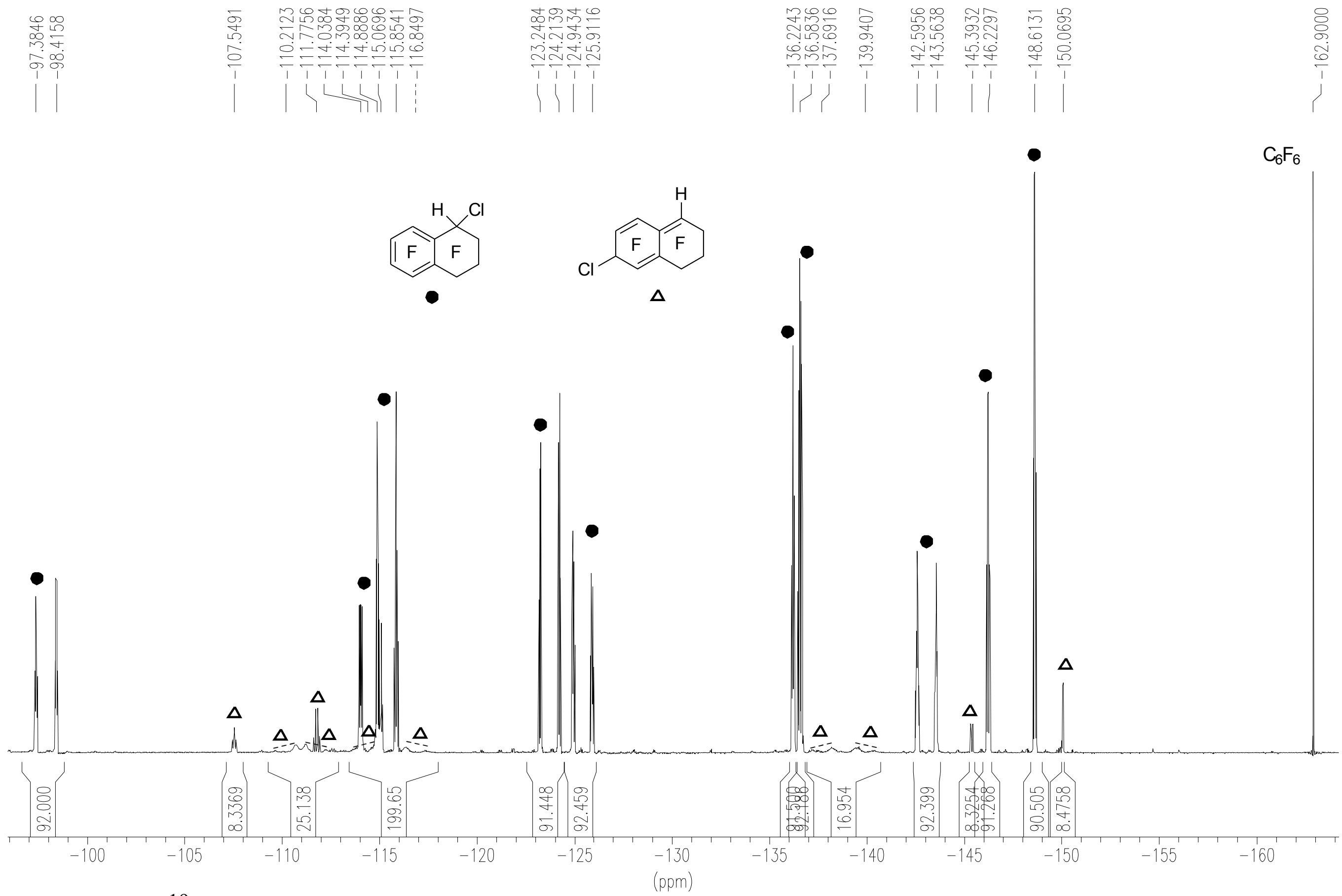

Figure S65. ^{19}F NMR spectrum of a mixture of **23c** and **47** ($CDCl_3$).

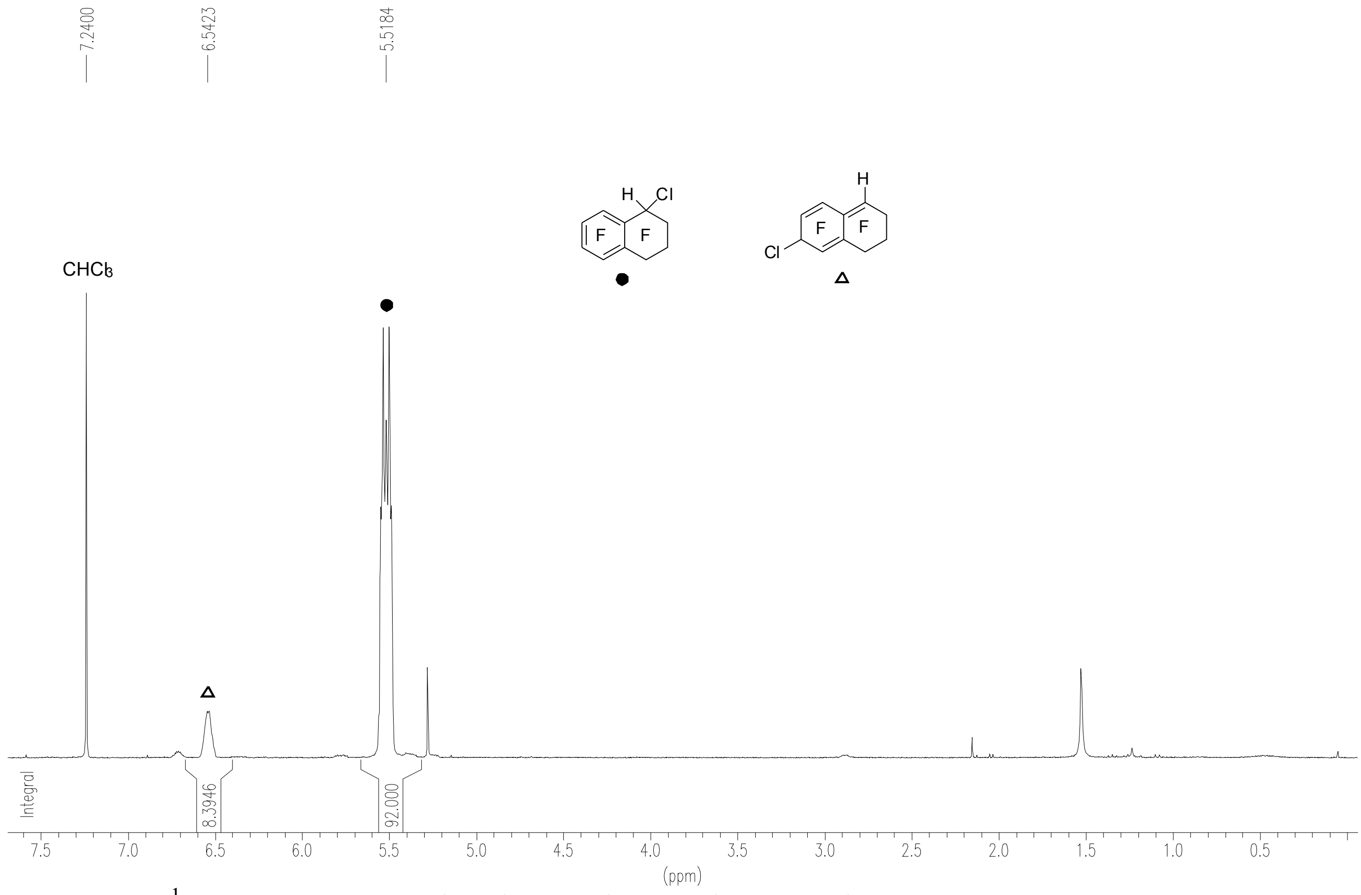

Figure S66. ^{1}H NMR spectrum of a mixture of **23c** and **47** ($CDCl_3$).

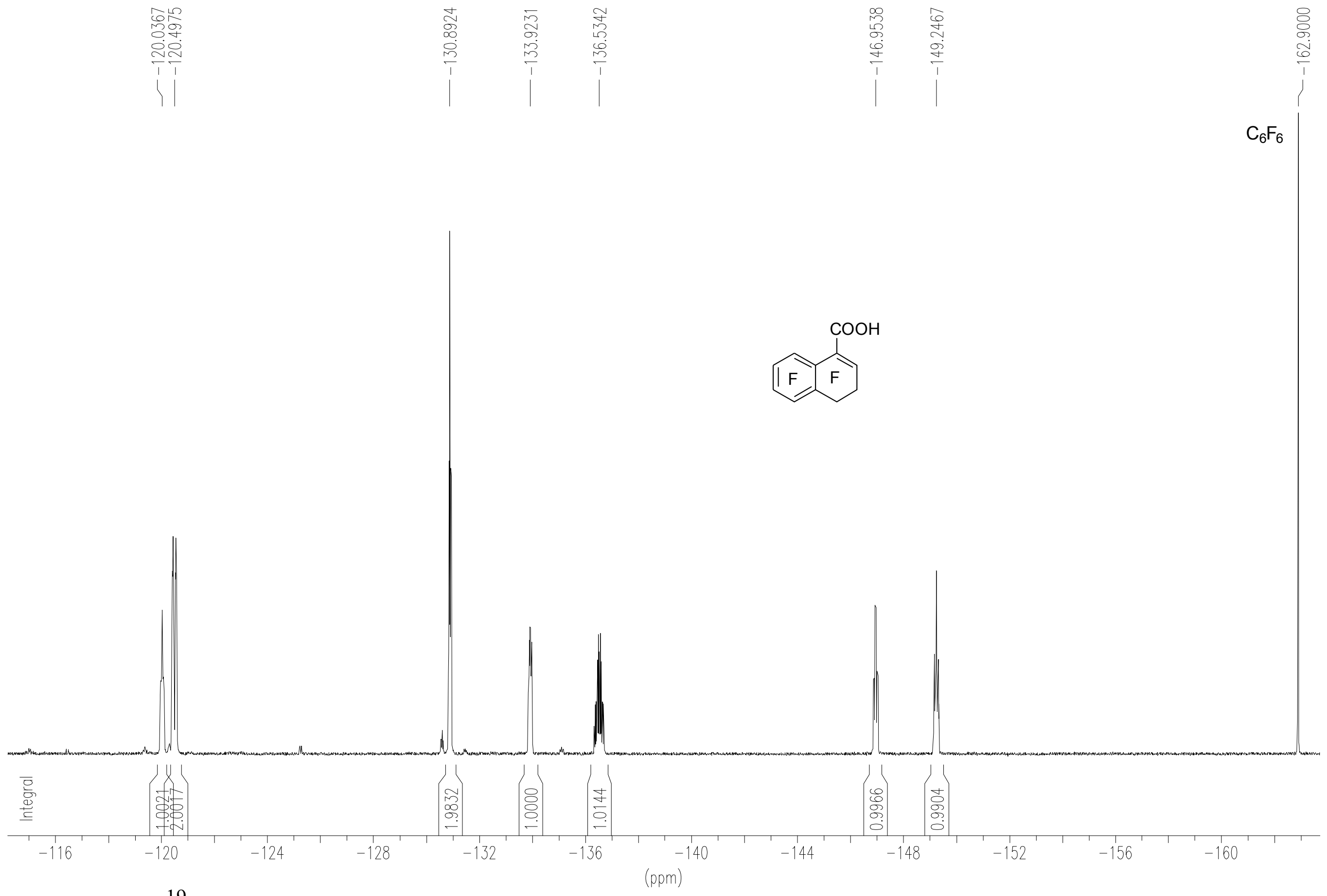

Figure S67. ^{19}F NMR spectrum of **25c** ($CDCl_3$).

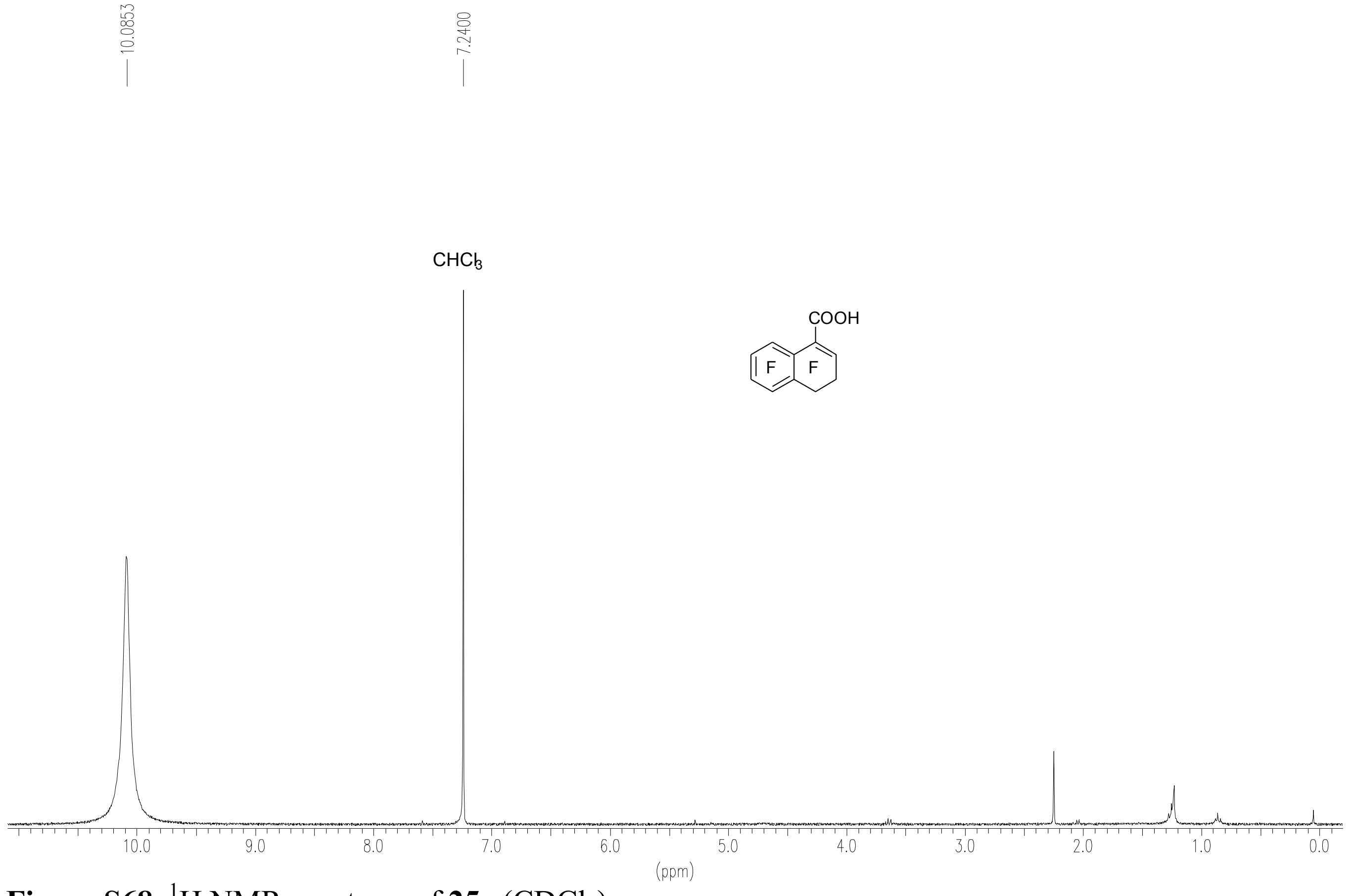

Figure S68. ^{1}H NMR spectrum of **25c** ($CDCl_3$).

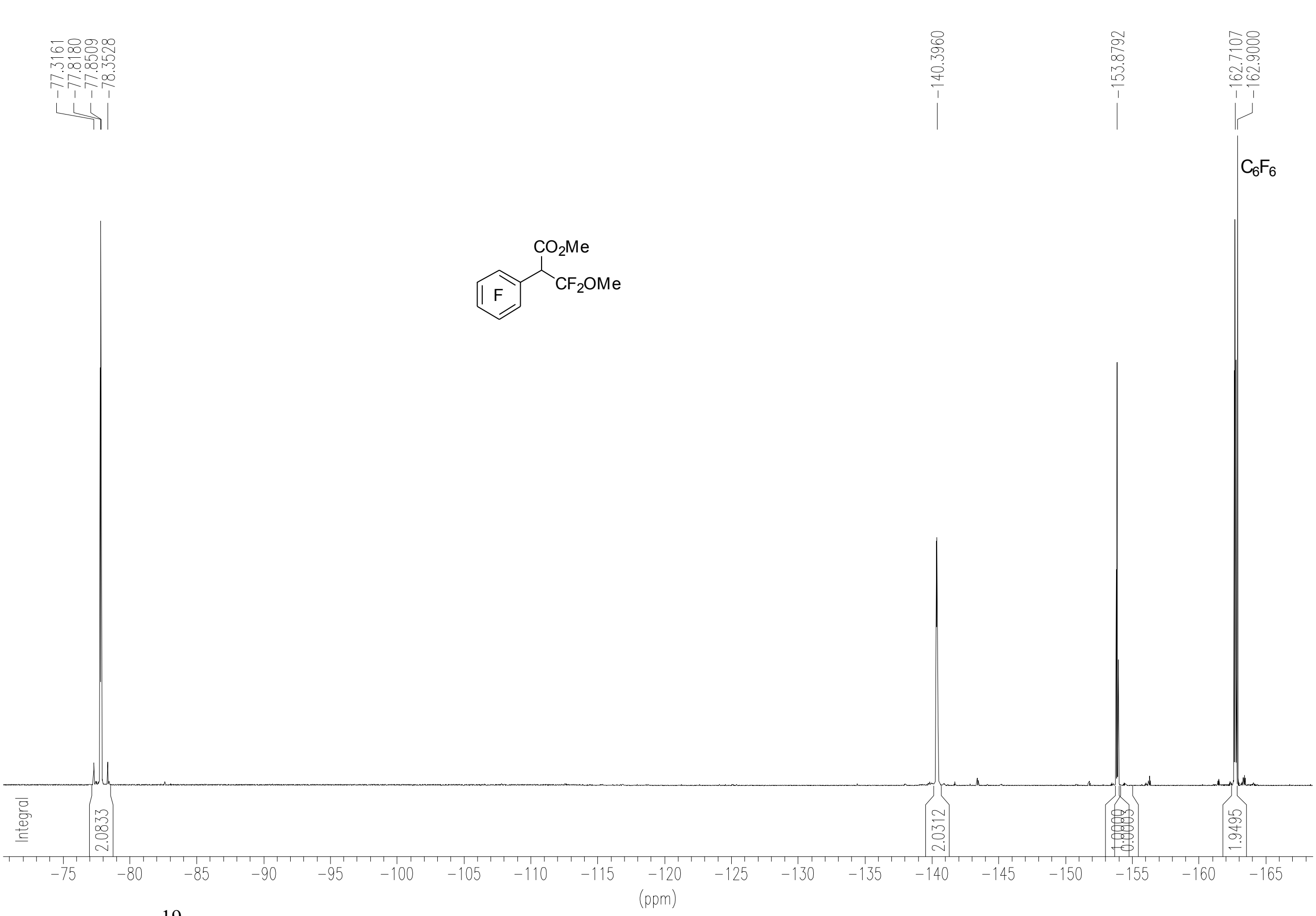

Figure S69. ^{19}F NMR spectrum of **28’** ($CDCl_3$).

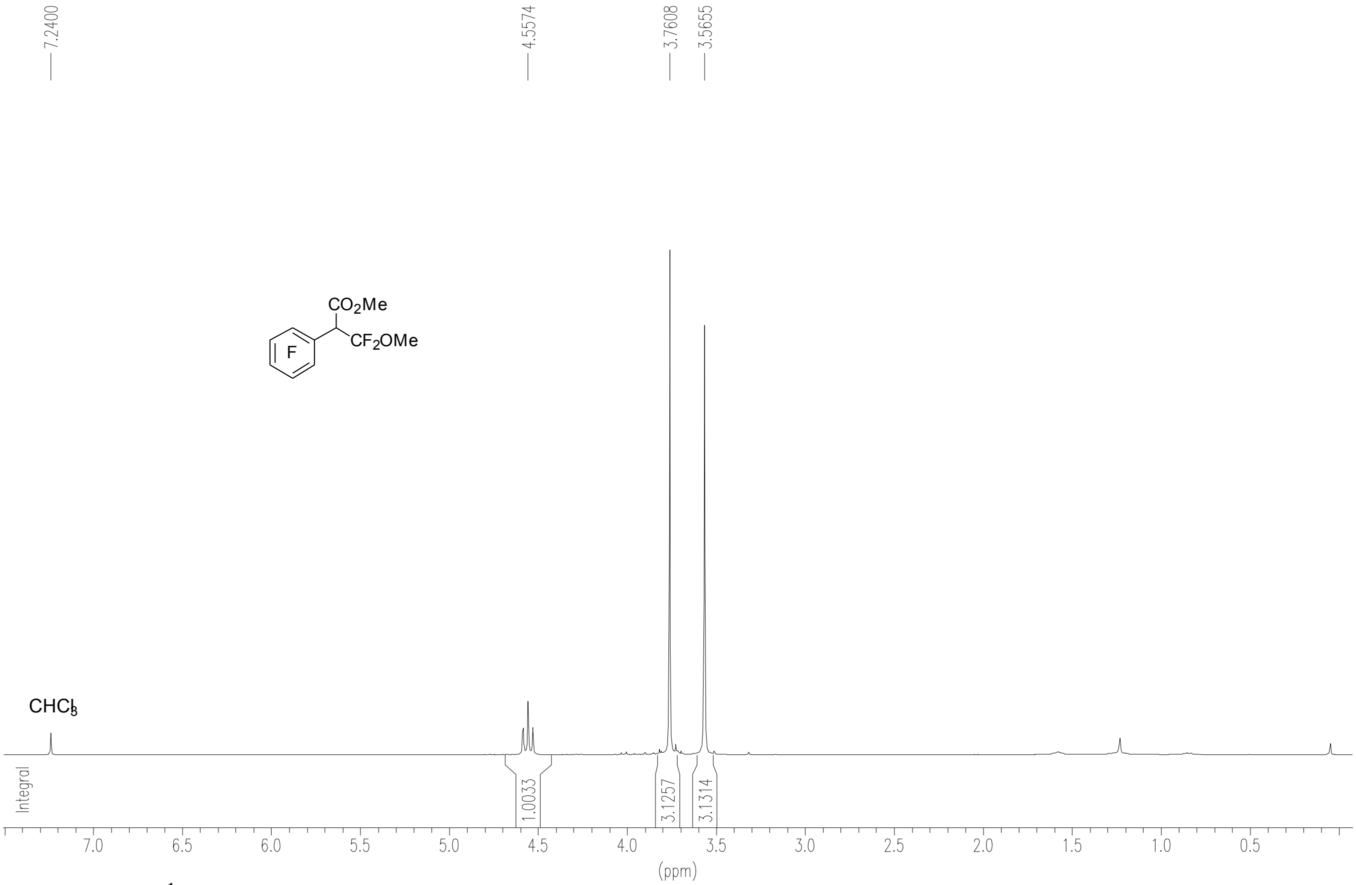

Figure S70. ^{1}H NMR spectrum of **28’** ($CDCl_3$).

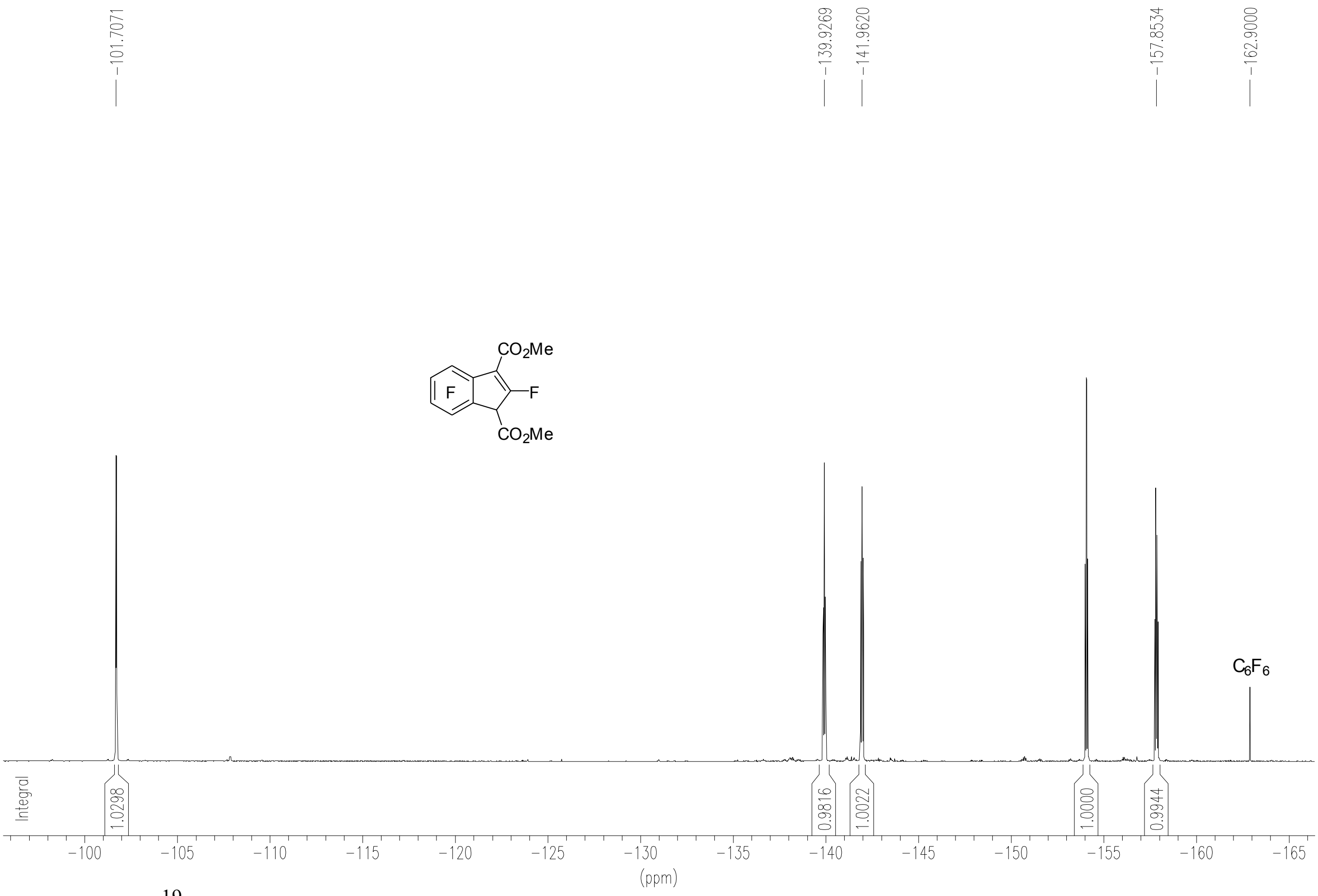

Figure S71. ^{19}F NMR spectrum of **29’** ($CDCl_3$).

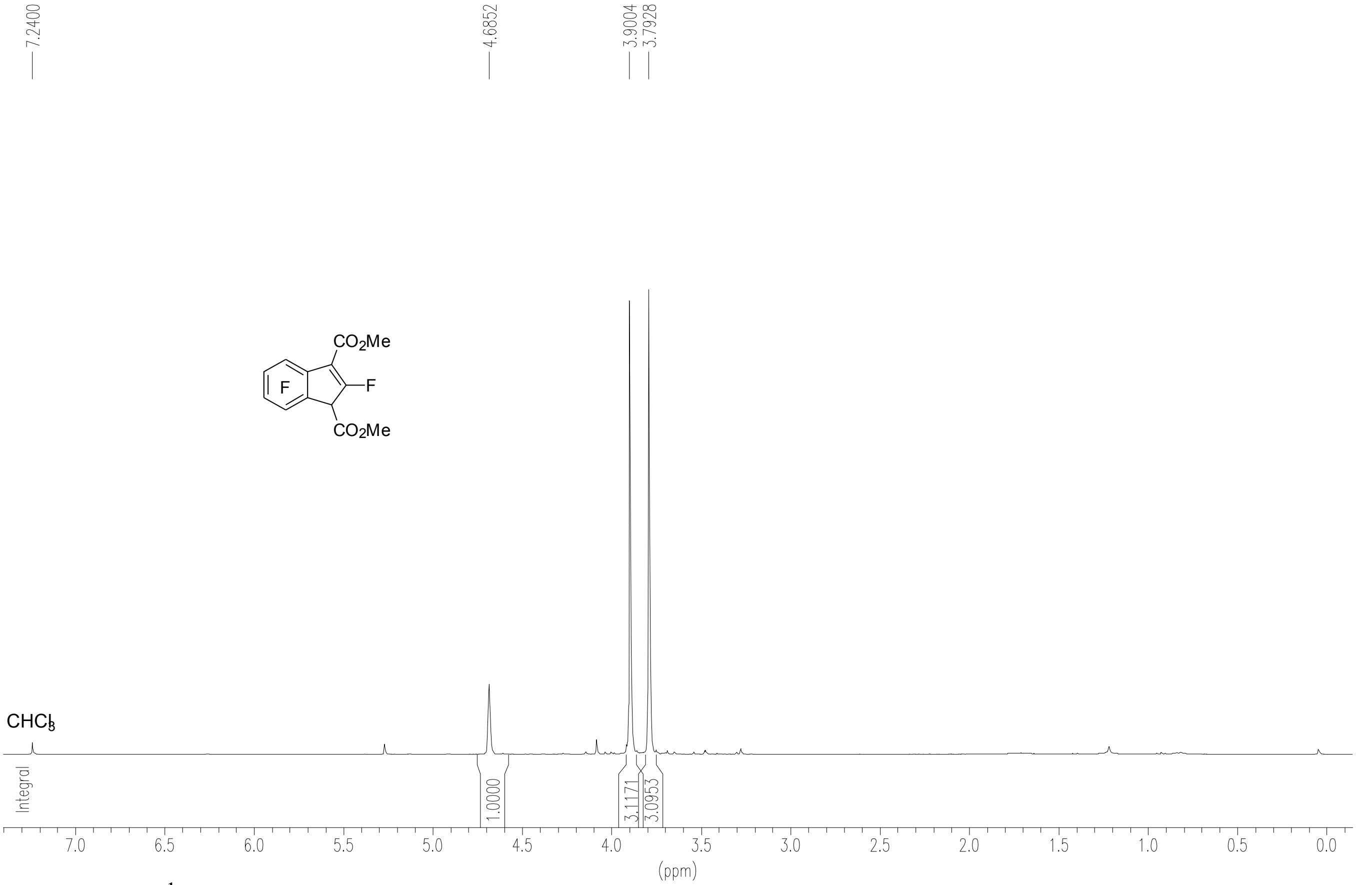

Figure S72. ^{1}H NMR spectrum of **29’** ($CDCl_3$).

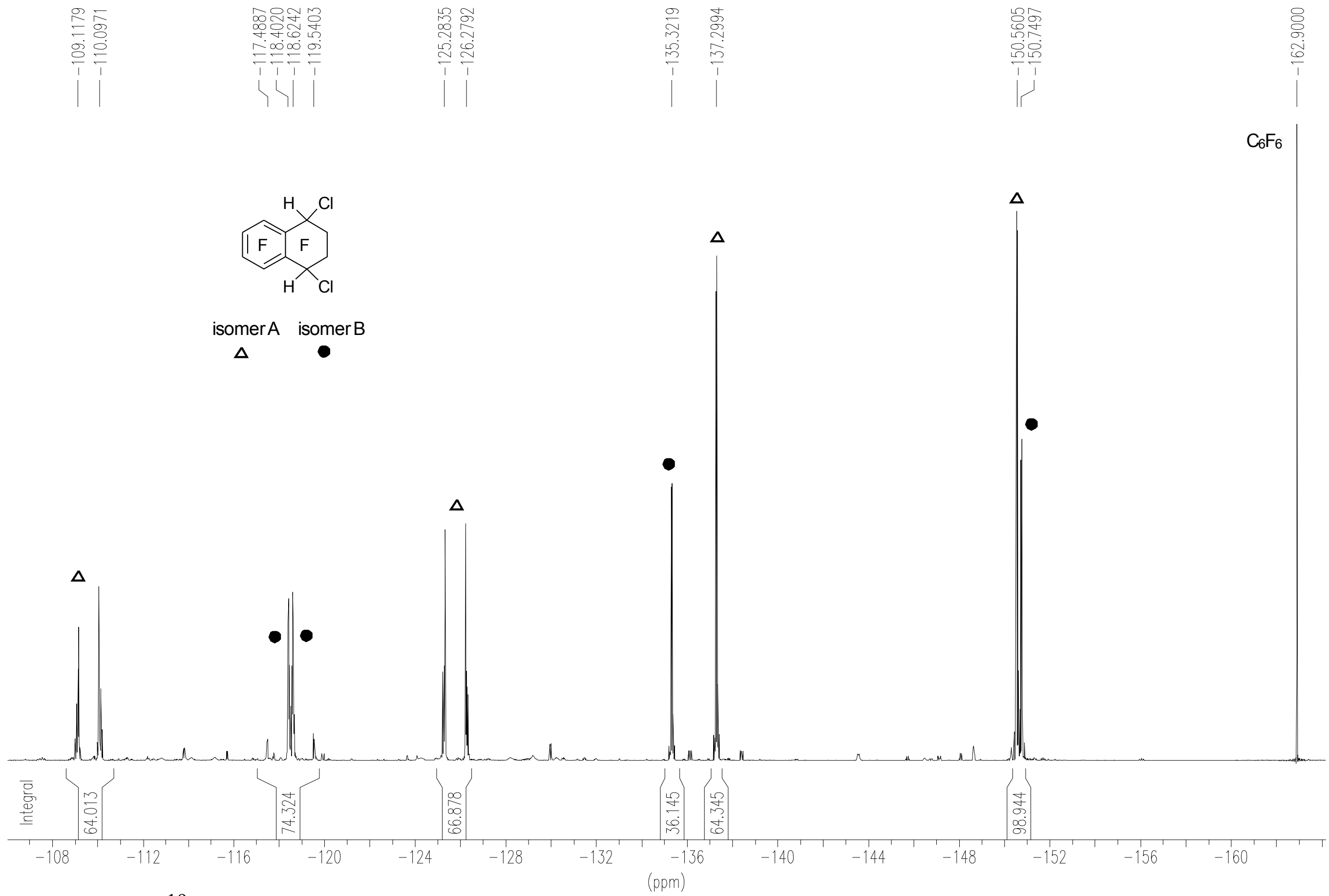

Figure S73. ^{19}F NMR spectrum of **36** ($CDCl_3$).

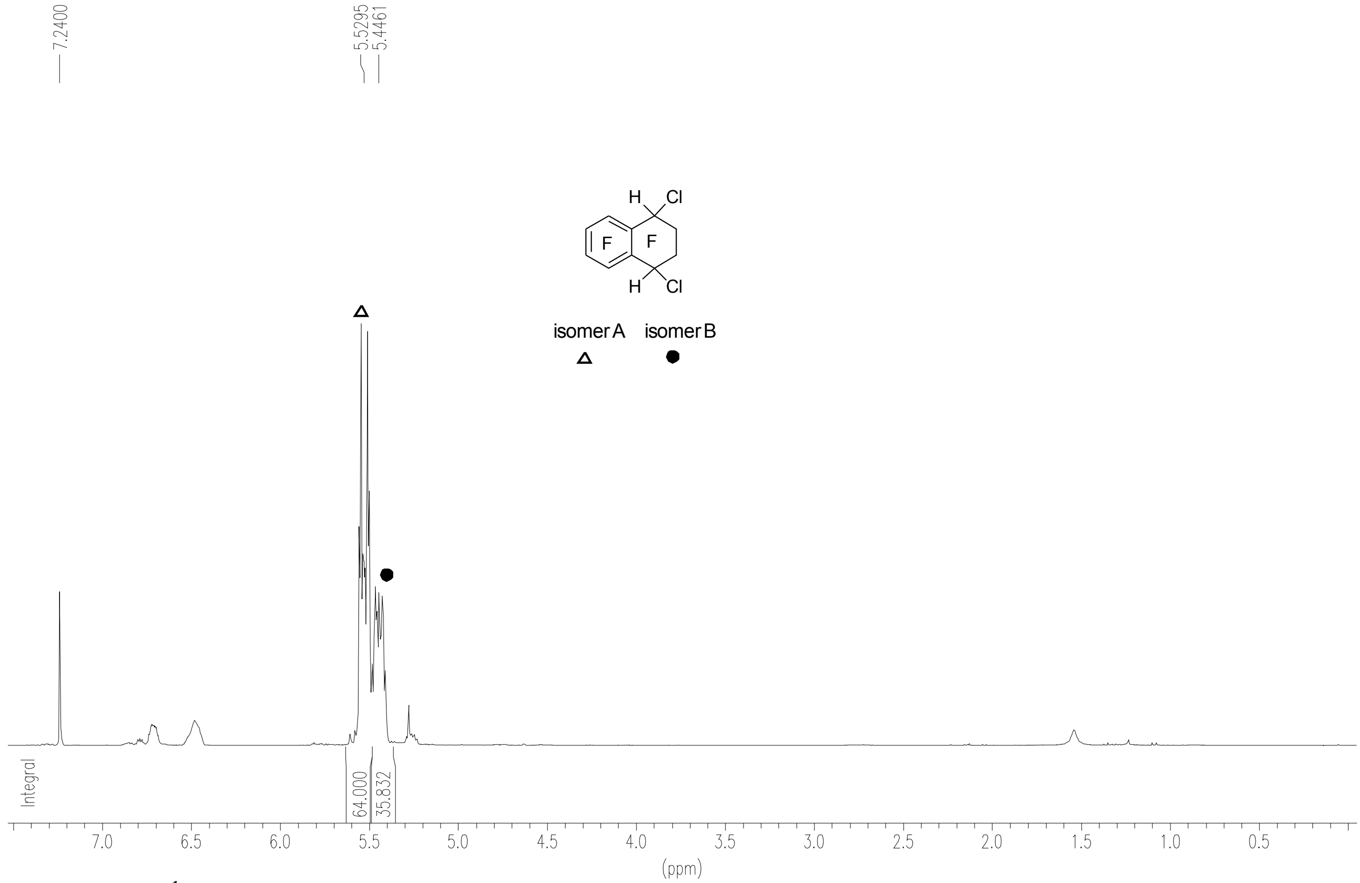

Figure S74. ^{1}H NMR spectrum of **36** ($CDCl_3$).

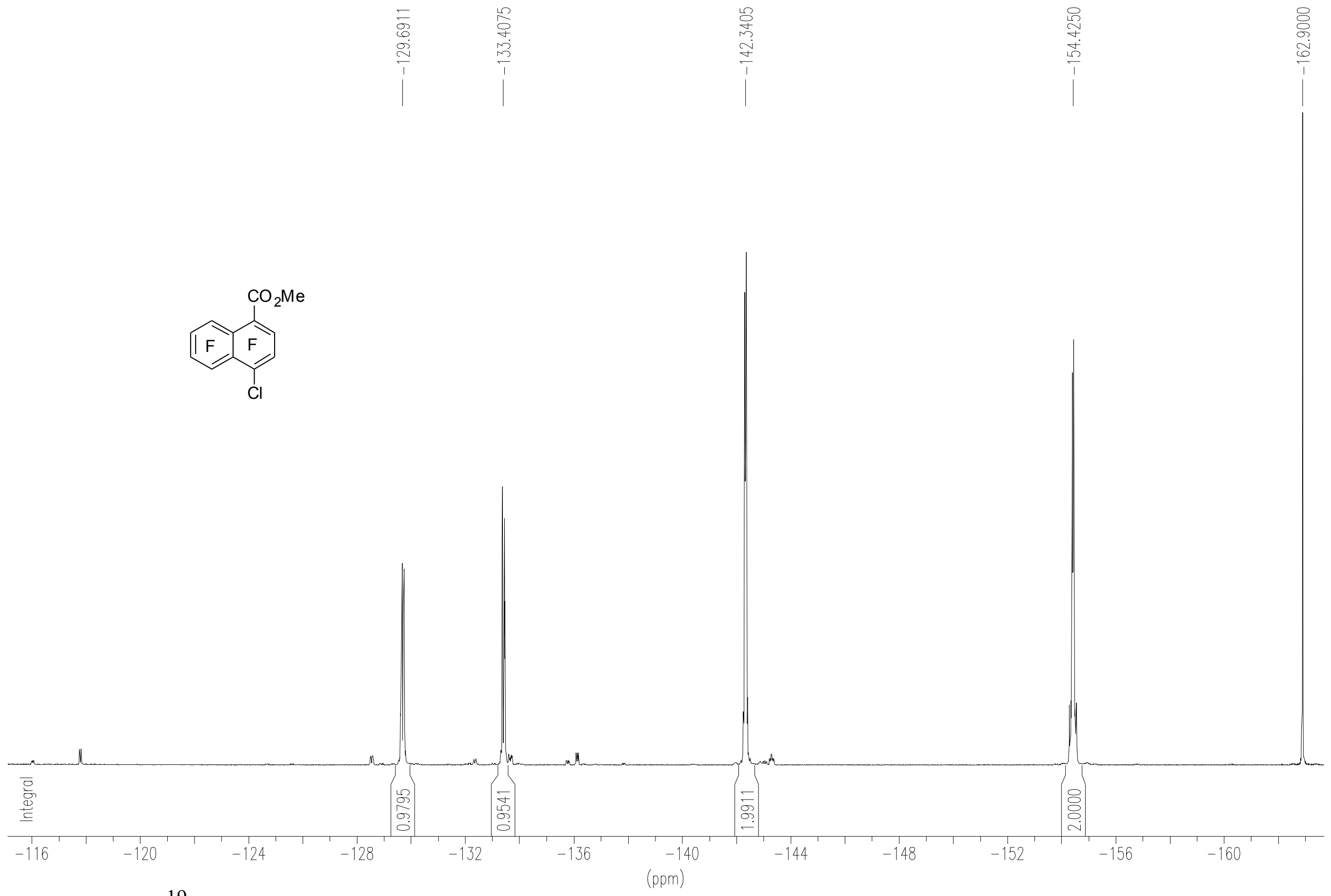

Figure S75. ^{19}F NMR spectrum of **38’** ($CDCl_3$).

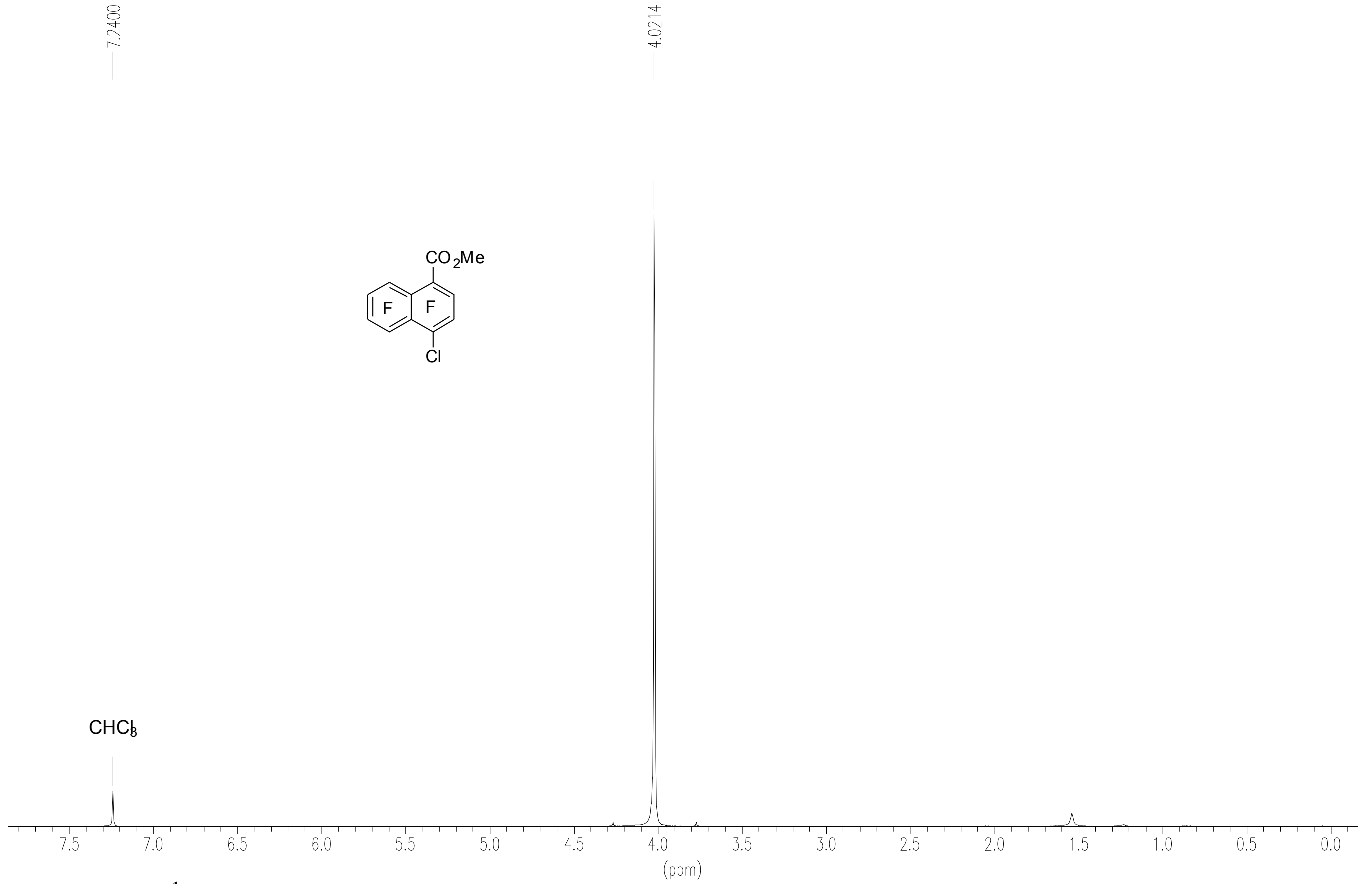

Figure S76. ^{1}H NMR spectrum of **38’** ($CDCl_3$).

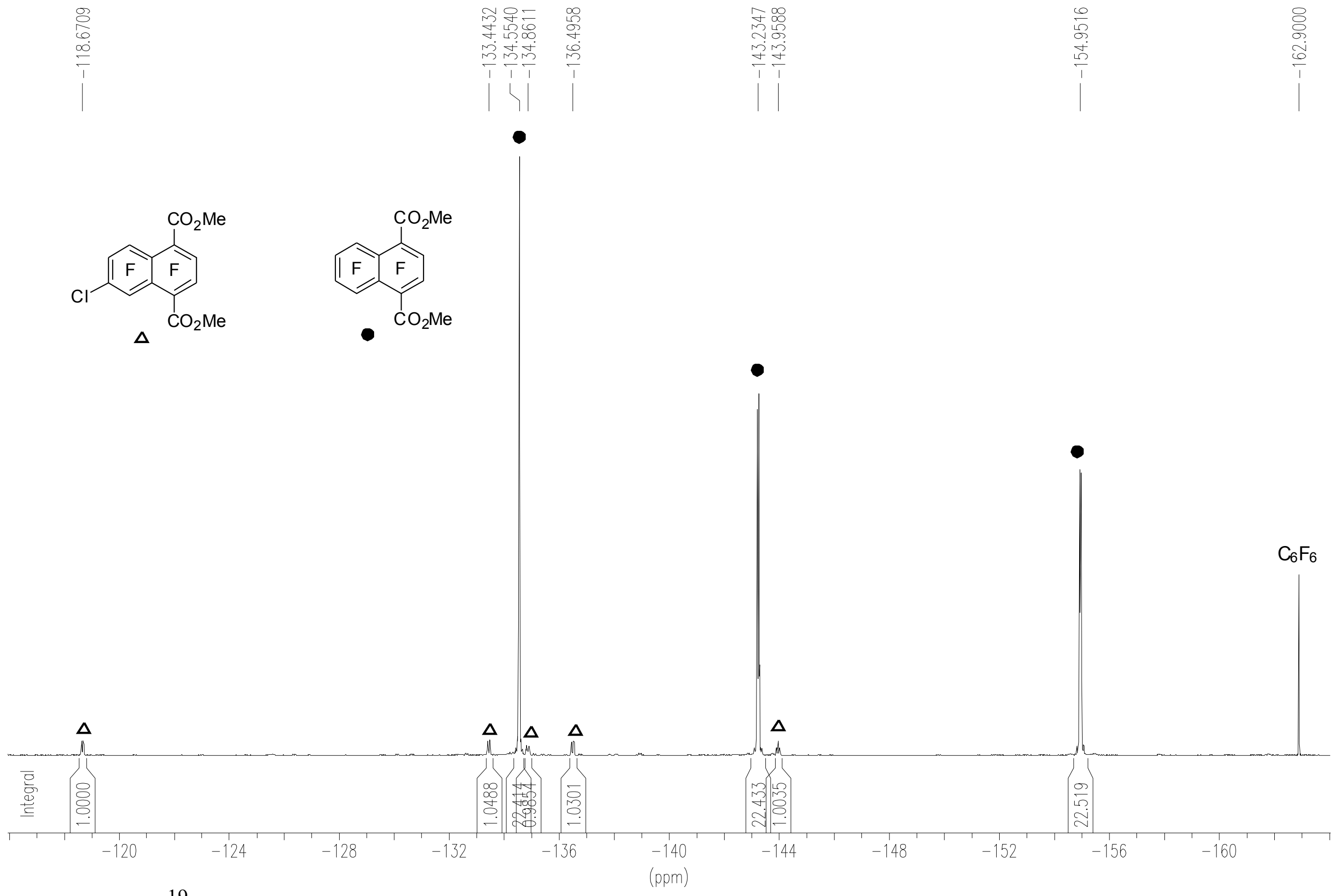

Figure S77. ^{19}F NMR spectrum of a mixture of **53’** and **39’** ($CDCl_3$).

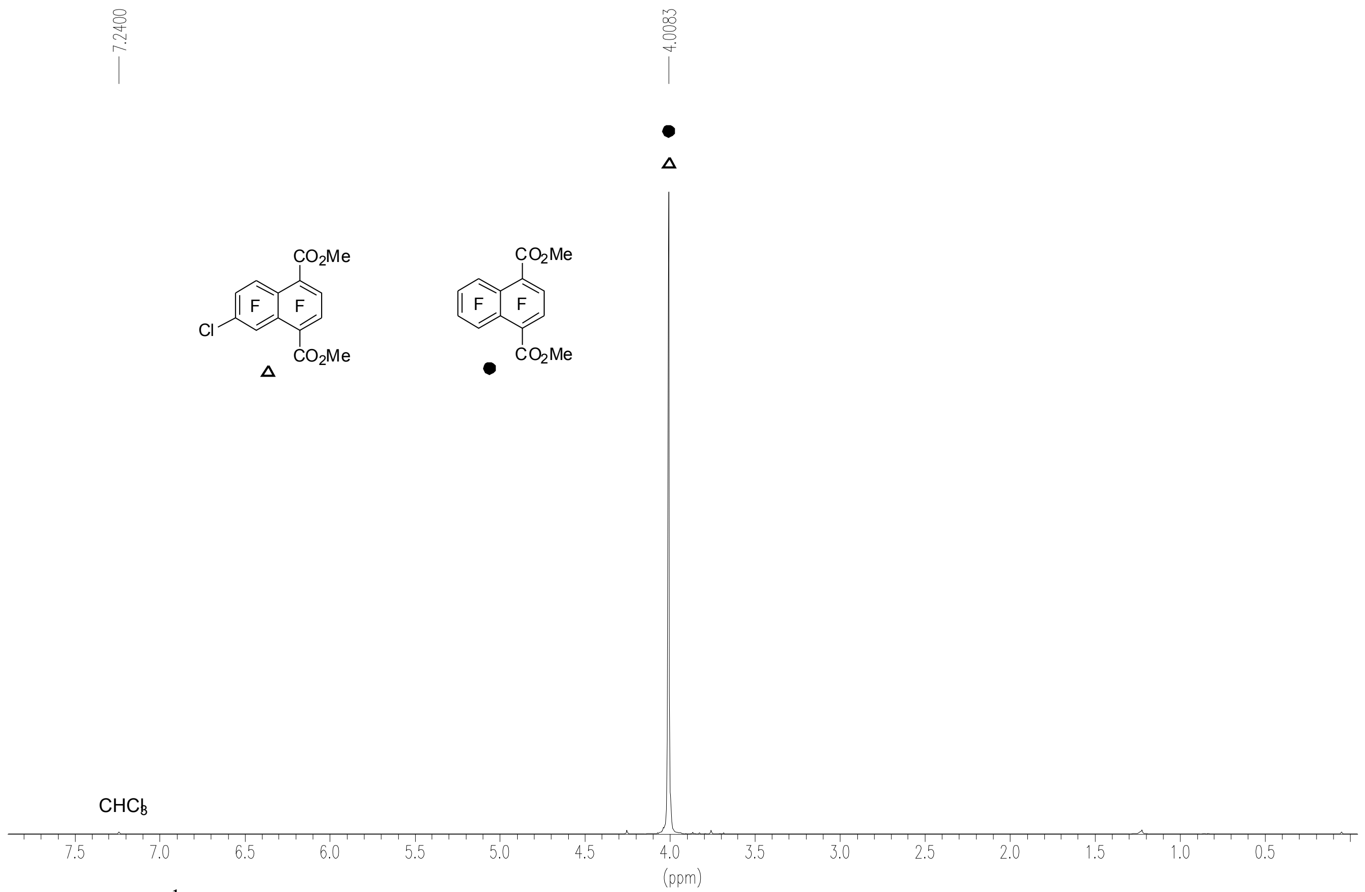

Figure S78. ^{1}H NMR spectrum of a mixture of **53’** and **39’** ($CDCl_3$).

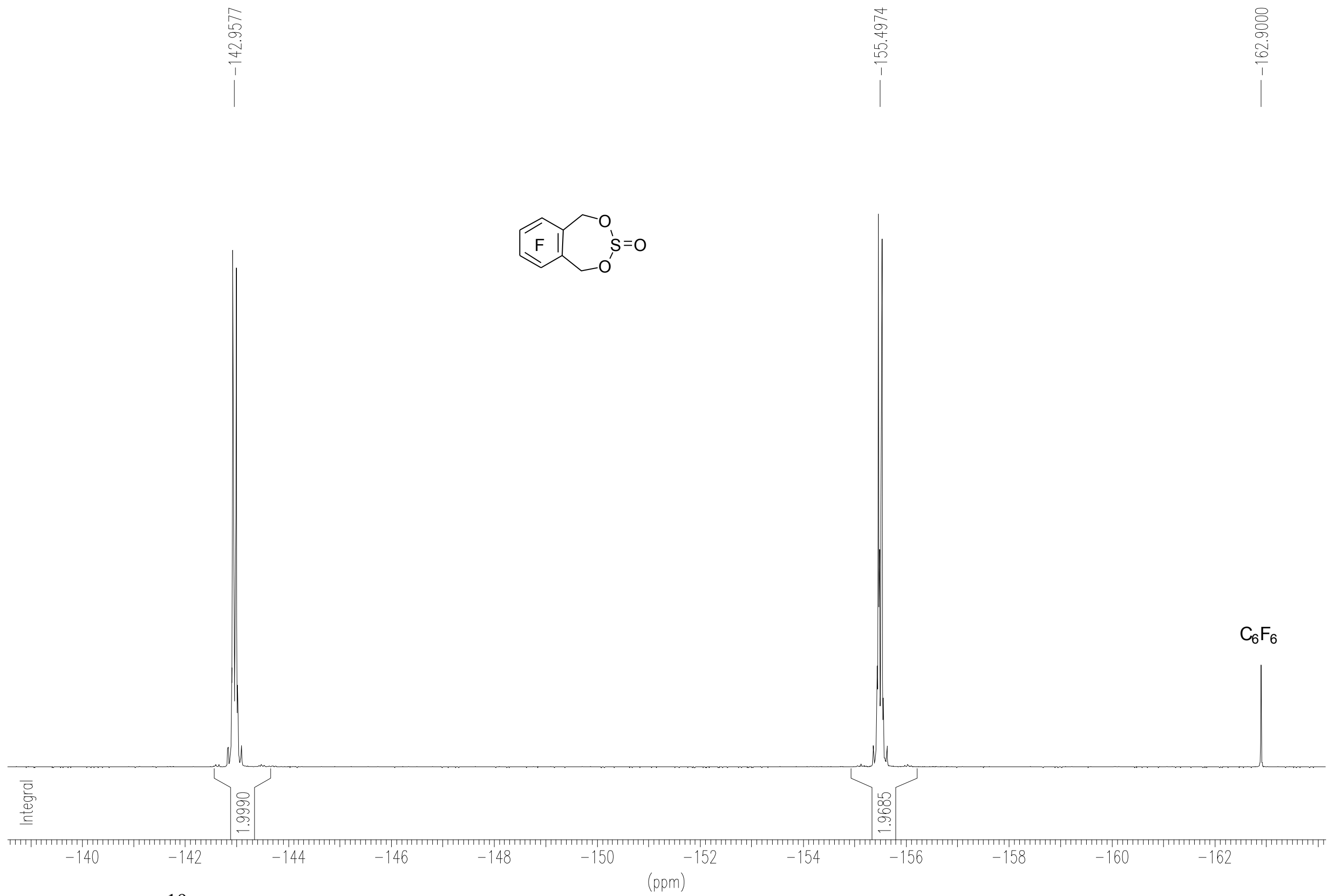

Figure S79. ^{19}F NMR spectrum of **44** ($CDCl_3$).

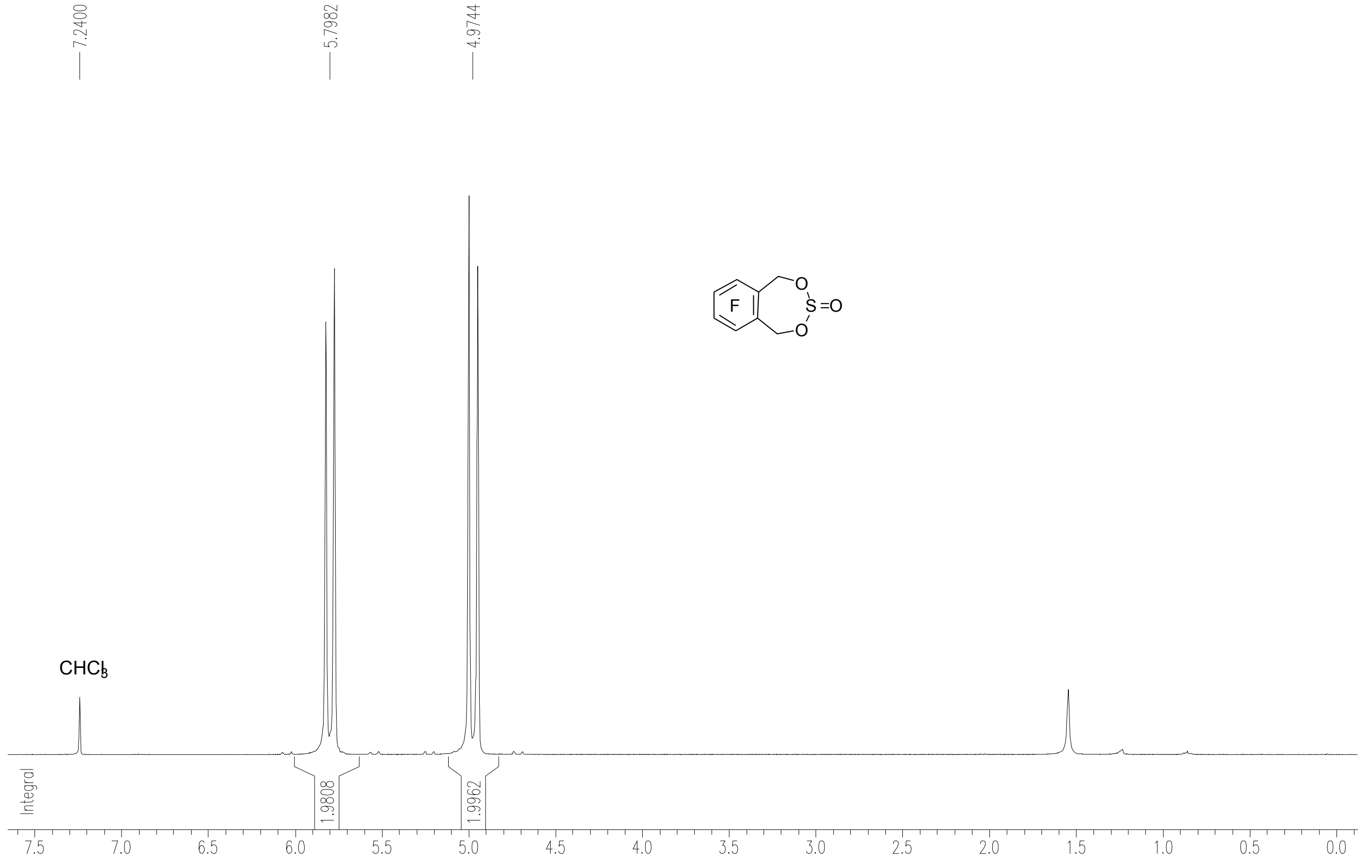

Figure S80. ^{1}H NMR spectrum of **44** ($CDCl_3$).
